# Medical treatment of eosinophilic esophagitis

**DOI:** 10.1002/14651858.CD004065.pub4

**Published:** 2023-07-20

**Authors:** James P Franciosi, Morris Gordon, Vassiliki Sinopoulou, Evan S Dellon, Sandeep K Gupta, Craig C Reed, Carolina Gutiérrez-Junquera, Rajitha D Venkatesh, Elizabeth A Erwin, Abdullah Egiz, Assem Elleithy, Edward B Mougey

**Affiliations:** Division of Gastroenterology, Hepatology, and NutritionNemours Children's HospitalOrlandoFLUSA; College of MedicineUniversity of Central FloridaOrlandoUSA; School of MedicineUniversity of Central LancashirePrestonUK; Department of Medicine, Division of Gastroenterology and Hepatology, Center for Esophageal Diseases and SwallowingUniversity of North Carolina School of MedicineChapel HillNCUSA; Division of Pediatric Gastroenterology, Hepatology and NutritionUniversity of Illinois College of Medicine at Peoria and Children's Hospital of IllinoisPeoriaINUSA; Pediatric GastroenterologyUniversity Hospital Puerta de Hierro Majadahonda. Autonomous University of MadridMadridSpain; Pediatrics, Gastroenterology & Hepatology & NutritionNationwide Children's HospitalColumbusOHUSA; Pediatric AllergyNationwide Children's HospitalColumbusOHUSA; Clinical Pharmacogenomics and Translational ResearchNemours Children's Health SystemJacksonvilleFLUSA

**Keywords:** Adult, Child, Humans, Adrenal Cortex Hormones, Adrenal Cortex Hormones/therapeutic use, Biological Products, Chronic Disease, Eosinophilic Esophagitis, Eosinophilic Esophagitis/drug therapy, Proton Pump Inhibitors, Proton Pump Inhibitors/therapeutic use, Randomized Controlled Trials as Topic, Remission Induction

## Abstract

**Background:**

Eosinophilic esophagitis (EoE) is a chronic antigen‐mediated eosinophilic inflammatory disease isolated to the esophagus. As a clinicopathologic disorder, a diagnosis of EoE requires a constellation of clinical symptoms of esophageal dysfunction and histologic findings (at least 15 eosinophils/high‐powered microscope field (eos/hpf)). Current guidelines no longer require the failure of response to proton pump inhibitor medications to establish a diagnosis of EoE, but continue to suggest the exclusion of other etiologies of esophageal eosinophilia.

The treatment goals for EoE are improvement in clinical symptoms, resolution of esophageal eosinophilia and other histologic abnormalities, endoscopic improvement, improved quality of life, improved esophageal function, minimized adverse effects of treatment, and prevention of disease progression and subsequent complications.

Currently, there is no cure for EoE, making long‐term treatment necessary. Standard treatment modalities include dietary modifications, esophageal dilation, and pharmacologic therapy. Effective pharmacologic therapies include corticosteroids, rapidly emerging biological therapies, and proton pump inhibitor medications.

**Objectives:**

To evaluate the efficacy and safety of medical interventions for people with eosinophilic esophagitis.

**Search methods:**

We searched CENTRAL, MEDLINE, Embase, ClinicalTrials.gov, and WHO ICTRP to 3 March 2023.

**Selection criteria:**

Randomized controlled trials (RCTs) comparing any medical intervention or food elimination diet for the treatment of eosinophilic esophagitis, either alone or in combination, to any other intervention (including placebo).

**Data collection and analysis:**

Pairs of review authors independently selected studies and conducted data extraction and risk of bias assessment. We expressed outcomes as a risk ratio (RR) and as the mean or standardized mean difference (MD/SMD) with 95% confidence interval (CI). We assessed the certainty of the evidence using GRADE.

Our primary outcomes were: clinical, histological, and endoscopic improvement, and withdrawals due to adverse events. Secondary outcomes were: serious and total adverse events, and quality of life.

**Main results:**

We included 41 RCTs with 3253 participants. Eleven studies included pediatric patients while the rest recruited both children and adults. Four studies were in patients with inactive disease while the rest were in patients with active disease. We identified 19 intervention comparisons. In this abstract we present the results of the primary outcomes for the two main comparisons: corticosteroids versus placebo and biologics versus placebo, based on the prespecified outcomes defined of the primary studies.

Fourteen studies compared corticosteroids to placebo for induction of remission and the risk of bias for these studies was mostly low.

Corticosteroids may lead to slightly better clinical improvement (20% higher), measured dichotomously (risk ratio (RR) 1.74, 95% CI 1.08 to 2.80; 6 studies, 583 participants; number needed to treat for an additional beneficial outcome (NNTB) = 4; low certainty), and may lead to slightly better clinical improvement, measured continuously (standard mean difference (SMD) 0.51, 95% CI 0.17 to 0.85; 5 studies, 475 participants; low certainty).

Corticosteroids lead to a large histological improvement (63% higher), measured dichotomously (RR 11.94, 95% CI 6.56 to 21.75; 12 studies, 978 participants; NNTB = 3; high certainty), and may lead to histological improvement, measured continuously (SMD 1.42, 95% CI 1.02 to 1.82; 5 studies, 449 participants; low certainty).

Corticosteroids may lead to little to no endoscopic improvement, measured dichotomously (RR 2.60, 95% CI 0.82 to 8.19; 5 studies, 596 participants; low certainty), and may lead to endoscopic improvement, measured continuously (SMD 1.33, 95% CI 0.59 to 2.08; 5 studies, 596 participants; low certainty).

Corticosteroids may lead to slightly fewer withdrawals due to adverse events (RR 0.64, 95% CI 0.43 to 0.96; 14 studies, 1032 participants; low certainty).

Nine studies compared biologics to placebo for induction of remission.

Biologics may result in little to no difference in clinical improvement, measured dichotomously (RR 1.14, 95% CI 0.85 to 1.52; 5 studies, 410 participants; low certainty), and may result in better clinical improvement, measured continuously (SMD 0.50, 95% CI 0.22 to 0.78; 7 studies, 387 participants; moderate certainty).

Biologics result in better histological improvement (55% higher), measured dichotomously (RR 6.73, 95% CI 2.58 to 17.52; 8 studies, 925 participants; NNTB = 2; moderate certainty). We could not draw conclusions for this outcome when measured continuously (SMD 1.01, 95% CI 0.36 to 1.66; 6 studies, 370 participants; very low certainty).

Biologics may result in little to no difference in endoscopic improvement, measured dichotomously (effect not estimable, low certainty). We cannot draw conclusions for this outcome when measured continuously (SMD 2.79, 95% CI 0.36 to 5.22; 1 study, 11 participants; very low certainty).

There may be no difference in withdrawals due to adverse events (RR 1.55, 95% CI 0.88 to 2.74; 8 studies, 792 participants; low certainty).

**Authors' conclusions:**

Corticosteroids (as compared to placebo) may lead to clinical symptom improvement when reported both as dichotomous and continuous outcomes, from the primary study definitions. Corticosteroids lead to a large increase in histological improvement (dichotomous outcome) and may increase histological improvement (continuous outcome) when compared to placebo. Corticosteroids may or may not increase endoscopic improvement (depending on whether the outcome is measured dichotomously or continuously). Withdrawals due to adverse events (dichotomous outcome) may occur less frequently when corticosteroids are compared to placebo.

Biologics (as compared to placebo) may not lead to clinical symptom improvement when reported as a dichotomous outcome and may lead to an increase in clinical symptom improvement (as a continuous outcome), from the primary study definitions. Biologics lead to a large increase in histological improvement when reported as a dichotomous outcome, but this is uncertain when reported as a continuous outcome, as compared to placebo. Biologics may not increase endoscopic improvement (dichotomous outcome), but this is uncertain when measured as a continuous outcome. Withdrawals due to adverse events as a dichotomous outcome may occur as frequently when biologics are compared to placebo.

## Plain language summary

**Medical treatments for eosinophilic esophagitis**

**Key messages**

We found that while corticosteroids may improve patients' symptoms, they certainly reduce the amount of allergic cells (eosinophils) and they may improve what the disease looks like under visual inspection (endoscopy), when compared to placebo, for children and adults with eosinophilic esophagitis. They may be just as safe as a placebo (dummy treatment).

We found that biologics (a type of treatment that uses substances made from living organisms to treat disease) may improve patients' symptoms, that they certainly reduce the amount of allergic cells, and that they may be no different in terms of what the disease looks like under visual inspection, when compared to placebo, for children and adults with eosinophilic esophagitis. They may be just as safe as a placebo.

**What is eosinophilic esophagitis?**

Eosinophilic esophagitis is a long‐term allergic condition in which the esophagus becomes inflamed (sore), which can lead to difficulty swallowing, vomiting, heartburn, and chest and stomach pain. Particles in foods or the air cause the immune system to have an allergic reaction and produce immune cells, which are called eosinophils. These build up in the esophagus, the tube that connects the mouth with the stomach. Eosinophilic esophagitis was first identified in the 1990s and since then it has been recognized as a major digestive illness. It is not known what causes it, but it might be related to genetics combined with environmental triggers. People with eosinophilic esophagitis tend to have other allergies as well. Currently, there is no cure for eosinophilic esophagitis, making long‐term treatment necessary. Standard treatments include diets, stretching of the esophagus (dilation), and drugs such as corticosteroids, biological medications, and proton pump inhibitor medications.

**What did we want to find out?**

We wanted to find out if the available medical treatments for eosinophilic esophagitis work for improving patients' symptoms, reducing the amount of allergic cells when measured under a microscope, and improving what the disease looks like under visual examination. We also wanted to find out how safe they are and if they improve quality of life.

**What did we do?**

We searched for randomized controlled trials (studies where people are assigned to one of two or more treatment groups using a random method) comparing any medical treatment for eosinophilic esophagitis with any other medical treatment, in both adults and children.  **What did we find?**

We found 41 studies with 3253 participants. Eleven studies were in children only while the rest were in a mix of children and adults. We identified 19 comparisons. In this summary, we present the results of the two main comparisons: corticosteroids compared to placebo and biologics compared to placebo.

We found that corticosteroids may be better than placebo at improving patients' symptoms. We are highly certain that corticosteroids are better than placebo at reducing the amount of eosinophils (allergic cells) when measured under a microscope. Corticosteroids may be better than placebo at improving what the disease looks like under visual examination (endoscopy). We also found that people taking corticosteroids may be less likely to leave a study due to unwanted or harmful effects (side effects), and that they probably experience a similar number of both serious side effects and side effects in total, compared to placebo. There may be no difference between corticosteroids and placebo in the improvement of quality of life.

We found that biologics may be better than placebo at improving patients' symptoms. It is likely that biologics are better than placebo at reducing the amount of allergic cells when measured under a microscope. Biologics may be no different to placebo at improving what the disease looks like under visual examination. We also found that people on biologics may be equally likely to leave a study due to side effects, or have serious side effects, and may experience similar numbers of total side effects, compared to placebo. There may be no difference between biologics and placebo in the improvement of quality of life.

**What are the limitations of the evidence?**

The evidence in children only was quite limited and we do not know if the conclusions above can definitely apply to children specifically. Another limitation of the evidence is that the outcomes were measured in many different ways, which may have weakened our conclusions. Other treatments used by the participants were also something that varied a lot between people, and may have affected our conclusions. Finally, we were limited in the conclusions we could make about the effects of sex, age, extent of disease, dosage, and type of corticosteroid or biologic.

**How up‐to‐date is this review?**

This review is up‐to‐date to 3 March 2023.

## Summary of findings

**Summary of findings 1:** Corticosteroids compared to placebo for induction of remission

**Corticosteroids compared to placebo for induction of remission**						
**Patient or population:** active EoE patients **Setting:** medical centers **Intervention:** corticosteroids **Comparison:** placebo						
**Outcomes**	**№ of participants** **(studies)**	**Certainty of the evidence (GRADE)**	**Relative effect (95% CI)**	Anticipated absolute effects^*^ (95% CI)		**Comments**
				Risk with placebo**	**Risk difference with corticosteroids**	
Clinical improvement (dichotomous) 2 to 12 weeks	583 (6 studies)	⊕⊕⊝⊝ **Low^a^**	RR 1.74 (1.08 to 2.80)	Study population		—
				350 per 1000	259 more per 1000 (28 more to 378 more)	
Clinical improvement (continuous) 2 to 12 weeks	475 (5 studies)	⊕⊕⊝⊝ **Low^a^**	—	—	SMD 0.51 higher (0.17 higher to 0.85 higher)	As a rule of thumb, 0.2 SMD represents a small difference, 0.5 a moderate, and 0.8 a large effect.
Histological improvement (dichotomous) 2 to 12 weeks	978 (12 studies)	⊕⊕⊕⊕ **High**	RR 11.94 (6.56 to 21.75)	Study population		NNTB = 3
				31 per 1000	339 more per 1000 (172 more to 643 more)	
Histological improvement (continuous) 2 to 12 weeks	449 (5 studies)	⊕⊕⊝⊝ **Low^b^**	—	—	SMD 1.42 higher (1.02 higher to 1.82 higher)	As a rule of thumb, 0.2 SMD represents a small difference, 0.5 a moderate, and 0.8 a large effect.
Endoscopic improvement (dichotomous) 6 to 12 weeks	102 (3 studies)	⊕⊕⊝⊝ **Low^c^**	RR 2.60 (0.82 to 8.19)	Study population		—
				136 per 1000	218 more per 1000 (24 less to 978 more)	
Endoscopic improvement (continuous) 6 to 12 weeks	596 (5 studies)	⊕⊕⊝⊝ **Low^d^**	—	—	SMD 1.33 higher (0.59 higher to 2.08 higher)	As a rule of thumb, 0.2 SMD represents a small difference, 0.5 a moderate, and 0.8 a large effect.
Withdrawals due to adverse events (2 to 12 weeks)	1032 (14 studies)	⊕⊕⊝⊝ **Low^e^**	RR 0.64 (0.43 to 0.96)	Study population		—
				124 per 1000	45 fewer per 1000 (71 fewer to 5 fewer)	
*The risk in the intervention group (and its 95% confidence interval) is based on the assumed risk in the comparison group and the **relative effect** of the intervention (and its 95% CI). ** The risk for the control intervention has been calculated by dividing the number of cases to the number of randomized participants, using the numbers of our analyses. The risk for the comparison intervention has been calculated by multiplying the control risk with the RR and CI limits. The risk difference has been calculated by subtracting the control risk from the comparison intervention risk. **CI:** confidence interval; **EoE:** eosinophilic esophagitis; **MD:** mean difference; **NNTB:** number needed to treat for an additional beneficial outcome; **RR:** risk ratio; **SMD:** standardized mean difference						
**GRADE Working Group grades of evidence** **High certainty:** we are very confident that the true effect lies close to that of the estimate of the effect. **Moderate certainty:** we are moderately confident in the effect estimate: the true effect is likely to be close to the estimate of the effect, but there is a possibility that it is substantially different. **Low certainty:** our confidence in the effect estimate is limited: the true effect may be substantially different from the estimate of the effect. **Very low certainty:** we have very little confidence in the effect estimate: the true effect is likely to be substantially different from the estimate of effect.						

^a^Downgraded once due to inconsistency (I² = 72% and I² = 55% respectively) and once due to imprecision. ^b^Downgraded once due to inconsistency (I² = 50%) and once due to risk of bias across multiple domains. ^c^Downgraded twice due to serious imprecision. ^d^Downgraded twice due to serious inconsistency (I² = 92%). ^e^Downgraded once due to imprecision and once due to risk of bias across multiple domains.

**Summary of findings 2:** Corticosteroids compared to placebo for maintenance of remission

**Corticosteroids compared to placebo for maintenance of remission**						
**Patient or population:** inactive EoE patients **Setting:** medical centers **Intervention:** corticosteroids **Comparison:** placebo						
**Outcomes**	**№ of participants** **(studies)**	**Certainty of the evidence (GRADE)**	**Relative effect (95% CI)**	Anticipated absolute effects^*^ (95% CI)		**Comments**
				**Risk with placebo**	**Risk difference with corticosteroids**	
Clinical improvement (dichotomous) 12 to 48 weeks	252 (2 studies)	⊕⊝⊝⊝ **Very low^a^**	RR 2.17 (0.75 to 6.27)	Study population		—
				297 per 1000	347 more per 1000 (74 fewer to 1000 more)	
Clinical improvement (continuous) 12 to 50 weeks	269 (3 studies)	⊕⊝⊝⊝ **Very low^a^**	—	—	SMD 0.51 higher (0.49 lower to 1.52 higher)	As a rule of thumb, 0.2 SMD represents a small difference, 0.5 a moderate, and 0.8 a large effect.
Histological improvement (dichotomous) 12 to 50 weeks	280 (3 studies)	⊕⊕⊕⊝ **Moderate^b^**	RR 4.58 (1.66 to 12.62)	Study population		NNTB = 3
				133 per 1000	476 more per 1000 (88 more to 1000 more)	
Histological improvement (continuous) 12 to 50 weeks	269 (3 studies)	⊕⊕⊕⊝ **Moderate^c^**	—	—	SMD 1.26 higher (0.74 higher to 1.78 higher)	As a rule of thumb, 0.2 SMD represents a small difference, 0.5 a moderate, and 0.8 a large effect.
Endoscopic improvement at study endpoint (dichotomous)	—	—	—	—	—	No data
Endoscopic improvement (continuous) 12 to 48 weeks	240 (2 studies)	⊕⊝⊝⊝ **Very low^a^**	—	—	SMD 1.34 higher (0.27 lower to 2.95 higher)	As a rule of thumb, 0.2 SMD represents a small difference, 0.5 a moderate, and 0.8 a large effect.
Withdrawals due to adverse events 12 to 50 weeks	280 (3 studies)	⊕⊕⊝⊝ **Low^d^**	RR 0.37 (0.16 to 0.87)	Study population		—
				552 per 1000	348 fewer per 1000 (464 fewer to 72 fewer)	
*The risk in the intervention group (and its 95% confidence interval) is based on the assumed risk in the comparison group and the **relative effect** of the intervention (and its 95% CI). ** The risk for the control intervention has been calculated by dividing the number of cases to the number of randomized participants, using the numbers of our analyses. The risk for the comparison intervention has been calculated by multiplying the control risk with the RR and CI limits. The risk difference has been calculated by subtracting the control risk from the comparison intervention risk. **CI:** confidence interval; **EoE:** eosinophilic esophagitis; **MD:** mean difference; **NNTB:** number needed to treat for an additional beneficial outcome; **RR:** risk ratio; **SMD:** standardized mean difference						
**GRADE Working Group grades of evidence** **High certainty:** we are very confident that the true effect lies close to that of the estimate of the effect. **Moderate certainty:** we are moderately confident in the effect estimate: the true effect is likely to be close to the estimate of the effect, but there is a possibility that it is substantially different. **Low certainty:** our confidence in the effect estimate is limited: the true effect may be substantially different from the estimate of the effect. **Very low certainty:** we have very little confidence in the effect estimate: the true effect is likely to be substantially different from the estimate of effect.						

^a^Downgraded twice due to serious inconsistency (I² = 97%, I² = 91%, and I² = 95% respectively) and imprecision. ^b^Downgraded once due to imprecision ^c^Downgraded once due to inconsistency (I² = 60%). ^d^Downgraded once due to inconsistency (I² = 69%) and once due to imprecision.

**Summary of findings 3:** Biologics compared to placebo for induction of remission

**Biologics compared to placebo for induction of remission**						
**Patient or population:** active EoE patients **Setting:** medical centers **Intervention:** biologics **Comparison:** placebo						
**Outcomes**	**№ of participants** **(studies)**	**Certainty of the evidence (GRADE)**	**Relative effect (95% CI)**	Anticipated absolute effects^*^ (95% CI)		**Comments**
				**Risk with placebo**	**Risk difference with biologics**	
Clinical improvement (dichotomous) 12 to 44 weeks	410 (5 studies)	⊕⊕⊝⊝ **Low^a^**	RR 1.14 (0.85 to 1.52)	Study population		—
				504 per 1000	71 more per 1000 (76 fewer to 262 more)	
Clinical improvement (continuous) 12 to 24 weeks	387 (7 studies)	⊕⊕⊕⊝ **Moderate^b^**	—	—	SMD 0.50 higher (0.22 higher to 0.78 higher)	As a rule of thumb, 0.2 SMD represents a small difference, 0.5 a moderate, and 0.8 a large effect.
Histological improvement (dichotomous) 12 to 44 weeks	925 (8 studies)	⊕⊕⊕⊝ **Moderate^b^**	RR 6.73 (2.58 to 17.52)	Study population		NNTB = 2
				115 per 1000	659 more (182 more to 1000 more)	
Histological improvement (continuous)	370 (6 studies)	⊕⊝⊝⊝ **Very low^c^**	—	—	SMD 1.01 higher (0.36 higher to 1.66 higher)	As a rule of thumb, 0.2 SMD represents a small difference, 0.5 a moderate, and 0.8 a large effect.
Endoscopic improvement (dichotomous) 13 weeks	11 (1 study)	⊕⊕⊝⊝ **Low^d^**	Not estimable	Study population		Both groups had zero patients with endoscopic improvement.
				Not estimable	Not estimable	
Endoscopic improvement (continuous) 12 to 24 weeks	197 (3 studies)	⊕⊝⊝⊝ **Very low^c^**	—	—	SMD 2.79 higher (0.36 higher to 5.22 higher)	As a rule of thumb, 0.2 SMD represents a small difference, 0.5 a moderate, and 0.8 a large effect.
Withdrawals due to adverse events 12 to 44 weeks	792 (8 studies)	⊕⊕⊝⊝ **Low^d^**	RR 1.55 (0.88 to 2.74)	Study population		—
				58 per 1000	32 more per 1000 (7 fewer to 101 more)	
*The risk in the intervention group (and its 95% confidence interval) is based on the assumed risk in the comparison group and the **relative effect** of the intervention (and its 95% CI). ** The risk for the control intervention has been calculated by dividing the number of cases to the number of randomized participants, using the numbers of our analyses. The risk for the comparison intervention has been calculated by multiplying the control risk with the RR and CI limits. The risk difference has been calculated by subtracting the control risk from the comparison intervention risk. **CI:** confidence interval; **EoE:** eosinophilic esophagitis; **MD:** mean difference; **NNTB:** number needed to treat for an additional beneficial outcome; **RR:** risk ratio; **SMD:** standardized mean difference						
**GRADE Working Group grades of evidence** **High certainty:** we are very confident that the true effect lies close to that of the estimate of the effect. **Moderate certainty:** we are moderately confident in the effect estimate: the true effect is likely to be close to the estimate of the effect, but there is a possibility that it is substantially different. **Low certainty:** our confidence in the effect estimate is limited: the true effect may be substantially different from the estimate of the effect. **Very low certainty:** we have very little confidence in the effect estimate: the true effect is likely to be substantially different from the estimate of effect.						

^a^Downgraded twice due to imprecision. ^b^Downgraded once due to imprecision. ^c^Downgraded twice due to serious inconsistency (I² = 83% and I² = 97% respectively) and once due to imprecision. ^d^Downgraded twice due to serious imprecision.

**Summary of findings 4:** Cromolyn sodium compared to placebo

**Cromolyn sodium compared to placebo**						
**Patient or population:** active EoE pediatric patients **Setting:** medical center **Intervention:** cromolyn sodium **Comparison:** placebo						
**Outcomes**	**№ of participants** **(studies)**	**Certainty of the evidence (GRADE)**	**Relative effect (95% CI)**	Anticipated absolute effects^*^ (95% CI)		**Comments**
				**Risk with placebo**	**Risk difference with cromolyn sodium**	
Clinical improvement (dichotomous)	—	—	—	—	—	No data
Clinical improvement (continuous) 8 weeks	14 (1 study)	⊕⊕⊝⊝ **Low^a^**	—	—	MD 4.70 higher (12.09 lower to 21.49 higher)	Measured on the Pediatric Eosinophilic Esophagitis Symptom Score (PEESS)
Histological improvement (dichotomous)	—	—	—	—	—	No data
Histological improvement (continuous) 8 weeks	15 (1 study)	⊕⊕⊝⊝ **Low^a^**	—	—	MD 14.20 higher (36.90 lower to 65.30 higher)	Measured as change in peak eos/hpf from baseline
Endoscopic improvement (dichotomous)	—	—	—	—	—	No data
Endoscopic improvement (continuous)	—	—	—	—	—	No data
Withdrawals due to adverse events at 8 weeks	16 (1 study)	⊕⊕⊝⊝ **Low^a^**	RR 0.27 (0.01 to 5.70)	Study population		—
				143 per 1000	104 less per 1000 (141 less to 672 more)	—
*The risk in the intervention group (and its 95% confidence interval) is based on the assumed risk in the comparison group and the **relative effect** of the intervention (and its 95% CI). ** The risk for the control intervention has been calculated by dividing the number of cases to the number of randomized participants, using the numbers of our analyses. The risk for the comparison intervention has been calculated by multiplying the control risk with the RR and CI limits. The risk difference has been calculated by subtracting the control risk from the comparison intervention risk. **CI:** confidence interval; **EoE:** eosinophilic esophagitis; **eos/hpf:** eosinophils/high‐power field; **MD:** mean difference; **NNTB:** number needed to treat for an additional beneficial outcome; **RR:** risk ratio; **SMD:** standardized mean difference						
**GRADE Working Group grades of evidence** **High certainty:** we are very confident that the true effect lies close to that of the estimate of the effect. **Moderate certainty:** we are moderately confident in the effect estimate: the true effect is likely to be close to the estimate of the effect, but there is a possibility that it is substantially different. **Low certainty:** our confidence in the effect estimate is limited: the true effect may be substantially different from the estimate of the effect. **Very low certainty:** we have very little confidence in the effect estimate: the true effect is likely to be substantially different from the estimate of effect.						

^a^Downgraded twice due to serious imprecision.

**Summary of findings 5:** PGD2R antagonist OC000459 compared to placebo

**PGD2R antagonist OC000459 compared to placebo**						
**Patient or population:** active EoE patients **Setting:** medical center **Intervention:** PGD2R antagonist OC000459 **Comparison:** placebo						
**Outcomes**	**№ of participants** **(studies)**	**Certainty of the evidence (GRADE)**	**Relative effect (95% CI)**	Anticipated absolute effects^*^ (95% CI)		**Comments**
				**Risk with placebo**	**Risk difference with PGD2R antagonist OC000459**	
Clinical improvement (dichotomous)	—	—	—	—		No data
Clinical improvement (continuous) 8 weeks	26 (1 study)	⊕⊝⊝⊝ **Very low^a^**	—	—	MD 1.06 lower (6.80 lower to 4.68 higher)	Measured as combined post‐treatment means of several questionnaires
Histological improvement (dichotomous)	—	—	—	—	—	No data
Histological improvement (continuous) 8 weeks	26 (1 study)	⊕⊝⊝⊝ **Very low^a^**	—	—	MD 26.21 higher (23.78 lower to 76.20 higher)	Measured as post‐treatment eosinophil load
Endoscopic improvement (dichotomous)	—	—	—	—	—	No data
Endoscopic improvement (continuous) 8 weeks	26 (1 study)	⊕⊝⊝⊝ **Very low^a^**	—	—	MD 0.49 lower (2.05 lower to 1.07 higher)	Measured on a 10‐point visual analogue scale
Withdrawals due to adverse events	26 (1 study)	⊕⊝⊝⊝ **Very low^a^**	Not estimable	Study population		—
				0 per 1000	0 per 1000	
*The risk in the intervention group (and its 95% confidence interval) is based on the assumed risk in the comparison group and the **relative effect** of the intervention (and its 95% CI). ** The risk for the control intervention has been calculated by dividing the number of cases to the number of randomized participants, using the numbers of our analyses. The risk for the comparison intervention has been calculated by multiplying the control risk with the RR and CI limits. The risk difference has been calculated by subtracting the control risk from the comparison intervention risk. **CI:** confidence interval; **EoE:** eosinophilic esophagitis; **MD:** mean difference; **NNTB:** number needed to treat for an additional beneficial outcome; **RR:** risk ratio; **SMD:** standardized mean difference						
**GRADE Working Group grades of evidence** **High certainty:** we are very confident that the true effect lies close to that of the estimate of the effect. **Moderate certainty:** we are moderately confident in the effect estimate: the true effect is likely to be close to the estimate of the effect, but there is a possibility that it is substantially different. **Low certainty:** our confidence in the effect estimate is limited: the true effect may be substantially different from the estimate of the effect. **Very low certainty:** we have very little confidence in the effect estimate: the true effect is likely to be substantially different from the estimate of effect.						

^a^Downgraded twice due to serious imprecision and once due to risk of bias for unclear allocation concealment and blinding of outcome assessment.

**Summary of findings 6:** Swallowed fluticasone compared to oral prednisone

**Swallowed fluticasone compared to oral prednisone**						
**Patient or population:** active EoE pediatric patients **Setting:** medical center **Intervention:** swallowed fluticasone **Comparison:** oral prednisone						
**Outcomes**	**№ of participants** **(studies)**	**Certainty of the evidence (GRADE)**	**Relative effect (95% CI)**	Anticipated absolute effects^*^ (95% CI)		**Comments**
				**Risk with comparator**	**Risk difference with corticosteroid**	
Clinical improvement at 4 weeks (dichotomous)	80 (1 study)	⊕⊝⊝⊝ **Very low^a^**	RR 1.09 (0.90 to 1.33)	Study population		—
				800 per 1000	72 more per 1000 (80 fewer to 200 more)	
Clinical improvement at 4 weeks (continuous)	—	—	—	—	—	No data
Histological improvement at 4 weeks (dichotomous)	80 (1 study)	⊕⊝⊝⊝ **Very low^a^**	RR 1.1 (0.87 to 1.38)	Study population		—
				750 per 1000	75 more per 1000 (98 fewer to 285 more)	
Histological improvement at 4 weeks (continuous)	68 (1 study)	⊕⊝⊝⊝ **Very low^a^**	—	—	MD 4.45 lower (9.08 lower to 0.18 higher)	Measured as mean peak eosinophils
Endoscopic improvement at 4 weeks (dichotomous)	80 (1 study)	⊕⊝⊝⊝ **Very low^a^**	RR 1.13 (0.91 to 1.41)	Study population		—
				750 per 1000	97 more per 1000 (68 fewer to 308 more)	
Endoscopic improvement (continuous)	—	—	—	—	—	No data
Withdrawals due to adverse events at 4 weeks	80 (1 study)	⊕⊝⊝⊝ **Very low^a^**	RR 0.50 (0.16 to 1.53)	Study population		—
				200 per 1000	100 fewer per 1000 (168 fewer to 106 more)	
*The risk in the intervention group (and its 95% confidence interval) is based on the assumed risk in the comparison group and the **relative effect** of the intervention (and its 95% CI). ** The risk for the control intervention has been calculated by dividing the number of cases to the number of randomized participants, using the numbers of our analyses. The risk for the comparison intervention has been calculated by multiplying the control risk with the RR and CI limits. The risk difference has been calculated by subtracting the control risk from the comparison intervention risk. **CI:** confidence interval; **EoE:** eosinophilic esophagitis; **MD:** mean difference; **NNTB:** number needed to treat for an additional beneficial outcome; **RR:** risk ratio; **SMD:** standardized mean difference						
**GRADE Working Group grades of evidence** **High certainty:** we are very confident that the true effect lies close to that of the estimate of the effect. **Moderate certainty:** we are moderately confident in the effect estimate: the true effect is likely to be close to the estimate of the effect, but there is a possibility that it is substantially different. **Low certainty:** our confidence in the effect estimate is limited: the true effect may be substantially different from the estimate of the effect. **Very low certainty:** we have very little confidence in the effect estimate: the true effect is likely to be substantially different from the estimate of effect.						

^a^Downgraded twice due to serious imprecision and once due to risk of bias for blinding of participants and personnel.

**Summary of findings 7:** Oral viscous budesonide compared to swallowed fluticasone

**Oral viscous budesonide compared to swallowed fluticasone**						
**Patient or population:** active EoE patients **Setting:** medical center **Intervention:** oral viscous budesonide **Comparison:** swallowed fluticasone						
**Outcomes**	**№ of participants** **(studies)**	**Certainty of the evidence (GRADE)**	**Relative effect (95% CI)**	Anticipated absolute effects^*^ (95% CI)		**Comments**
				Risk with s**wallowed fluticasone**	Risk difference with o**ral viscous budesonide**	
Clinical improvement at study endpoint (dichotomous)	—	—	—	—	—	No data
Clinical improvement at 8 weeks (continuous)	84 (1 study)	⊕⊕⊝⊝ **Low^a^**	—	—	MD 0.6 lower (3.78 lower to 2.58 higher)	Measured on the Dysphagia Score Questionnaire
Histological improvement at study endpoint (dichotomous)	129 (1 study)	⊕⊕⊝⊝ **Low^a^**	RR 1.13 (0.84 to 1.51)	Study population		—
				547 per 1000	71 more per 1000 (88 fewer to 279 more)	—
Histological improvement at 8 weeks (continuous)	111 (1 study)	⊕⊕⊝⊝ **Low^a^**	—	—	MD 6.2 higher (5.63 lower to 18.03 higher)	Measured as eosinophils per high‐power field
Endoscopic improvement at study endpoint (dichotomous)	—	—	—	—	—	No data
Endoscopic improvement at 8 weeks (continuous)	111 (1 study)	⊕⊕⊝⊝ **Low^a^**	—	—	MD 0.7 higher (0.03 lower to 1.43 higher)	Measured on the endoscopic reference score
Withdrawals due to adverse events at 8 weeks	129 (1 study)	⊕⊕⊝⊝ **Low^a^**	RR 0.98 (0.42 to 2.32)	Study population		—
				141 per 1000	3 fewer per 1000 (82 fewer to 186 more)	—
*The risk in the intervention group (and its 95% confidence interval) is based on the assumed risk in the comparison group and the **relative effect** of the intervention (and its 95% CI). ** The risk for the control intervention has been calculated by dividing the number of cases to the number of randomized participants, using the numbers of our analyses. The risk for the comparison intervention has been calculated by multiplying the control risk with the RR and CI limits. The risk difference has been calculated by subtracting the control risk from the comparison intervention risk. **CI:** confidence interval; **EoE:** eosinophilic esophagitis; **MD:** mean difference; **NNTB:** number needed to treat for an additional beneficial outcome; **RR:** risk ratio; **SMD:** standardized mean difference						
**GRADE Working Group grades of evidence** **High certainty:** we are very confident that the true effect lies close to that of the estimate of the effect. **Moderate certainty:** we are moderately confident in the effect estimate: the true effect is likely to be close to the estimate of the effect, but there is a possibility that it is substantially different. **Low certainty:** our confidence in the effect estimate is limited: the true effect may be substantially different from the estimate of the effect. **Very low certainty:** we have very little confidence in the effect estimate: the true effect is likely to be substantially different from the estimate of effect.						

^a^Downgraded twice due to serious imprecision.

**Summary of findings 8:** Esomeprazole compared to fluticasone

**Esomeprazole compared to fluticasone**						
**Patient or population:** active EoE patients **Setting:** medical centers **Intervention:** esomeprazole **Comparison:** fluticasone						
**Outcomes**	**№ of participants** **(studies)**	**Certainty of the evidence (GRADE)**	**Relative effect (95% CI)**	Anticipated absolute effects^*^ (95% CI)		**Comments**
				Risk with f**luticasone**	Risk difference with e**someprazole**	
Clinical improvement (dichotomous)	—	—	—	—	—	No data
Clinical improvement at 8 weeks (continuous)	67 (2 studies)	⊕⊝⊝⊝ **Very low^a^**	—	—	SMD 0.28 higher (0.2 lower to 0.76 higher)	As a rule of thumb, 0.2 SMD represents a small difference, 0.5 a moderate, and 0.8 a large effect.
Histological improvement at 8 weeks (dichotomous)	72 (2 studies)	⊕⊝⊝⊝ **Very low^b^**	RR 1.62 (0.77 to 3.41)	Study population		—
				222 per 1000	151 more per 1000 (42 fewer to 551 more)	
Histological improvement at 8 weeks (continuous)	70 (2 studies)	⊕⊝⊝⊝ **Very low^b^**	—	—	SMD 0.28 higher (0.20 lower to 0.76 higher)	As a rule of thumb, 0.2 SMD represents a small difference, 0.5 a moderate, and 0.8 a large effect.
Endoscopic improvement (dichotomous)	—	—	—	—	—	The studies reported data on specific endoscopic findings, which can be found in Table 9.
Endoscopic improvement (continuous)	—	—	—	—	—	No data
Withdrawals due to adverse events at 8 weeks	72 (2 studies)	⊕⊝⊝⊝ **Very low^b^**	RR 0.95 (0.07 to 13.38)	Study population		—
				83 per 1000	4 fewer per 1000 (77 fewer to 1000 more)	
*The risk in the intervention group (and its 95% confidence interval) is based on the assumed risk in the comparison group and the **relative effect** of the intervention (and its 95% CI). ** The risk for the control intervention has been calculated by dividing the number of cases to the number of randomized participants, using the numbers of our analyses. The risk for the comparison intervention has been calculated by multiplying the control risk with the RR and CI limits. The risk difference has been calculated by subtracting the control risk from the comparison intervention risk. **CI:** confidence interval; **EoE:** eosinophilic esophagitis; **MD:** mean difference; **NNTB:** number needed to treat for an additional beneficial outcome; **RR:** risk ratio; **SMD:** standardized mean difference						
**GRADE Working Group grades of evidence** **High certainty:** we are very confident that the true effect lies close to that of the estimate of the effect. **Moderate certainty:** we are moderately confident in the effect estimate: the true effect is likely to be close to the estimate of the effect, but there is a possibility that it is substantially different. **Low certainty:** our confidence in the effect estimate is limited: the true effect may be substantially different from the estimate of the effect. **Very low certainty:** we have very little confidence in the effect estimate: the true effect is likely to be substantially different from the estimate of effect.						

^a^Downgraded once for inconsistency (I² = 81%), once for imprecision, and once for risk of bias ^b^Downgraded twice for serious imprecision and once for risk of bias for blinding of participants, personnel, and outcome assessment, and selective reporting.

**1:** Primary outcome ‐ endoscopic improvement

**Study ID**	**Endoscopic improvement system used**	**Continuous or dichotomous**	**Outcome data ‐ endoscopic improvement at study endpoint**
Alexander 2012	Any endoscopic findings yes/no Alexander 2012 Not validated	Dichotomous; endoscopic findings not seen	Resolution of all endoscopic findings of EoE was seen in 8.3% (1 of 12) of placebo‐treated patients who completed the study and who had an abnormal baseline esophagogastroduodenoscopy. In the fluticasone‐treated patients who completed the trial, resolution of pretreatment abnormal endoscopic findings was seen in 26.7% (4 of 15). Dichotomous (used for endoscopic dichotomous analysis): Fluticasone: 4/21 Placebo: 1/21
Alexander 2017	Endoscopic findings described by the gastroenterologist Alexander 2017 No specific score was used	Not reported	No quantitative data were reported – "no differences in endoscopic findings of EoE"
Assa'ad 2011	Post hoc	Not reported	Not reported
Bhardwaj 2017	Not reported	Not reported	Not reported
Butz 2014	Not reported	Not reported	Not reported
Clayton 2014	Not reported	Not reported	Not reported
Dellon 2012	Morphological endoscopic findings described by the gastroenterologist No specific score was used	Dichotomous	No prespecified aggregate score, cannot use All budesonide, nebulized vs budesonide, oral viscous at end of trial: Rings: 10/11 vs 4/11 Narrowing: 6/11 vs 2/11 Stricture: 3/11 vs 2/11 Furrows: 6/11 vs 4/11 White plaques/exudates: 3/11 vs 3/11 Pallor/decreased vascularity: 2/11 vs 0/11 Crepe‐paper: 0/11 vs 0/11 Erosive esophagitis: 0/11 vs 0/11
Dellon 2017	**EREFS** Hirano 2013 Validated	Continuous Edema (0 to 2) Rings (0 to 3) Exudates (0 to 2) Furrows (0 to 2) Strictures (0 to 1)	Change in EREFS from baseline at end of trial, mean (SD) (used for endoscopic continuous analysis): Budesonide: –3.8 (3.9)/49 Placebo: 0.4 (6.7)/38
Dellon 2019	**EREFS** Hirano 2013 Validated	Continuous	EREFS at end of trial, mean (SD) (used for endoscopic continuous analysis): Budesonide: 2.1 (1.7)/56 Fluticasone: 2.8 (2.2)/55
Dellon 2021b	**EREFS** Hirano 2013 Validated	Continuous	From digitized figure 3C, change in EREFS at end of trial, mean (SD) (used for endoscopic continuous analysis): Budesonide: ‐0.99 (‐2.93)/24 Placebo: 0.60 (3.30)/21
Dellon 2022	**EREFS** Hirano 2013 Validated	Continuous	Change in EREFS from baseline at end of trial, mean (SD) (used for endoscopic continuous analysis): Dupilumab: ‐3.2 (0.41) n = 35/7 imputed Placebo: ‐0.3 (0.41) n = 26/13 imputed
Dellon 2022a	**EREFS** Hirano 2013 Validated	Continuous Endoscopic severity measured by the change from baseline in the EREFS (edema/rings/exudates/furrows/strictures (EoE Endoscopic Reference Score)) at week 12	Change in EREFS from baseline at end of trial, mean (SD) (used for endoscopic continuous analysis): APT‐1011 3 mg twice‐daily: –2.2 (1.84)/20 APT‐1011 3 mg at bedtime: –3.2 (2.28)/21 APT‐1011 1.5 mg twice‐daily: –2.9 (1.92)/22 APT‐1011 1.5 mg at bedtime: –2.4 (1.85)/21 All treatment arms: APT‐1011: ‐2.68 (1.91)/84 Placebo: –0.7 (1.31)/19
Dellon 2022b	Presentation, not publications	Not reported	Not reported
De Rooij 2022	**EREFS** Hirano 2013 Validated Inflammatory score Fibrostenotic score	Endoscopic features are scored according to the EREFS classification and sub‐classified as (i) inflammatory signs including white exudates, edema, and linear furrows (ii) fibrostenotic signs including rings and strictures The following scores were reported as median (IQR) EREFS ‐ post‐treatment Inflammatory score ‐ post‐treatment Fibrostenotic score ‐ post‐treatment	Median (IQR) reported, cannot use EREFS, median (IQR) (used for analysis) Four‐food elimination diet = 4 (1 to 4), SD = 3.20503 Four‐food elimination diet + amino acid formula = 3 (1.5 to 4), SD = 2.746079 Inflammatory score, median (IQR) Four‐food elimination diet: 2 (1 to 2) Four‐food elimination diet + amino acid formula: 2 (1 to 2) Fibrostenotic score, median (IQR) Four‐food elimination diet: 1 (1 to 2) Four‐food elimination diet + amino acid formula: 1 (1 to 2)
Dohil 2010	Endoscopy scoring tool Aceves 2009 Not validated	Continuous Pre‐ and post‐scores Mucosal pallor/reduced vasculature Linear furrows/mucosal thickening, white plaques, concentric rings/stricture Friability/“tissue‐paper” mucosa Histology scoring tools Epithelial histology score Peak eosinophil count Absent = 0 Present = 1	Endoscopy score at end of trial, mean (SD) (used for endoscopic continuous analysis): Budesonide + PPI: 1.5 (2.5)/15 Placebo + PPI: 5.4 (2.8)/9
Gupta 2015	Not reported	Not reported	Not reported
Heine 2019	Not reported	Not reported	Not reported
Hirano 2019	**EREFS** Hirano 2013 Validated	**EREFS:** Continuous, mean difference	EREFS at end of trial, mean (SD) (used for endoscopic continuous analysis): RPC4046 180 mg: 5.3 (4.2)/27 RPC4046 360 mg: 4.8 (3.4)/30 RPC4046 = 5.04 (3.71)/57 Placebo: 7.9 (5.1)/32
Hirano 2020	**EREFS** Hirano 2013 Validated Change in esophageal distensibility plateau as measured by functional lumen imaging	Continuous	Change in EREFS from baseline at end of trial, LS mean change from baseline (SD) (used for endoscopic continuous analysis), N/imputed n: Dupilumab: ‐1.9 (1.4)/23/0 Placebo: ‐0.3 (1.5)/24/2
Hirano 2020f	**EREFS** Hirano 2013 Validated	Dichotomous Change from baseline to week 8/end of trial improvement/no change/worsening	Supplementary Table 3, data are from pre‐specified analyses (used for endoscopic dichotomous analysis): APT‐1011 at 1.5 mg twice‐daily: 5/8 APT‐1011 at 3.0 mg daily: 5/8 APT‐1011: 10/16 Placebo: 0/8 Placebo: improvement 0; no change 7; worsening 1 APT‐1011 1.5 mg: improvement 5; no change 2; worsening 1 APT‐1011 3 mg: improvement 5; no change 3; worsening 0 Continuous outcomes, data are from post hoc analyses (cannot use) APT‐1011 at 1.5 mg twice‐daily: ‐2.92 (95% CI ‐4.68 to ‐0.88) APT‐1011 at 3.0 mg daily: ‐2.74 (95% CI ‐4.5 to ‐0.88) APT‐1011: ‐2.83 (1.72) n = 16 Placebo: 0 (1.72) n = 8
Hirano 2021	**EREFS** Hirano 2013 Validated	Continuous (mean (SD))	EREFS at end of trial, mean (SD) (used for endoscopic continuous analysis): Budesonide: 4.2 (3.3)/202 Placebo: 6.2 (3.7)/93
Kliewer 2019	**EREFS** Hirano 2013 Validated	Continuous (change in mean (SD))	From NCT02610816, change in EREFS from baseline at end of trial, mean (SD) (used for endoscopic continuous analysis): 1‐food elimination diet change from baseline: ‐0.7 (2.2)/22 4‐food elimination diet change from baseline: ‐1.3 (2.2)/12
Kliewer 2021	**EREFS** Hirano 2013 Validated	Continuous EREFS change from baseline mean (SD)	Change in EREFS from baseline at end of trial, mean (SD) (used for endoscopic continuous analysis): 1‐food elimination at 6 weeks: ‐1.32 (3.28)/67 6‐food elimination at 6 weeks: ‐1.74 (3.91)/62
Konikoff 2006	No scoring system used Konikoff 2006	Dichotomous (number of patients with esophageal furrowing, epithelial hyperplasia, and esophageal mastocytosis)	Dichotomous, endoscopic: lack of furrows in the esophagus at end of trial (used for endoscopic dichotomous analysis): Fluticasone: 11/21 (52.4%) Placebo: 5/15 (33.3%) After treatment, significantly fewer individuals in the fluticasone propionate (FP) group had endoscopic distal esophageal furrowing compared with the placebo group (50% vs 91%). Endoscopic distal esophageal furrowing was not present in any FP responders (0/10) after treatment, while all FP non‐responders (10/10) had persistent furrowing in the distal esophagus. Treatment withFP significantly reduced epithelial hyperplasia in both the proximal and distal esophagus, as assessed by histologic examination of H&E‐stained sections. Placebo had no effect. In the FP group, mast cell counts were significantly decreased by treatment (17.1 ± 3.5 pre‐treatment vs 7.3 ± 2.2 post‐treatment mast cells/hpf in the proximal esophagus and 17.9 ± 3.1 pre‐treatment vs 9.8 ± 2.2 post‐treatment mast cells/hpf in the distal esophagus) and post‐treatment mast cell counts were significantly lower in the FP group than in the placebo group. FP responders had significantly lower post‐treatment mast cell counts than FP non‐responders (1.8 ± 0.5 vs 13.3 ± 3.6 mast cells/hpf in the proximal esophagus and 2.9 ± 1.0 vs 17.5 ± 2.5 mast cells/hpf in the distal esophagus).
Lieberman 2018	Not reported	Not reported	Not reported
Lucendo 2019	**EREFS** Hirano 2013 Validated	Continuous mean (SD) at end of trial	EREFS at end of trial, mean (SD) (used for endoscopic continuous analysis). Calculated from supplementary Table 5: Budesonide: 1.3(1.04)/59 Placebo: 4.6(1.26)/28
Miehlke 2016	Endoscopic score Global assessment of endoscopic appearance was determined using a 100 mm visual analogue scale (VAS) Not validated	**Continuous** Mean change in total endoscopic intensity score Endoscopic abnormalities: absent (0), mild (1), moderate (2), or severe (3): white exudates, furrows, edema, fixed rings, crêpe paper sign, short‐segment stenosis, long‐distance stenosis. Total endoscopic intensity score ranged from 0 to 21. Mean change in VAS endoscopic score (No SD reported)	No SD reported, cannot use data AT 2 weeks Endoscopic intensity score: Budesonide effervescent tablet 2 x 1 mg: ‐4.1 Budesonide effervescent tablet 2 x 2 mg: ‐3.4 Budesonide viscous suspension 2 x 2mg: ‐3.6 Placebo: ‐0.7 VAS endoscopic score: Budesonide effervescent tablet 2 x 1 mg: ‐37.4 Budesonide effervescent tablet 2 x 2 mg: ‐31.7 Budesonide viscous suspension 2 x 2mg: ‐25.2 Placebo: ‐9.6
Moawad 2013	Endoscopic assessment	Dichotomous Improvement of endoscopic findings	No aggregate outcome reported, cannot use data Stenosis on index endoscopy Fluticasone: (4/5) 80% ESO: (4/5) 80% Concentric rings Fluticasone: (2/16) 13% ESO: (7/16) 44% Longitudinal furrows Fluticasone: (3/17) 18% ESO: (7/17) 41% White plaques Fluticasone: (2/4) 24% ESO: (5/5) 100%
Oliva 2018	**EREFS** Hirano 2013 Validated	Not reported	Not reported
Peterson 2010	No scoring system, but morphological assessment Not validated	Continuous No threshold of success defined	No aggregate outcome reported, cannot use data Note: it is unclear of these were the findings at baseline or at 8 weeks (end of the study) Rings, n (%) Fluticasone 14/15 Esomeprazole 15/15 Furrows, n(%) Fluticasone 1/15 Esomeprazole 1/15 Abscesses, n(%) Fluticasone 3/15 Esomeprazole 2/15
Rothenberg 2015	Not reported	Not reported	Not reported
Rothenberg 2022	Not reported	Not reported	Not reported
Schaefer 2008	Endoscopy score Schaefer 2008	Dichotomous, improvement of one or more histological grades	Calculated from Table 5, improvement of one or more histological grades at end of trial (used for endoscopic dichotomous analysis): Fluticasone: 34/40 Prednisone: 30/40
Spergel 2012	Not reported	Not reported	Not reported
Spergel 2020	**EREFS** Hirano 2013 Validated	Continuous No threshold of success was identified but a mean difference of change was calculated	EREFs at end of trial, mean (SD) (used for analysis) Viaskin milk = 1.93 (1.58)/15 Placebo = 1.60 (1.67)/5 Change in EREFS from baseline at end of trial, mean (SD) (used for endoscopic continuous analysis): Mean ± SD Viaskin milk = –0.07 (1.49)/15 Placebo = –0.80 (1.30)/5
Straumann 2010a	Endoscopic eosinophilic esophagitis abnormalities Straumann 2003	Dichotomous Endoscopic findings were graded by means of a simple overall score: absent, minor (fine nodules, fine whitish reticular structures, furrows), moderate (bright white scale‐ or plaque‐like structures, corrugated rings) or severe (mucosal lesions, fixed stenosis) Absence of features is defined as the primary outcome	Absence of esophageal abnormalities at end of trial (used for endoscopic dichotomous analysis): Absent: Mepolizumab: 0/5 Placebo: 0/6 Minor n = 0/5, n = 1/6 Moderate n = 3/5, n = 3/6 Severe n = 2/5, n = 2/6
Straumann 2010b	Macroscopic assessment during endoscopy	Dichotomous Threshold of success was not established. However, more disappearing endoscopic features counts as success. Roughly classified as absent, minimal, moderate, or severe… []. Additionally, the presence of 6 major signs of EoE (white exudates, red furrows, corrugated rings, solitary rings, crêpe paper sign, and severe stenosis impossible to pass with the standard endoscope) as well as signs of fungal infection were recorded.	No aggregate score reported, cannot use data Among the 10 patients with complete histologic remission: 10/10 had white exudates disappear 8/9 had red furrows disappear 8/9 had corrugated rings persist
Straumann 2011	Not reported	Not reported	Not reported
Straumann 2013	The global appearance of endoscopic abnormalities was assessed using a 10 cm visual analogue scale	Continuous; compared means; no pre‐specified treatment response threshold	Change in global assessment of endoscopic appearance from baseline at end of trial, mean (SD) (used for endoscopic continuous analysis): OC004549: 6.06 (1.79)/14 Placebo: ‐5.57 (2.20)/12
Straumann 2020	**EREFS** Hirano 2013 Validated There were many endoscopic outcomes reported; however, all were exploratory	Continuous	EREFS at end of trial, mean (SD) (used for endoscopic continuous analysis): Budesonide 0.5 mg twice‐daily: 1 (1.2)/65 Budesonide 1.0 mg twice‐daily: 1 (1.1)/65 Budesonide: 1 (1.14)/130 Placebo: 4 (1.8)/65
Tytor 2021	Not reported	Not reported	Not reported

AAF: amino acid‐based formula; BET: budesonide effervescent tablet; BOV: budesonide, oral viscous; CG: control group; CI: confidence interval; EoE: eosinophilic esophagitis; EoT: end of treatment; EREFS: EoE Endoscopic Reference Score; FFED: four food elimination diet; IG: intervention group; ESO: esomeprazole; IQR: interquartile range; LS: least squares; NEB: nebulized/swallowed budesonide solution; OVB: viscous/swallowed budesonide solution; PPI: proton pump inhibitor; SD: standard deviation; VAS: visual analogue scale

**Summary of findings 9:** One‐food elimination diet compared to four‐food elimination diet

**One‐food elimination diet compared to four‐food elimination diet**						
**Patient or population:** active EoE pediatric patients **Setting:** medical centers **Intervention:** one‐food elimination diet **Comparison:** four‐food elimination diet						
**Outcomes**	**№ of participants** **(studies)**	**Certainty of the evidence (GRADE)**	**Relative effect (95% CI)**	Anticipated absolute effects^*^ (95% CI)		**Comments**
				Risk with f**our‐food elimination diet**	Risk difference with one‐f**ood elimination diet**	
Clinical improvement (dichotomous)	—	—	—	—	—	—
Clinical improvement at 12 weeks (continuous)	50 (1 study)	⊕⊝⊝⊝ **Very low^a^**	—	—	MD 7.5 lower (16.28 lower to 1.28 higher)	Measured on the EoE Symptom Activity Index
Histological improvement at 12 weeks (dichotomous)	63 (1 study)	⊕⊝⊝⊝ **Very low^a^**	RR 2.26 (1.15 to 4.43)	Study population		—
				280 per 1000	353 more per 1000 (42 more to 960 more)	
Histological improvement at study endpoint (continuous)	—	—	—	—	—	No data
Endoscopic improvement at study endpoint (dichotomous)	—	—	—	—	—	No data
Endoscopic improvement at 12 weeks (continuous)	34 (1 study)	⊕⊝⊝⊝ **Very low^a^**	—	—	MD 0.6 lower (2.15 lower to 0.95 higher)	Measured on the endoscopic reference score
Withdrawals due to adverse events at 12 weeks	63 (1 study)	⊕⊝⊝⊝ **Very low^a^**	RR 0.33 (0.11 to 0.98)	Study population		—
				320 per 1000	214 fewer per 1000 (285 fewer to 6 fewer)	
*The risk in the intervention group (and its 95% confidence interval) is based on the assumed risk in the comparison group and the **relative effect** of the intervention (and its 95% CI). ** The risk for the control intervention has been calculated by dividing the number of cases to the number of randomized participants, using the numbers of our analyses. The risk for the comparison intervention has been calculated by multiplying the control risk with the RR and CI limits. The risk difference has been calculated by subtracting the control risk from the comparison intervention risk. **CI:** confidence interval; **EoE:** eosinophilic esophagitis; **MD:** mean difference; **NNTB:** number needed to treat for an additional beneficial outcome; **RR:** risk ratio; **SMD:** standardized mean difference						
**GRADE Working Group grades of evidence** **High certainty:** we are very confident that the true effect lies close to that of the estimate of the effect. **Moderate certainty:** we are moderately confident in the effect estimate: the true effect is likely to be close to the estimate of the effect, but there is a possibility that it is substantially different. **Low certainty:** our confidence in the effect estimate is limited: the true effect may be substantially different from the estimate of the effect. **Very low certainty:** we have very little confidence in the effect estimate: the true effect is likely to be substantially different from the estimate of effect.						

^a^Downgraded twice due to serious imprecision and once due to risk of bias for unclear blinding of participants and personnel, and attrition.

**Summary of findings 10:** One‐food elimination diet compared to six‐food elimination diet

**One‐food elimination diet compared to six‐food elimination diet**						
**Patient or population:** active EoE pediatric patients **Setting:** medical centers **Intervention:** one‐food elimination diet **Comparison:** six‐food elimination diet						
**Outcomes**	**№ of participants** **(studies)**	**Certainty of the evidence (GRADE)**	**Relative effect (95% CI)**	Anticipated absolute effects^*^ (95% CI)		**Comments**
				Risk with s**ix‐food elimination diet**	Risk difference with one‐f**ood elimination diet**	
Clinical improvement (dichotomous)	—	—	—	—	—	No data
Clinical improvement at 6 weeks (continuous)	129 (1 study)	⊕⊝⊝⊝ **Very low^a^**	—	—	MD 5.2 lower (11.06 lower to 0.66 higher)	Measured on the EoE Symptom Activity Index
Histological improvement at 6 weeks (dichotomous)	129 (1 study)	⊕⊝⊝⊝ **Very low^a^**	RR 0.85 (0.54 to 1.33)	Study population		—
				403 per 1000	60 fewer per 1000 (185 fewer to 133 more)	—
Histological improvement at 6 weeks (continuous)	129 (1 study)	⊕⊝⊝⊝ **Very low^a^**	—	—	MD 6.8 higher (10.4 lower to 24 higher)	Measured as changes in the EoE Histologic Scoring System
Endoscopic improvement at study endpoint (dichotomous)	—	—	—	—	—	No data
Endoscopic improvement at 6 weeks (continuous)	129 (1 study)	⊕⊝⊝⊝ **Very low^a^**	—	—	MD 0.42 lower (1.67 lower to 0.83 higher)	Measured on the endoscopic reference score
Withdrawals due to adverse events	129 (1 study)	⊕⊝⊝⊝ **Very low^a^**	RR 0.62 (0.11 to 3.57)	Study population		—
				403 per 1000	60 fewer per 1000 (185 fewer to 133 more)	—
*The risk in the intervention group (and its 95% confidence interval) is based on the assumed risk in the comparison group and the **relative effect** of the intervention (and its 95% CI). ** The risk for the control intervention has been calculated by dividing the number of cases to the number of randomized participants, using the numbers of our analyses. The risk for the comparison intervention has been calculated by multiplying the control risk with the RR and CI limits. The risk difference has been calculated by subtracting the control risk from the comparison intervention risk. **CI:** confidence interval; **EoE:** eosinophilic esophagitis; **MD:** mean difference; **NNTB:** number needed to treat for an additional beneficial outcome; **RR:** risk ratio; **SMD:** standardized mean difference						
**GRADE Working Group grades of evidence** **High certainty:** we are very confident that the true effect lies close to that of the estimate of the effect. **Moderate certainty:** we are moderately confident in the effect estimate: the true effect is likely to be close to the estimate of the effect, but there is a possibility that it is substantially different. **Low certainty:** our confidence in the effect estimate is limited: the true effect may be substantially different from the estimate of the effect. **Very low certainty:** we have very little confidence in the effect estimate: the true effect is likely to be substantially different from the estimate of effect.						

^a^Downgraded twice due to serious imprecision and risk of bias for blinding of participants and personnel, and other bias.

**Summary of findings 11:** Four‐food elimination diet with omeprazole compared to omeprazole

**Four‐food elimination diet with omeprazole compared to omeprazole**						
**Patient or population:** active EoE patients **Setting:** medical centers **Intervention:** four‐food elimination diet with omeprazole **Comparison:** omeprazole						
**Outcomes**	**№ of participants** **(studies)**	**Certainty of the evidence (GRADE)**	**Relative effect (95% CI)**	Anticipated absolute effects^*^ (95% CI)		**Comments**
				Risk with o**meprazole**	**Risk difference with four‐food elimination diet with omeprazole**	
Clinical improvement (dichotomous)	—	—	—	—	—	No data
Clinical improvement (continuous)	—	—	—	—		No data
Histological improvement at 8 to 12 weeks (dichotomous)	64 (1 study)	⊕⊝⊝⊝ **Very low^a^**	RR 1.57 (0.99 to 2.48)	Study population		—
				438 per 1000	250 more per 1000 (4 fewer to 648 more)	
Histological improvement at 8 to 12 weeks (continuous)	58 (1 study)	⊕⊝⊝⊝ **Very low^a^**	—	—	MD 9.50 higher (11.18 lower to 30.18 higher)	—
Endoscopic improvement at study endpoint (dichotomous)	—	—	—	—	—	No data
Endoscopic improvement at study endpoint (continuous)	—	—	—	—	—	No data
Withdrawals due to adverse events at 8 to 12 weeks	64 (1 study)	⊕⊝⊝⊝ **Very low^a^**	RR 5.00 (0.62 to 40.44)	Study population		—
				31 per 1000	124 more per 1000 (12 fewer to 1000 more)	
*The risk in the intervention group (and its 95% confidence interval) is based on the assumed risk in the comparison group and the **relative effect** of the intervention (and its 95% CI). ** The risk for the control intervention has been calculated by dividing the number of cases to the number of randomized participants, using the numbers of our analyses. The risk for the comparison intervention has been calculated by multiplying the control risk with the RR and CI limits. The risk difference has been calculated by subtracting the control risk from the comparison intervention risk. **CI:** confidence interval; **EoE:** eosinophilic esophagitis; **MD:** mean difference; **NNTB:** number needed to treat for an additional beneficial outcome; **RR:** risk ratio; **SMD:** standardized mean difference						
**GRADE Working Group grades of evidence** **High certainty:** we are very confident that the true effect lies close to that of the estimate of the effect. **Moderate certainty:** we are moderately confident in the effect estimate: the true effect is likely to be close to the estimate of the effect, but there is a possibility that it is substantially different. **Low certainty:** our confidence in the effect estimate is limited: the true effect may be substantially different from the estimate of the effect. **Very low certainty:** we have very little confidence in the effect estimate: the true effect is likely to be substantially different from the estimate of effect.						

^a^Downgraded twice due to serious imprecision and once due to risk of bias for blinding of participants, personnel, and outcome assessment, unclear attrition, and selective reporting.

**Summary of findings 12:** Four‐food elimination and amino acid formula compared to four‐food elimination diet

**Four‐food elimination and amino acid formula compared to four‐food elimination diet**						
**Patient or population:** active EoE patients **Setting:** medical center **Intervention:** four‐food elimination and amino acid formula **Comparison**: four‐food elimination diet						
**Outcomes**	**№ of participants** **(studies)**	**Certainty of the evidence (GRADE)**	**Relative effect (95% CI)**	Anticipated absolute effects^*^ (95% CI)		**Comments**
				Risk with **four‐food elimination diet**	**Risk difference with four‐food elimination and amino acid formula**	
Clinical improvement (dichotomous)	—	—	—	—	—	No data
Clinical improvement (continuous)	41 (1 study)	⊕⊝⊝⊝ **Very low^a^**	—	—	MD 0.50 lower (2.41 lower to 1.41 higher)	—
Histological improvement at 6 weeks (dichotomous)	41 (1 study)	⊕⊝⊝⊝ **Very low^a^**	RR 1.90 (0.79 to 4.60)	Study population		—
				250 per 1000	225 more per 1000 (53 fewer to 900 more)	—
Histological improvement at 6 weeks (continuous)	41 (1 study)	⊕⊝⊝⊝ **Very low^a^**	—	—	MD 13.8 higher (9.5 lower to 37.1 higher)	Measured as peak eosinophil count
Endoscopic improvement (dichotomous)	—	—	—	—	—	No data
Endoscopic improvement at study endpoint (continuous)	41 (1 study)	⊕⊝⊝⊝ **Very low^a^**	—	—	MD 1.00 lower (2.83 lower to 0.83 higher)	Measured on the endoscopic reference score
Withdrawals due to adverse events at 6 weeks	41 participants (1 study)	⊕⊝⊝⊝ **Very low^a^**	RR 0.95 (0.06 to 14.22)	Study population		—
				50 per 1000	3 fewer per 1000 (47 fewer to 661 more)	—
*The risk in the intervention group (and its 95% confidence interval) is based on the assumed risk in the comparison group and the **relative effect** of the intervention (and its 95% CI). ** The risk for the control intervention has been calculated by dividing the number of cases to the number of randomized participants, using the numbers of our analyses. The risk for the comparison intervention has been calculated by multiplying the control risk with the RR and CI limits. The risk difference has been calculated by subtracting the control risk from the comparison intervention risk. **CI:** confidence interval; **EoE:** eosinophilic esophagitis; **MD:** mean difference; **NNTB:** number needed to treat for an additional beneficial outcome; **RR:** risk ratio; **SMD:** standardized mean difference						
**GRADE Working Group grades of evidence** **High certainty:** we are very confident that the true effect lies close to that of the estimate of the effect. **Moderate certainty:** we are moderately confident in the effect estimate: the true effect is likely to be close to the estimate of the effect, but there is a possibility that it is substantially different. **Low certainty:** our confidence in the effect estimate is limited: the true effect may be substantially different from the estimate of the effect. **Very low certainty:** we have very little confidence in the effect estimate: the true effect is likely to be substantially different from the estimate of effect.						

^a^Downgraded twice due to serious imprecision and once due to risk of bias for unclear randomization, blinding of participants, personnel, and outcome assessment.

**Summary of findings 13:** Nebulized budesonide compared to viscous budesonide

**Nebulized budesonide compared to viscous budesonide**						
**Patient or population:** active EoE patients **Setting:** medical center **Intervention:** nebulized budesonide **Comparison:** viscous budesonide						
**Outcomes**	**№ of participants** **(studies)**	**Certainty of the evidence (GRADE)**	**Relative effect (95% CI)**	Anticipated absolute effects^*^ (95% CI)		**Comments**
				**Risk with viscous budesonide**	**Risk difference with nebulized budesonide**	
Clinical improvement (dichotomous)	—	—	—	—	—	No data
Clinical improvement at 8 weeks (continuous)	22 (1 study)	⊕⊝⊝⊝ **Very low^a^**			MD 6.00 lower (18.3 lower to 6.3 higher)	—
Histological improvement at 8 weeks (dichotomous)	—	—	—	—	—	No data
Histological improvement at 8 weeks (continuous)	22 (1 study)	⊕⊝⊝⊝ **Very low^a^**	—	—	MD 78.00 higher (20.81 higher to 135.19 higher)	—
Endoscopic improvement (dichotomous)	25 (1 study)	⊕⊝⊝⊝ **Very low^a^**	—	—	—	The study reported specific endoscopic characteristics which can be found in Table 9
Endoscopic improvement (continuous)	—	—	—	—	—	No data
Withdrawals due to adverse events at 8 weeks	25 (1 study)	⊕⊝⊝⊝ **Very low^a^**	Not estimable	Study population		—
				0 per 1000	0 fewer per 1000 (0 fewer to 0 fewer)	
*The risk in the intervention group (and its 95% confidence interval) is based on the assumed risk in the comparison group and the **relative effect** of the intervention (and its 95% CI). ** The risk for the control intervention has been calculated by dividing the number of cases to the number of randomized participants, using the numbers of our analyses. The risk for the comparison intervention has been calculated by multiplying the control risk with the RR and CI limits. The risk difference has been calculated by subtracting the control risk from the comparison intervention risk. **CI:** confidence interval; **EoE:** eosinophilic esophagitis; **MD:** mean difference; **NNTB:** number needed to treat for an additional beneficial outcome; **RR:** risk ratio; **SMD:** standardized mean difference						
**GRADE Working Group grades of evidence** **High certainty:** we are very confident that the true effect lies close to that of the estimate of the effect. **Moderate certainty:** we are moderately confident in the effect estimate: the true effect is likely to be close to the estimate of the effect, but there is a possibility that it is substantially different. **Low certainty:** our confidence in the effect estimate is limited: the true effect may be substantially different from the estimate of the effect. **Very low certainty:** we have very little confidence in the effect estimate: the true effect is likely to be substantially different from the estimate of effect.						

^a^Downgraded twice due to imprecision and once due to risk of bias for blinding of participants and personnel.

**Summary of findings 14:** Viaskin milk patch compared to placebo

**Viaskin milk patch compared to placebo**						
**Patient or population:** active EoE pediatric patients **Setting:** medical center **Intervention:** Viaskin milk patch **Comparison:** placebo						
**Outcomes**	**№ of participants** **(studies)**	**Certainty of the evidence (GRADE)**	**Relative effect (95% CI)**	Anticipated absolute effects^*^ (95% CI)		**Comments**
				**Risk with placebo**	Risk difference with **Viaskin milk patch**	
Clinical improvement (dichotomous)	—	—	—	—	—	No data
Clinical improvement at 44 weeks (continuous)	9 (1 study)	⊕⊕⊝⊝ **Low^a^**	—	—	MD 1.29 higher (0.83 lower to 3.41 higher)	Measured on the eosinophilic esophagitis symptom score
Histological improvement (dichotomous)	—	—	—	—	—	No data
Histological improvement at 44 weeks (continuous)	9 (1 study)	⊕⊕⊝⊝ **Low^a^**	—	—	MD 69.43 higher (21.75 lower to 160.61 higher)	Measured as change in maximum esophageal eosinophil count from baseline to end of study
Endoscopic improvement (dichotomous)	—	—	—	—	—	No data
Endoscopic improvement at 44 weeks (continuous)	20 (1 study)	⊕⊕⊝⊝ **Low^a^**	—	—	MD 0.33 lower (2 lower to 1.34 higher)	Measured on the endoscopic reference score
Withdrawals due to adverse events at 44 weeks	20 (1 study)	⊕⊕⊝⊝ **Low^a^**	RR 1.12 (0.05 to 23.99)	Study population		—
				0 per 1000	66 fewer per 1000 (372 fewer to 660 more)	
*The risk in the intervention group (and its 95% confidence interval) is based on the assumed risk in the comparison group and the **relative effect** of the intervention (and its 95% CI). ** The risk for the control intervention has been calculated by dividing the number of cases to the number of randomized participants, using the numbers of our analyses. The risk for the comparison intervention has been calculated by multiplying the control risk with the RR and CI limits. The risk difference has been calculated by subtracting the control risk from the comparison intervention risk. **CI:** confidence interval; **EoE:** eosinophilic esophagitis; **MD:** mean difference; **NNTB:** number needed to treat for an additional beneficial outcome; **RR:** risk ratio; **SMD:** standardized mean difference						
**GRADE Working Group grades of evidence** **High certainty:** we are very confident that the true effect lies close to that of the estimate of the effect. **Moderate certainty:** we are moderately confident in the effect estimate: the true effect is likely to be close to the estimate of the effect, but there is a possibility that it is substantially different. **Low certainty:** our confidence in the effect estimate is limited: the true effect may be substantially different from the estimate of the effect. **Very low certainty:** we have very little confidence in the effect estimate: the true effect is likely to be substantially different from the estimate of effect.						

^a^Downgraded twice due to serious imprecision.

**Summary of findings 15:** Leukotriene receptor antagonist compared to placebo for maintenance of remission

**Leukotrienereceptor antagonist compared to placebo for maintenance of remission**						
**Patient or population:** inactive EoE patients **Setting:** medical center **Intervention:** leukotriene receptor antagonist **Comparison:** placebo						
**Outcomes**	**№ of participants** **(studies)**	**Certainty of the evidence (GRADE)**	**Relative effect (95% CI)**	Anticipated absolute effects^*^ (95% CI)		**Comments**
				**Risk with placebo**	Risk difference with l**eukotriene receptor antagonist**	
Clinical improvement at 26 weeks (dichotomous)	41 (1 study)	⊕⊝⊝⊝ **Very low^a^**	RR 1.68 (0.66 to 4.28)	Study population		—
				238 per 1000	162 more per 1000 (81 fewer to 781 more)	
Clinical improvement (continuous)	—	—	—	—	—	No data
Histological improvement (dichotomous)	—	—	—	—	—	No data
Histological improvement (continuous)	—	—	—	—	—	No data
Endoscopic improvement (dichotomous)	—	—	—	—	—	No data
Endoscopic improvement (continuous)	—	—	—	—	—	No data
Withdrawals due to adverse events at 26 weeks	41 (1 study)	⊕⊝⊝⊝ **Very low^a^**	RR 2.10 (0.21 to 21.39)	Study population		—
				48 per 1000	53 more per 1000 (38 fewer to 979 more)	—
*The risk in the intervention group (and its 95% confidence interval) is based on the assumed risk in the comparison group and the **relative effect** of the intervention (and its 95% CI). ** The risk for the control intervention has been calculated by dividing the number of cases to the number of randomized participants, using the numbers of our analyses. The risk for the comparison intervention has been calculated by multiplying the control risk with the RR and CI limits. The risk difference has been calculated by subtracting the control risk from the comparison intervention risk. **CI:** confidence interval; **EoE:** eosinophilic esophagitis; **MD:** mean difference; **NNTB:** number needed to treat for an additional beneficial outcome; **RR:** risk ratio; **SMD:** standardized mean difference						
**GRADE Working Group grades of evidence** **High certainty:** we are very confident that the true effect lies close to that of the estimate of the effect. **Moderate certainty:** we are moderately confident in the effect estimate: the true effect is likely to be close to the estimate of the effect, but there is a possibility that it is substantially different. **Low certainty:** our confidence in the effect estimate is limited: the true effect may be substantially different from the estimate of the effect. **Very low certainty:** we have very little confidence in the effect estimate: the true effect is likely to be substantially different from the estimate of effect.						

^a^Downgraded twice due to serious imprecision and once due to risk of bias for unclear selective reporting.

**Summary of findings 16:** Mepolizumab 10 mg/kg compared to mepolizumab 0.55 mg/kg

**Mepolizumab 10 mg/kg compared to mepolizumab 0.55 mg/kg**						
**Patient or population:** active EoE pediatric patients **Setting:** medical centers **Intervention:** mepolizumab 10 mg/kg **Comparison:** mepolizumab 0.55 mg/kg						
**Outcomes**	**№ of participants** **(studies)**	**Certainty of the evidence (GRADE)**	**Relative effect (95% CI)**	Anticipated absolute effects^*^ (95% CI)		**Comments**
				**Risk with viscous mepolizumab 0.55 mg/kg**	**Risk difference with mepolizumab 10 mg/kg**	
Clinical improvement (dichotomous)	—	—	—	—	—	No data
Clinical improvement at (continuous)	—	—	—	—	—	No data
Histological improvement at 12 weeks (dichotomous)	39 (1 study)	⊕⊝⊝⊝ **Very low^a^**	RR 1.19 (0.37 to 3.77)	Study population		—
				211 per 1000	40 more per 1000 (133 fewer to 584 more)	
Histological improvement (continuous)	—	—	—	—	—	No data
Endoscopic improvement (dichotomous)	—	—	—	—	—	No data
Endoscopic improvement (continuous)	—	—	—	—	—	No data
Withdrawals due to adverse events at 8 weeks	39 (1 study)	⊕⊝⊝⊝ **Very low^a^**	RR 0.63 (0.12 to 3.38)	Study population		—
				158 per 1000	48 more per 1000 (139 fewer to 376 more)	
*The risk in the intervention group (and its 95% confidence interval) is based on the assumed risk in the comparison group and the **relative effect** of the intervention (and its 95% CI). ** The risk for the control intervention has been calculated by dividing the number of cases to the number of randomized participants, using the numbers of our analyses. The risk for the comparison intervention has been calculated by multiplying the control risk with the RR and CI limits. The risk difference has been calculated by subtracting the control risk from the comparison intervention risk. **CI:** confidence interval; **EoE:** eosinophilic esophagitis; **MD:** mean difference; **NNTB:** number needed to treat for an additional beneficial outcome; **RR:** risk ratio; **SMD:** standardized mean difference						
**GRADE Working Group grades of evidence** **High certainty:** we are very confident that the true effect lies close to that of the estimate of the effect. **Moderate certainty:** we are moderately confident in the effect estimate: the true effect is likely to be close to the estimate of the effect, but there is a possibility that it is substantially different. **Low certainty:** our confidence in the effect estimate is limited: the true effect may be substantially different from the estimate of the effect. **Very low certainty:** we have very little confidence in the effect estimate: the true effect is likely to be substantially different from the estimate of effect.						

^a^Downgraded twice due to serious imprecision and once due to risk of bias.

**Summary of findings 17:** Mepolizumab 2.5 mg/kg compared to mepolizumab 0.55 mg/kg

**Mepolizumab 2.5 mg/kg compared to mepolizumab 0.55 mg/kg**						
**Patient or population:** active EoE pediatric patients **Setting:** medical centers **Intervention:** mepolizumab 2.5 mg/kg **Comparison:** mepolizumab 0.55 mg/kg						
**Outcomes**	**№ of participants** **(studies)**	**Certainty of the evidence (GRADE)**	**Relative effect (95% CI)**	Anticipated absolute effects^*^ (95% CI)		**Comments**
				**Risk with viscous mepolizumab 0.55 mg/kg**	**Risk difference with mepolizumab 2.5 mg/kg**	
Clinical improvement (dichotomous)	—	—	—	—	—	No data
Clinical improvement at (continuous)	—	—	—	—	—	No data
Histological improvement at 12 weeks (dichotomous)	39 (1 study)	⊕⊝⊝⊝ **Very low^a^**	RR 2.14 (0.79 to 5.79)	Study population		—
				211 per 1000	241 more per 1000 (44 fewer to 1000 more)	
Histological improvement (continuous)	—	—	—	—	—	No data
Endoscopic improvement (dichotomous)	—	—	—	—	—	No data
Endoscopic improvement (continuous)	—	—	—	—	—	No data
Withdrawals due to adverse events at 8 weeks	39 (1 study)	⊕⊝⊝⊝ **Very low^a^**	RR 0.32 (0.04 to 2.79)	Study population		—
				158 per 1000	107 fewer per 1000 (152 fewer to 283 more)	—
*The risk in the intervention group (and its 95% confidence interval) is based on the assumed risk in the comparison group and the **relative effect** of the intervention (and its 95% CI). ** The risk for the control intervention has been calculated by dividing the number of cases to the number of randomized participants, using the numbers of our analyses. The risk for the comparison intervention has been calculated by multiplying the control risk with the RR and CI limits. The risk difference has been calculated by subtracting the control risk from the comparison intervention risk. **CI:** confidence interval; **EoE:** eosinophilic esophagitis; **MD:** mean difference; **NNTB:** number needed to treat for an additional beneficial outcome; **RR:** risk ratio; **SMD:** standardized mean difference						
**GRADE Working Group grades of evidence** **High certainty:** we are very confident that the true effect lies close to that of the estimate of the effect. **Moderate certainty:** we are moderately confident in the effect estimate: the true effect is likely to be close to the estimate of the effect, but there is a possibility that it is substantially different. **Low certainty:** our confidence in the effect estimate is limited: the true effect may be substantially different from the estimate of the effect. **Very low certainty:** we have very little confidence in the effect estimate: the true effect is likely to be substantially different from the estimate of effect.						

^a^Downgraded twice due to serious imprecision and once due to risk of bias for unclear randomization, unclear blinding of outcome assessment, and selective reporting.

**Summary of findings 18:** Mepolizumab 10 mg/kg compared to mepolizumab 2.5 mg/kg

**Mepolizumab 10 mg/kg compared to mepolizumab 2.5 mg/kg**						
**Patient or population:** active EoE pediatric patients **Setting:** medical centers **Intervention:** mepolizumab 10 mg/kg **Comparison:** mepolizumab 2.5 mg/kg						
**Outcomes**	**№ of participants** **(studies)**	**Certainty of the evidence (GRADE)**	**Relative effect (95% CI)**	Anticipated absolute effects^*^ (95% CI)		**Comments**
				**Risk with viscous mepolizumab 2.5 mg/kg**	**Risk difference with mepolizumab 10 mg/kg**	
Clinical improvement (dichotomous)	—	—	—	—	—	No data
Clinical improvement at (continuous)	—	—	—	—	—	No data
Histological improvement at 12 weeks (dichotomous)	40 (1 study)	⊕⊝⊝⊝ **Very low^a^**	RR 0.56 (0.23 to 1.37)	Study population		—
				450 per 1000	198 fewer per 1000 (347 fewer to 167 more)	
Histological improvement (continuous)	—	—	—	—	—	No data
Endoscopic improvement (dichotomous)	—	—	—	—	—	No data
Endoscopic improvement (continuous)	—	—	—	—	—	No data
Withdrawals due to adverse events at 8 weeks	40 (1 study)	⊕⊝⊝⊝ **Very low^a^**	RR 2.00 (0.20 to 20.33)	Study population		—
				50 per 1000	50 more per 1000 (40 fewer to 967 more)	—
*The risk in the intervention group (and its 95% confidence interval) is based on the assumed risk in the comparison group and the **relative effect** of the intervention (and its 95% CI). ** The risk for the control intervention has been calculated by dividing the number of cases to the number of randomized participants, using the numbers of our analyses. The risk for the comparison intervention has been calculated by multiplying the control risk with the RR and CI limits. The risk difference has been calculated by subtracting the control risk from the comparison intervention risk. **CI:** confidence interval; **EoE:** eosinophilic esophagitis; **MD:** mean difference; **NNTB:** number needed to treat for an additional beneficial outcome; **RR:** risk ratio; **SMD:** standardized mean difference						
**GRADE Working Group grades of evidence** **High certainty:** we are very confident that the true effect lies close to that of the estimate of the effect. **Moderate certainty:** we are moderately confident in the effect estimate: the true effect is likely to be close to the estimate of the effect, but there is a possibility that it is substantially different. **Low certainty:** our confidence in the effect estimate is limited: the true effect may be substantially different from the estimate of the effect. **Very low certainty:** we have very little confidence in the effect estimate: the true effect is likely to be substantially different from the estimate of effect.						

^a^Downgraded twice due to serious imprecision and once due to risk of bias for unclear randomization, unclear blinding of outcome assessment, and selective reporting.

**Summary of findings 19:** Six‐food elimination diet compared to swallowed fluticasone compared to swallowed budesonide compared to oral viscous budesonide

**Six‐food elimination diet compared to swallowed fluticasone compared to swallowed budesonide compared to oral viscous budesonide**					
**Patient or population:** active EoE pediatric patients **Setting:** medical center **Comparison:** six‐food elimination diet versus swallowed fluticasone versus swallowed budesonide versus oral viscous budesonide					
**Outcomes**	**№ of participants** **(studies)**	**Certainty of the evidence (GRADE)**	**Relative effect (95% CI)**	**Reported results**	**Comments**
Clinical improvement (dichotomous)	—	—	—	—	No data
Clinical improvement (continuous)	—	—	—	—	No data
Histological improvement at 8 weeks (dichotomous)	64 (1 study)	⊕⊝⊝⊝ **Very low^a^**		69% of participants in the six‐food elimination diet achieved histological improvement, 67% in the swallowed fluticasone group, 75% in the swallowed budesonide group, and 85% in the oral viscous budesonide group.	—
Histological improvement (continuous)	—	—	—	—	No data
Endoscopic improvement (dichotomous)	—	—	—	—	No data
Endoscopic improvement (continuous)	—	—	—	—	No data
Withdrawals due to adverse events at 26 weeks	—	—	—	—	No data
*The risk in the intervention group (and its 95% confidence interval) is based on the assumed risk in the comparison group and the **relative effect** of the intervention (and its 95% CI). ** The risk for the control intervention has been calculated by dividing the number of cases to the number of randomized participants, using the numbers of our analyses. The risk for the comparison intervention has been calculated by multiplying the control risk with the RR and CI limits. The risk difference has been calculated by subtracting the control risk from the comparison intervention risk. **CI:** confidence interval; **EoE:** eosinophilic esophagitis; **MD:** mean difference; **NNTB:** number needed to treat for an additional beneficial outcome; **RR:** risk ratio; **SMD:** standardized mean difference					
**GRADE Working Group grades of evidence** **High certainty:** we are very confident that the true effect lies close to that of the estimate of the effect. **Moderate certainty:** we are moderately confident in the effect estimate: the true effect is likely to be close to the estimate of the effect, but there is a possibility that it is substantially different. **Low certainty:** our confidence in the effect estimate is limited: the true effect may be substantially different from the estimate of the effect. **Very low certainty:** we have very little confidence in the effect estimate: the true effect is likely to be substantially different from the estimate of effect.					

^a^Downgraded twice due to risk of bias and once due to imprecision for unclear randomization, unclear blinding of outcome assessment, and selective reporting.

## Background

### Description of the condition

Eosinophilic esophagitis (EoE) is a chronic type 2 antigen‐mediated inflammatory disorder of the esophagus, causing upper gastrointestinal symptoms and characterized by increased esophageal infiltration with intraepithelial eosinophils (Liacouras 2011; Rothenberg 2015). It was originally described during the 1970s in adults with symptoms of esophagitis, who often had allergies, and who had high esophageal eosinophil counts. The diagnostic criteria for eosinophilic esophagitis are clinical symptoms of esophageal disease, a histological abnormality of 15 or more intraepithelial eosinophils per high‐power field (hpf) on endoscopy, the exclusion of gastroesophageal reflux disease (GERD), and consistent endoscopic findings (Furuta 2007). Eosinophilic esophagitis affects young infants to adults. In young children, the symptoms may be associated with feeding difficulties, vomiting, weight loss, and abdominal pain, however dysphagia and food impaction occur more often among teenagers and adults (Croese 2003; Liacouras 1998; Liacouras 2011; Orenstein 2000). In eosinophilic esophagitis, the mucosa may look normal macroscopically, however thickening, ringing, furrowing, and erosion have been reported (Dellon 2018; Furuta 2015; Hassall 1996; Khan 2003; Orenstein 2000).

Currently, there is no cure for eosinophilic esophagitis, making long‐term treatment critical. Treatment goals for eosinophilic esophagitis include improvement in clinical symptoms, resolution of esophageal eosinophilia and other histologic abnormalities, endoscopic improvement, improved quality of life, improved esophageal function, minimized adverse effects of treatment, and prevention of disease progression and subsequent complications (Muir 2021). Standard treatment modalities for eosinophilic esophagitis include dietary, pharmacologic, and endoscopic interventions. Dietary therapy involves empiric food elimination (most commonly milk protein) through dietary elimination and/or formula. Although effective, this approach can result in several endoscopies initially and be challenging to sustain long‐term (Kelly 1995; Markowitz 2003). Pharmacologic therapy includes proton pump inhibitors, topical glucocorticoids, and rapidly emerging biologics, including the first (US) Food & Drug Administration (FDA) approved medication for eosinophilic esophagitis (Greuter 2017; Muir 2021; Faubion 1998; Liacouras 1998; Teitelbaum 2002). Endoscopic intervention such as esophageal dilation is effective in relieving symptoms such as dysphagia, however it does not alter the underlying inflammation. This therapy is often used in conjunction with diet and/or medication. Eosinophilic esophagitis is a chronic disease that requires ongoing therapy and long‐term monitoring. If left untreated, eosinophilic esophagitis can result in complications such as fibrostenosis and strictures of the esophagus (Muir 2021; Schoepfer 2013).

### Description of the intervention

Medical management of eosinophilic esophagitis includes pharmacological therapy and dietary elimination.

Steroids are anti‐inflammatory drugs that have been used for the induction and maintenance of remission of eosinophilic esophagitis (Rank 2020). Budesonide and fluticasone have been the most frequently used, and to a lesser extent prednisone, beclomethasone, mometasone, and ciclesonide. Although initially administered systemically, swallowed topical administration has been the most frequent way of delivery, either adapted from asthma formulations (swallowed metered dose or nebulized solutions) or with compound viscous formulations. More recently esophageal‐specific topical steroid formulations, either oral suspension or oro‐dispersible tablets, have been developed.

Diverse biological therapies have been studied for the treatment of eosinophilic esophagitis including anti‐IL5 (mepolizumab, reslizumab), anti‐IgE (omalizumab), anti‐IL4r (dupilumab), anti‐IL13 (RPC4046, alias cendakimab; QAX576, alias dectrekumab), and anti‐sialic acid binding Ig‐like lectin 8 (Siglec‐8) (lirentelimab) (Nhu 2022).

There is very limited evidence of the efficacy of other drugs including mast cell inhibitors (sodium cromoglycate), leukotriene receptor antagonists (montelukast), and chemoattractant receptor‐homologous molecule on Th2 cells (CRTH2) antagonist (OC000459) for the treatment of eosinophilic esophagitis (Straumann 2013).

The role of proton pump inhibitors (PPIs) has evolved from a tool to diagnose eosinophilic esophagitis, by excluding other entities associated with esophageal eosinophilia, to a true treatment for the condition (Dellon 2018). Different PPI drugs, such as omeprazole, esomeprazole, pantoprazole, and lansoprazole have been used to induce and maintain remission (Franciosi 2022).

Elimination diets have been used since the description of the disease, providing evidence that eosinophilic esophagitis is predominantly triggered by food antigens (Kelly 1995). Dietary strategies for treatment of eosinophilic esophagitis comprise elemental diet, allergy testing‐directed elimination diet, and empiric elimination diets, avoiding the most frequent food triggers (milk, wheat, egg, soy/legumes, nuts, and fish/seafood).

### How the intervention might work

Inflammation and the resulting symptoms of esophageal dysfunction in eosinophilic esophagitis are thought to result from penetration of the esophageal mucosa by food or aero‐antigens resulting in cellular response and symptoms of esophageal dysfunction. A breach in the integrity of the esophageal epithelium, potentially facilitated by gastric acid exposure and/or carriage of genetic variants that compromise epithelial barrier function, allows ingress of food or aeroallergens leading to initiation of an immune response. Interleukins produced by activated Th2 cells can act directly to recruit eosinophils to the esophagus (IL‐5), or can stimulate the epithelium to express inflammatory genes (IL‐4/IL‐13), including eotaxin‐3, by activation of cell surface receptors that signal through a pathway involving JAKs and STAT6. Esophageal eosinophilia in eosinophilic esophagitis is driven largely by STAT6‐dependent local expression of eotaxin‐3.

Medical therapies for eosinophilic esophagitis have targeted esophageal inflammation broadly (corticosteroids), or targeted biologic mediators, including anti‐IL5 (mepolizumab, reslizumab), anti‐IgE (omalizumab), anti‐IL4r (dupilumab), anti‐IL13 (RPC4046, alias cendakimab; QAX576, alias dectrekumab), and anti‐sialic acid binding Ig‐like lectin 8 (lirentelimab) (Nhu 2022). The proposed mechanism of PPI therapy is by gastric acid suppression leading to a restoration of esophageal barrier function and unrelated PPI‐mediated anti‐inflammatory effects. Elimination diets have been used since the initial description of the disease, providing evidence that eosinophilic esophagitis is predominantly triggered by food antigens. Dietary strategies for treating eosinophilic esophagitis include the elemental diet, the allergy testing‐directed elimination diet, and empiric elimination diets, which avoid the most frequent food triggers (milk, wheat, egg, soy/legumes, nuts, and fish/seafood).

### Why it is important to do this review

There is no universally accepted treatment for eosinophilic esophagitis. Topical corticosteroids, hypoallergenic diets, proton pump inhibitors, biologics, and dilation have all been used to treat eosinophilic esophagitis (Muir 2021). In 2010, a previous version of this review was published that included only three randomized controlled trials (RCTs). The number of published RCTs in pediatric and adult eosinophilic esophagitis that meet our inclusion criteria has grown substantially, with a rapid pace of clinical trial publications and changing outcome metrics. The purpose of this review is to review the evidence from RCTs evaluating non‐surgical interventions for eosinophilic esophagitis.

## Objectives

To evaluate the efficacy and safety of medical interventions for people with eosinophilic esophagitis.

## Methods

### Criteria for considering studies for this review

#### Types of studies

We considered all types of randomized controlled trials (RCTs) for inclusion. We excluded quasi‐randomized trials (using no or non‐appropriate randomization).

#### Types of participants

People of any age with a diagnosis of eosinophilic esophagitis, either with active disease (increased number of eosinophils (at least 15 eos/hpf) on esophageal biopsy and with symptoms of esophageal dysfunction) or inactive disease.

We applied no restrictions on sex, disease duration, or previous medication exposure.

We considered studies with only a subset of eligible participants for inclusion. If the subset had been planned for a subgroup analysis, we explored its impact through the methods described in Subgroup analysis and investigation of heterogeneity. If a subgroup analysis had not been planned, the authoring team liaised to discuss the effect this may have on the planned outcomes and whether further subgroup analysis was necessary.

#### Types of interventions

Studies comparing any medical intervention (e.g. topical corticosteroid, biologic therapy, systemic corticosteroid, leukotriene receptor antagonist, mast cell stabilizer, epicutaneous immunotherapy, proton pump inhibitor) or food elimination diet (e.g. empiric elimination diet, elemental diet), either alone or in combination, to any other intervention.

#### Types of outcome measures

We included both dichotomous and continuous outcomes. When multiple thresholds were prespecified by the study, we chose the most inclusive threshold for the analysis. Study outcomes were not relevant for determining study eligibility.

##### Primary outcomes

- Clinical symptom improvement ‐ defined by the study, either as a clinically successful improvement based on achieving a prespecified threshold on a symptom scoring scale (dichotomous), or absolute or relative symptom scores (continuous) ‐ as measured by the authors at study end and all available intermediate study time points.
- Histological improvement ‐ defined by the study, using a recognized histological grading system (continuous), achieving a prespecified eosinophil count threshold measured per high‐powered microscope field (dichotomous), or absolute or relative eosinophil counts per high‐powered microscope field (continuous) ‐ as measured by the authors at study end and all available intermediate study time points.
- Endoscopic improvement ‐ defined by the study, achieving a prespecified threshold on an endoscopic scoring scale (dichotomous), or absolute or relative endoscopic assessment scores (continuous) ‐ as measured by the authors at study end and all available intermediate study time points.
- Withdrawals due to adverse events ‐ (dichotomous) ‐ as measured by the authors at study end.

##### Secondary outcomes

- Participants with serious adverse events as defined by the study (dichotomous) ‐ as measured by the authors at study end.
- Total number of participants with adverse events as defined by the study (dichotomous) ‐ as measured by the authors at study end.
- Quality of life (QOL) improvement as defined by the study, either as an improvement in quality of life based on achieving a prespecified threshold on a QOL scoring scale (dichotomous), or absolute or relative QOL scores (continuous) ‐ as measured by the authors at study end and all available intermediate study time points.

### Search methods for identification of studies

#### Electronic searches

On 24 October 2021 and 3 March 2023, the Cochrane Gut Information Specialist searched the following sources:

- Cochrane Central Register of Controlled Trials (CENTRAL) via the Cochrane Library (until search date; Appendix 1);
- MEDLINE via Ovid SP (1946 to March 02, 2023; Appendix 2);
- Embase via Ovid SP (1974 to 2023 Week 08; Appendix 3);
- ClinicalTrials.gov (until search date; Appendix 4);
- World Health Organization International Clinical Trials Registry Platform (WHO ICTRP) (until search date; Appendix 5).

There were no limitations to publication date, language, status, or document type in this search.

#### Searching other resources

We handsearched reference lists from the trials identified by electronic searching to identify further relevant trials. Other sources that we searched included reference lists of textbooks, reviews (*Cochrane Database of Systematic Review*s and others), previous trials, and conference proceedings.

### Data collection and analysis

#### Selection of studies

Pairs of authors independently assessed publications identified by the search strategy to determine eligibility based on the above inclusion criteria using Covidence, initially as titles/abstracts, followed by full‐text assessments. Any disagreement was resolved by discussion and consensus among the authors. If consensus could not be reached, a third author was consulted. We documented the results of this process in a flow diagram (PRISMA 2020).

#### Data extraction and management

We collected data from the included studies using a piloted data collection form. Pairs of authors independently extracted data. Disagreements were resolved by discussion and consensus. A third author was consulted when consensus was not reached.

The extracted data included the following:

- General information (title, journal, year, publication type).
- Study information (design, setting, dates, single‐ or multi‐center; RCT duration and endpoints; study outcomes; funding source; conflicts of interest).
- Participant information (disease activity; diagnostic criteria; inclusion and exclusion criteria; age; sex; concomitant medications).
- Intervention and control (type, dose, method of delivery of medication).
- Eligibility (total number of patients randomized and reaching end of study).
- Review outcomes (continuous scoring system or dichotomous success definition; outcome data at study endpoints).

For studies requiring translation we used online translation software, and if this was not adequate we sought translations by speakers of the relevant languages.

#### Assessment of risk of bias in included studies

Using the Cochrane risk of bias tool (Higgins 2011), pairs of authors independently assessed the risk of bias of each included study. We assessed the following factors:

- sequence generation (i.e. randomization method);
- allocation sequence concealment;
- blinding;
- incomplete outcome data (i.e. methods used by investigators to deal with attrition);
- selective outcome reporting (i.e. investigators reported all outcomes); and
- other potential sources of bias (i.e. anything else that could have increased bias).

We judged studies to be of high, low, or unclear risk of bias. Disagreement was resolved by consensus via discussion. A third author resolved cases where consensus was not reached.

#### Measures of treatment effect

We analyzed all data using Review Manager Web (RevMan Web 2022). For dichotomous outcomes, we expressed the treatment effect as a risk ratio (RR) with corresponding 95% confidence interval (CI). For continuous outcomes, we expressed the treatment effect as a mean or standardized mean difference (MD or SMD) with 95% CI.

#### Unit of analysis issues

The participant was the unit of analysis. For studies comparing more than two intervention groups, we made multiple pairwise comparisons between all possible pairs of intervention groups. In multiple‐arm studies comparing different medication dosages to a comparator, we combined the different dosage groups into one. When we analyzed multiple treatment groups separately (e.g. different interventions within medication class groups), we divided the placebo group across the treatment groups.

To deal with repeated observations of participants, we divided shared intervention groups evenly among the comparisons. For dichotomous outcomes, we divided both the number of events and the total number of participants. To deal with events that may re‐occur (e.g. adverse events), we reported on the proportion of participants who experienced at least one event. For continuous outcomes, we only divided the total number of participants, and left the means and standard deviations unchanged. We included cross‐over studies, but we only pooled their data if they were reported separately before and after cross‐over, and we only used pre‐cross‐over data.

In the case of cluster‐RCTs we planned to use study data only if the authors used appropriate statistical methods in taking the clustering effect into account. We would also exclude cluster‐RCTs from a sensitivity analysis to assess their impact on the results.

#### Dealing with missing data

Where data were missing, we contacted the corresponding authors of included studies to supply any unreported or unclear data. For all outcomes, we carried out analyses on an intention‐to‐treat (ITT) basis; that is, we included all participants randomized to each group in the analyses, and we analyzed all participants in the group to which they were allocated regardless of whether they received the allocated intervention.

For dichotomous efficacy outcomes we used the numbers randomized as denominators. As numerators, we used the numbers as reported by the authors. We assumed participants with missing or unclear data to be treatment failures. For safety outcomes, we considered participants with missing or unclear withdrawal data as withdrawals due to adverse events. The denominators used for this outcome were as reported by the authors. For serious and total adverse events we used the numbers of events per participants, as reported by the authors. Outcome data reported for mixes of randomized and non‐randomized participants or post hoc data were discarded and not used for analysis.

For our dichotomous improvement outcomes, we scored an event when the prespecified threshold defined by the study was achieved. In studies that included threshold definitions for both partial and complete improvement, the total number of dichotomous events recorded reflects the sum of both the partial and complete events.

For missing continuous data, we estimated standard deviations from other available data, such as standard errors, or we imputed them using the methods suggested in Higgins 2021b. We conducted analyses for continuous outcomes based on participants completing the trial, in line with available case analysis; this assumes that data were missing at random. If there was a discrepancy between the number randomized and the number analyzed in each treatment group, we calculated and reported the percentage lost to follow‐up in each group.

We attempted to convert data presented in graphic from only to numerical data by digitizing them. When it was not possible to obtain missing data or gain clarity from the study authors, we recorded this in our risk of bias assessments, and rated it for bias based on the extent to which the missing data could bias our outcomes. Data that could not be used in our meta‐analyses due to inadequate reporting (e.g. data not presented per intervention group, no available variance measures, data presented in graphic format which we could not convert) have been presented narratively in the additional tables.

Some studies may have reported data for more than one definition/threshold of a given outcome. We have reported which outcome definitions/thresholds we used in our meta‐analyses in the description of included studies of the results section.

We employed the same methods in our subgroup and sensitivity analyses.

#### Assessment of heterogeneity

We scrutinized studies to ensure that they were clinically homogenous in terms of participants, interventions, comparators, and outcomes. To test for statistical heterogeneity, we used a Chi² test. A P value of less than 0.1 gave an indication of the presence of heterogeneity. We quantified and represented inconsistency using the I² statistic. We interpreted the thresholds as follows (Higgins 2021a):

- 0% to 40%: might not be important;
- 30% to 60%: may represent moderate heterogeneity;
- 50% to 90%; may represent substantial heterogeneity;
- 75% to 100%: considerable heterogeneity.

In the case of considerable statistical heterogeneity, we investigated whether this could be explained on clinical grounds or by risk of bias, in which case we conducted sensitivity analyses. If we could not find reasons for considerable statistical heterogeneity, we presented the results narratively, in detail.

#### Assessment of reporting biases

Our use of an inclusive search strategy minimized most reporting biases. We investigated publication bias using a funnel plot for outcomes with 10 or more studies and determined the magnitude of publication bias by visual inspection of the asymmetry of the funnel plot or other methods mentioned in the *Cochrane Handbook for Systematic Reviews of Interventions* (Egger 1997; Higgins 2021a).

#### Data synthesis

We combined data from individual studies for meta‐analysis when we deemed the interventions, patient groups, and outcomes to be sufficiently similar (determined by consensus). We calculated the pooled RR and corresponding 95% CI for dichotomous outcomes. We calculated the pooled MD and corresponding 95% CI for continuous outcomes that were measured using the same units. We calculated the pooled standardized mean difference (SMD) and 95% CI when different scales were used to measure the same underlying construct. We carried out meta‐analysis using a random‐effects model.

#### Subgroup analysis and investigation of heterogeneity

We carried out subgroup analyses on the primary outcomes to further study the effects of a number of variables on the outcomes, when there were enough studies (Deeks 2021), using the formal test for subgroup differences in RevMan Web 2022. Our planned subgroup analyses were decided by our review team as the characteristics most likely to have an impact on outcomes, and were:

1. age of participants (children < 18 years old, adults > 18 years old or mixed age populations);
2. specific interventions within categories (within the category of biologics these were grouped by mechanism including anti‐IL13/anti‐IL4R, anti‐IL5, anti‐IgE, anti‐sialic acid binding Ig‐like lectin 8; for others by specific intervention);
3. routes of delivery (specific to corticosteroids): esophageal‐specific or not esophageal‐specific through an inhaled route referred to as adapted asthma.

Any planned subgroup analyses that were ultimately not performed were due to no data being available or the original analysis comprising three or fewer studies.

The statistical methods described previously also applied to the subgroup analyses.

#### Sensitivity analysis

We conducted sensitivity analyses for the primary outcomes based on the following:

1. fixed‐effect instead of random‐effects model;
2. removing outcome data from studies that employed non‐validated measures;
3. removing outcome data from non‐peer‐reviewed studies;
4. removing outcome data from studies judged to be at high risk for any risk of bias domain;
5. dichotomous histological reporting thresholds of < 15 eos/hpf, which was the first threshold used at the emergence of the field (Konikoff 2006) and currently employed;
6. dichotomous histological reporting thresholds of < 6 eos/hpf, which is currently advised by the FDA (Reed 2018);
7. dichotomous histological reporting thresholds of < 1 eos/hpf, which signifies full or complete remission (Greuter 2017).

Any planned sensitivity analyses that were ultimately not performed were due to no data being available or the original analysis comprising three or fewer studies.

The statistical methods described previously also applied to the sensitivity analyses.

#### Summary of findings and assessment of the certainty of the evidence

We have presented summary of findings tables and GRADE decisions for all comparisons for all of our dichotomous and continuous primary outcomes. We assessed the overall certainty of evidence supporting the primary and secondary outcomes using the GRADE approach (Schünemann 2021). Evidence retrieved from RCTs is usually regarded as high‐certainty. However, the certainty rating may be downgraded as a result of:

- risk of bias;
- indirect evidence;
- inconsistency (unexplained heterogeneity);
- imprecision;
- publication bias.

GRADE Working Group grades of evidence:

- High certainty: we are very confident that the true effect lies close to that of the estimate of the effect.
- Moderate certainty: we are moderately confident in the effect estimate; the true effect is likely to be close to the estimate of the effect, but there is a possibility that it is substantially different.
- Low certainty: our confidence in the effect estimate is limited; the true effect may be substantially different from the estimate of the effect.
- Very low certainty: we have very little confidence in the effect estimate; the true effect is likely to be substantially different from the estimate of effect.

For the abstract of this review, we decided to focus on the two main comparisons of corticosteroids against placebo and biologics against placebo for induction of remission, reflecting what is most commonly used in current practice.

## Results

### Description of studies

#### Results of the search

The literature search identified 3103 records through database searching and alternative sources. After removal of duplicates, 3089 unique records remained. Examination of the titles and abstracts left 294 records for full‐text screening. After assessing all 294 records, we identified 208 records of 41 studies that met the inclusion criteria and these were included in the review (Characteristics of included studies table).

We excluded 39 studies (45 records; Characteristics of excluded studies table).

We identified 30 ongoing studies (39 records) (Characteristics of ongoing studies table). We categorized two studies (two records) as awaiting classification (Characteristics of studies awaiting classification table).

The results of the search are presented in the PRISMA flow diagram (Figure 1).

**1:**
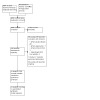
Study flow diagram.

#### Included studies

##### Setting

All studies took place in hospitals and medical centers, in North America, Europe, and Australia.

Twenty‐three studies were multi‐center studies (Assa'ad 2011; Butz 2014; Clayton 2014; Dellon 2017; Dellon 2022; Dellon 2021b; Dellon 2022a; Dellon 2022b; Gupta 2015; Heine 2019; Hirano 2019; Hirano 2020; Hirano 2020f; Hirano 2021; Kliewer 2019; Kliewer 2021; Lucendo 2019; Miehlke 2016; Rothenberg 2015; Rothenberg 2022; Spergel 2012; Straumann 2020; Tytor 2021).

Eighteen studies were single‐center studies (Alexander 2012; Alexander 2017; Bhardwaj 2017; Dellon 2012; Dellon 2019; De Rooij 2022; Dohil 2010; Konikoff 2006; Lieberman 2018; Moawad 2013; Oliva 2018; Peterson 2010; Schaefer 2008; Spergel 2020; Straumann 2010a; Straumann 2010b; Straumann 2011; Straumann 2013).

##### Participants

The 41 RCTs included 3253 participants.

Eleven studies were in pediatric patients (Assa'ad 2011; Dohil 2010; Gupta 2015; Heine 2019; Kliewer 2019; Konikoff 2006; Lieberman 2018; Oliva 2018; Schaefer 2008; Spergel 2012; Spergel 2020), while the rest were in both children and adults.

Four studies were in patients with inactive disease (Alexander 2017; Dellon 2021b; Straumann 2011; Straumann 2020), while the rest were in patients with active disease.

The use of add‐on therapies per included study can be found in Table 21.

**2:** Included studies' characteristics

**Study ID**	**Adults/children or both**	**Interventions**	**Control**	**Induction or maintenance at the time of randomization**	**Disease activity for induction studies/Definition of remission for maintenance studies**	**RCT duration and measurement time points**	**Concomitant medications and diet modifications (mandatory and/or allowed)**
Alexander 2012	Children and adults (18 to 65)	Fluticasone 880 μg twice‐daily, aerosolized/swallowed, 6 weeks	Placebo twice‐daily, aerosolized/swallowed, 6 weeks	Induction	Peak eosinophil level of 20 or more eosinophils (eos)/hpf on esophageal biopsy	Duration: 6 weeks Measurement points: 2 weeks, phone interview, med compliance, MDQ‐2 week, side effects questionnaire Measurement points: 4 weeks, phone interview, med compliance, MDQ‐2 week, side effects questionnaire Measurement points: 6 weeks, EDG, MDQ‐2 week, side effects questionnaire, 24‐hour urine	**PPI** All patients enrolled after the establishment of the consensus definition of EoE in 2007 had at least 1 month of twice‐daily PPI therapy without resolution of dysphagia (fluticasone: 52.4% (11 of 21); placebo: 57.1% (12 of 21)). Repeat endoscopy post‐PPI therapy was not performed routinely before study initiation. The baseline MDQ‐30 documenting dysphagia was completed after PPI treatment. Patients on PPI medications with symptomatic relief of heartburn or regurgitation and with persistent dysphagia were allowed to continue their PPI medications at the same dose during the study (fluticasone: 26.3% (5 of 19); placebo: 0% (0 of 15)). **Diet** Four treatment patients avoided fibrous foods: 2 patients had a partial symptom response and complete histologic response, 1 patient had a complete symptom response and partial histologic response, and 1 patient had a partial symptom response and no histologic response. Two placebo‐treated patients avoided fibrous foods: 1 patient had a complete symptom response and no histologic response, and 1 patient had a partial symptom response and no histologic response. Fibrous food avoidance remained unchanged throughout the study in 5 of the 6 patients. One treatment patient, who had a complete symptomatic response, advanced to an unrestricted diet for the last 2 weeks of the study. **Steroids** Not reported
Alexander 2017	Children and adults (18 to 65)	Montelukast 2 x 10 mg/day, orally at bedtime, 26 weeks	Placebo tablets 2/day, orally at bedtime, 26 weeks	Maintenance (after steroid induction successful on endoscopic screening)	Remission was defined as the absence of dysphagia as defined as an answer of yes to the question of "Have you had trouble swallowing unrelated to a sore throat or cold?”, a severity of at least moderate, and a frequency of at least 1 or more times per week	Duration: 26 weeks Measurement points: side effects: 2, 4, 8, 12, 16, 20, and 24 weeks Symptoms: 2, 4, 8, 12, 20, and 26 weeks	**PPI** Patients on PPI medications with symptomatic relief of heartburn or regurgitation and with persistent dysphagia before topical steroid treatment were allowed to continue their PPI medications at the same dose during the study. **DIet** No restrictions applied. **Steroids** **A.Mandatory prior to randomization** – Patients were given topical steroids in the form of swallowed aerosolized fluticasone at 880 µg twice‐daily OR swallowed budesonide ‐ Rincinolgel 3 mg twice‐daily for at least 6 weeks. Patients kept compliance logs that were reviewed at telephone interviews at 2‐ to 4‐week intervals during the study; 90% compliance was required for continued study inclusion. **B.Allowed** during study – Patients on nasal/inhaled steroids for rhinitis and/or asthma were allowed to continue on the same dose. **C.** No new topical steroid medication was initiated during the study or during the pre study swallowed steroid treatment period.
Assa'ad 2011	Children (2 to 17)	Mepolizumab 3 x 0.55 mg/kg, intravenous infusion, 3 monthly doses Mepolizumab 3 x 2.5 mg/kg, intravenous infusion, 3 monthly doses Mepolizumab 3 x 10 mg/kg, intravenous infusion, 3 monthly doses	Only comparator arms	Induction	Peak eosinophil level of 20 or more eosinophils (eos)/hpf on esophageal biopsy	Duration: 12 weeks Measurement points: histologic, safety, tolerability, mean intraepithelial eosinophil counts, improvement of histopathologic and endoscopic findings, blood eosinophil counts, and frequency and severity of EoE symptoms at 9 to 12 weeks	**PPI** Mepolizumab 3 x 0.55 mg/kg: 6/19 (31.6%) Mepolizumab 3 x 2.5 mg/kg: 6/20 (30.0%) Mepolizumab 3 x 10 mg/kg: 6/20 (30.0%) **Diet** Mepolizumab 3 x 0.55 mg/kg: 4/19 (21.0%) Mepolizumab 3 x 2.5 mg/kg: 6/20 (30.0%) Mepolizumab 3 x 10 mg/kg: 8/20 (40%) **Steroids** Required to terminate steroid therapy.
Bhardwaj 2017	Children and adults (18 to 65)	Beclomethasone diphosphate 80 μg twice‐daily, aerosolized/swallowed, 8 weeks	Placebo twice‐daily, aerosolized/swallowed, 8 weeks	Induction	Peak eosinophil level of 15 or more eosinophils (eos)/hpf on esophageal biopsy	Duration: 8 weeks Measurement points: histologic, symptoms, peripheral blood eosinophil counts, the tissue MCT level, tissue IL‐13, CCL2, CCL‐5, IL‐17F, IL‐10, IL‐25, and thymic stromal lymphopoietin (TSLP) expression all at 8 weeks	**PPI** All patient continued PPI (except n = 1 in the placebo group) **Diet** No diet elimination. During the screening period all patients were asked to discontinue dietary restrictions, if any. **Steroids** During the screening period of 12 weeks before the treatment periods, the enrolled patients were asked to discontinue all previous topical corticosteroids for EoE
Butz 2014	Children and adults (3 to 30)	Fluticasone propionate 880 μg twice‐daily, aerosolized/swallowed, 12 weeks	Placebo twice‐daily, aerosolized/swallowed, 12 weeks	Induction	24 or more eosinophils/hpf in the proximal or distal esophagus while being treated with a PPI for at least 2 months or having a negative pH probe	Duration: 12 weeks Measurement points: histologic and EoE symptom score at 12 weeks	**PPI** Participants were instructed not to change PPI dosage and/or diet therapy during the study. **Diet** Not reported **Steroids** Not reported
Clayton 2014	Children and adults (≥ 15)	Omalizumab 0.016 mg/kg/IgE (IU/mL), subcutaneous every 2 to 4 weeks, 16 weeks	Placebo, subcutaneous every 2 to 4 weeks, 16 weeks	Induction	> 15 eosinophils/hpf in esophageal biopsy specimen, not responsive to maximal‐dose PPI	Duration: 16 weeks Measurement points: histologic and dysphagia symptoms at 16 weeks	**PPI** The participants to were only on proton pump inhibitors during the trial once the consensus criteria for eosinophilic esophagitis were published in 2007. A majority of the participants (all but 5 in the treatment group and 4 in the control group) were treated with high‐dose, twice‐daily proton pump inhibitors for the duration of the study and for at least 8 weeks prior to the initial biopsy and the beginning of the study. **Diet** Not reported **Steroids** Not reported
Dellon 2012	Children and adults (≥ 18)	Budesonide solution (1 mg/2 mL) twice‐daily, nebulized/swallowed, 8 weeks Budesonide solution (1 mg/2 mL) with 5 g of sucralose twice‐daily, swallowed, 8 weeks	Only comparator arms	Induction	Symptoms of esophageal dysfunction and had persistent esophageal eosinophilia (≥ 15 eosinophils in one high‐power field) after 8 weeks or treatment with twice‐daily proton pump inhibitor	Duration: 8 weeks Measurement points: histologic, dysphagia symptom scores, endoscopic, and safety at 8 weeks	**PPI** Previously prescribed PPIs were discontinued as patients included in this study did not have either a symptomatic or histologic response to a high‐dose PPI trial. **Diet** "No dietary elimination therapy was allowed in either group during the study period, and no other concurrent therapy for eosinophilic esophagitis was allowed. " **Steroids** Subjects were excluded if previously treated with topical steroids.
Dellon 2017	Children and adults (11 to 40)	Budesonide oral suspension 2 mg/10 mL twice‐daily, swallowed, 12 weeks	Placebo 10 mL twice‐daily, swallowed, 12 weeks	Induction	Symptoms of esophageal dysfunction and at least 15 intra‐epithelial eosinophils per hpf after an 8‐week, high‐dose PPI	Duration: 12 weeks Measurement points: histologic, dysphagia symptom score, and endoscopic score at 12 weeks	**PPI** PPI‐responsive patients were excluded. PPI‐responsive is defined as < 15 eos/hpf. Changing the PPI regimen for non‐responsive patients was also a reason for exclusion. **Diet** Not reported **Steroids** The use of corticosteroids (topical or systemic) in the 4 weeks preceding the screening endoscopy was an exclusion criterion. Changes to the inhaled corticosteroid regimen were also exclusion criteria.
Dellon 2019	Children and adults (16 to 80)	Budesonide 1 mg/4 mL twice‐daily with 10 g of sucralose, swallowed + placebo inhaler twice‐daily, aerosolized/swallowed, 12 weeks Placebo 4 mL twice‐daily with 10 g of sucralose, swallowed + fluticasone 880 μg twice‐daily, aerosolized/swallowed, 12 weeks	Only comparator arms	Induction	Cases had to have dysphagia or other symptoms of esophageal dysfunction, persistent esophageal eosinophilia (15 eosinophils in at least 1 high‐power field (eos/hpf)) after 8 weeks of treatment with a twice‐daily PPI, and other competing causes of esophageal eosinophilia excluded. A symptom threshold was not required for study entry.	Duration: 8 weeks Measurement points: histologic, dysphagia symptom score, and endoscopic score at 8 weeks	**PPI** No changes in baseline PPI medication dose were allowed during the study period. **Diet** No dietary changes were allowed during the study period. **Steroids** Not reported
Dellon 2021b	Children and adults (11 to 55)	Budesonide oral suspension 2.0 mg twice‐daily	Placebo	Maintenance	Eosinophil histology relapse was defined as an eosinophil count of greater than or equal to (≥) 15 per high‐power field (eos/hpf) from at least 2 of 3 levels of the esophagus. Dysphagia symptom relapse was defined as having at least 4 days of dysphagia (with answer 'Yes' for question 2 in DSQ (Dysphagia Symptom Questionnaire)) in the 2‐week period prior to the scheduled visit, as determined by the DSQ.	Duration: 36 weeks Measurement points: histologic, dysphagia symptom score, endoscopic score at 36 weeks	**PPI** Budesonide oral suspension: 22 (88.0%) Placebo: 20 (87.0%) Participants were 100% prior PPI failures. **Diet** **Steroids** Budesonide oral suspension: 6 (24.0%) Placebo: 3 (13.0%)
Dellon 2022	Children and adults (≥ 12)	Dupilumab 300 mg subcutaneously weekly	Placebo	Induction	"A documented diagnosis of EoE by endoscopic biopsy"	Duration: 24 weeks Measurement points: histologic, dysphagia symptom score, endoscopic score at 24 weeks	**PPI** Not reported Participants were 100% prior PPI failures **Diet** Dupilumab: 17/42 Placebo: 16/39 **Steroids** Not reported
Dellon 2022a	Children and adults (18 to 75)	Active APT‐1011 with 4 arms varying in dosage: 3 mg twice‐daily 3 mg at bedtime 1.5 mg twice‐daily 1.5 mg at bedtime	Placebo disintegrating tablet	Induction	Defined as 3 episodes of dysphagia per week during the last 14 days of the 4‐week baseline symptom assessment phase and a Global EoE Symptom Score of > 3), and active esophageal eosinophilia (after evaluation of 5 biopsies from proximal and distal esophageal locations and at least 1 biopsy with a peak count of 15 eos/HPF) after documentation of failed histologic response on 8 weeks of high‐dose PPI	Duration: 12 weeks Measurement points: Measurement points: histologic, dysphagia symptom score, endoscopic score at 12 weeks	**PPI** Patients on a PPI were required to maintain a stable regimen **Diet** Changes in diet were prohibited **Steroids** Corticosteroids are prohibited. However, randomization was stratified by current esophageal stricture(s) and a positive response to prior corticosteroid use **Biologics and immunomodulator** Biologics and immunomodulator were prohibited
Dellon 2022b	Children and adults (12 to 70)	Lirentelimab 1 + 3 + 3 + 3 + 3 + 3 mg/kg, intravenous infusion, 6 monthly doses Lirentelimab 1 + 1 + 1 + 1 + 1 + 1 mg/kg, intravenous infusion, 6 monthly doses	Placebo 1 + 1 + 1 + 1 + 1 + 1 matching saline, intravenous infusion, 6 monthly doses	Induction	Peak eosinophil level of 15 or more eosinophils (eos)/hpf on esophageal biopsy	Duration: 24 weeks Measurement points: histologic and Dysphagia Symptom Score at 23 to 24 weeks	**PPI** Not reported **Diet** Not reported **Steroids** Not reported
De Rooij 2022	Children and adults (not reported)	Elimination of 4 foods including wheat/gluten, milk, egg, and either soy or legumes (FFED) + amino acid‐based formula (AAF)	Elimination of 4 foods including wheat/gluten, milk, egg, and either soy or legumes (FFED)	Induction	Symptoms of esophageal dysfunction (Straumann Dysphagia Instrument (SDI) score of ≥ 1) and ≥ 15 eosinophils (eos) per microscopic hpf on baseline biopsy	Duration: 6 weeks Measurement points: histologic, endoscopic, clinical, and nutritional outcomes were evaluated between week 1 and week 6	**PPI** Patients were on PPI from baseline **Diet** Dietitian specialized in allergies for extensive nutritional evaluation. To guarantee sufficient intake and to improve diet adherence, patients subsequently received personalized nutritional advice with restriction of gluten, milk, soy, and eggs (four food elimination diet). The amount of prescribed amino acid‐based formula added to the four food elimination diet in the intervention group was 30% of patients’ daily caloric requirements based on body mass index and weekly physical activity. **Steroids** The inability to stop anti‐inflammatory drugs (i.e. topical or systemic steroids) was an exclusion criterion
Dohil 2010	Children (1 to 17)	Budesonide suspension (0.5 mg/2 mL) + PPI	Sterile water + PPI	Induction	Peak eosinophil level of 20 or more eosinophils (eos)/hpf on esophageal biopsy	Duration: 12 weeks Measurement points: histologic, symptomatic, and endoscopic score at 12 weeks	**PPI** Patients were a mixture of being on PPI and not, prior to PPI therapy as part of both arms. Once assigned a group, patients were given PPI as part of the intervention group or the placebo group. **Diet** Patients who were on diet restrictions as part of their treatment to EoE were allowed to continue with these restrictions. Dietary restrictions were reported in a non‐specific pattern throughout the study. Restrictions were limited to: E, eggs; F, fish; milk; nuts; soy and wheat. **Steroids** Patients were excluded from the study of they needed a systemic corticosteroid
Gupta 2015	Children (2 to 18)	Low‐dose OBS: oral budesonide suspension (OBS) 0.05 mg/mL at bedtime and placebo after breakfast for 12 weeks, with a total daily dose of 0.35 mg (2 to 9 years) or 0.50 mg (10 to 18 years), followed by a 3‐week taper period Medium‐dose OBS: oral budesonide suspension (OBS) 0.2 mg/mL at bedtime and placebo after breakfast for 12 weeks, with a total daily dose of 1.4 mg (2 to 9 years) or 2.0 mg (10 to 18 years), followed by a 3‐week taper period High‐dose OBS: oral budesonide suspension (OBS) 0.2 mg/mL at bedtime (hs) and after breakfast for 12 weeks, with a total daily dose of 2.8 mg (2 to 9 years) or 4.0 mg (10 to 18 years), followed by a 3‐week taper period	Placebo twice‐daily at bedtime and after breakfast for 12 weeks with a 3‐week taper period	Induction	Esophageal biopsy must show ≥ 20 eos per HPF (400x, 0.3 mm^2^ HPF) at 2 or more levels of the esophagus following 4 weeks of high‐dose PPI (type, actual dosage not specified)	Duration: 12 weeks Measurement points: clinical symptom scores, and safety at weeks 2, 4, 8, and 12. Histologic at 12 weeks.	**PPI** Four weeks of high‐dose PPI therapy (type, actual dosage not specified) were required for inclusion **Diet** Dietary therapy, environmental therapy, and/or medical regimens (including gastric acid suppression, if any) in effect at the Screening Visit, were continued during treatment **Steroids** Not reported
Heine 2019	Children (1 to 18)	4‐food elimination diet + PPI 4‐food elimination diet (strictly avoiding all foods containing cow’s milk, soy, wheat or egg) Omeprazole: 7.5 kg to 9.9 kg: 5 mg in the morning and 10 mg at night, 10.0 kg to 14.9 kg: 10 mg twice‐daily, 15.0 kg to 19.9 kg: 15 mg twice‐daily, > 20 kg: 20 mg twice‐daily	PPI alone Omeprazole: 7.5 kg to 9.9 kg: 5 mg in the morning and 10 mg at night, 10.0 kg to 14.9 kg: 10 mg twice‐daily, 15.0 kg to 19.9 kg: 15 mg twice‐daily, > 20 kg: 20 mg twice‐daily	Induction	≥ 15 eosinophils per high‐power field; HPF	Duration: 8 to 12 weeks Measurement points: histologic at 8 to 12 weeks	**PPI** Use controlled **Diet** Controlled **Steroids** No steroids allowed
Hirano 2019	Children and adults (18 to 65)	RPC4046 180 mg (n =31), or RPC4046 360 mg (n = 34) subcutaneously once weekly	Placebo subcutaneous injections once weekly	Induction	Symptoms of dysphagia for a minimum of 4 days over 2 weeks (within the 4‐week screening period) and histologic evidence of EoE, defined as a peak count of ≥ 15 eosinophils per high‐power field (eos/hpf; microscope hpf = 0.3 mm^2^) at any 2 of 3 levels of the esophagus (proximal, mid, distal) when off antiinflammatory therapy for EoE	Duration: 16 weeks Measurement points: histologic, dysphagia symptoms, endoscopic, and participants global assessment of disease severity score at 16 weeks	**PPI** If patient was screened while on PPI they had to agree to maintain the same dose over the 16‐week study period **Diet** No mention **Steroids** Excluded
Hirano 2020	Children and adults (18 to 65)	Weekly subcutaneous dupilumab 300 mg (loading dose, 600 mg on day 1) for 12 weeks	Placebo subcutaneously for 12 weeks	Induction	Active esophageal inflammation was to be evident at screening (i.e. peak cell count ≥ 15 eosinophils per high‐power field (eos/HPF): 400 magnification of a 0.3 mm^2^ field) as indicated by esophageal pinch biopsy specimens from at least 2 of 3 esophageal sites from endoscopy performed no more than 2 weeks after at least 8 weeks of treatment with high dose	Duration: 12 weeks Measurement points: Straumann Dysphagia Instrument (SDI) patient‐reported outcome (PRO) score, histologic endoscopic reference score, esophageal distensibility, and safety at 12 weeks	Patients could receive concomitant medications as needed at the investigator’s discretion, except for those that were prohibited. If medically necessary, rescue medications or emergency esophageal dilation could be provided. Patients who received rescue therapy were discontinued from study treatment and considered non‐responders. Prohibited concomitant medications included medications used for the treatment of EoE, allergen immunotherapy, live attenuated vaccines, and any investigational drug other than dupilumab. **PPI** Patients using stable doses of PPIs at screening were permitted to continue the same dosing regimen until the end‐of‐treatment visit; those not using PPIs in the 8 weeks before screening were prohibited from starting them. Prior history of treatment with high‐dose PPIs at baseline: Dupilumab: 23 (100%) Placebo: 24 (100%) PPI treatment ongoing at baseline: Dupilumab: 14 (60.9%) Placebo: 15 (62.5%) **Diet** Patients were instructed not to modify their diets during the study **Steroids** Prohibited. If required for rescue therapy, the patient was discontinued from the study.
Hirano 2020f	Children and adults (12 to 55)	APT‐1011 (fluticasone propionate tablets) at 1.5 mg once in morning and once in the evening, APT‐1011 at 3.0 mg once a day APT‐1011 3.0 mg once a day group had a placebo tablet in the morning bottle and a 3.0 mg fluticasone propionate tablet in the evening bottle	The placebo group had a placebo tablet in both morning and evening bottles	Induction	Esophageal mucosal peak eosinophil count ≥ 24 per high‐power field (HPF) (HPF; radius = 0.275 mm; 400×)	Duration: 8 weeks Measurement points: treatment‐emergent adverse events at 2, 4, 6, and 8 weeks Exploratory outcomes: histologic and endoscopic at 8 weeks. Physician Global Assessment of the participant’s overall EoE activity, the Patient Global Assessment of symptom severity and EEsAI PRO, and Mayo Dysphagia Questionnaire at weeks 4 and 8.	**PPI** Current: APT‐1011 2 x 1.5 mg: 5 (62.5%) APT‐1011 1 x 3 mg: 5 (62.5%) Placebo: 6 (75%); Prior: APT‐1011 2 x 1.5 mg: 3 (37.5%) APT‐1011 1 x 3 mg: 3 (37.5%) Placebo: 2 (25%) **Diet** Not reported **Steroids** Not reported
Hirano 2021	Children and adults (11 to 55)	**Study group 2:** budesonide oral suspension (BOS) 2.0 mg twice‐daily (10 mL at a concentration of 0.2 mg/mL)	Placebo	Induction	(≥ 15 eosinophils/high‐power field (eos/hpf) from at least 2 levels of the esophagus	Duration: 12 weeks Measurement points: Measurement points: histologic, dysphagia symptom score, endoscopic score at 12 weeks	**PPI** Budesonide oral suspension: 176 (82.6%) Placebo: 92 (87.6%) **Diet** Budesonide oral suspension: 11 (10.5%) Placebo: 21 (9.9%) **Steroids** Budesonide oral suspension: 40 (18.8%) Placebo: 19 (18.1%)
Kliewer 2019	Children (6 to 17)	1 ‐ food (milk) elimination diet (1FED); 4 ‐ food (milk, egg, wheat, soy) elimination diet (4FED)	No placebo, only comparator arms	Induction	Histologically confirmed active EoE (≥ 15 eos/hpf) and symptoms of esophageal dysfunction	Duration: 12 weeks Measurement points: histologic, symptomatic, and quality of life at 12 weeks	**PPI** Not reported. Failure of a PPI trial was required for inclusion. **Diet** Not reported **Steroids** Exclusionary.
Kliewer 2021	Children and adults (18 to 60)	1‐food elimination: animal milk 6‐food elimination: animal milk, wheat, egg, soy, tree nuts/peanuts, seafood	No placebo, only comparator arms	Induction	≥ 15 eos/hpf + symptoms and lack of PPI response	Duration: 6 weeks Measurement points: histologic, EoE Histologic Scoring System (EoEHSS), EoE Endoscopic Reference Score (EREFS), EoE Symptom Activity Index (EEsAI), and quality of life (EoE‐QoL‐A) at 6 weeks	**PPI** PPI failure required for inclusion, not clear how concomitant PPI was handled **Diet** Elimination determined by randomization. No other restrictions specified. **Steroids** Excluded
Konikoff 2006	Children (1 to 18)	Swallowed fluticasone propionate (FP) (880 mg/day)	Patients were treated with swallowed FP or placebo. Patients were given identical metered‐dose inhalers of FP or placebo.	Induction	The primary outcome measure, as specified before the study was initiated, was complete histologic response to treatment as defined by a peak eosinophil count of 1 eosinophil in all 400x HPFs in both the proximal and distal esophagus.	Duration: 12 weeks Measurement points: histologic, endoscopic, and vomiting at 12 weeks	**PPI** Fluticasone: 8 (38%) Placebo: 5 (33%) **Acid suppression (PPI or H2‐RA)** Fluticasone: 10 (48%) Placebo: 7 (47%) **Diet** Not reported **Montelukast** Fluticasone: 4 (19%) Placebo: 0 (0%) **Steroids** Not reported
Lieberman 2018	Children (2 to 17)	Participants 2 to 12 years of age ‐ 100 mg of cromolyn ‐ 1 ampule mixed with 1 teaspoon of sugar 4 times daily Participants 13 to 18 years of age ‐ 200 mg cromolyn ‐ 2 ampules mixed with 2 teaspoons of sugar 4 times daily (ClinicalTrials.gov)	Saline ampules, participants 2 to 12 years of age ‐ 1 ampule mixed with 1 teaspoon of sugar 4 times daily Participants 13 to 18 years of age ‐ 2 ampules mixed with 2 teaspoons of sugar 4 times daily (ClinicalTrials.gov)	Induction	≥ 15 eosinophils per high‐power field (eos/hpf) following at least 8 weeks of high‐dose PPI therapy and a normal esophageal pH probe	Duration: 8 weeks Measurement points: symptoms at 4 and 8 weeks. Endoscopic and symptoms at 8 weeks.	**PPI** Not reported **Diet** Not reported **Steroids** Patients on concomitant treatment with swallowed corticosteroids were excluded. Any prior use of swallowed corticosteroids required a 4‐week washout period.
Lucendo 2019	Children and adults (18 to 75)	Budesonide orodispersible tablets (BOT; 1 mg twice‐daily)	Placebo	Induction	Patients had to have a severity of 4 points on a 0 to 10 numerical rating scale (NRS) for either dysphagia or odynophagia for 1 day in the week before randomization. Additionally, Patient’s Global Assessment (PatGA) of EoE activity was to be 4 points on a 0 to 10 NRS. Histologic activity with peak eos ≥ 65/mm^2^ hpf in at least 1 hpf (corresponding to ≥ 20 eos/hpf), as measured in a total of 6 hpf derived from 6 biopsies, 2 each from the proximal, mid, and distal segments of the esophagus.	Duration: 6 weeks Measurement points: histologic at 6 weeks. Dysphagia, EEsAI‐Pro at 2, 4, and 6 weeks	**PPI** Budesonide: 7 (12%) Placebo: 3 (10%) **Diet** Not reported **Steroids** Not reported
Miehlke 2016	Children and adults (18 to 75)	Budesonide effervescent tablet (BET) 2 x 1 mg/day Budesonide effervescent tablet (BET) 2 x 2 mg/day Budesonide viscous suspension 2 x 2 mg/day	Placebo	Induction	Clinical symptoms of esophageal dysfunction (dysphagia score ≥ 3), peak eosinophils (eos) ≥ 65/mm^2^ high‐power fields (hpf) in at least 1 hpf (corresponding to ≥ 20 eos/hpf), and eosinophilic tissue infiltration with a mean cell density ≥ 16 eos/mm^2^, as measured in a total of 30 hpf derived from 6 biopsies, 2 each from the proximal, mid, and distal segments of the esophagus	Duration: 2 weeks Measurement points: histologic, symptomatic, endoscopic, and safety at 2 weeks	**PPI** Patients with a clinicopathological response to a treatment with proton pump inhibitors (PPIs) at a standard dose with a treatment duration of at least 2 weeks were excluded **Diet** Patients with dietary restrictions within 4 weeks prior to screening or during treatment were excluded. Patients who had an intake of grapefruit food/drinks were excluded **Steroids** Patients excluded if they received: Topical/systemic therapies for any reason that may have affected assessment of primary and secondary end points (i.e. systemic glucocorticoids, histamine antagonists, mast cell stabilizers, leukotriene receptor antagonists, biologics, immunosuppressants) concomitantly or within 4 weeks prior to screening Topical therapy (topical steroids, inhaled sodium cromoglycate concomitant or within 2 weeks prior to screening)
Moawad 2013	Children and adults (≥ 18)	Esomeprazole 40 mg once daily 8 weeks	Fluticasone propionate 440 µg twice‐daily 8 weeks	Induction	One clinical symptom of esophageal dysfunction (dysphagia, food impaction, heartburn) with ≥ 15 eosinophils/hpf	Duration: 8 weeks Measurement points: histologic and dysphagia symptom score at 8 weeks	**PPI/steroid** From NCT00895817, patients had to agree to a 1‐month washout of both PPI and steroids to be eligible **Diet** Not reported
Oliva 2018	Children (not reported)	Six‐food elimination diet Swallowed fluticasone Swallowed budesonide Oral viscous budesonide	No placebo, only comparator arms	Induction	Not reported	Duration: 8‐week induction, 34 week maintenance Measurement points: histologic, clinical symptoms and endoscopic scores at 8 and 42 weeks	**PPI** Not reported **Diet** NBot reported **Steroids** Not reported
Peterson 2010	Children and adults (18 to 80)	Esomeprazole (40 mg by mouth every morning) for 8 weeks	Aerosolized, swallowed fluticasone (440 µg by mouth twice a day) for 8 weeks	Induction	≥ 15 eosinophils averaged over 5 high‐power fields on esophageal biopsy in participants with symptoms of dysphagia, food impaction or chest pain	Duration: 8 weeks Measurement points: histologic and dysphagia scores at 8 weeks	**PPI** If patients were on esomeprazole or fluticasone prior the trial, they needed to withhold the treatment for a month prior to be included. One arm received esomeprazole (40 mg by mouth every morning) for 8 weeks. **Diet** Not reported **Steroids** Not reported
Rothenberg 2015	Children and adults (18 to 50)	IV QAX576 6 mg/kg at weeks 0, 4, and 8	IV placebo at weeks 0, 4, and 8	Induction	Peak eosinophil density of 24 cells or greater per high‐power field (hpf; 3400 magnification) in the proximal or distal esophagus validated by a central laboratory pathology service	Duration: 12 weeks Measurement points: histologic and dysphagia symptom score at 12 weeks	**PPI/steroids** Patients already on PPIs, nasal, or inhaled steroids were allowed to continue these throughout the study. **Diet** Patients were instructed to maintain their baseline diet throughout the study.
Rothenberg 2022	Children and adults (≥ 12)	Dupilumab 300 mg subcutaneously weekly	Placebo	Induction	"A documented diagnosis of EoE by endoscopic biopsy"	Duration: 24 weeks Measurement points: histologic and dysphagia symptom scores at 24 weeks	**PPI / Steroids** No information reported for concomitant PPI or steroids. Participants were 100% prior PPI failures. **Diet** Food elimination diet at screening Dupilumab: 60/161 Placebo: 29/79
Schaefer 2008	Children (1 to 18)	Fluticasone: swallowed fluticasone by metered dose inhaler (110 μg per puff for ages 1 to 10 years and 220 μg per puff for ages 11 years or older, 2 puffs 4 times/day) for 4 weeks	Prednisone: oral P suspension/tablet (1 mg/kg/dose twice a day; maximum 30 mg twice a day) for 4 weeks	Induction	Esophageal mucosal biopsy specimens showing ≥ 15 eos/hpf with negative pH probe studies	Duration: 4‐week induction Measurement points: histologic, clinical symptoms and safety at 4 weeks	**PPI** Not reported **Diet** During the entire study, patients continued a regular diet except for foods identified as possible allergens by allergy testing. **Steroids** None of the patients were on corticosteroids at the time of initial endoscopy or at study enrollment.
Spergel 2012	Children (5 to 18)	1, 2, or 3 mg/kg reslizumab	Placebo	Induction	Defined as ≥ 24 eosinophils in ≥ 1 high‐power field (hpf))	Duration: 15 weeks Measurement points: histologic, and the physician’s global assessment score at week 15	**PPI** "Patients were also allowed to take medications for acid reflux if the doses remained stable throughout the study; use of these therapies on an as‐needed basis was not permitted." **Diet** "Patients were instructed to maintain their baseline diet throughout the study." **Steroids** "Patients were allowed to take inhaled corticosteroids, and nasal corticosteroids for allergies if they were started before the first dose of study medication, if the patients had symptoms of eosinophilic esophagitis while taking these medications, and if the doses remained stable during the study period."
Spergel 2020	Children (4 to 17)	Viaskin milk 500 µg participants epicutaneously administered daily (up to 24 hours application per day) with a patch containing 500 µg cows milk proteins	Viaskin placebo participants epicutaneously administered daily (up to 24 hours application per day) with a patch containing a matching placebo formulation	Induction (no previous agent to induce induction prior randomization)	≥ 15 eos/HPF	Duration: 44 weeks Measurement points: histologic at 44 weeks	**PPI** Patients were diagnosed with EoE if EGD and biopsy showed ≥ 15 eos/hpf after at least 2‐month period of being on a high‐dose PPI (1 to 2 mg/kg dose twice‐daily) **Diet** All patient had 2 biopsies. First biopsy whilst being on milk‐rich diet. Second biopsy on a milk‐free diet. Milk was reintroduced after 9 months of randomization, where the primary endpoint was measured at the 11th month. **Steroid** Participants on swallowed corticosteroids for eosinophilic esophagitis were excluded
Straumann 2010a	Children and adults (≥ 18)	Mepolizumab intravenous infusion at a dose of 750 mg diluted in 150 mL of 0.9% sodium chloride solution for the first 2 infusions Day 0 and day 7	Placebo IV day 0 and day 7	Induction	At least one episode of dysphagia per week in the 4 weeks prior to the start of study medication and a peak esophageal eosinophilia of ≥ 20 eosinophils	Duration: 13 weeks Measurement points: histologic, symptomatic and endoscopic at 4 and 13 weeks	Any current anti‐eosinophil treatment was discontinued and patients were directed to avoid any changes in their feeding habits during the entire study period. **PPI** GERD was excluded in all patients by pretreatment with PPIs in standard dosages plus negative endoscopy for signs of reflux disease, and by pH monitoring (optional). **Diet** 1 patient excluded in placebo group due to lack of efficacy and was on elemental diet. **Steroids** Selected patients unresponsive to steroids. No steroids permitted at least 6 weeks prior to starting the trial.
Straumann 2010b	Children and adults (> 14)	Budesonide administered as 0.25 mg/mL suspension twice per day at bedtime and in the morning after breakfast via a nebulizer. Patients instructed swallowing continuously the accumulated liquid.	0.9% saline administered as 4 mL twice per day at bedtime and in the morning after breakfast via a nebulizer. Patients instructed swallowing continuously the accumulated liquid.	Induction	Clinicopathologic definition of esophageal symptoms in combination with ≥ 20 eosinophils per high‐power field	Duration: 2 weeks Measurement points: histologic, dysphagia scores, endoscopic at 2 weeks	**PPI** Previously established proton pump inhibition was continued throughout the study period. Budesonide: 13/18 Placebo: 10/18 **Diet** Not reported **Steroids** Not reported
Straumann 2011	Children and adults (> 14)	0.5 mg/day budesonide as 0.25 mg/mL suspension formulation applied using an inhalation system consisting of a PARI UNI light compressor and PARI TIA nebulizer, twice per day at bedtime and in the morning after breakfast; patients instructed to nebulize the suspension into the oral cavity and to swallow continuously the accumulated liquid	0.9% saline 1 mL via an inhalation system consisting of a PARI UNI light compressor and PARI TIA nebulizer, twice per day at bedtime and in the morning after breakfast; patients instructed to nebulize the suspension into the oral cavity and to swallow continuously the accumulated liquid	Maintenance	Clinically, endoscopically, and histologically confirmed eosinophilic esophagitis after proton pump inhibitor trial	Duration: 50 week maintenance Measurement points: histologic, symptomatic, endoscopic, and safety at 50 weeks	**PPI** Previously established proton pump inhibition was continued throughout the study period. **Diet** Not reported **Steroids** Throughout the study period, participants took no other anti‐eosinophil medication.
Straumann 2013	Children and adults (18 to 75)	OC004549 100 mg tablets, twice‐daily after meals for 8 weeks	Placebo	Induction	Patient with previously clinically, endoscopically, and histologically confirmed EoE (according to Liacouras 2011 definition)	Duration: 8 weeks Measurement points: histologic and physician’s global assessment of disease activity at 8 weeks	**PPI / Steroids** Patients discontinued all specific treatments for EoE (e.g. corticosteroids, leukotriene antagonists, histamine blockers, mast cell stabilizers); medications stopped 2 weeks prior to baseline exam; previously established PPI therapies for secondary reflux were continued throughout the study in a constant dose. **Diet** Not reported
Straumann 2020	Children and adults (18 to 75)	Budesonide orodispersible tablet 0.5 mg twice‐daily and 1.0 mg twice‐daily	Placebo	Maintenance	Previously confirmed diagnosis of PPI‐refractory EoE according to consensus guidelines (Dellon et al. Gastroenterology 2018; Lucendo AJ et al. United European Gastroenterol J. 2017)	Duration: 48 week maintenance Measurement points: histologic, EEsAI‐PRO an 48 weeks	**PPI** Concomitant PPI treatment was to be kept stable. **Diet** Dietary restriction was not permitted. **Steroids** The use of other swallowed topical steroids, systemic glucocorticoids, immunosuppressants or biologic drugs was not permitted.
Tytor 2021	Children and adults (≥ 18)	Mometasone furoate 4 spray doses 50 µg by mouth to be swallowed 4 times daily after meals with no eating or drinking allowed 30 minutes after intake. Duration of treatment is 8 weeks.	Placebo For 8 weeks	Induction	Newly diagnosed EoE with a peak eosinophil count of at least 15 cells per HPF in any area in any of at least 6 esophageal biopsies including at least 3 biopsies from the upper‐respective lower‐third part of the esophagus, and total WDS score ≥ 5	Duration: 8 weeks Measurement points: dysphagia score at 8 weeks	**PPI** PPIs were not allowed from 2 weeks before the start and during the treatment period. **Diet** Not reported **Steroids** Systemic or topical corticosteroid treatment during the last 4 months was not allowed.

AAF: amino acid‐based formula; CG: control group; DB: double‐blind; DSQ: Dysphagia Symptom Questionnaire; EDG: esophagogastroduodenoscopy; EoE: eosinophilic esophagitis; FED: food elimination diet; FFED: four‐food elimination diet; GERD: gastroesophageal reflux disease; HPF/hpf: high‐power field; IG: intervention group; IV: intravenous; MCT: mast cell tryptase; MDQ: Mayo Dysphagia Questionnaire; NR: not reported; PPI: proton pump inhibitor; PRO: patient‐reported outcome; SC: subcutaneous; SDI: Straumann Dysphagia Instrument; WDS: Watson Dysphagia Scale

##### Interventions

###### For induction of remission

1. Fourteen studies compared corticosteroids to placebo for induction of remission (Alexander 2012; Bhardwaj 2017; Butz 2014; Dellon 2017; Dellon 2022a; Dohil 2010; Gupta 2015; Hirano 2020f; Hirano 2021; Konikoff 2006; Lucendo 2019; Miehlke 2016; Straumann 2010b; Tytor 2021).
2. Nine studies compared biologics to placebo for induction of remission (Clayton 2014; Dellon 2022; Dellon 2022b; Hirano 2019; Hirano 2020; Rothenberg 2015; Rothenberg 2022; Spergel 2012; Straumann 2010a).
3. One study compared cromolyn sodium to placebo for induction of remission (Lieberman 2018).
4. One study compared PGD2R antagonist OC000459 to placebo for induction of remission (Straumann 2013).
5. One study compared swallowed fluticasone to oral prednisone for induction of remission (Schaefer 2008).
6. One study compared oral viscous budesonide to swallowed fluticasone for induction of remission (Dellon 2019).
7. Two studies compared esomeprazole to fluticasone for induction of remission (Moawad 2013; Peterson 2010).
8. One study compared a one‐food elimination diet to a four‐food elimination diet for induction of remission (Kliewer 2019).
9. One study compared a one‐food elimination diet to a six‐food elimination diet for induction of remission (Kliewer 2021).
10. One study compared a four‐food elimination diet with omeprazole to omeprazole for induction of remission (Heine 2019).
11. One study compared a four‐food elimination diet with amino acid formula to a four‐food elimination diet for induction of remission (De Rooij 2022).
12. One study compared nebulized swallowed budesonide to viscous swallowed budesonide (Dellon 2012).
13. One study compared Viaskin milk patch to placebo (Spergel 2020).
14. One study compared a low dose of the biologic mepolizumab (0.55 mg/kg) to a medium dose (2.5 mg/kg) and to a high dose (10 mg/kg) (Assa'ad 2011).
15. One study compared a six‐food elimination diet to swallowed fluticasone to swallowed budesonide and to oral viscous budesonide (Oliva 2018).

The duration of induction RCTs ranged from two weeks (Miehlke 2016; Straumann 2010b) to 44 weeks (Spergel 2020).

###### For maintenance of remission

1. Three studies compared corticosteroids to placebo for maintenance of remission (Dellon 2021b; Straumann 2011; Straumann 2020).
2. One study compared leukotriene receptor antagonist to placebo for maintenance of remission (Alexander 2017).

The duration of maintenance RCTs ranged from 36 weeks (Dellon 2021b) to 50 weeks (Straumann 2011).

The earliest RCT was published in 2006 (Konikoff 2006), and there has been an exponential growth in RCTs in recent years.

##### Outcomes

###### Definitions of dichotomous clinical improvement thresholds

1. Three studies derived a dichotomous clinical outcome from the Mayo Dysphagia Questionnaire (two‐week recall; Peloquin 2006). In Alexander 2012 and Alexander 2017, complete symptom response was defined as an answer of “no” to the question, “In the past 2 weeks, have you had trouble swallowing, not associated with other cold symptoms (such as strep throat or mononucleosis)?” on the Mayo dysphagia questionnaire two‐week version. A partial symptom response was defined as an answer of “yes” to the earlier‐described question and a decrease in the severity of at least two levels (or to a level of “Doesn't bother me at all”), or a decrease in the frequency of at least one level. In Rothenberg 2015, the sum change of Mayo Dysphagia Questionnaire items 1, 2, 4, 9, 10, 13, 14, 16, 20, and 21 from baseline was computed at end of therapy. A positive sum change was scored as an improvement, a negative sum change was scored as a worsening, and a zero‐sum change was scored as unchanged.
2. Two studies derived a dichotomous clinical outcome from the Dysphagia Symptom Questionnaire (DSQ; Hudgens 2017). In Dellon 2021b, efficacy was defined as maintenance of the ≥ 30% reduction in DSQ score from baseline that was achieved during the induction phase. Improvement as defined by Dellon 2021b, was scored as a dichotomous event. In Hirano 2021, efficacy was defined as a ≥ 30% reduction in DSQ score from baseline at end of therapy.
3. Two studies derived a dichotomous clinical outcome from the Eosinophilic Esophagitis Activity Index (EEsAI; Schoepfer 2014). In Hirano 2020, efficacy was defined as a ≥ 40% improvement in EEsAI score from baseline to end of therapy. In Straumann 2020, efficacy was defined as score of ≤ 20 on the EEsAI at end of therapy.
4. One study derived a dichotomous clinical outcome from the Dysphagia Symptom Score (DSS; Straumann 2010). In Straumann 2010b, frequency and intensity of dysphagia events were scored on a scale of 1 to 4 and 1 to 5, respectively. The Dysphagia Symptom Score was the sum of the two. A clinical response was defined as a decrease in the Dysphagia Symptom Score of at least three points compared with baseline.
5. One study derived a dichotomous clinical outcome from the EoE Clinical Symptom Score (EoE CSS; Dohil 2010). In Gupta 2015, efficacy was defined as a ≥ 50% reduction in the EoE CSS from baseline to end of therapy.
6. One study derived a dichotomous clinical outcome from an esophagus‐related symptom score (Straumann 2003). In Straumann 2010a, clinical efficacy was defined as an improvement of ≥ 1 grade of the esophagus‐related symptom score from baseline to end of therapy.
7. One study derived a dichotomous clinical outcome from a pair of numerical rating scales (0 to 10) that individually assessed dysphagia and odynophagia (Lucendo 2019). In Lucendo 2019, clinical efficacy was defined as a score of ≤ 2 on each rating scale on each day of the week before end of therapy.
8. One study derived a dichotomous clinical outcome from the frequency of dysphagia and/or heartburn (Bhardwaj 2017). In Bhardwaj 2017, clinical efficacy was defined as a decrease in the frequency of dysphagia and/or heartburn from baseline to the end of therapy.
9. One study derived a dichotomous clinical outcome from the presence or absence of the presenting symptoms by patient/guardian report and by physician assessment (Schaefer 2008). In Schaefer 2008, clinical efficacy was defined as the absence of presenting symptoms at end of therapy.
10. One study derived a dichotomous clinical outcome from a Physician's Eosinophilic Esophagitis Global Assessment Score (PGA, Schoepfer 2014). In Spergel 2012, clinical efficacy was defined as an improvement of ≥ 1 level on the Physician's Eosinophilic Esophagitis Global Assessment Score from baseline to end of therapy.
11. Twenty‐seven studies did not define a dichotomous clinical outcome or did not publish data for a dichotomous clinical outcome in a form that could be used by this review (Assa'ad 2011; Butz 2014; Clayton 2014; Dellon 2012; Dellon 2017; Dellon 2019; Dellon 2022; Dellon 2022a; Dellon 2022b; De Rooij 2022; Dohil 2010; Heine 2019; Hirano 2019; Hirano 2020f; Kliewer 2019; Kliewer 2021; Konikoff 2006; Lieberman 2018; Miehlke 2016; Moawad 2013; Oliva 2018; Peterson 2010; Rothenberg 2022; Spergel 2020; Straumann 2011; Straumann 2013; Tytor 2021).

###### Scales used for clinical improvement continuous measurement

1. Six studies reported a continuous clinical outcome based on the Dysphagia Symptom Questionnaire (DSQ; Hudgens 2017). Dellon 2019 and Hirano 2021 reported mean Dysphagia Symptom Questionnaire scores at end of therapy. Dellon 2017, Dellon 2022, Dellon 2021b, and Rothenberg 2022 reported mean change in Dysphagia Symptom Questionnaire score from baseline to end of therapy.
2. Five studies reported a continuous clinical outcome based on the Straumann Dysphagia Instrument (SDI; Straumann 2010). Straumann 2010b and Straumann 2011 reported the mean Straumann Dysphagia Instrument score at end of therapy. Miehlke 2016, Hirano 2020, and De Rooij 2022 reported the mean change in Straumann Dysphagia Instrument score from baseline to end of therapy.
3. Two studies reported a continuous clinical outcome based on the Pediatric Eosinophilic Esophagitis Symptom Score version 2.0 (PEESS V2.0; Franciosi 2011). Kliewer 2019 and Spergel 2020 reported mean Pediatric Eosinophilic Esophagitis Symptom Scores version 2.0 at end of therapy.
4. Two studies reported a continuous clinical outcome based on the Mayo Dysphagia Questionnaire (two‐week recall; Peloquin 2006). Moawad 2013 and Rothenberg 2015 reported mean Mayo Dysphagia Questionnaire (two‐week recall) scores at end of therapy.
5. One study reported a continuous clinical outcome based on the Pediatric Eosinophilic Esophagitis Symptom Score version 1.0 (PEESS V1.0; Pentiuk 2009). Lieberman 2018 reported mean Pediatric Eosinophilic Esophagitis Symptom Scores version 1.0 at end of therapy.
6. Two studies reported a continuous clinical outcome based on the Eosinophilic Esophagitis Activity Index (EEsAI; Schoepfer 2014). Kliewer 2021 and Straumann 2020 reported mean Eosinophilic Esophagitis Activity Index scores at end of therapy.
7. One study reported a continuous clinical outcome based on the proportion of days that participants reported difficulty in swallowing averaged over the seven days prior to the clinic visit (Straumann 2010a). Straumann 2010a reported the mean proportion of dysphagia‐free days in the week prior to the last clinic visit at end of therapy.
8. One study reported a continuous clinical outcome based on the Dysphagia Scale (DiSario 2002). Peterson 2010 reported mean Dysphagia Scale score at end of therapy.
9. One study reported a continuous clinical outcome based on the Watson Dysphagia Score (WDS; Dakkak 1992). Tytor 2021 reported mean change in Watson Dysphagia Score from baseline to end of therapy.
10. One study reported a mean continuous clinical outcome, at end of therapy, based on the following criteria: 0 = no dysphagia; 1 = solid food dysphagia monthly; 2 = solid food dysphagia < weekly; 3 = solid food dysphagia > weekly and < daily; 4 = solid food dysphagia daily; 5 = solid food dysphagia with every meal; and 6 = dysphagia for solid and liquid food (Clayton 2014).
11. One study reported a continuous clinical outcome based on the Mayo Dysphagia Questionnaire (30‐day recall; McElhiney 2009). Dellon 2012 reported mean Mayo Dysphagia Questionnaire (30‐day recall) scores at end of therapy.
12. One study reported a continuous clinical outcome based on the sum of scores from a visual dysphagia questionnaire (VDQ; Straumann 2013) and chest pain as recorded by the Straumann Dysphagia Instrument (SDI; Straumann 2010). Straumann 2013 reported mean composite visual dysphagia questionnaire/Straumann Dysphagia Instrument chest pain scores at end of therapy.
13. One study reported a continuous clinical outcome based on a symptom scoring tool (Aceves 2009). Dohil 2010 reported the mean symptom score at end of therapy.
14. One study reported a continuous clinical outcome based on Daily Dysphagia Symptom Diary (DSD) scores (DSD; Hirano 2019). Hirano 2019 reported mean change in Daily Dysphagia Symptom Diary scores from baseline to end of therapy.
15. Fifteen studies did not report a continuous clinical outcome or did not publish data for a clinical continuous outcome in a form that could be used by this review (Alexander 2012; Alexander 2017; Assa'ad 2011; Bhardwaj 2017; Butz 2014; Dellon 2022a; Dellon 2022b; Gupta 2015; Heine 2019; Hirano 2020f; Konikoff 2006; Lucendo 2019; Oliva 2018; Schaefer 2008; Spergel 2012).

###### Definitions of dichotomous histological improvement thresholds

1. Three studies reported a dichotomous histological threshold of < 20 mean peak eos/hpf at end of therapy (Dohil 2010; Straumann 2010b; Straumann 2011).
2. Fifteen studies reported a dichotomous histological threshold of < 15 mean peak eos/hpf at end of therapy (Butz 2014; Dellon 2012; Dellon 2019; Dellon 2022; Dellon 2021b; De Rooij 2022; Hirano 2019; Hirano 2020; Hirano 2020f; Hirano 2021; Kliewer 2019; Kliewer 2021; Peterson 2010; Spergel 2012; Straumann 2020).
3. One study reported a dichotomous histological threshold of ≤ 7 mean peak eos/hpf at end of therapy (Moawad 2013).
4. Eight studies reported a dichotomous histological threshold of ≤ 6 mean peak eos/hpf at end of therapy (Assa'ad 2011; Dellon 2017; Dellon 2022a; Dellon 2022b; Gupta 2015; Konikoff 2006; Rothenberg 2022; Straumann 2010a).
5. Two studies reported a dichotomous histological threshold of < 5 mean peak eos/hpf at end of therapy (Lucendo 2019; Miehlke 2016).
6. One study defined a dichotomous complete histologic response as a decrease in the mean eosinophil level of 90% from baseline to end of therapy. A partial response was defined as a decrease of more than 50% from baseline to end of therapy (Alexander 2012).
7. One study defined dichotomous histologic response as a decrease in mean peak eos/hpf of 75% from baseline to end of therapy (Rothenberg 2015).
8. One study defined dichotomous complete histologic response as "normal biopsy specimens" at end of therapy (Schaefer 2008).
9. Nine studies did not report a dichotomous histologic response or did not publish data for a dichotomous histologic outcome in a form that could be used by this review (Alexander 2017; Bhardwaj 2017; Clayton 2014; Heine 2019; Lieberman 2018; Oliva 2018; Spergel 2020; Straumann 2013; Tytor 2021).

###### Scales used for histological improvement continuous measurement

1. Twenty‐three studies reported continuous histologic outcomes based on mean peak counts of eosinophils per high‐powered microscope field. Six studies reported a continuous histologic outcome as the change in mean peak eos/hpf from baseline to end of therapy (Dellon 2017; De Rooij 2022; Hirano 2019; Hirano 2020; Kliewer 2021; Straumann 2020). Seventeen studies reported a continuous histologic outcome as mean peak eos/hpf at end of therapy (Bhardwaj 2017; Clayton 2014; Dellon 2012; Dellon 2019; Dohil 2010; Hirano 2021; Lieberman 2018; Moawad 2013; Peterson 2010; Rothenberg 2015; Schaefer 2008; Spergel 2012; Spergel 2020; Straumann 2010a; Straumann 2010b; Straumann 2011; Straumann 2013).
2. Eighteen studies did not report a continuous histologic response or did not publish data for a continuous histologic outcome in a form that could be used by this review (Alexander 2012; Alexander 2017; Assa'ad 2011; Butz 2014; Dellon 2022; Dellon 2021b; Dellon 2022a; Dellon 2022b; Gupta 2015; Heine 2019; Hirano 2020f; Kliewer 2019; Konikoff 2006; Lucendo 2019; Miehlke 2016; Oliva 2018; Rothenberg 2022; Tytor 2021).

###### Definitions of dichotomous endoscopic improvement thresholds

1. Two studies defined a dichotomous endoscopic outcome from histologic grading (Schaefer 2008). In Schaefer 2008 and Straumann 2010a, improvement of ≥ 1 histologic grade was scored as a dichotomous endoscopic event.
2. One study derived a dichotomous endoscopic outcome from the Endoscopic Reference Score (EREFS; Hirano 2013). In Hirano 2020f, efficacy was defined as the sign of the change in EREFS score from baseline to end of therapy.
3. One study derived a dichotomous endoscopic outcome from esophageal furrows (Konikoff 2006). In Konikoff 2006, the lack of esophageal furrows at end of therapy was scored as a dichotomous endoscopic event.
4. One study defined a dichotomous endoscopic outcome from endoscopic findings (Alexander 2012). In Alexander 2012, resolution of all endoscopic findings was scored as a dichotomous endoscopic event.
5. Thirty‐five studies did not report a dichotomous endoscopic response or did not publish data for a dichotomous endoscopic outcome in a form that could be used by this review (Alexander 2017; Assa'ad 2011; Bhardwaj 2017; Butz 2014; Clayton 2014; Dellon 2012; Dellon 2017; Dellon 2019; Dellon 2022; Dellon 2021b; Dellon 2022a; Dellon 2022b; Dohil 2010; Gupta 2015; Heine 2019; Hirano 2019; Hirano 2020; Hirano 2021; Kliewer 2019; Kliewer 2021; Lieberman 2018; Lucendo 2019; Miehlke 2016; Moawad 2013; Oliva 2018; Peterson 2010; Rothenberg 2015; Rothenberg 2022; Spergel 2012; Spergel 2020; Straumann 2010b; Straumann 2011; Straumann 2013; Straumann 2020; Tytor 2021).

###### Scales used for endoscopic improvement continuous measurement

1. Thirteen studies reported continuous endoscopic outcomes based on the Endoscopic Reference Score (EREFS; Hirano 2013). Eight studies reported the mean change in Endoscopic Reference Score from baseline to end of therapy (Dellon 2017; Dellon 2022; Dellon 2021b; Dellon 2022a; Hirano 2020; Kliewer 2019; Kliewer 2021; Spergel 2020). Five studies reported mean Endoscopic Reference Score at end of therapy (Dellon 2019; Hirano 2019; Hirano 2021; Lucendo 2019; Straumann 2020).
2. One study reported continuous endoscopic outcomes based on an Endoscopy Scoring Tool (EST, Aceves 2009). In Dohil 2010, the mean score from the Endoscopy Scoring Tool was reported at end of therapy.
3. One study reported continuous endoscopic outcomes based on endoscopic findings (Straumann 2013). In Straumann 2013, the mean endoscopic findings score was reported at end of therapy.
4. Twenty‐five studies did not report a continuous endoscopic response or did not publish data for a continuous endoscopic outcome in a form that could be used by this review (Alexander 2017; Assa'ad 2011; Bhardwaj 2017; Butz 2014; Clayton 2014; Dellon 2012; Dellon 2022b; De Rooij 2022; Gupta 2015; Heine 2019; Hirano 2020f; Konikoff 2006; Lieberman 2018; Miehlke 2016; Moawad 2013; Oliva 2018; Peterson 2010; Rothenberg 2015; Rothenberg 2022; Schaefer 2008; Spergel 2012; Straumann 2010a; Straumann 2010b; Straumann 2011; Tytor 2021).

###### Withdrawals due to adverse events

1. Information about withdrawals due to adverse events was reported for 39 studies (Alexander 2012; Alexander 2017; Assa'ad 2011; Bhardwaj 2017; Butz 2014; Clayton 2014; Dellon 2012; Dellon 2017; Dellon 2019; Dellon 2022; Dellon 2021b; Dellon 2022a; Dellon 2022b; De Rooij 2022; Dohil 2010; Gupta 2015; Heine 2019; Hirano 2019; Hirano 2020; Hirano 2020f; Hirano 2021; Kliewer 2019; Kliewer 2021; Konikoff 2006; Lieberman 2018; Lucendo 2019; Miehlke 2016; Moawad 2013; Peterson 2010; Rothenberg 2015; Schaefer 2008; Spergel 2012; Spergel 2020; Straumann 2010a; Straumann 2010b; Straumann 2011; Straumann 2013; Straumann 2020; Tytor 2021).
2. Two studies did not report information about withdrawals due to adverse events or did not publish data for withdrawals due to adverse events in a form that could be used by this review (Oliva 2018; Rothenberg 2022).

###### Serious adverse events

1. Thirty‐seven studies reported information about serious adverse events (as defined by the study) (Alexander 2012; Alexander 2017; Assa'ad 2011; Bhardwaj 2017; Butz 2014; Clayton 2014; Dellon 2012; Dellon 2017; Dellon 2019; Dellon 2022; Dellon 2021b; Dellon 2022b; De Rooij 2022; Dohil 2010; Gupta 2015; Hirano 2019; Hirano 2020; Hirano 2020f; Hirano 2021; Kliewer 2019; Kliewer 2021; Konikoff 2006; Lieberman 2018; Lucendo 2019; Miehlke 2016; Moawad 2013; Peterson 2010; Rothenberg 2015; Schaefer 2008; Spergel 2012; Spergel 2020; Straumann 2010a; Straumann 2010b; Straumann 2011; Straumann 2013; Straumann 2020; Tytor 2021).
2. Four studies did not report information about serious adverse events (as defined by the study) or did not publish data for serious adverse events in a form that could be used by this review (Dellon 2022a; Heine 2019; Oliva 2018; Rothenberg 2022).

###### Total adverse events

1. Thirty‐three studies reported information about adverse events (as defined by the study) (Alexander 2012; Alexander 2017; Assa'ad 2011; Butz 2014; Clayton 2014; Dellon 2012; Dellon 2017; Dellon 2019; Dellon 2021b; Dellon 2022a; Dellon 2022b; De Rooij 2022; Dohil 2010; Gupta 2015; Hirano 2019; Hirano 2020; Hirano 2020f; Hirano 2021; Kliewer 2019; Kliewer 2021; Konikoff 2006; Lucendo 2019; Miehlke 2016; Moawad 2013; Peterson 2010; Rothenberg 2015; Spergel 2012; Spergel 2020; Straumann 2010a; Straumann 2010b; Straumann 2011; Straumann 2020; Tytor 2021).
2. Eight studies did not report information about adverse events (as defined by the study) or did not publish data for adverse events in a form that could be used by this review (Bhardwaj 2017; Dellon 2022; Heine 2019; Lieberman 2018; Oliva 2018; Rothenberg 2022; Schaefer 2008; Straumann 2013).

###### Quality of life

1. Four studies reported continuous quality of life outcomes based on the Adult Eosinophilic Esophagitis Quality of Life questionnaire (EoE QoL‐A; Taft 2011). Lucendo 2019 and Straumann 2020 reported mean Adult Eosinophilic Esophagitis Quality of Life questionnaire scores at end of therapy. Hirano 2020 and Kliewer 2021 reported mean change in Adult Eosinophilic Esophagitis Quality of Life questionnaire scores from baseline to end of therapy.
2. One study reported continuous quality of life outcomes based on the Pediatric Quality of Life 4.0, Eosinophilic Esophagitis Module (PedsQl 4.0 EoE; Franciosi 2013). Kliewer 2019 reported mean change in Pediatric Quality of Life 4.0, Eosinophilic Esophagitis from baseline to end of therapy.
3. Thirty‐six studies did not report a continuous quality of life score or did not publish data for quality of life in a form that could be used by this review (Alexander 2012; Alexander 2017; Assa'ad 2011; Bhardwaj 2017; Butz 2014; Clayton 2014; Dellon 2012; Dellon 2017; Dellon 2019; Dellon 2022; Dellon 2021b; Dellon 2022a; Dellon 2022b; De Rooij 2022; Dohil 2010; Gupta 2015; Heine 2019; Hirano 2019; Hirano 2020f; Hirano 2021; Konikoff 2006; Lieberman 2018; Miehlke 2016; Moawad 2013; Oliva 2018; Peterson 2010; Rothenberg 2015; Rothenberg 2022; Schaefer 2008; Spergel 2012; Spergel 2020; Straumann 2010a; Straumann 2010b; Straumann 2011; Straumann 2013; Tytor 2021).

##### Contact with authors

We contacted authors of 35 studies with requests for data and clarification where risk of bias was unclear (Alexander 2012; Alexander 2017; Assa'ad 2011; Bhardwaj 2017; Butz 2014; Clayton 2014; Dellon 2012; Dellon 2017; Dellon 2019; Dellon 2021b; Dellon 2022; Dellon 2022a; Dellon 2022b; De Rooij 2022; Dohil 2010; Gupta 2015; Heine 2019; Hirano 2019; Hirano 2020; Hirano 2020f; Hirano 2021; Kliewer 2019; Kliewer 2021; Lieberman 2018; Miehlke 2016; Moawad 2013; Oliva 2018; Peterson 2010; Rothenberg 2015; Rothenberg 2022; Spergel 2020; Straumann 2010b; Straumann 2011; Straumann 2013; Straumann 2020; Tytor 2021). We received responses from all except 11 (Assa'ad 2011; Bhardwaj 2017; Clayton 2014; De Rooij 2022; Heine 2019; Hirano 2020f; Hirano 2021; Oliva 2018; Straumann 2010b; Straumann 2011; Straumann 2013).

##### Funding sources and conflicts of interest

Twenty‐six studies received funding from pharmaceutical companies (Alexander 2017; Assa'ad 2011; Clayton 2014; Dellon 2012; Dellon 2017; Dellon 2022; Dellon 2021b; Dellon 2022a; Dellon 2022b; Dohil 2010; Gupta 2015; Hirano 2019; Hirano 2020; Hirano 2020f; Hirano 2021; Lucendo 2019; Miehlke 2016; Rothenberg 2015; Rothenberg 2022; Spergel 2012; Spergel 2020; Straumann 2010a; Straumann 2010b; Straumann 2011; Straumann 2013; Straumann 2020).

Thirteen studies received funding from universities, foundations, medical associations or research institutions, and no funding from pharmaceutical companies (Alexander 2012; Bhardwaj 2017; Butz 2014; Dellon 2019; Heine 2019; Kliewer 2019; Kliewer 2021; Konikoff 2006; Lieberman 2018; Moawad 2013; Peterson 2010; Schaefer 2008; Tytor 2021).

All studies that reported conflicts of interest had authors with conflicts of interest, except three (Bhardwaj 2017; Clayton 2014, Moawad 2013).

Two studies did not report on their funding or conflicts of interest (De Rooij 2022; Oliva 2018). Six studies did not report on conflicts of interest (Heine 2019; Kliewer 2019; Kliewer 2021; Konikoff 2006; Peterson 2010; Schaefer 2008).

More details about the funding and conflicts of interest of the included studies can be found in the Characteristics of included studies tables.

Table 21 is a summary of key characteristics of the included studies.

#### Excluded studies

We excluded 39 studies (45 records) for the reasons presented in the Characteristics of excluded studies table, summarized below.

- Twenty‐eight studies were excluded due to the wrong study design (Ceves 2005; Della 2017; Eluri 2017; Eluri 2017a; EUCTR2014‐002465‐30‐IT 2014; Francis 2012; Helou 2008; Hudgens 2017; JPRN‐UMIN000021041 2016; JPRN‐UMIN000026704 2017; Kagalwalla 2006; Kruszewski 2016; Kuzumoto 2021; Molina‐Infante 2017; NCT01498497 2011; NTR4892 2014; Safroneeva 2015; Safroneeva 2018; Safroneeva 2018a; Savarino 2015; Song 2020; Spergel 2002; Spergel 2005; Syverson 2020; Vazquez‐Elizondo 2013; Wang 2017; Warners 2016; Wechsler 2017)
- Seven studies were excluded due to the wrong population (Braathen 2006; Comer 2017; Dellon 2020c; Hefner 2016; Tripp 2017; Wright 2020; Wright 2021).
- One study was excluded for the wrong intervention (Kavitt 2016).
- Three studies were abandoned RCTs without results (NCT01458418 2011; NCT01702701 2012; NCT01821898 2013).

### Risk of bias in included studies

The results of our risk of bias assessments are presented in Figure 2 and the risk of bias tables in the Characteristics of included studies table. We conducted our initial assessment using the information presented in the published papers. In studies where the risk of bias assessment was unclear, we sought clarification from at least one author or contact person (or both) per study. Where we received responses, we adapted our initial assessment accordingly.

**2:**
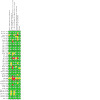
Risk of bias summary: review authors' judgments about each risk of bias item for each included study.

#### Allocation

Three studies did not sufficiently describe randomization and therefore were at unclear risk of bias (Assa'ad 2011; De Rooij 2022; Oliva 2018). We rated all other studies at low risk of bias for randomization.

We rated six studies at unclear risk of bias for allocation concealment, as they did not provide enough information about their selection and allocation concealment processes (Hirano 2020f; Hirano 2021; Oliva 2018; Straumann 2010b; Straumann 2011; Straumann 2013). We rated all other studies at low risk of bias for allocation concealment.

#### Blinding

We rated eight studies at high risk of performance bias for not blinding participants and/or personnel (Dellon 2012; Heine 2019; Kliewer 2019; Kliewer 2021; Moawad 2013; Oliva 2018; Peterson 2010; Schaefer 2008), and three studies at unclear risk, as not enough information was available (Clayton 2014; De Rooij 2022; Dohil 2010). We rated all other studies at low risk for performance bias.

We rated detection bias as high risk in three studies, for not blinding outcome assessors (Heine 2019; Oliva 2018; Peterson 2010), and five studies at unclear risk (Assa'ad 2011; De Rooij 2022; Straumann 2010b; Straumann 2011; Straumann 2013). We rated all other studies at low risk for detection bias.

#### Incomplete outcome data

We rated attrition bias unclear in four studies where it was not possible to judge whether incomplete outcome data had affected outcomes (Bhardwaj 2017; Heine 2019; Kliewer 2019; Oliva 2018). We rated all other studies at low risk for attrition bias.

#### Selective reporting

We rated the risk of bias for selective reporting as high in three studies (Assa'ad 2011; Heine 2019; Peterson 2010), and as unclear in eight studies (Alexander 2017; Bhardwaj 2017; Butz 2014; Clayton 2014; Hirano 2020f; Miehlke 2016; Oliva 2018; Rothenberg 2022). We rated all other studies at low risk of reporting bias.

#### Other potential sources of bias

We rated two studies as unclear for other potential sources of bias (Kliewer 2021; Oliva 2018). We rated all other studies at low risk of other bias.

### Effects of interventions

See: Table 1; Table 2; Table 3; Table 4; Table 5; Table 6; Table 7; Table 8; Table 10; Table 11; Table 12; Table 13; Table 14; Table 15; Table 16; Table 17; Table 18; Table 19; Table 20

All outcome data used in our analyses can be found in Table 22; Table 23; Table 9; Table 24; Table 25.

**3:** Primary outcome ‐ clinical improvement

**Study ID**	**Validated symptom scoring system**	**Continuous or dichotomous**	Outcome data ‐ clinical symptom treatment success **at study endpoint**
Alexander 2012	Mayo Dysphagia Questionnaire (MDQ)‐ 2 week Peloquin 2006 Validated	Dichotomous: A complete symptom response was defined as an answer of “no” to the question, “In the past 2 weeks, have you had trouble swallowing, not associated with other cold symptoms (such as strep throat or mononucleosis)?” on the Mayo Dysphagia Questionnaire 2‐week version. A partial symptom response was defined as an answer of “yes” to the earlier‐described question and a decrease in the severity of at least 2 levels (or to a level of “Doesn't bother me at all”), or a decrease in the frequency of at least 1 level. If there was a decrease in one variable (frequency or severity) and an increase in the other variable then this was classified as no response.	Partial or complete response at end of trial (used for clinical dichotomous analysis): Fluticasone: 12/21 Placebo: 7/21 Complete at end of trial: Fluticasone: 9/21 Placebo: 6/21
Alexander 2017	Mayo Dysphagia Questionnaire (MDQ) ‐ 2 weeks Peloquin 2006 Validated	Dichotomous Remission was defined as the absence of dysphagia	In remission at end of trial (used for clinical dichotomous analysis): Montelukast: 8/20 Placebo: 5/21
Assa'ad 2011	Pain in stomach severity score Regurgitation bothersome score Feeling something stuck in throat bothersome score Flood 2008 Not validated	Continuous The presence and severity of the following symptoms were assessed: abdominal and chest or throat pain, regurgitation, vomiting, solid and liquid food dysphagia (age 8 to 17 years only), difficulty drinking, and difficulty eating solid foods. Reported as mean CI. Pain in stomach severity score: 0 = none 1 = a little 2 = somewhat 3 = quite a bit 4 = a whole lot Regurgitation bothersome score: 1 = not bothered at all 2 = bothered a little 3 = somewhat bothered 4 = bothered quite a lot 5 = bothered a whole lot Feeling something stuck in throat bothersome score: no details given apart from that it is applicable to 8 to 17 years	No primary outcome defined, no aggregate score defined. Data not used. **Pain in stomach severity score:** ‐0.277 (‐0.617, 0.062), ‐0.149 (‐0.412, 0.115), ‐0.157 (‐0.458, 0.144) **Proportion of days with pain in stomach:** ‐14.02 (‐25.93,‐2.12), ‐12.44 (‐21.66, ‐3.21), ‐10.11 (‐20.70, 0.48) **Pain in chest/throat severity score:** ‐0.419 (‐0.796,‐0.042), ‐0.063 (‐0.356, 0.230), ‐0.049 (‐0.382, 0.285) **Proportion of days with pain in chest/throat:** ‐24.37 (‐39.34,‐9.40), ‐5.09 (‐16.64, 6.47), ‐10.16 (‐23.38, 3.06) **Regurgitation bothersome score:** ‐0.307 (‐0.741, 0.128), 0.017 (‐0.320, 0.354), ‐0.047 (‐0.431, 0.337) **Proportion of days with regurgitation:** ‐10.26 (‐24.44, 3.91), 3.8 (‐7.32, 14.93), ‐4.64 (‐17.15, 7.88) **Feeling something stuck in throat bothersome score:** ‐0.751 (‐1.135,‐0.368), 0.238 (‐0.621, 0.145), ‐0.510 (‐0.982, ‐0.038) **Proportion of days with feeling of something stuck in throat:** ‐21.56 (‐33.73,‐9.40), ‐12.11 (‐24.29, 0.06), ‐17.44 (‐32.34, ‐2.54) **Pain with drinking:** ‐0.005 (‐0.363, 0.354), 0.085 (‐0.194, 0.364), ‐0.166 (‐0.483, 0.151) **Difficulty with drinking:** 0.008 (‐0.193, 0.208), 0.046 (‐0.110, 0.202), ‐0.152 (‐0.329, 0.026) **Pain with eating solid food:** ‐0.493 (‐0.819,‐0.167), ‐0.123 (‐0.375, 0.130), ‐0.137 (‐0.444, 0.170) **Difficulty with eating solid food:** ‐0.474 (‐0.794,‐0.153), ‐0.174 (‐0.422, 0.074), ‐0.119 (‐0.418, 0.180) **Proportion of days with vomiting:** ‐2.40 (‐6.58, 1.77), ‐3.54 (‐6.74,‐0.34), ‐4.56 (‐8.32, ‐0.80) **Vomiting frequency:** ‐0.048 (‐0.296, 0.199), 0.04 (‐0.149, 0.229), ‐0.060 (‐0.281, 0.161)
Bhardwaj 2017	Daily diary card The frequency of dysphagia ± heartburn Bhardwaj 2017 Not validated	Dichotomous: Improved yes/no	Dichotomous improvement, first 8 weeks only (used in clinical dichotomous analysis): Budesonide: 3/9 Placebo: 1/9
Butz 2014	EoE symptom score Pentiuk 2009 Not validated	Continuous Score asks participants about the frequency and the severity of their symptoms The threshold for success is not clearly defined	Available as a figure, not possible to digitize. Data not used.
Clayton 2014	Dysphagia symptom score (DSQ) Clayton 2014 Not validated	Continuous Score as follows: 0 = no dysphagia; 1 = solid food dysphagia monthly; 2 = solid food dysphagia < weekly; 3 = solid food dysphagia > weekly, < daily; 4 = solid food dysphagia daily; 5 = solid food dysphagia with every meal; and 6 = dysphagia for solid and liquid food The threshold for success is defined as a mean difference that is significant on a statistical test reported as the mean difference and the corresponding P value.	Change in DSQ score from baseline, mean (SD) at end of trial (used in clinical continuous analysis): Omalizumab: ‐1.2 (1.22)/16 Placebo: ‐1.7 (0.78)/14 Dysphagia score before treatment means (SD): Omalizumab: 4.0 (0.7)/16 Placebo: 5.5 (0.5)/14 Dysphagia score after treatment means (SD): Omalizumab: 2.8 (1.39)/16 Placebo: 3.8 (0.84)/14
Dellon 2012	Mayo Dysphagia Questionnaire (MDQ) Score ‐ 30 day McElhiney 2009 Validated	Continuous The authors compared mean MDQ pre‐ and post‐treatment	MDQ score at end of trial, mean (SD) (used for clinical continuous analysis): Nebulized budesonide solution: 10 (12)/11 Oral viscous budesonide: 16 (17)/11
Dellon 2017	Dysphagia symptom questionnaire (DSQ) Hudgens 2017 Validated	Continuous The authors compared mean DSQ pre‐ and post‐treatment	Change in score at end of trial, means (SD) (used for clinical continuous outcomes): Budesonide oral suspension: ‐14.3 (13)/49 Placebo: ‐7.5 (10.7)/38
Dellon 2019	Dysphagia symptom questionnaire (DSQ) Hudgens 2017 Validated EEsAI‐PRO Schoepfer 2014 Validated	Continuous	DSQ score at end of trial, mean (SD) (used for clinical continuous analysis): Budesonide: 4.8 (7.3)/46 Fluticasone: 4.2 (7.5)/38 EEsAI post‐treatment, mean (SD): Budesonide: 22.1 (18.9)/32 Fluticasone: 28.0 (20.4)/38
Dellon 2021b	Dysphagia symptom questionnaire (DSQ) Hudgens 2017 Validated	Dichotomous and continuous Percentage of group that did not relapse	In remission at end of trial (used for clinical dichotomous analysis): Budesonide oral suspension: 19/25 Placebo: 13/23 Digitized from Figure 3A, change in DSQ at end of trial, mean (SD) (used for clinical continuous analysis): Budesonide oral suspension: ‐1.50 (‐10.70)/24 Placebo: ‐0.11 (‐12.06)/21 Symptom relapse at end of trial: Budesonide oral suspension: 6/25 Placebo: 10/23
Dellon 2022	Dysphagia symptom questionnaire (DSQ) Hudgens 2017 Validated	Continuous	Change in DSQ score at end of trial, LS mean (SD) (used for clinical continuous analysis): Dupilumab: −21.92 (13.39) n = 28/11 imputed Placebo: ‐9.60 (17.20) n = 38/4 imputed DSQ score at end of trial, LS mean (SE): Dupilumab: ‐21.92 (2.53) n = 28/11 imputed Placebo: ‐9.60 (2.79) n = 38/4 imputed
Dellon 2022a	Dysphagia symptom questionnaire (DSQ) Hudgens 2017 Validated EEsAI‐PRO Schoepfer 2014 Validated	Continuous	No SDs reported. Can not use data. DSQ at end of trial: APT‐1011 3 mg twice‐daily: 5.6/20 APT‐1011 3 mg at bedtime: 3.6/21 APT‐1011 1.5 mg twice‐daily: 11.8/22 APT‐1011 1.5 mg at bedtime: 3.8/21 Placebo: 9.1/16
Dellon 2022b	Dysphagia symptom questionnaire (DSQ) Hudgens 2017 Validated	Continuous Mean absolute change in DSQ at weeks 23 to 24 (no SD reported)	No SDs reported. Cannot use data. DSQ score, mean change at end of trial, mean, no SD Lirentelimab 3 mg/kg: –17.4 Lirentelimab 1 mg/kg: –11.9 Placebo: –14.6
De Rooij 2022	Straumann Dysphagia Instrument (SDI) Straumann 2010 Not validated	Continuous Change from baseline to week 6	Change in total SDI score from baseline to week 6, median [IQR] (SD). FFED + AAF: −2 [−4, −2] (2.34)/21 FFED: ‐2.5 [−4.25, −1] (3.70)/20
Dohil 2010	Symptom scoring tool Dohil 2010 Not validated	Continuous Pre‐ and post‐ scores Heartburn/regurgitation; abdominal pain; nausea/vomiting; anorexia/early satiety; dysphagia symptom induced nocturnal wakening; gastrointestinal bleeding	Symptom scoring tool at end of trial, mean (SD) (used for clinical continuous analysis): Oral viscous budesonide + PPI: 1.2 (1.87)/21 Placebo + PPI: 1.85 (1.8)/11
Gupta 2015	EoE Clinical Symptom Score Dohil 2010 Not validated	Dichotomous Based on a physician’s assessment of the frequency and disruptiveness of multiple symptoms within 6 categories (heartburn; abdominal pain; nocturnal awakening with symptoms; nausea, regurgitation, or vomiting; anorexia or early satiety; and dysphagia, odynophagia, or food impaction) and the use and disruptiveness of coping behaviors, determined by questioning of the subject and/or caregiver	Dichotomous symptom response (Figure 3) (used for clinical dichotomous analysis): Budesonide, low‐dose: 11/21 (64.7%) Budesonide, medium‐dose: 15/19 (78.9%) Budesonide, high‐dose: 9/20 (52.9%) All treatment groups: 35/60 (58.3%) Placebo: 14/21 (77.8%)
Heine 2019	Clinical symptom score Not validated	Not reported	Not reported
Hirano 2019	Daily Dysphagia Symptom Diary (DSD) scores Hirano 2019 Not validated Eosinophilic Esophagitis Activity Index (EEsAI) Schoepfer 2014 Validated Patient's global impression of EoE symptoms Hirano 2019	DSD: continuous, mean change EEsAI: continuous, mean change Patient's global impression of EoE symptoms: dichotomous	DSD, digitized from Figure 3D, week 16, mean reduction in the DSD composite score (SD) (used for clinical continuous analysis): RPC4046: ‐13.31 (15.26)/34 Placebo: ‐6.41 (15.40)/34 Patient's global impression of EoE symptoms, dichotomous, from Figure 4C (used for clinical dichotomous analysis): RPC4046: 0.444 (31) + 0.222 (31) + 0.645 (34) + 0.194 (34)/66 = 14 + 7 + 22 + 7 = 50/66 Placebo: 0.364 (34) + 0.212 (34)/34 = 12 + 7 = 19/34
Hirano 2020	Straumann Dysphagia Instrument (SDI) Straumann 2010 No validated Eosinophilic Esophagitis Activity Index (EEsAI) scores Schoepfer 2014	Continuous (primary outcome was the change in value of the PRO score at week 10. A secondary outcome was the change as a percentage) Dichotomous (one of the secondary outcomes was a PRO score change of equal to or greater than 3)	SDI PRO mean change Mean (SE) (used for clinical continuous analysis): Dupilumab: ‐3.2 (0.61)/17 SD: 2.52 Placebo: ‐1.1 (0.67)/14 SD: 2.51 EEsAI PRO ≥ 40% improvement from baseline (used for clinical dichotomous analysis): Dupilumab: 6/23 Placebo: 2/24
Hirano 2020f	EEsAI was adapted for use in this trial Schoepfer 2014 Not validated in adapted form Patient eosinophilic esophagitis global assessment Schoepfer 2014 Validated Physician eosinophilic esophagitis Global Assessment Schoepfer 2014 Validated Mayo Dysphagia Questionnaire‐30 McElhiney 2009 Validated Gastrointestinal Symptom Rating Scale Validated	Continuous	All clinical response data are for post hoc analyses only, cannot use
Hirano 2021	Dysphagia Symptom Questionnaire (DSQ) Hudgens 2017	Continuous Dichotomous ≥ 30% reduction in DSQ score from baseline to week 12	DSQ at end of trial, mean (SD) (used for clinical continuous analysis): Budesonide oral suspension: 19.5 (17.0)/198 Placebo: 22.6 (17.5)/89 Dichotomous (used for clinical dichotomous analysis): Budesonide oral suspension: 112/213 Placebo: 41/105
Kliewer 2019	PEESS V2.0 Franciosi 2011 Validated	Continuous	PEESS at end of trial, mean (SD) (used for clinical continuous analysis): 1‐FED: 23.5 (18.3)/33 4‐FED: 16.0 (13.0)/17
Kliewer 2021	EoE Symptom Activity Index (EEsAI) Schoepfer 2014 Validated	Continuous	Change in EEsAI at end of trial, mean (SD) (used for clinical continuous analysis): 1‐FED: ‐3.0 (16.9)/67 6‐FED: ‐8.2 (17.0)/62
Konikoff 2006	Clinical symptom assessment Konikoff 2006 Not validated	Dichotomous	No prespecified aggregate value reported, cannot use data. FP improves vomiting. The most common clinical symptoms at the start of the study were abdominal pain (reported in 16/28 patients for whom symptom information was available (57%)), vomiting (15/28 (54%)), and dysphagia (13/29 (45%)). Only vomiting improved significantly with treatment with FP (67% pretreatment vs 27% post‐treatment). All patients who responded histologically had a concurrent resolution of their vomiting (6/6), while vomiting in FP non‐responders did not resolve (0/4).
Lieberman 2018	PEESS V1.0 Pentiuk 2009 Not validated	Continuous: PEESS	Digitized from Figure 3 mean (SD) at end of trial (used for clinical continuous analysis): Cromolyn sodium: 17.5 (19.2)/8 Placebo: 22.2 (12.8)/6
Lucendo 2019	NRS for dysphagia, odynophagia Lucendo 2019 NRS for PatGA and PGA EEsAI‐PRO Schoepfer 2014 Validated Dysphagia‐free days	Dichotomous Clinical remission (symptoms severity of 2 points on each 0 to 10 NRS for dysphagia and odynophagia, respectively on each day in the week before end of trial)	Rate of patients with clinical remission (as defined in the primary end point) at end of trial (used as clinical dichotomous outcome): Budesonide: 35/59 Placebo: 4/29
Miehlke 2016	Straumann Dysphagia Instrument (SDI) Straumann 2010 Not validated	**Ordinal** (0 to 9) **Dichotomous** Clinical response defined as a decrease in the dysphagia score of at least 3 points compared with baseline Frequency of dysphagia ranging from none (0) to several times per day (4) and intensity of dysphagia ranging from unhindered swallowing (0) to long‐lasting complete obstruction requiring endoscopic intervention (5). Total scores ranged from 0 to 9.	Decrease in mean dysphagia score from baseline at end of trial. Digitized from supplementary Figure 2, mean dysphagia score (SD) (used in clinical continuous analysis): Budesonide: ‐2.34 (2.66)/53 Placebo: ‐1.99 (2.85)/17
Moawad 2013	Mayo Dysphagia Questionnaire (MDQ) ‐ 2 weeks Peloquin 2006	Continuous	MDQ at end of trial, mean (SD) (used for clinical continuous analysis): Esomeprazole Pos: 1.4 (4.5)/21 Fluticasone Pos: 12 (16)/19
Oliva 2018	Not reported	Not reported	Not reported
Peterson 2010	Dysphagia scale DiSario 2002 Not validated Revalidated reflux disease questionnaire (RDQ) Aanen 2006 Not validated	Continuous The dysphagia scale ranged from 0 to 7 A score of 0 = no dysphagia; 1 = solid food dysphagia once in 3 to 12 months; 2 = solid food dysphagia once in 1 to 3 months; 3 = solid food dysphagia once every 2 to 4 weeks; 4 = solid food dysphagia once every 1 to 2 weeks; 5 = solid food dysphagia once every 1 to 7 days; 6 = solid food dysphagia with every meal; 7 = dysphagia to solid and liquid food No details were provided for the revalidated reflux disease questionnaire (RDQ) apart from a pre‐treatment score No threshold of success provided for both score, but a percentage of change is noted	Dysphagia score at end of trial, mean (SD) (used for clinical continuous analysis): Esomeprazole: 2.3 (2.0)/11 Fluticasone: 1.7 (1.6)/12
Rothenberg 2015	Mayo Dysphagia Questionnaire (MDQ) ‐ 2 weeks Peloquin 2006	Dichotomous Sum change of Mayo Dysphagia Questionnaire items 1, 2, 4, 9, 10, 13, 14, 16, 20, 21. A positive sum change was scored as an improvement, a negative sum change was scored as a worsening, and a zero‐sum change was scored as unchanged. Continuous MDQ score digitized from supplement article Figure E1	Sign of change from baseline in MDQ score, positive vs no change and negative at end of trial (used for clinical dichotomous analysis): Treatment: 10/17 Placebo: 4/8 Data extracted from supplementary Figure E1, MDQ score, mean (SD) at end of trial (used for clinical continuous analysis): Treatment: 1.933333 (3.494213)/15 Placebo: 0.285714 (3.638419)/7
Rothenberg 2022	Dysphagia Symptom Questionnaire (DSQ) Hudgens 2017	Continuous	Least squares mean absolute changes in DSQ score at end of trial, mean (SE) (used for clinical continuous analysis): Dupilumab: ‐23.78 (1.86), n not reported 80?, SD (16.64) Placebo: ‐13.86(1.91), n not reported 79?, SD (16.98)
Schaefer 2008	Resolution of the presenting symptom(s) including, abdominal pain, dysphagia, epigastric pain, foreign body, feeding problems, heartburn, regurgitation, vomiting, and weight loss. Schaefer 2008 Not validated A daily symptom diary was maintained by the patient/guardian while on corticosteroid therapy. Clinical assessment was performed at weeks 4, 12, 18, and 24 to monitor for the presence or absence of the presenting esophageal symptom(s). Schaefer 2008 Not validated	Dichotomous Proportion of symptom‐free patients at follow‐up (4 weeks). Kaplan–Meier analysis was performed including all 80 patients based on intention‐to‐treat analysis. A log‐rank test was used to compare survival curves between treatments.	Proportion of symptom‐free patients at end of trial (used for clinical dichotomous analysis): Prednisone: 32/40 Fluticasone: 35/40
Spergel 2012	Physician’s EoE global assessment Schoepfer 2014 Validated Patient’s predominant EoE symptom Schoepfer 2014 Validated	Continuous and dichotomous Taking into account physical findings, vital signs, the patient’s predominant eosinophilic esophagitis symptom assessment, the patient’s symptom diary, and dietary questions, physicians answered the following question: "In the review of the subject’s symptoms and the physical assessment, what is your global assessment of the subject’s eosinophilic esophagitis?" Physicians answered "none", "mild", "moderate", "severe", or "very severe". **Patient’s predominant EoE symptom** Made up of dysphagia, abdominal/chest pain, vomiting/regurgitation	**Physician's EoE global assessment** Mean shift score from baseline to end of therapy. No SD reported, cannot use. Reslizumab (1 mg) = –0.85 Reslizumab (2 mg) = –1.02 Reslizumab (3 mg) = –1.12 Placebo = –1.14 **Patient’s predominant EoE symptom** Mean shift score from baseline to end of therapy. No SD reported, cannot use. Reslizumab (1 mg) = –0.94 Reslizumab (2 mg) = –1.20 Reslizumab (3 mg) = –1.28 Placebo = –1.44 Dichotomous: Physician's Global Assessment at end of trial (used for clinical dichotomous analysis): Reslizumab (1 mg): 31/56 Reslizumab (2 mg): 32/57 Reslizumab (3 mg): 37/57 Reslizumab: 100/170 Placebo: 37/57
Spergel 2020	PEESS V2.0 Franciosi 2011 Validated	Continuous Total score is reported with a range of 0 to 9. A lower score is better.	Change from baseline in PEESS V2.0, mean (SD) at end of trial (used for clinical continuous analysis): Viaskin milk: 0.71 (1.11)/7 Placebo: 2.00 (1.41)/2
Straumann 2010a	Esophagus‐related symptom score Straumann 2003 Not validated Dysphagia days Straumann 2010a Not validated	Dichotomous Clinical improvement in eosinophilic esophagitis Continuous, percent dysphagia days	Improvement at end of trial (used for clinical dichotomous analysis): Mepolizumab: 3/5 Placebo: 3/6 Digitized from Figure 3B percent dysphagia days at end of trial, mean (SD) (used for clinical continuous analysis): Mepolizumab: 71.91 (17.34)/5 Placebo: 55.14 (20.83)/6
Straumann 2010b	Clinical symptoms were assessed by frequency and intensity of dysphagia events without use of a validated PRO Straumann 2010	**Dichotomous** Clinical response **Continuous** Post‐scores (mean ± SD) The following non‐validated scores were used to assess dysphagia. Frequency of dysphagia events; none = 0; once per week = 1; several times per week = 2; once per day = 3; and several times per day = 4. Intensity of dysphagia events: swallowing unhindered = 0; slight sensation of resistance = 1; slight retching with delayed passage = 2; short period of obstruction necessitating intervention = 3; longer‐lasting period obstruction only removable by vomiting = 4; and long‐lasting complete obstruction requiring endoscopic intervention = 5 A clinical response was defined as a decrease in the dysphagia score of at least 3 points compared with baseline	Clinical response (used for clinical dichotomous analysis): Budesonide: 13/18 Placebo: 4/18 Symptom scores at end of trial, mean (SD) (used for clinical continuous analysis: Budesonide: 2.22 (2.02)/18 Placebo: 4.72 (1.96)/18
Straumann 2011	Dysphagia symptom score Straumann 2010 Not validated	Continuous, pre‐ and post‐scores	Symptom score at end of trial, mean (SD) (used for clinical continuous analysis): Budesonide: 0.79 (1.37)/14 Placebo: 0.71 (1.20)/14
Straumann 2013	Dysphagia was assessed using a visual dysphagia questionnaire (VDQ) Straumann 2013 Not validated Chest pain was assessed using a "pain questionnaire" Straumann 2010 Not validated Combined the VDQ and pain questionnaire for a "total score" Physician’s global assessment Validated	Continuous (compared post‐treatment means); no pre‐specified response threshold	Total PRO score at end of trial, mean (SD) (used for clinical continuous analysis): OC000459: 10.79 (6.52)/14 Placebo: 9.73 (8.16)/12
Straumann 2020	Dysphagia assessed via numerical rating scale (1 to 10) Straumann 2020 Not validated Odynophagia assessed via numerical rating scale (1 to 10) Straumann 2020 Not validated EEsAI Schoepfer 2014 Validated	Dichotomous for dysphagia and odynophagia (clinical remission defined as a severity of ≤ 2 points on 1‐ to 10‐point numerical rating scale (NRS) for dysphagia and a severity of ≤ 2 points on a 0‐ to 10‐point NRS for odynophagia on each day in the last week of induction treatment) EEsAI‐PRO score also dichotomous ≤ 20 at end of treatment	Weekly EEsAI‐PRO score of ≤ 20 at end of trial (used for clinical dichotomous analysis): Budesonide 0.5 mg twice‐daily: 49/68 Budesonide 1.0 mg twice‐daily: 50/68 Budesonide: 99/136 Placebo: 14/68 EEsAI‐PRO at end of trial, mean (SD) (used for clinical continuous analysis): Budesonide 0.5 mg: 14 (18.5)/65 Budesonide 1.0 mg: 11 (18.0)/66 Budesonide: 12.49 (18.10)/131 Placebo: 39 (21.4)/65
Tytor 2021	Watson Dysphagia Score (WDS) Dakkak 1992 Not validated	Continuous, difference in Watson Dysphagia Scale score (0 to 45) at 8 weeks from screening	Change in Watson Dysphagia Scale score at end of trial, mean (SD) (used for clinical continuous analysis): Mometasone: ‐6.0 (7.1)/16 Placebo: ‐1.8 (6.7)/17

BOS: budesonide oral suspension; BOT: budesonide orodispersible tablet; CG: control group; CI: confidence interval; DSD: daily symptom diary; DSQ: Dysphagia Symptom Questionnaire; EEsAI: Eosinophilic Esophagitis Activity Index; EoT: end of treatment; FP: fluticasone propionate; HD: high‐dose; IG: intervention group; LD: low‐dose; LS: least squares; NEB: nebulized/swallowed budesonide solution; NRS: numerical rating scale; OVB: viscous/swallowed budesonide solution; PEESS: Pediatric Eosinophilic Esophagitis Symptom Severity; PRO: patient‐reported outcome; SE: standard error

**4:** Primary outcome ‐ histological improvement

**Study ID**	**Histological improvement system used**	**Continuous or dichotomous**	Outcome data ‐ histological improvement **at study endpoint**
Alexander 2012	Eosinophils were counted using a 40x objective, a field diameter of 0.625 mm, and a field area of 0.307 mm^2^. The peak eosinophil count per high‐powered field was reported. From the area of the greatest density under low‐powered review, 5 random fields were chosen. Peak eosinophil counts from these 5 fields were used to calculate a mean eosinophil count.	Continuous A complete histologic response was defined as a decrease in the mean eosinophil level of more than 90% from the pretreatment value. A partial response was defined as a decrease in more than 50% from the pretreatment value.	Partial or complete at end of trial (used for histological dichotomous analysis): Fluticasone: 17/21 Placebo: 1/21 Complete (≤ 2) at end of trial: Fluticasone: 13/21 Placebo: 0/21
Alexander 2017	No histological scoring system used	Not reported	Not reported
Assa'ad 2011	Peak eosinophils count	Dichotomous Measuring number of patients with mean peak eos ≤ 5 (complete responders)	Used for histological dichotomous analysis at end of trial (< 15): Mepolizumab 0.55 mg/kg: 4/19 Mepolizumab 2.5 mg/kg: 9/20 Mepolizumab 10 mg/kg: 5/20 Mepolizumab combined: 18/59
Bhardwaj 2017	Eosinophils/hpf	Continuous Amount of eosinophils in esophageal tissue (cells/hpf) compared to baseline	Eos/hpf at end of trial from Table 1, mean (SD) (used for histological continuous analysis): Beclomethasone: 2 (4) n = 4 Placebo: 22.2 (23.1) n = 5
Butz 2014	Peak eosinophils/hpf	Dichotomous Defined as mean peak eosinophils of ≤ 1 eos/hpf in both distal and proximal	Dichotomous outcome mean peak ≤ 1 at end of trial: Fluticasone: 15/28 Placebo group: 0/14 Dichotomous outcome mean peak ≤ 6 at end of trial: Fluticasone: 17/28 Placebo group: 0/14 Dichotomous outcome mean peak ≤ 14 at end of trial (used for histological dichotomous analysis): Fluticasone: 18/28 Placebo group: 1/14
Clayton 2014	Eosinophils/hpf	Continuous Pre‐ and post‐treatment eosinophils/hpf	Eos/hpf at end of trial, mean (SD) (used for histological continuous analysis): Omalizumab: 39 (15)/16 Placebo: 33 (12)/14
Dellon 2012	Eosinophils/hpf 2 biopsies were procured from the distal esophagus (3 cm above the gastroesophageal junction), 1 from the mid‐esophagus (8 cm above), and 2 from the proximal esophagus (13 cm above). A total of 5 hpf (hpf; hpf size = 0.24 mm^2^) were examined per each of the 5 biopsy specimens from each patient and the maximum eosinophil count (eos/hpf) was defined as the count in the hpf in the area of the highest eosinophil density after review of all 25 hpf in each patient. The mean eosinophil count for each patient was calculated after examination of all 25 hpf.	Continuous Post‐treatment max and mean eos count was reported Dichotomous Histological response (complete, near‐complete, and partial responses) for both groups as well	Post‐treatment peak eosinophil count (SD) at end of trial (used for histological continuous analysis): Budesonide, nebulized: 89 (94)/11 Budesonide, oral viscous: 11 (23)/11 Complete response (mean peak < 1 eos/hpf at end of trial): Budesonide, nebulized: 3/13 Budesonide, oral viscous: 7/12 Near‐complete (mean peak < 7 eos/hpf at end of trial): Budesonide, nebulized: 4/13 Budesonide, oral viscous: 8/12 Partial (mean peak < 15 eos/hpf at end of trial) (used for histological dichotomous analysis): Budesonide, nebulized: 5/13 Budesonide, oral viscous: 8/12
Dellon 2017	Peak ≤ 6 eosinophils/hpf and histological responders	Dichotomous Peak ≤ 6 eos/hpf Continuous Mean difference	Mean peak difference (SD) eosinophils/hpf at end of trial (used for histological continuous analysis): Budesonide: –117.0 (111.6)/49 Placebo: –17.3 (83.8)/38 Mean peak ≤ 6 eos/hpf at end of trial (used for histological dichotomous analysis): Budesonide: n = 19/51 Placebo: n = 1/42
Dellon 2019	Eosinophils/hpf	Continuous and dichotomous Mean value and < 15 eos/hpf	Post‐treatment, eos/hpf, mean peak (SD) at end of trial (used for histological continuous analysis): Budesonide: 14.7 (29.0)/56 Fluticasone: 20.9 (34.3)/55 Mean peak < 15 eos/hpf at end of trial (used for histological dichotomous analysis): Budesonide: 40/65 Fluticasone: 35/64
Dellon 2021b	Eosinophils/hpf	Continuous and dichotomous Percentage that did not relapse	Histologic response across all esophageal regions, peak mean eos/hpf at end of trial: Mean peak ≤ 1 eos/hpf at end of trial: Budesonide: 15/25 Placebo: 0/23 Mean peak ≤ 6 eos/hpf at end of trial: Budesonide: 19/25 Placebo: 1/23 Mean peak < 15 eos/hpf at end of trial (used for histological dichotomous analysis): Budesonide: 19/25 Placebo: 3/23 Digitized from figure 3B (used for histological continuous analysis): Budesonide: 15.2(45.8) / 24 Placebo: 76.8 (50.5) / 21
Dellon 2022	Peak eosinophils/hpf	Dichotomous	Mean peak ≤ 6 eos/hpf at end of trial Dupilumab: 25/42 Placebo: 2/39 Mean peak < 15 eos/hpf at end of trial (used for histological dichotomous analysis): Dupilumab: 27/42 Placebo: 3/39
Dellon 2022a	≤ 6 peak eosinophils/hpf	Dichotomous Histologic response at week 12, defined as the percentage of participants with ≤ 6 peak eos/hpf	≤ 6 mean peak eos/hpf at end of trial (used for histological dichotomous analysis): APT‐1011 3 mg twice‐daily: 16/20 APT‐1011 3 mg at bedtime: 14/21 APT‐1011 1.5 mg twice‐daily: 19/23 APT‐1011 1.5 mg at bedtime: 10/21 APT‐1011: 59/85 Placebo: 0/21
Dellon 2022b	≤6 eosinophils/hpf in peak hpf Peak eosinophils ≤ 1 eosinophils/hpf at week 24	Dichotomous n of patients with eos ≤ 6 at 24 weeks (primary endpoint) n of patients with eos ≤ 1 at 24 weeks	≤ 6 mean peak eos/hpf at end of trial (used for histological dichotomous analysis): Lirentelimab 3 mg/kg: 80/91 Lirentelimab 1 mg/kg: 86/93 Lirentelimab: 166/184 Placebo: 10/92 Mean peak ≤ 1 eos/hpf at end of trial: Lirentelimab 3 mg/kg: 77/91 Lirentelimab 1 mg/kg: 82/93 Lirentelimab: 159/184 Placebo: 4/92
De Rooij 2022	Peak eosinophils count	Continuous Mean of absolute change in peak eosinophil count from baseline to week 6, eos/hpf, mean (SD) Dichotomous Histological remission rates at 6 weeks	Absolute change in peak eos count at end of trial (SD) (used for histological continuous analysis): FFED: −26.2 (39.9)/20 FFED + AAF: −40 (36)/21 Mean peak < 15 eos/hpf at end of trial (used for histological dichotomous analysis): FFED: 5/20 FFED + AAF: 10/21
Dohil 2010	Eosinophils count/hpf	Continuous: peak eos/hpf Dichotomous: < 20 peak eos/hpf	Continuous outcomes, mean peak eos/hpf at end of trial (SD): Budesonide: 4.8 (7.0)/15 Placebo + PPI: 65.6 (43.3)/9 Dichotomous outcomes ≤ 6 mean peak at end of trial, (used for histological dichotomous analysis): Budesonide: 14/21 Placebo: 1/11
Gupta 2015	At least 2 mucosal pinch biopsies were obtained from the proximal, mid, and distal esophagus ‐ all 3 esophageal levels Peak eosinophil count of ≤ 6 eosinophils/hpf	Dichotomous	Peak eosinophil count of mean peak ≤ 6/hpf at end of trial (used for histological dichotomous analysis): Budesonide, 1ow‐dose: 2/21 Budesonide, medium‐dose: 8/19 Budesonide, high‐dose: 13/20 Budesonide: 23/60 Placebo: 0/21
Heine 2019	The peak eosinophil count (eosinophils/hpf)	Median eosinophil counts	Median mucosal eosinophil counts (IQR) at end of trial PPI + four food elimination diet: 2.5 (IQR 0.5 to 19)/27, SD: 24.52 PPI: 12 (IQR 0 to 37)/31, SD: 52.55 <10 eosinophils/HPF at end of trial PPI + four food elimination diet: 22/32 (69%) PPI: 14/32 (44%)
Hirano 2019	Eosinophils per high‐power field (eos/hpf)	Eosinophils/hpf: **c**ontinuous/dichotomous Response = < 15 eos/hpf Complete response = < 6 eos/hpf	Eosinophils/hpf change from baseline at end of trial (SD) (used for histological continuous analysis): RPC4046 180 mg: ‐94.76 (67.27)/28 RPC4046 360 mg: ‐99.90 (79.53)/30 RPC4046: ‐97.42 (72.02)/58 Placebo: ‐4.42 (59.94)/32 Mean peak < 15 eos/hpf at end of trial (used for histological dichotomous analysis): RPC4046 180 mg: 14/32 (44%) RPC4046 360 mg: 15/34 (44%) RPC4046: 29/66 Placebo: 0/34 (0%) Mean peak < 6 eos/hpf at end of trial: RPC4046 180 mg: 7/32 (22%) RPC4046 360 mg: 6/34 (18%) RPC4046: 13/66 Placebo: 0/34 (0%)
Hirano 2020	Intraepithelial eosinophils/hpf	Continuous (mean intraepithelial eos/hpf change) Dichotomous	LS mean change from baseline (SE) at end of trial, eos/HPF (used for histological continuous analysis): Dupilumab: –96.4 (9.44)/23, SD = 45.3 Placebo: –9.7 (9.65)/24, SD = 47.3 Patients with response mean peak < 1 eos/hpf at end of trial: Dupilumab: 3/23 (13.0%) Placebo: 0/24 Patients with response ≤ 6 mean peak eos/hpf at end of trial (used for histological dichotomous analysis): Dupilumab: 15/23 Placebo: 0/24 Patients with response < 15 mean peak eos/HPF at end of trial post hoc: Dupilumab: 19/23 Placebo: 0/24
Hirano 2020f	Esophageal eosinophil counts per hpf in all parts of the esophagus were assessed	Continuous (mean/median)	Mean change in eos/hpf at end of trial, no SD, cannot use: APT‐1011 1 mg: −63.8 APT‐1011 3 mg: −34.0 Placebo: −14.8 < 15 mean peak eos/hpf at end of trial (used for histological dichotomous analysis): APT‐1011 1 mg: 6/8 APT‐1011 3 mg: 5/8 APT‐1011: 11/16 Placebo: 1/8 0 eos/hpf at end of trial: APT‐1011: 10/16 Placebo: 1/8
Hirano 2021	Maximum peak eosinophil count, eos/hpf Proportion of strict histologic responders (≤ 6 eos/hpf across all available esophageal levels (proximal, middle, or distal) Proportion of patients achieving a deep histologic response, < 1 eos/hpf Proportion of patients achieving a histologic response (< 15 eos/hpf)	Continuous (mean) Dichotomous (see previous entry)	Mean peak eos/hpf (SD) at end of trial (used for histological continuous analysis): Budesonide: 21.9 (34.6)/201 Placebo: 69.9 (38.4)/92 Dichotomous mean peak eos/hpf < 15 at end of trial (used for histological dichotomous analysis): Budesonide: 132/215 Placebo: 1/107 Dichotomous outcome mean peak eos/hpf ≤ 6 at end of trial: Budesonide: 113/215 Placebo: 1/107 Dichotomous outcome mean peak eos/hpf ≤ 1 at end of trial: Budesonide: 69/215 Placebo: 0/107
Kliewer 2019	Dichotomous: remission is defined as clinical esophageal peak eosinophil count < 15 eosinophils per high‐power field (eos/hpf) at 12 weeks Complete remission is defined as ≤ 1 peak eos/hpf and partial remission as 2 to 14 peak eos/hpf at 12 weeks	Dichotomous	From NCT02610816 at end of trial mean peak eos/hpf < 15 (used remission for histological dichotomous analysis): 1‐food elimination diet: Remission: 24/38 Partial remission: 8/38 Complete remission: 7/38 4‐food elimination diet: Remission: 7/25 Partial remission: 4/25 Complete remission: 3/25
Kliewer 2021	Peak eos/hpf < 15	Dichotomous and continuous	Dichotomous mean peak eos/hpf < 15 at end of trial (used for histological dichotomous analysis): 1‐food elimination: 23/67 6‐food elimination: 25/62 Continuous, decrease in peak eos/hpf from baseline (SD) at end of trial: 1‐food elimination diet: ‐24.5 (57.6)/67 6‐food elimination diet: ‐17.7 (41.3)/62
Konikoff 2006	Peak eosinophil count eos/hpf	Dichotomous, remission defined as a peak eosinophil count of ≤ 1 eosinophil/hpf. Also reported ≤ 6 eos/hpf.	Mean peak ≤ 1 eos/hpf at end of trial: Fluticasone: 10/21 Placebo: 1/15 Mean peak ≤ 6 eos/hpf at end of trial (used for histological dichotomous analysis): Fluticasone: 11/21 Placebo: 2/15
Lieberman 2018	Change in peak eos/hpf from baseline following 8 weeks of treatment (magnification and field of view not specified; email PI)	Continuous: eos/hpf	Eosinophils/hpf (SD) at end of trial digitized from Figure 2 (used for histological continuous analysis): Cromolyn sodium: 57.3 (44.0)/9 Placebo: 71.4 (52.8)/6
Lucendo 2019	Peak eosinophils, eos/hpf	Dichotomous (rate of patients with histologic remission (i.e. peak eos < 16/mm^2^ hpf; equivalent to < 5 eos/hpf) at week 6)	Mean peak eos < 16/mm^2^ hpf at end of trial (used for histological dichotomous analysis): Budesonide: 55/59 Placebo: 0/29
Miehlke 2016	Mean eos/mm^2^/hpf (On each esophageal biopsy specimen, all levels were surveyed and the eosinophils in the most densely infiltrated area were counted in 5 hpf. Total of biopsies per patient: 6. Total of hpf evaluated 30).	Dichotomous Rate of histological remission defined as mean of < 16 eos/mm^2^/hpf Continuous Change in mean number of eos/mm^2^/hpf from baseline to end of trial	Mean peak of < 16 eos/mm^2^/hpf at end of trial (2 weeks) (used for histological dichotomous analysis): Budesonide effervescent tablet 2 x 1 mg: 19/19 Budesonide effervescent tablet 2 x 2 mg: 17/19 Budesonide oral viscous suspension 2 x 2 mg: 17/19 Budesonide: 53/57 Placebo: 0/19 Change in mean number of eos/mm^2^/hpf at end of trial, no SD reported, cannot use: Budesonide effervescent tablet 2 x 1 mg: ‐227 Budesonide effervescent tablet 2 x 2 mg: ‐287 Budesonide oral viscous suspension 2 x 2 mg: ‐180 Placebo: ‐8
Moawad 2013	Change in peak eosinophils/hpf and eosinophils ≤ 7/hpf	Continuous Peak eos/hpf Dichotomous ≤ 7 eos/hpf	Peak mean eos/hpf (SD) at end of trial (used for histological continuous analysis): Esomeprazole: 30.5 (33.7)/21 Fluticasone: 39.2 (29.4)/21 Mean peak ≤ 7 eos/hpf at end of trial (used for histological dichotomous analysis): Esomeprazole: 7/21 Fluticasone: 4/21
Oliva 2018	Peak eosinophil count/hpf	Dichotomous Histologic response < 15 eos/hpf	Only percentages, no numbers as total number of patients in each group not reported at end of trial: 6‐food elimination diet: 69% Swallowed fluticasone: 67% Swallowed budesonide: 75% Oral viscous budesonide: 85%
Peterson 2010	Eosinophils/hpf	Continuous (note participant counts in Table 1 are misleading for post therapy eos/hpf; see Figure 3 and 4) Partial resolution as ≤ 15 eos/hpf and complete resolution as ≤ 5 eos/HPF	Post‐treatment mean max (SD) at end of trial (used for histological continuous analysis): Esomeprazole: 37.2 (28.6)/12 Fluticasone: 48.1 (42)/13 Mean peak ≤ 15 eos/hpf at end of trial (used for histological dichotomous analysis): Esomeprazole: 6/15 Fluticasone: 4/15 Mean peak ≤ 5 eos/hpf at end of trial: Esomeprazole: 4/15 Fluticasone: 2/15
Rothenberg 2015	Eosinophils/hpf	Continuous Mean peak eos/hpf Dichotomous Responders were defined by a reduction in the peak eosinophil counts per hpf by 75% or more at day 85, compared with the baseline counts	Mean peak eos/hpf reduced by 75% at end of trial (used for histological dichotomous analysis): QAX576: 6/17 Placebo: 1/8 Mean peak eos/hpf (SD) digitized from Figure 2 at end of trial (used for histological continuous analysis): QAX576: 42.7 (52.7)/15 Placebo: 72.5 (40.8)/8
Rothenberg 2022	Eosinophils/hpf	Dichotomous mean peak ≤ 6 eosinophils	Mean peak ≤ 6 eos/hpf at end of trial (used for histological dichotomous analysis): Dupilumab: 47/80 Placebo: 5/79
Schaefer 2008	Basal cell zone thickness as a percentage of the epithelial thickness and the maximum number of eos/hpf	Continuous Points were assigned based on basal cell zone thickness as a percentage of the epithelial thickness, and the maximum number of eos/hpf. Points were summed and the totals were translated into histologic grades (normal, mild, moderate, and severe). Grades were assigned a numeric value for statistical analysis.	Histologic improvement by a grade of 1 or more at end of trial (used for histological dichotomous analysis): Prednisone: 75% (30/40) Fluticasone: 85% (34/40) "Complete" histologic resolution (defined as normal biopsy specimens; at end of trial): Prednisone: 65% (26/40) Fluticasone: 45% (18/40) Continuous, data from extracted Table 4 at end of trial (used for histological continuous analysis): Mean peak eos/hpf at end of trial (SD): Prednisone: 2.13 (7.75)/32 Fluticasone: 6.58 (11.55)/36
Spergel 2012	Peak esophageal eosinophil count and the change from baseline to the end of therapy	Continuous Dichotomous: mean peak < 15 eos/hpf	Results are from supplementary Figure E1 that has been digitized; mean peak < 15 eos/hpf at end of trial (used for histological dichotomous analysis): Reslizumab 1 mg/kg: 33/55 Reslizumab 2 mg/kg: 32/57 Reslizumab 3 mg/kg: 39/57 Reslizumab: 104/169 Placebo: 20/57 Eosinophils/hpf mean (SD) at end of trial (used for histological continuous analysis): Reslizumab 1 mg/kg: 42.1 (46.5)/40 Reslizumab 2 mg/kg: 23.9 (25.0)/38 Reslizumab 3 mg/kg 35.9 (23.3)/45 Reslizumab: 37.0 (23.4)/123 Placebo: 99.6 (62.4)/46
Spergel 2020	Change in maximum esophageal eosinophil count from baseline to end of double‐blind treatment	Continuous No threshold of success was identified but a mean difference of change was calculated	Mean peak eos at end of trial (SD) (used for histological continuous analysis): Viaskin milk: 25.57 (31.19)/7 Placebo: 95.00 (63.64)/2 Change from baseline mean (SD) at end of trial: Viaskin milk: –26.86 (22.53)/15 Placebo: 42.50 (31.82)/5
Straumann 2010a	Peak eosinophil count/hpf (area 0.3072 mm^2^) Mean eos/hpf	Dichotomous ≤ 5 eos/hpf Continuous Percentage of reduction in mean eos/hpf	Mean peak ≤ 5 eos/hpf at end of trial (used for histological dichotomous analysis): Mepolizumab: 0/5 Placebo: 0/6 Mean (SD) eos/hpf end of trial (used for histological continuous analysis): Mepolizumab: 33.83 (22.82)/5 Placebo: 53.99 (24.51)/6
Straumann 2010b	Reduction in the esophageal eosinophil load; also present peak eosinophil counts and categorized by the level of response; eosinophil load defined as mean eosinophil number measured in a total of 40 hpf from 2 x 4 biopsy specimens taken from the proximal and distal esophagus	Continuous (means) Dichotomous (by the degree of remission) At least 4 biopsy specimens were taken endoscopically from the proximal half and from the lower half of the esophagus and additionally from any lesion. In all 8 esophageal biopsy specimens, all levels were surveyed and the eosinophils in the most densely infiltrated area were counted in 5 consecutive hpf (area of field 0.3072 mm^2^).	Mean peak eos/hpf (SD) at end of trial (used for histological continuous analysis): Budesonide: 17.7 (26.7)/18 Placebo: 125.6 (67.6)/18 Budesonide vs placebo ≤ 5: 13/18 vs 2/18 5 to 20: 3/18 vs 0/18 > 20: 2/18 vs 16/18 Mean peak ≤ 20 eos/hpf (used for histological dichotomous analysis): Budesonide: 16/18 Placebo: 2/18
Straumann 2011	Looked at eosinophil load; eos load defined as the mean eosinophil number measured in a total of 40 hpf from 2 x 4 biopsy specimens each, taken from the proximal and distal esophagus; all levels were surveyed and the eos in the most densely infiltrated area were counted in 5 hpf ‐area of microscopic filed = 0.3072 mm^2^	Continuous Dichotomous	Mean peak eos/hpf (SD) at end of trial (used for histological continuous analysis): Budesonide: 29.9 (30.6)/14 Placebo: 51.1 (31.1)/14 ≤ 5 eos: 5/14 vs 0/14 5 to 20 eos: 2/14 vs 4/14 > 20 eos: 7/14 vs 10/14 ≤ 20 (used for histological dichotomous analysis): Budesonide: 7/14 Placebo: 4/14
Straumann 2013	Total of 40 hpf from 2 x 4 biopsies taken from the proximal and distal esophagus In all 8 esophageal biopsies, eosinophils in the most densely infiltrated area were counted in 5 consecutive hpf. Area of microscopic field = 0.3072 mm^2^.	Continuous (compared post‐treatment means); no pre‐specified response threshold	Mean peak eos/hpf at end of trial (SD) (used for histological continuous analysis): OC000459: 73.26 (58.29)/14 Placebo: 99.47 (69.95)/12
Straumann 2020	2 biopsies of each esophageal third Counted eosinophils in the most densely infiltrated area (HPF 0.345 mm^2^)	Dichotomous Histologic relapse (i.e. peak of ≥ 48 eos/mm^2^ hpf (corresponding to ≥ 15 eos/hpf in prior studies) Continuous Comparison of change in peak eos/hpf from baseline to end of trial	Histologic relapse at end of trial: Budesonide 0.5 mg twice‐daily: 9/68 Budesonide 1.0 mg twice‐daily: 7/68 Placebo: 61/68 Mean peak < 15 eos/hpf at end of trial (used for histological dichotomous analysis): Budesonide: 120/136 Placebo: 7/68 Change in mean peak eos/mm^2^ from baseline to end of treatment, mean (SD) (used for histological continuous analysis): Budesonide 0.5 mg twice‐daily: 38 (112.6)/66 Budesonide 1.0 mg twice‐daily: 21 (64)/65 Budesonide: 29.56 (91.11)/131 Placebo: 262 (216.3)/65
Tytor 2021	Not reported	Not reported	Not reported

AAF: amino acid‐based formula; BET: budesonide effervescent tablet; BOS: budesonide oral suspension; BOV: budesonide, oral viscous; CG: control group; ED: elimination diet; eos: eosinophils; FFED: four food elimination diet; hpf: high‐power field; IG: intervention group; IQR: interquartile range; LS: least squares; NEB: nebulized/swallowed budesonide solution; OBS: oral budesonide solution; OVB: viscous/swallowed budesonide solution; PPI: proton pump inhibitor; SD: standard deviation

**5:** Primary outcome ‐ withdrawals due to adverse events

**Study ID**	**Withdrawals due to adverse events**	**Reasons for withdrawals**
Alexander 2012	Fluticasone: 2/21 (9.5%) Placebo: 6/21 (28.6%)	The causes of dropout were travel in 2 patients, scheduling in 3 patients, family issues in 2 patients, and change of mind on study involvement 1 week after initiation in 1 patient
Alexander 2017	Montelukast: 2/20 Placebo: 1/21	The causes of the withdrawals were personal and travel reasons
Assa'ad 2011	Mepolizumab 0.55 mg/kg: 3/19 Mepolizumab 2.5 mg/kg: 1/20 Mepolizumab 10 mg/kg: 2/20	Mepolizumab 0.55 mg/kg: 1 withdrew consent due to an adverse event; 2 withdrew consent; 1 was lost to follow‐up Mepolizumab 2.5 mg/kg: 1 withdrew consent due to lack of efficacy Mepolizumab 10 mg/kg: 1 withdrew for other reasons
Bhardwaj 2017	Beclomethasone: 0/9 Placebo: 0/9	No withdrawals reported
Butz 2014	Fluticasone: 5/28 (17.9%) Placebo: 1/14 (7.1%)	Fluticasone: 2 because of prohibited medications, 2 because of loss to follow‐up evaluation, and 1 because of an adverse event (AE) (absence seizure that was deemed unlikely to be related to FP) Placebo: 1 participant from the placebo group was lost to follow‐up
Clayton 2014	Omalizumab: 0/16 Placebo: 0/14	No withdrawals reported
Dellon 2012	Budesonide, nebulized: 0/13 Budesonide, oral viscous: 0/12	No withdrawals reported
Dellon 2017	Budesonide: 2/51 (3.9%) Placebo: 3/42 (7.1%)	Budesonide: 1 because of an adverse event, 1 owing to lack of compliance Placebo: 1 because of lack of efficacy, 1 because of lack of compliance, and 1 owing to pregnancy, 1 additional patient in the placebo arm did not have an evaluable post‐treatment biopsy
Dellon 2019	Budesonide: 9/65 (0%) Fluticasone: 9/64 (0%)	Fluticasone: 1 had an adverse event of a food bolus impaction necessitating an emergency department visit and study withdrawal (from: NCT02019758, SAE was in the fluticasone arm) 1 participant in each group did not receive the intervention after randomization, and 8 in each group were lost to follow‐up and did not undergo the week‐8 endoscopy
Dellon 2021b	Supplementary Figure 1 used for analysis: Budesonide: 3/25 Placebo: 4/23	Budesonide: 2 withdrawal by patient, 1 adverse event Placebo: 4 withdrawal by patient
Dellon 2022	Dupilumab: 0/42 Placebo: 0/39	No withdrawals reported
Dellon 2022a	Supplementary Figure 2: APT‐1011 3 mg twice‐daily (n = 1/20) APT‐1011 3 mg at bedtime (n = 2/21) APT‐1011 1.5 mg twice‐daily (n = 2/22) APT‐1011 1.5 mg at bedtime (n = 4/21) Placebo (n = 5/20) Total: APT‐1011: 9/84 Placebo: 5/20	APT‐1011 3 mg twice‐daily; 1 adverse event APT‐1011 3 mg at bedtime; 1 withdrawal of consent, 1 for other reasons APT‐1011 1.5 mg twice‐daily; 1 withdrawal of consent, 1 adverse event APT‐1011 1.5 mg at bedtime; 3 withdrawal of consent, 1 for other reasons Placebo: 2 withdrawal of consent, 2 adverse events, 1 for other reasons
Dellon 2022b	Lirentelimab 3 mg/kg: 11/91 Lirentelimab 1 mg/kg: 6/93 Lirentelimab: 17/184 Placebo: 4/92	Not reported
De Rooij 2022	Four‐food elimination: 1/20 Four‐food elimination + amino acid formula: 1/21	Four‐food elimination: 1 non‐compliance Four‐food elimination + amino acid formula group: 1 non‐compliant to AAF for personal reasons
Dohil 2010	Budesonide: 7/21 (33%) Placebo: 2/11 (18%)	Budesonide: 2 not wanting to take Splenda, 2 acute asthma requiring systemic corticosteroids, 1 non‐compliance with therapy, 1 transient rash attributed to lansoprazole, 1 lost to follow‐up Placebo: reasons not given
Gupta 2015	From NCT00762073: Budesonide, low‐dose: 4/21 (19.0%) Budesonide, medium‐dose: 2/19 (10.5%) Budesonide, high‐dose: 3/20 (15%) Budesonide: 9/60 (15%) Placebo: 3/21 (14.3%)	Budesonide, low‐dose: 1 lack of efficacy, 1 non‐compliance, 2 withdrawal by patient Budesonide, medium‐dose: 1 adverse event, 1 withdrawal by patient Budesonide, high‐dose: 1 adverse event, 1 non‐compliance, 1 withdrawal by patient Placebo: 1 lack of efficacy, 1 adverse event, 1 withdrawal by patient
Heine 2019	Four food elimination diet + PPI: 5/32 PPI: 1/32	Four‐food elimination diet + PPI: 5 for noncompliance PPI: 1 for non‐compliance
Hirano 2019	From Figure 2: RPC4046 180: 4/31 RPC4046 360: 4/30 RPC4046: 8/61 Placebo: 2/34	RPC4046 180: 3 adverse events, 1 withdrew consent RPC4046 360: 1 adverse event, 1 withdrew consent, 2 for other reasons Placebo: 2 withdrew consent
Hirano 2020	Dupilumab: 1/23 Placebo: 4/24	Dupilumab: 1 adverse event Placebo: 1 protocol non‐compliance, 3 cited as "Other"
Hirano 2020f	APT‐1011 1 mg: 0/8 (0%) APT‐1011 3 mg: 0/8 (0%) APT‐1011: 0/16 Placebo: 2/8 (25%)	Placebo: 1 protocol violation, 1 patient needed excluded medication
Hirano 2021	Budesonide: 11/215 (5.1%) Placebo: 11/107 (10.3%)	Budesonide: 8 withdrawal by patient, 1 adverse event, 1 non‐compliance, 1 physician decision Placebo: 8 withdrawal by patient, 3 adverse events
Kliewer 2019	Four‐food elimination diet: 8/25 (36.0) One‐food elimination diet: 4/38 (15.8)	Four‐food elimination diet: 3 participants withdrew because the diet was too difficult to adhere to, 2 withdrew before initiating the diet, 1 withdrew for insurance reasons, 1 was withdrawn due to an unrelated adverse event requiring a prohibited medication, and 1 was lost to follow‐up One‐food elimination diet: 2 withdrew because the diet was too difficult to adhere to, 2 were lost to follow‐up
Kliewer 2021	From NCT02778867 at end of phase 1: Six‐food elimination: 3/62 One‐food elimination: 2/67	Six‐food elimination: 3 withdrawal by patient; 2 unwilling to continue, 1 non‐compliant One‐food elimination: 2 withdrawal by patient; 1 insurance reasons, 1 unknown
Konikoff 2006	Fluticasone: 1/21 (0%) Placebo: 4/15 (20%)	Fluticasone: 1 did not meet the inclusion criteria for the diagnosis of EoE Placebo: 3 for increased symptoms, 1 for non‐compliance
Lieberman 2018	Cromolyn sodium: 0.0% (0/9) Placebo: 14.3% (1/7)	Placebo: 1 increased GI symptoms within 1 week of beginning study
Lucendo 2019	Budesonide: 8/59 (13.6) Placebo: 3/29 (10.3)	Budesonide: multiple reasons possible including, 3 protocol violations, 4 in/exclusion criteria violated, 1 prohibited concomitant medicine, 1 non‐compliant Placebo: multiple reasons possible including, 1 protocol violations, 1 in/exclusion criteria violated, 1 prohibited concomitant medicine, 3 non‐compliant
Miehlke 2016	Budesonide effervescent tablet 2 x 1 mg: 0/19 (0%) Budesonide effervescent tablet 2 x 2 mg: 2/19 (10.5%) Budesonide oral viscous suspension 2 x 2 mg: 2/19 (10.5%) Budesonide: 4/57 Placebo: 2/19 (10.5%)	Budesonide effervescent tablet 2 x 2 mg: 1 non‐compliant, 1 no post‐therapy biopsy Budesonide oral viscous suspension 2 x 2 mg: 1 insufficient baseline disease, 1 no post‐therapy biopsy Placebo: 2 insufficient baseline disease
Moawad 2013	Fluticasone: 2/21 (14.3%) Esomeprazole: 0/21 (0%)	Fluticasone: 1 with worsening of migraine headaches, which he attributed to FP, 1 with bothersome GERD‐related symptoms and discontinued the steroid, and began treatment with a PPI
Oliva 2018	Withdrawals due to adverse events were not reported	Not reported
Peterson 2010	Fluticasone: 1/15 Esomeprazole: 3/15	Reasons for dropout were unwillingness to perform the second EGD in 3 patients. One patient was withdrawn by the IRB for an interpretation of "inadequate pathology". All dropouts occurred prior to completion of the second dysphagia questionnaire. Two patients who completed the second EGD did not complete the second dysphagia questionnaire.
Rothenberg 2015	QAX576: 2/17 Placebo: 0/8	QAX576: 1 because of a positive drug screen, 1 because of a non–drug‐related serious adverse event
Rothenberg 2022	None reported	None reported
Schaefer 2008	Prednisone: 8/40 Fluticasone: 4/40	Prednisone: non‐compliance with medication, n = 4; patient/family decision, n = 2; lost to follow‐up evaluation, n = 1; adverse effect, n = 1 (increased appetite and abdominal pain) Fluticasone: non‐compliance with medication, n = 2; patient/family decision, n = 2
Spergel 2012	Reslizumab 1 mg/ml: 8/56 Reslizumab 2 mg/ml: 11/57 Reslizumab 3 mg/ml: 7/57 Reslizumab: 26/170 Placebo: 6/57	Reslizumab 1 mg/mL: 1 adverse event, 5 lack of efficacy, 1 lost to follow‐up, 1 other Reslizumab 2 mg/mL: 9 lost to follow‐up, 2 for other reasons Reslizumab 3 mg/mL: 6 lack of efficacy, 1 other Placebo: 4 lack of efficacy, 1 lost to follow‐up, 1 other
Spergel 2020	Viaskin milk: 1/15 (0%) Placebo: 0/5 (0%)	Viaskin milk: withdrawal of consent
Straumann 2010a	Mepolizumab: 0/5 Placebo: 0/6	No withdrawals reported
Straumann 2010b	Budesonide: 0/18 Placebo: 0/18	No withdrawals reported
Straumann 2011	Budesonide: 5/14 Placebo: 9/14	All withdrawals were due to clinical relapse
Straumann 2013	OC000456: 0/14 Placebo: 0/12	No withdrawals reported
Straumann 2020	Budesonide 0.5: 9/68 Budesonide 1.0: 9/68 Budesonide: 18/136 Placebo: 45/68	Budesonide 0.5: 7 due to lack of efficacy, 2 due to lack of co‐operation Budesonide 1.0: 5 due to lack of efficacy, 2 due to adverse event (retinitis and allergic dermatitis), 2 due to lack of co‐operation Placebo: 42 due to lack of efficacy, 3 due to lack of patient's co‐operation
Tytor 2021	Mometasone: 1/17 (5.9%) Placebo: 2/19 (10.5%)	Mometasone: 1 lost to follow‐up Placebo: 2 lost to follow‐up

AAF: amino acid‐based formula; BOS: budesonide oral suspension; BOT: budesonide orodispersible tablet; CG: control group; EGD: esophagogastroduodenoscopy; EoE: eosinophilic esophagitis; FFED: four food elimination diet; FP: fluticasone propionate; GERD: gastroesophageal reflux disease; GI: gastrointestinal; IG: intervention group; IRB: institutional review board; N/A: not applicable; NEB: nebulized/swallowed budesonide solution; OVB: viscous/swallowed budesonide solution; SAE: serious adverse event; TEAE: treatment‐emergent adverse event

**6:** Secondary outcomes

**Study ID**	**Patients with serious adverse events**	**Patients with total adverse events**	**Quality of life**
Alexander 2012	From NCT00275561: Fluticasone: 0/21 Placebo: 0/21	From NCT00275561: Fluticasone: 7/21 (36.8%) (sore throat, 2/21 (10.53%); esophageal candidiasis, 5/21 (26.32%); hoarseness, 0/21 (0.00%); 24‐hour urine cortisol, 23.2 ± 2.5 g/24 hours) Placebo: 6/21 (40%) (sore throat, 3/21 (20.00%); esophageal candidiasis, 0/21 (0.00%); hoarseness, 3/21 (20.00%); 24‐hour urine cortisol, 15.5 ± 2.5 µg/24 hours)	Not reported
Alexander 2017	Montelukast: 0/20 Placebo: 0/21	Montelukast: 0/20 Placebo: 0/21	Not reported
Assa'ad 2011	From NCT00358449: Mepolizumab 0.55 mg/kg: 0/19 Mepolizumab 2.5 mg/kg: 1/20; 1 x foreign body trauma Mepolizumab 10 mg/kg: 2/20; 1 x chest discomfort, 1 x esophageal injury	From NCT00358449: Mepolizumab 0.55 mg/kg: 18/19 Mepolizumab 2.5 mg/kg: 14/20 Mepolizumab 10 mg/kg: 18/20 Most common: vomiting (16.9%), diarrhea (13.6%), and upper abdominal pain (10.2%)	Not reported
Bhardwaj 2017	n = 0 "No significant adverse effects were reported with the study drug"	Not reported	Not reported
Butz 2014	From NCT00426283: Fluticasone: 0/28 Placebo: 0/14 n = 1 SAE: absence seizure that was deemed unlikely to be related to fluticasone group	From NCT00426283: Fluticasone: 19/28 Placebo: 9/14 Fluticasone (n = 28): Eye disorders 0/14 (0.0%); gastrointestinal disorders 7/14 (25.0%); chest pain 1/14 (3.6%); immune system disorders 2/14 (7.1%); infections and infestations 3/14 (10.7%); injury, poisoning, and procedural complications: scrapes and cuts 1/14 (3.6%); investigations: abnormal laboratory values 5/14 (17.9%); nervous system disorders 4/14 (14.3%); respiratory, thoracic, and mediastinal disorders 3/14 (10.7%); skin and subcutaneous tissue disorders 0/14 (0.0%) Placebo (n = 14): Eye disorders 2/14; gastrointestinal disorders 2/14 (14.3%); chest pain 1/14 (7.1%); immune system disorders 0/14 (0.0%); infections and infestations 3/14 (21.4%); injury, poisoning, and procedural complications: scrapes and cuts 0/14 (0.0%); investigations: abnormal laboratory values 1/14 (7.1%); nervous system disorders 0 (0.0%); respiratory, thoracic, and mediastinal disorders 3/14 (21.4%); skin and subcutaneous tissue disorders 1/14 (7.1%)	Not reported
Clayton 2014	From NCT00123630: Omalizumab: 0/16 Placebo: 0/14	From NCT00123630: Omalizumab: 0/16 Placebo: 0/14	Not reported
Dellon 2012	From NCT00961233: Budesonide, nebulized: 0/13 Budesonide, oral viscous: 0/12	From NCT00961233: Budesonide, nebulized: 0/13 Budesonide, oral viscous: 0/12 Used for analysis. Budesonide, nebulized: 1/11 ‐ candidal esophagitis, 1/11 epistaxis Budesonide, oral viscous: 2/11 candidal esophagitis	Not reported
Dellon 2017	From NCT01642212: Budesonide: 1/51 Placebo: 0/42 n = 1; budesonide group Food poisoning	From NCT01642212: Budesonide: 20/51 Placebo: 21/42 Reported as: budesonide; placebo All TEAEs: 24/51; 21/42 TEAEs related to study drug: 5/51; 4/42 Severe TEAE: 1/51; 0/42 Serious adverse events: 1/51; 0/42 TEAEs leading to withdrawal from study: 1/51; 0/42 TEAEs related to study drug and leading to withdrawal from study: 1/51; 0/42 Infections and infestations: 13/51; 7/42 Nasopharyngitis: 3/51; 4/42 Upper respiratory tract infection: 3/51; 2/42 Sinusitis: 2/51; 1/42 *Clostridium difficile* infection: 1/51; 0/42 Oral candidiasis: 1/51; 0/42 Esophageal candidiasis: 1/51; 0/42 Gastrointestinal disorders: 3/51; 9/42 Diarrhea: 0/51; 1/42 Food poisoning: 2/51; 0/42 Vomiting: 1/51; 1/42 Abdominal pain/discomfort: 0/51; 3/42 Respiratory disorders: 6/51; 3/42 Oropharyngeal pain: 2/51; 2/42 Cough: 1/51; 0/42 Dyspnea: 1/51; 0/42 Allergic rhinitis: 1/51; 0/42 Skin disorders: 3/51; 3/42 Acne: 1/51; 0/42 Contact dermatitis: 1/51; 0/42 Eczema: 0/51; 1/42 General: 3/51; 2/42 Fever: 1/51; 1/42 Fatigue: 1/51; 0/42	Not reported
Dellon 2019	From NCT02019758: Oral viscous budesonide: 0/65 Active fluticasone: 1/64; food impaction	From NCT02019758: Oral viscous budesonide: 10/65 Active fluticasone: 15/64 Adverse event: Esophageal candidiasis: budesonide 8 (12%); fluticasone 10 (16%) Oral candidiasis: budesonide 2 (3%); fluticasone 1 (2%) Food impaction: budesonide 0 (0%); fluticasone 1 (2%) Sore throat: budesonide 0 (0%); fluticasone 2 (3%) Chest pain: budesonide 0 (0%); fluticasone 1 (2%) Pneumonia: budesonide 0 (0%); fluticasone 1 (2%)	Not reported
Dellon 2021b	Any severe TEAE from NCT02736409: Budesonide: 0/25 (0) Placebo: 1/23 (4.3); back pain Any severe TEAE Budesonide: 9 (6.9) Placebo: 3 (4.6)	Any TEAE From NCT02736409: Budesonide: 21/25 (84%) Placebo: 14/23 (60.9%) TEAEs experienced by 2.5% of the total Upper respiratory tract infection Budesonide: 12 (9.2%) Placebo: 2 (3.1%) Nasopharyngitis Budesonide: 7 (5.3%) Placebo: 3 (4.6%) Sinusitis Budesonide: 5 (3.8%) Placebo: 3 (4.6%) Esophageal candidiasis Budesonide: 4 (3.1%) Placebo: 5 (7.7%) Influenza Budesonide: 5 (3.8%) Placebo: 1 (1.5%) Nausea Budesonide: 9 (6.9%) Placebo: 4 (6.2%) Vomiting Budesonide: 13 (9.9%) Placebo: 1 (1.5%) Diarrhea Budesonide: 5 (3.8%) Placebo: 4 (6.2%) Dysphagia Budesonide: 3 (2.3%) Placebo: 1 (1.5%) Gastritis Budesonide: 6 (4.6%) Placebo: 0 (0.0%) Blood cortisol decreased Budesonide: 4 (3.1%) Placebo: 4 (6.2%) Cough Budesonide: 5 (3.8%) Placebo: 2 (3.1%) Fatigue Budesonide: 4 (3.1%) Placebo: 1 (1.5%) Mood swings Budesonide: 2 (1.5%) Placebo 3 (4.6%)	Not reported
Dellon 2022	None reported	Not reported as individuals/group, cannot use Injection‐site reactions: Dupilumab: 7/42(16.7%) Placebo: 14/39 (0.3%) Nasopharyngitis: Dupilumab: 5/42 (11.9%) Placebo: 4/39 (10.3%)	Not reported
Dellon 2022a	APT‐1011 3 mg at bedtime (n = 1/22) (5%) APT‐1011: 1/85 Placebo: 0/21	Total adverse events = 63/85 (74%) APT‐1011 3 mg twice‐daily (17/20) APT‐1011 3 mg at bedtime (16/22) APT‐1011 1.5 mg twice‐daily (17/22) APT‐1011 1.5 mg at bedtime (13/21) Placebo (13/21) APT‐1011 = 63/85 Placebo: 13/21	EoE Adult Quality of Life Questionnaire (EoE‐QoLA) but no data reported
Dellon 2022b	Total n = 3 Lirentelimab high‐dose: 2 Lirentelimab low‐dose: 0 Placebo: 1 Lirentelimab: 2/184 Placebo: 1/92 Type not reported	≥ 1 TEAE Lirentelimab high‐dose: 61/91 Lirentelimab low‐dose: 65/93 Placebo: 53/92 Lirentelimab: 126/184 Placebo: 53/92 Infusion reaction Lirentelimab high‐dose: 35/91 Lirentelimab low‐dose: 24/91 Placebo: 11/92 Headache Lirentelimab high‐dose: 6/91 Lirentelimab low‐dose: 8/93 Placebo: 6/92	Not reported
De Rooij 2022	Four‐food elimination diet + amino acid formula: 0/20 Four‐food elimination diet: 0/21	Four‐food elimination diet + amino acid formula: 1/20; emergency room visit due to severe abdominal pain after eating a kiwi Four‐food elimination diet: 0/21	EoEQoL score Change in total EoE‐QoL score from baseline to week 6, median (IQR) (cannot use) Four‐food elimination diet + amino acid formula: 0.1 (0.04 to 0.56) Four‐food elimination diet: 0 (−0.08 to 0.4)
Dohil 2010	From NCT00638456: Budesonide + PPI: 0/21 PPI: 0/11	From NCT00638456: Budesonide + PPI: 3/21 PPI: 5/11 Budesonide + PPI: 3 1: emesis 1: oral Candida 1: transient headache PPI: 5 1: eczema worse 1: chest infection 1: mild abdominal pain 1: transient headache 1: transient diarrhea	Not reported
Gupta 2015	From NCT00762073: Budesonide: 1/60; diet refusal Placebo: 0/21 G1 ‐ 1 G2 ‐ 0 G3 ‐ 1 G4 ‐ 1	From NCT00762073: Budesonide: 13 + 16 + 17 = 46/60 Placebo: 10/21 Budesonide most frequent adverse events: rash, 10/60 (17%); diarrhea, 10/60 (17%); pyrexia, 10/60 (17%); cough, 9/60 (15%); sinusitis, 9/60 (15%); nasopharyngitis, 8/60 (13%); oropharyngeal pain, 8/60 (13%); headache, 7/60 (12%) Placebo most frequent adverse events: pyrexia, 3/21 (14%); headache, 2/21 (10%); vomiting, 2/21 (10%); asthma, 2/21 (10%)	Not reported
Heine 2019	Not reported	Not reported	Not reported
Hirano 2019	SAE: RPC4046 180 mg: 0/32 (0%) RPC4046 360 mg: 1/34 (2.9%); appendicitis RPC4046: 1/66 Placebo: 2/34 (5.9%); 1 x umbilical hernia, 1 x appendicitis	Total AE: RPC4046 180 mg: 20/32 (63%) RPC4046 360 mg: 29/34 (85%) RPC4046: 49/66 Placebo: 22/34 (65%) Adverse events in placebo, RPC4046 180 mg, RPC4046 360 mg: Headache: 5 (14.7%), 5 (16.1%), 7 (20.6%) Upper respiratory tract infection: 3 (8.8%), 5 (16.1%), 5 (14.7%) Arthralgia: 0, 4 (12.9%), 2 (5.9%) Nasopharyngitis: 0, 3 (9.7%), 3 (8.8%) Diarrhea: 2 (5.9%), 3 (9.7%), 2 (5.9%) Nausea: 4 (11.8%), 2 (6.5%), 3 (8.8%) Abdominal pain: 0, 2 (6.5%), 2 (5.9%) Dizziness: 2 (5.9%), 3 (9.7%), 1 (2.9%) Oropharyngeal pain: 0, 1 (3.2%), 3 (8.8%) Sinusitis: 0, 3 (9.7%), 1 (2.9%) Vomiting: 2 (5.9%), 1 (3.2%), 3 (8.8%) Contact dermatitis: 0, 1 (3.2%), 2 (5.9%) Fatigue: 1 (2.9%), 2 (6.5%), 1 (2.9%) Injection site erythema: 2 (5.9%), 0, 3 (8.8%) Urticaria: 0, 2 (6.5%), 1 (2.9%) Myalgia: 0, 1 (3.2%), 2 (5.9%) Contusion: 1 (2.9%), 2 (6.5%), 0 Cough: 1 (2.9%), 2 (6.5%), 0 Gastroenteritis: 1 (2.9%), 2 (6.5%), 0 Hypersensitivity: 0, 0, 2 (5.9%) Injection site hematoma: 0, 0, 2 (5.9%) Injection site pruritus: 1 (2.9%), 0, 2 (5.9%) Ligament sprain: 1 (2.9%), 0, 2 (5.9%)	Not reported
Hirano 2020	From NCT02379052: Dupilumab: 3/23; 1 x‐food allergy, 1 x blood creatine phosphokinase increased, 1 x abortion spontaneous Placebo: 0/24	From NCT02379052: Dupilumab: 18/23 Placebo: 14/24 Dupilumab, major adverse events: injection site erythema, 8/23 (34.78%); injection site rash, 3/23 (13.04%); injection site urticaria, 2/23 (8.70%); injection site inflammation, 3/23 (13.04%); injection site pain, 2/23 (8.70%); nasopharyngitis, 5/23 (21.74%) Placebo, major adverse events: injection site erythema, 2/24 (8.33%); injection site pain, 2/24 (8.33%); upper respiratory tract infection, 3/24 (12.50%); abdominal pain, 2/24 (8.33%); nausea, 3/24 (12.50%); dizziness, 2/24 (8.33%)	From NCT02379052: LS mean change from baseline (SE) Dupilumab: 0.80 (0.137) 23 SD = (0.66) Placebo: 0.47 (0.141) 24 SD = (0.69) QoL was assessed using Eosinophilic Esophagitis Quality of Life (EoE‐QOL‐A) score, version 3.0 from baseline to week 12 EoE‐QOL‐A total score week 12, n (%): Dupilumab: 23/0 Placebo: 21/3 LS mean change from baseline (SE): Dupilumab: 0.8 (0.1) Placebo: 0.5 (0.1) Difference vs placebo (95% CI): 0.3 (‐0.1 to 0.7) Unclear if 30 items or 24 items
Hirano 2020f	APT‐1011: 0/16 Placebo: 0/8	APT‐1011: 12/16 Placebo: 6/8 Major adverse events, placebo, APT‐1011 1.5 mg, APT‐1011 3.0 mg: blood cortisol decreased: 2 (25%), 3 (37.5%), 1 (12.5%); diarrhea: 0, 0, 2 (25%); nasopharyngitis: 0, 1 (12.5%), 1 (12.5%) A total of 41 TEAEs were reported by 18 participants: 12 participants receiving APT‐1011 reported 26 TEAEs and 6 participants receiving placebo reported 15 TEAEs	Not reported
Hirano 2021	From NCT02605837: Budesonide: 2/215 Placebo: 1/107	From NCT02605837: Budesonide: 63/215 Placebo: 28/107 Any non‐serious TEAE Budesonide: 130 (61.0%) Placebo: 64 (61.0%) Total: 194 (61.0%) Any mild TEAE Budesonide: 69 (32.4%) Placebo: 38 (36.2%) Total: 107 (33.6%) Any moderate TEAE Budesonide: 56 (26.3%) Placebo: 24 (22.9%) Total 80 (25.2%) Any severe TEAE Budesonide: 5 (2.3%) Placebo: 2 (1.9%) Total 7 (2.2%) Any serious TEAE 2 Budesonide: (0.9%) Placebo: 1 (1.0%) Total 3 (0.9%) Any life‐threatening TEAE Budesonide: 0 (0.0%) Placebo: 0 (0.0%) Total 0 (0.0%) TEAE related to study treatment Budesonide: 45 (21.1%) Placebo: 23 (21.9%) Total 68 (21.4%) TEAE related to EoE Budesonide: 11 (5.2%) Placebo: 6 (5.7%) Total 17 (5.3%) TEAE leading to dose discontinuation Budesonide: 3 (1.4%) Placebo: 5 (4.8%) Total 8 (2.5%) TEAE leading to study discontinuation Budesonide: 1 (0.5%) Placebo: 3 (2.9%) Total 4 (1.3%) Infections and infestations Nasopharyngitis Budesonide: 11 (5.2%) Placebo: 4 (3.8%) Total 15 (4.7%) Sinusitis Budesonide: 9 (4.2%) Placebo: 3 (2.9%) Total 12 (3.8%) Esophageal candidiasis Budesonide: 8 (3.8%) Placebo: 2 (1.9%) Total 10 (3.1%) Oral candidiasis Budesonide: 8 (3.8%) Placebo: 0 (0.0%) Total 8 (2.5%) Gastrointestinal disorders Nausea Budesonide: 6 (2.8%) Placebo: 3 (2.9%) Total 9 (2.8%) Vomiting Budesonide: 4 (1.9%) Placebo: 4 (3.8%) Total 8 (2.5%) Investigations ACTH stimulation test abnormal Budesonide: 6 (2.8%) Placebo: 3 (2.9%) Total 9 (2.8%) Respiratory, thoracic, and mediastinal disorders Cough Budesonide: 6 (2.8%) Placebo: 3 (2.9%) Total 9 (2.8%) Skin and subcutaneous tissue disorders Acne Budesonide: 5 (2.3%) Placebo: 3 (2.9%) Total 8 (2.5%) Nervous system disorders Headache Budesonide: 7 (3.3%) Placebo: 1 (1.0%) Total 8 (2.5%)	Not reported
Kliewer 2019	From NCT02610816 One‐food elimination diet: 1/38; abdominal pain Four‐food elimination diet: 1/25; abdominal pain	From NCT02610816 One‐food elimination diet: 5/38 Four‐food elimination diet: 8/25 One‐food elimination diet: abdominal pain, 2/38 (5.26%); vomiting, 1/38 (2.63%); cough, 1/38 (2.63%); nasal congestion, 1/38 (2.63%) Four‐food elimination diet: abdominal pain, 2/25 (8.00%); vomiting, 2/25 (8.00%); cough, 2/25 (8.00%); urticaria, 2/25 (8.00%)	Change from baseline in Pediatric Quality of Life Inventory Version 3.0 EoE Module (PedsQL 3.0 EoE) at 12 weeks One‐food elimination diet: 9.7 (11.3)/31 Four‐food elimination diet: 9.8 (14.1)/16
Kliewer 2021	From NCT02778867: One‐food elimination diet: 0/67 Six‐food elimination diet: 0/62 One‐food elimination: at 6 weeks, 0/67 Six‐food elimination: at 6 weeks, 0/62	From NCT02778867: One‐food elimination diet: 1/67; Diarrhea Six‐food elimination diet: 2/62; Diarrhea	EoEoE‐Qol‐A (24 items), change from baseline: One‐food elimination: at 6 weeks ‐0.9 (10.2)/67 Six‐food elimination: at 6 weeks ‐0.33 (11.8)/62
Konikoff 2006	Fluticasone: 0/21 Placebo: 0/15	Fluticasone: 1/21; esophageal candidiasis Placebo: 0/15	Not reported
Lieberman 2018	Cromolyn: 0/9 Placebo: 0/7	Not reported as individuals/group, cannot use Adverse events reported: Nausea: cromolyn group 55.6% (5/9); placebo group 14.3% (1/7) Abdominal pain: cromolyn group 44.4% (4/9); placebo group 28.6% (2/7) Headache: cromolyn group 44.4% (4/9); placebo group 14.3% (1/7) Vomiting: cromolyn group 22.2% (2/9); placebo group 14.3% (1/7) Upper respiratory tract infection: cromolyn group 22.2% (2/9); placebo group 14.3% (1/7) Fatigue: cromolyn group 22.2% (2/9); placebo group 0.0% (0/9) Sore throat: cromolyn group 22.2% (2/9); placebo group 0.0% (0/9) Lip swelling: cromolyn group 11.1% (1/9); placebo group 0.0% (0/9) Sinus infection: cromolyn group 11.1% (1/9); placebo group 14.3% (1/7) Dysphagia: cromolyn group 11.1% (1/9); placebo group 0.0% (0/9) Diarrhea: cromolyn group 11.1% (1/9); placebo group 0.0% (0/9) Eye pain: cromolyn group 11.1% (1/9); placebo group 0.0% (0/9) Mood change: cromolyn group 11.1% (1/9); placebo group 0.0% (0/9) Hypernatremia: cromolyn group 11.1% (1/9); placebo group 0.0% (0/9)	Not reported
Lucendo 2019	Budesonide: 6/59 (10.1%) Placebo: 13/29 (44.8%) Severe TEAE esophageal food impaction TEAE related to study drug	Any TEAE: Budesonide: 37/59 (62.7%) Placebo: 12/29 (41.1%) TEAEs by occurring in 2 patients in any treatment group, reported as budesonide; placebo: Gastrointestinal disorders: 10/59 (16.9%); 3/29 (10.3%) Gastroesophageal reflux disease: 3/59 (5.1%); 0/29 (0%) Nausea: 2/29 (3.4%); 0/29 (0%) Infections and infestations: 21/59 (35.6%); 6/29 (20.7%) Suspected local fungal infection, thereof: 14/59 (23.7%); 0/29 (0%) Histologically confirmed: 10/59 (16.9%); 0/29 (0%) Histologically confirmed with suspected endoscopic signs: 8/59 (13.6%); 0/29 (0%) Histologically confirmed with suspected endoscopic signs and clinical symptoms: 3/59 (5.1%); 0/29 (0%) Nasopharyngitis: 2/59 (3.4%); 1/29 (3.4%) Pharyngitis: 1/59 (1.7%); 2/29 (6.9%) Investigations: 5/59 (8.5%); 0/29 (0%) Blood cortisol decreased: 3/59 (5.1%); 0/29 (0%) Nervous system disorders: 5/59 (8.5%); 1/29 (3.4%) Headache: 4/59 (6.8%); 1/29 (3.4%) Respiratory, thoracic and mediastinal disorders: 2/59 (3.4%); 2/29 (6.9%) Asthma: 0/59 (0%); 2/29 (6.9%) Vascular disorders: 3/59 (5.1%); 0/29 (0%) Hypertension: 2/59 (3.4%); 0/29 (0%)	EoE‐QoL‐A 30‐items (weighted average) baseline, mean (SD) Budesonide: 2.3 (0.8) Placebo: 2.3 (0.8) EoT, mean (SD): Budesonide: 2.8 (0.9)/59 Placebo: 2.6 (0.7)/29 Change from baseline to EoT, mean (95% CI) Budesonide: 0.5 (0.32 to 0.62) Placebo: 0.2 (0.06 to 0.42) BOT–placebo, mean difference (95% CI) 0.23 (–0.010 to 0.472)
Miehlke 2016	Budesonide: 0/57 Placebo: 0/19	Budesonide: 4 + 5 + 6 = 15/57 Placebo: 0/19 BET 2 x 1 mg: 5/19: 3 esophageal candidiasis, 1 increased WBC count, 1 pruritus BET 2 x 2mg: 6/19: 1 nausea, 1 blistering oral mucosa, 3 esophageal candidiasis, 1 blood cortisol decreased BOV 2 x 2 mg: 6/19: 1 bowel movement irregularity, 1 lip edema, 3 esophageal candidiasis, 1 pruritus	Not reported
Moawad 2013	From NCT00895817: Esomeprazole: 0/21 Swallowed fluticasone: 0/21	From NCT00895817: Esomeprazole: 0/21 Swallowed fluticasone: 0/21 Used for analysis: FP arm n = 3/21 n = 1; 1 patient had worsening of migraine headaches, which he attributed to FP (discontinued) n = 1; GERD‐related symptoms and discontinued the steroid, and began treatment with a PPI (discontinued) n = 1; esophageal candidiasis PPI arm n = 0/21	Not reported
Oliva 2018	Not reported	Not reported	Not reported
Peterson 2010	Esomeprazole: 0/15 Fluticasone: 0/15	Esomeprazole: 0/15 Fluticasone: 0/15	Not reported
Rothenberg 2015	QAX576: 1/17; asymptomatic cyst‐like lesion in the right calf that predated enrollment in the study, but upon subsequent investigations, it turned out to be a spindle cell sarcoma Placebo: 1/8; not reported	QAX576: 10/17 Placebo: 5/8 Adverse events, reported as QAX576; placebo: Cough: 4 (23.5%), 1 (12.5%); nasal congestion: 3 (17.6%), 2 (25.0%); oropharyngeal pain: 3 (17.6%), 2 (25.0%); gastroesophageal reflux disease: 4 (23.5%), 0 (0.0%); headache: 3 (17.6%), 1 (12.5%); nausea: 3 (17.6%), 1 (12.5%); chills: 2 (11.8%), 1 (12.5%); contusion: 2 (11.8%), 1 (12.5%); vomiting: 2 (11.8%), 1 (12.5%); arthralgia: 1 (5.9%), 1 (12.5%); back pain: 1 (5.9%), 1 (12.5%); dermatitis: 2 (11.8%), 0 (0.0%); dizziness: 1 (5.9%), 1 (12.5%); fatigue: 2 (11.8%), 0 (0.0%); ligament sprain: 1 (5.9%), 1 (12.5%); nasal mucosal discoloration: 2 (11.8%), 0 (0.0%); pyrexia: 2 (11.8%), 0 (0.0%); sinus congestion: 2 (11.8%), 0 (0.0%); sinusitis: 1 (5.9%), 1 (12.5%); tonsillolith: 0 (0.0%), 2 (25.0%)	Not reported
Rothenberg 2022	None reported	Not reported as individuals/group, cannot use Injection‐site reactions: Dupilumab: 30/80 (37.5%) Placebo: 26/79 (33.3%) Fever: Dupilumab: 5/80 (6.3%) Placebo: 1/79 (1.3%)	Not assessed
Schaefer 2008	Prednisone: 7.5% (3/40) Systemic adverse effects (hyperphagia, weight gain, and/or cushingoid features) Fluticasone: 0% (0/40)	Systemic adverse effects (hyperphagia, weight gain, and/or cushingoid features) Prednisone: 40% (16/40) Fluticasone: 0% (0/40) Esophageal candidal overgrowth Prednisone: 0% (0/40) Fluticasone: 15% (6/40)	Not reported
Spergel 2012	From NCT00538434: Reslizumab 1 mg/kg: 1/55 Reslizumab 2 mg/kg: 1/57 Reslizumab 3 mg/kg: 1/57 Reslizumab: 3/169 Placebo: 2/57 Abdominal pain 0; 0; 0; 1 Anaphylaxis 0; 0; 0; 1 Gastrointestinal inflammation 1; 0; 0; 0 Respiratory distress 0; 0; 1; 0 Syncope 0; 0; 0; 1 Viral gastroenteritis 0; 1; 0; 0 Patients who discontinued because of an adverse event 1; 0; 0; 0	From NCT00538434: Reslizumab 1 mg/kg: 38/55 Reslizumab 2 mg/kg: 29/57 Reslizumab 3 mg/kg: 39/57 Reslizumab: 106/169 Placebo: 40/57 Headache: 8; 6; 12; 7 Cough: 5; 6; 6; 6 Nasal congestion: 7; 3; 4; 8 Pharyngo‐laryngeal pain: 6; 3; 4; 9 Upper respiratory tract infection: 5; 4; 6; 5 Nausea: 6; 2; 4; 3 Pyrexia: 4; 2; 3; 6 Sinusitis: 5; 1; 5; 4 Upper abdominal pain: 4; 2; 4; 5 Nasopharyngitis: 3; 2; 1; 7 Diarrhea: 4; 3; 0; 5	Child Health Questionnaire (CHQ) Validated Landgraf 2014 **Physical summary score** Mean difference: Arm 1 (1 mg) = ‐4.75 (‐17.35 to 8.03) Arm 2 (2 mg) = ‐1.47 (‐14.17 to 11.23) Arm 3 (3 mg) = 1.36 (‐11.19 to 13.91) **Psychosocial summary score** Mean difference: Arm 1 (1 mg) = ‐6.38 (‐15.01 to 2.24) Arm 2 (2 mg) = ‐3.41 (‐12.05 to 5.23) Arm 3 (3 mg) = ‐0.43 (‐9.00 to 8.15) **Global health summary score** Mean difference: Arm 1 = ‐1.57 (‐16.98 to 13.85) Arm 2 = ‐5.54 (‐20.94 to 9.87) Arm 3 = ‐1.06 (‐16.16 to 14.06) From NCT00538434: no SDs reported, cannot use
Spergel 2020	Viaskin milk: 0/15 Placebo: 1/5; vocal cord dysfunction in a participant with asthma leading to a hospitalization at day 2 of the study	**Total (n** = **20)** Blood and lymphatic system disorders Ear and labyrinth disorders Eye disorders Gastrointestinal disorders General disorders and administration site conditions Infections and Infestations Injury, poisoning, and procedural complications Metabolism and nutrition disorders Musculoskeletal and connective tissue disorders Nervous system disorders Respiratory, thoracic, and mediastinal disorders Skin and subcutaneous tissue disorders **Viaskin milk (n** = **15)** Blood and lymphatic system disorders Ear and labyrinth disorders Eye disorders Gastrointestinal disorders General disorders and administration site conditions Infections and Infestations Injury, poisoning, and procedural complications Metabolism and nutrition disorders Musculoskeletal and connective tissue disorders Nervous system disorders Respiratory, thoracic, and mediastinal disorders Skin and subcutaneous tissue disorders **Placebo (n = 5)** Blood and lymphatic system disorders Ear and labyrinth disorders Eye disorders Gastrointestinal disorders General disorders and administration site conditions Infections and Infestations Injury, poisoning, and procedural complications Metabolism and nutrition disorders Musculoskeletal and connective tissue disorders Nervous system disorders Respiratory, thoracic, and mediastinal disorders Skin and subcutaneous tissue disorders Viaskin milk: 15/15 Placebo: 5/5	PedsQL ‐ Quality of life (validated) Viaskin milk: 24.4 (20.68)/7 Placebo: 38.00 (18.38)/2
Straumann 2010a	Mepolizumab: 0/5 Placebo: 0/6	Mepolizumab: 2/5 Placebo: 2/6 Any adverse event: 2/5 (used) Nausea = 0/5 Esophageal food impaction = 0/5 Vomiting = 0/5 Fatigue = 1/5 Upper respiratory tract infection = 1/5 ANy adverse event: 2/6 (used) Nausea = 1/6 Esophageal food impaction = 1/6 Vomiting = 1/6 Vomiting = 0/6 Upper respiratory tract infection = 0/6	Not reported
Straumann 2010b	Budesonide: 0/18 Placebo: 0/18	Budesonide: 4/18 Placebo: 1/18 Budesonide: 3/18 mild signs of clinically asymptomatic esophageal candidiasis on follow‐up endoscopy 1/18 histologic Candida without endoscopic findings of the same Placebo: 1/18 hoarseness	Not reported
Straumann 2011	Budesonide: 0/14 Placebo: 0/14	Budesonide: 0/14 Placebo: 0/14	Not reported
Straumann 2013	OC000459: 1/14 Placebo: 0/12 1/14 patients with a serous event in the OC000459 arm – acute appendicitis in follow‐up period	Not reported as individuals/group, cannot use 1/14 patients with a serous event in the OC000459 arm – acute appendicitis in follow‐up period 1/12 patients in the placebo arm with an adverse event ‐ dizziness 15 minor events; 6 x OC000459 and 9 x placebo	Not reported
Straumann 2020	Budesonide 0.5 mg: 3/68 Budesonide 1.0 mg: 1/68 Placebo: 0/68 Budesonide: 4/136 Placebo: 0/68 Not clear on particulars; the 1/68 likely a skull fracture	Budesonide 0.5 mg: 57/68 Budesonide 1.0 mg: 59/68 Budesonide: 116/ Placebo = 61/68 Cartilage injury: 1 (1.5%), 0, 0; upper limb fracture: 1 (1.5%), 0, 0; sinusitis: 1 (1.5%), 0, 0; inguinal hernia: 1 (1.5%), 0, 0; skull fracture: 0, 1 (1.5%), 0; condition aggravated (clinical relapse%): 7 (10.3%), 5 (7.4%), 41 (60.3%); food impaction needing endoscopic intervention: 0, 0, 2 (2.9%); chest pain: 0, 1 (1.5%), 0; retinitis: 0, 1 (1.5%), 0; oropharyngeal pain: 0, 1 (1.5%), 0; dermatitis allergic: 0, 1 (1.5%), 0; esophageal dilation: 0, 0, 1 (1.5%); food impaction needing endoscopic intervention: 0, 0, 2 (2.9%); food impaction without need for endoscopic intervention: 0, 3 (4.4%), 0; eye disorders: 1 (1.5%), 1 (1.5%), 1 (1.5%); cataract nuclear: 0, 0, 1 (1.5%); gastrointestinal disorders: 5 (7.4%), 5 (7.4%), 0; general disorders and administration site conditions: 2 (2.9%), 2 (2.9%), 0; infections and infestations: 12 (17.6%), 10 (14.7%), 1 (1.5%); candidiasis overall: 12 (17.6%), 9 (13.2%), 0; suspected symptomatic candidiasis: 11 (16.2%), 8 (11.8%), 0; histologic confirmed candidiasis: 5 (7.4%), 2 (2.9%), 0; histologic confirmed and symptomatic candidiasis: 4 (5.9%), 1 (1.5%), 0; investigations: 3 (4.4%), 2 (2.9%), 0; blood cortisol decreased: 2 (2.9%),e 2 (2.9%), 0; neoplasms benign, malignant and unspecified: 0, 1 (1.5%), 0; lipoma: 0, 1 (1.5%), 0; nervous system disorders: 3 (4.4%), 3 (4.4%), 0; dysgeusia: 0, 1 (1.5%), 0; reproductive system and breast disorders: 0, 1 (1.5%), 1 (1.5%); respiratory, thoracic and mediastinal disorders: 0, 1 (1.5%), 0; skin and subcutaneous tissue disorders: 1 (1.5%), 3 (4.4%), 0; vascular disorders: 0, 1 (1.5%), 0; hypertension: 0, 1 (1.5%), 0	Eosinophilic esophagitis quality of life scale for adults (EoE‐QoL‐A) questionnaire version 2.0 S. Bajaj, T. Taft, L. Keefer, et al. Validity, usability, and acceptability of the eosinophilic esophagitis quality of life scale for adults (EoE‐QOL‐A) T.H. Taft, E. Kern, M.A. Kwiatek, et al. The adult eosinophilic oesophagitis quality of life questionnaire: a new measure of health‐related quality of life Aliment Pharmacol There, 34 (2011), pp. 790‐798 EoE‐QoL‐A end of treatment (mean ± SD) BOT 0.5: ‐3.3 ± 0.46/68 BOT 1.0: ‐3.5 ± 0.48/68 BOT: ‐3.4 (0.48)/136 Placebo: ‐2.8 ± 0.75/68
Tytor 2021	Mometasone: 0/17 Placebo: 0/19	Mometasone: 0/17 Placebo: 0/19	The organ‐related QoL was evaluated using the EORTC QLQ‐OES18 (originally developed and validated for patients with esophagus cancer) (References: Blazeby JM, Conroy T, Hammerlid E, et al. Clinical and psychometric validation of an EORTC questionnaire module, the EORTC QLQOES18, to assess quality of life in patients with oesophageal cancer. Eur J Cancer. 2003;39(10):1384–1394. Blazeby JM, Alderson D, Winstone K, et al. Development of an EORTC questionnaire module to be used in quality of life assessment for patients with oesophageal cancer. The EORTC Quality of Life Study Group. Eur J Cancer. 1996;32(11):1912–1917) General QoL, Short Form‐36 (SF‐36) (Sullivan M, Karlsson J, Ware JE. Jr., The Swedish SF‐36 Health Survey–I. Evaluation of data quality, scaling assumptions, reliability and construct validity across general populations in Sweden. Soc Sci Med. 1995;41(10):1349–1358) No difference between placebo and intervention although no numerical data provided

AAF: amino acid‐based formula; AE: adverse event; BET: budesonide effervescent tablet; BOS: budesonide oral suspension; BOT: budesonide orodispersible tablet; BOV: budesonide, oral viscus; CG: control group; EoE: eosinophilic esophagitis; EoT: end of treatment; FFED: four food elimination diet; FP: fluticasone propionate; GERD: gastroesophageal reflux disease; IG: intervention group; NEB: nebulized/swallowed budesonide solution; LS: least squares; QOL: quality of life; SAE: serious adverse event; SE: standard error; TEAE: treatment‐emergent adverse event; WBC: white blood cell

#### Corticosteroids versus placebo for induction of remission

Fourteen studies compared corticosteroids to placebo for induction of remission (Alexander 2012; Bhardwaj 2017; Butz 2014; Dellon 2017; Dellon 2022a; Dohil 2010; Gupta 2015; Hirano 2020f; Hirano 2021; Konikoff 2006; Lucendo 2019; Miehlke 2016; Straumann 2010b; Tytor 2021).

##### Primary outcomes

###### Clinical improvement

Six studies compared corticosteroids to placebo for clinical improvement as a dichotomous outcome (Alexander 2012; Bhardwaj 2017; Gupta 2015; Hirano 2021; Lucendo 2019; Straumann 2010b).

Corticosteroids (n = 210/380) may lead to slightly better clinical improvement compared to placebo (n = 71/203), measured as a dichotomous outcome (risk ratio (RR) 1.74, 95% confidence interval (CI) 1.08 to 2.80). The results are of low certainty due to inconsistency and imprecision (Analysis 1.1; Table 1).

Sensitivity analyses using a fixed‐effect model (RR 1.54, 95% CI 1.25 to 1.89; Analysis 1.2), and for validated instruments (RR 1.39, 95% CI 1.08 to 1.79; Analysis 1.3), showed similar results.

The subgroup analyses provided only limited explanation for the variation in treatment effect across the studies. While there was a statistically significant difference in the interaction test for subgroup analysis by age (Analysis 1.4), this was based on only one study in children younger than 18 years old (Gupta 2015), and five studies in mixed adult and child participants (Alexander 2012; Bhardwaj 2017; Hirano 2021; Lucendo 2019; Straumann 2010b). The remaining subgroup analyses were based on type of corticosteroid (one beclomethasone study (Bhardwaj 2017), four budesonide studies (Gupta 2015; Hirano 2021; Lucendo 2019; Straumann 2010b), and one fluticasone study (Alexander 2012)), and based on corticosteroid delivery method (three studies using an adapted asthma delivery method (Alexander 2012; Bhardwaj 2017; Straumann 2010b), and three studies using an esophageal‐specific method (Gupta 2015; Hirano 2021; Lucendo 2019)). There was insufficient evidence in these subgroup analyses to determine whether there were any differences in the subgroup effects (see Analysis 1.5 and Analysis 1.6).

Five studies compared corticosteroids to placebo for clinical improvement as a continuous outcome (Dellon 2017; Dohil 2010; Hirano 2021; Straumann 2010b; Tytor 2021).

Corticosteroids (n = 302) may lead to slightly better clinical improvement compared to placebo (n = 173), measured as a continuous outcome (standardized mean difference (SMD) 0.51, 95% CI 0.17 to 0.85). The results are of low certainty due to inconsistency and imprecision (Analysis 1.7; Table 1).

Sensitivity analyses using a fixed‐effect model (SMD 0.37, 95% CI 0.18 to 0.56; Analysis 1.8), and for validated instruments (SMD 0.35, 95% CI 0.07 to 0.64; Analysis 1.9), showed similar results.

Again, the subgroup analyses provided limited explanation for the variation in treatment effect. The test for subgroup differences by age did not show a statistical difference (Analysis 1.10), however it was only based on one study in children younger than 18 years old (Dohil 2010), and four studies on mixed adult and child participants (Dellon 2017; Hirano 2021; Straumann 2010b; Tytor 2021). Similarly, we observed no difference for the type of corticosteroid (Analysis 1.11), based on four studies of budesonide (Dellon 2017; Dohil 2010; Hirano 2021; Straumann 2010b), and one of mometasone (Tytor 2021). However, we observed a difference between subgroups for corticosteroid delivery method (Analysis 1.12), based on one study using an adapted asthma delivery method (Straumann 2010b), and four using an esophageal‐specific method (Dellon 2017; Dohil 2010; Hirano 2021; Tytor 2021).

###### Histological improvement

Twelve studies compared corticosteroids to placebo for histological improvement as a dichotomous outcome (Alexander 2012; Butz 2014; Dellon 2017; Dellon 2022a; Dohil 2010; Gupta 2015; Hirano 2020f; Hirano 2021; Konikoff 2006; Lucendo 2019; Miehlke 2016; Straumann 2010b).

Corticosteroids (n = 428/652) lead to a large histological improvement compared to placebo (n = 10/326), measured as a dichotomous outcome (RR 11.94, 95% CI 6.56 to 21.75, NNTB = 3). These results are of high certainty (Analysis 1.13; Table 1).

Funnel plot inspection did not reveal publication bias (Figure 3).

**3:**
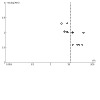


A sensitivity analysis using a fixed‐effect model showed similar results but a higher magnitude of effect (RR 18.87, 95% CI 10.57 to 33.71; Analysis 1.14).

Sensitivity analyses for histological thresholds of < 15 eos/hpf (RR 18.47, 95% CI 4.45 to 76.72; Analysis 1.15), ≤ 6 eos/hpf (RR 14.03, 95% CI 6.73 to 29.26; Analysis 1.16), and ≤ 1 eos/hpf (RR 10.97, 95% CI 3.12 to 38.55; Analysis 1.17) showed similar results.

A subgroup analysis for participant age, based on three studies in children younger than 18 years old (Dohil 2010; Gupta 2015; Konikoff 2006), and eight studies in mixed adult and child participants did not reveal differences (Alexander 2012; Butz 2014; Dellon 2017; Dellon 2022a; Hirano 2021; Lucendo 2019; Miehlke 2016; Straumann 2010b) (Analysis 1.18).

A subgroup analysis for the type of corticosteroid, with seven studies of budesonide (Dellon 2017; Dohil 2010; Gupta 2015; Hirano 2021; Lucendo 2019; Miehlke 2016; Straumann 2010b), and four studies of fluticasone (Alexander 2012; Butz 2014; Dellon 2022a ; Konikoff 2006), did not reveal differences (Analysis 1.19).

A subgroup analysis for corticosteroid delivery method, based on seven studies using an esophageal‐specific method (Dellon 2017; Dellon 2022a; Dohil 2010; Gupta 2015; Hirano 2021; Lucendo 2019; Miehlke 2016), and four using an adapted asthma delivery method (Alexander 2012; Butz 2014; Konikoff 2006; Straumann 2010b), did not reveal differences (Analysis 1.20).

Five studies compared corticosteroids to placebo for histological improvement as a continuous outcome (Bhardwaj 2017; Dellon 2017; Dohil 2010; Hirano 2021; Straumann 2010b).

Corticosteroids (n = 287) may lead to histological improvement compared to placebo (n = 162), measured as a continuous outcome (SMD 1.42, 95% CI 1.02 to 1.82). The results are of low certainty, due to inconsistency and risk of bias (Analysis 1.21; Table 1).

A sensitivity analysis using a fixed‐effect model showed similar results (SMD 1.33, 95% CI 1.12 to 1.55; Analysis 1.22).

Little insight could be gained from the subgroup analyses. We observed no differences between subgroups for the following: participant age (Analysis 1.23), based on only one study in children younger than 18 years old (Dohil 2010), and four studies in mixed adult and child participants (Bhardwaj 2017; Dellon 2017; Hirano 2021; Straumann 2010b); the type of steroid (Analysis 1.24), based on only one study of beclomethasone (Bhardwaj 2017), and four studies of budesonide (Dellon 2017; Dohil 2010; Hirano 2021; Straumann 2010b); and corticosteroid delivery method (Analysis 1.25), based on two studies using an adapted asthma delivery method (Bhardwaj 2017; Straumann 2010b), and three studies using an esophageal‐specific method (Dellon 2017; Dohil 2010; Hirano 2021).

###### Endoscopic improvement

Three studies compared corticosteroids to placebo for endoscopic improvement as a dichotomous outcome (Alexander 2012; Hirano 2020f; Konikoff 2006).

Corticosteroids (n = 25/58) may lead to little to no endoscopic improvement compared to placebo (n = 6/44), measured as a dichotomous outcome (RR 2.60, 95% CI 0.82 to 8.19). The results are of low certainty due to serious imprecision (Analysis 1.26; Table 1).

A sensitivity analysis using a fixed‐effect model showed similar results (RR 2.73, 95% CI 1.27 to 5.86; Analysis 1.27).

We cannot draw any conclusions from a sensitivity analysis based on validated instruments (RR 5.87, 95% CI 1.11 to 31.02; Analysis 1.28). The results are of very low certainty due to serious imprecision and risk of bias.

Subgroup analyses for participant age (Analysis 1.29), and corticosteroid delivery method (Analysis 1.30), provided limited explanation for the variation in treatment effect. They were based on only one study in children younger than 18 years old (Konikoff 2006), and two studies in mixed adult and child participants (Alexander 2012; Hirano 2020f), and two studies using an adapted asthma delivery method (Alexander 2012; Konikoff 2006), and one study using an esophageal‐specific method (Hirano 2020f).

Five studies compared corticosteroids to placebo for endoscopic improvement as a continuous outcome (Dellon 2017; Dellon 2022a; Dohil 2010; Hirano 2021; Lucendo 2019).

Corticosteroids (n = 409) may lead to endoscopic improvement compared to placebo (n = 187), measured as a continuous outcome (SMD 1.33, 95% CI 0.59 to 2.08). The results are of low certainty due to serious inconsistency (Analysis 1.31; Table 1).

A sensitivity analysis using a fixed‐effect model showed similar results (SMD 0.93, 95% CI 0.74 to 1.11; Analysis 1.32).

We cannot draw any conclusions from a sensitivity analysis based on validated instruments (SMD 1.31, 95% CI 0.46 to 2.17; Analysis 1.33).

A subgroup analysis for participant age, based on one study in children younger than 18 years old (Dohil 2010), and four studies in mixed adult and child participants (Dellon 2017; Dellon 2022a; Hirano 2021; Lucendo 2019), revealed no differences between subgroups and there was no statistical difference from placebo in both groups. This was based on limited data for children (Analysis 1.34).

A subgroup analysis for type of steroid, based on four studies of budesonide (Dellon 2017; Dohil 2010; Hirano 2021; Lucendo 2019), and one study of fluticasone (Dellon 2022a), showed similar statistically significant differences from placebo in both groups (Analysis 1.35).

###### Withdrawals due to adverse events

Fourteen studies provided data on withdrawals due to adverse events when corticosteroids were compared to placebo (Alexander 2012; Bhardwaj 2017; Butz 2014; Dellon 2017; Dellon 2022a; Dohil 2010; Gupta 2015; Hirano 2020f; Hirano 2021; Konikoff 2006; Lucendo 2019; Miehlke 2016; Straumann 2010b; Tytor 2021).

Corticosteroids (n = 59/678) may lead to slightly fewer withdrawals due to adverse events compared to placebo (n = 44/354) (RR 0.64, 95% CI 0.43 to 0.96; Analysis 1.36). The results are of low certainty due to imprecision and risk of bias (Table 1).

A sensitivity analysis using a fixed‐effect model showed similar effects (RR 0.65, 95% CI 0.44 to 0.94; Analysis 1.37).

Subgroup analysis for the following did not reveal any differences between subgroups (Analysis 1.38; Analysis 1.39; Analysis 1.40): participant age, based on three studies in children younger than 18 years old (Dohil 2010; Gupta 2015; Konikoff 2006), and 11 studies in mixed adult and child participants (Alexander 2012; Bhardwaj 2017; Butz 2014; Dellon 2017; Dellon 2022a; Hirano 2020f; Hirano 2021; Lucendo 2019; Miehlke 2016; Straumann 2010b; Tytor 2021); type of steroid, based on one study of beclomethasone (Bhardwaj 2017), seven of budesonide (Dellon 2017; Dohil 2010; Gupta 2015; Hirano 2021; Lucendo 2019; Miehlke 2016; Straumann 2010b), five of fluticasone (Alexander 2012; Butz 2014; Dellon 2022a; Hirano 2020f; Konikoff 2006), and one of mometasone (Tytor 2021); and corticosteroid delivery method, based on five studies using an adapted asthma method (Alexander 2012; Bhardwaj 2017; Butz 2014; Konikoff 2006; Straumann 2010b), and nine studies using an esophageal‐specific method (Dellon 2017; Dellon 2022a; Dohil 2010; Gupta 2015; Hirano 2020f; Hirano 2021; Lucendo 2019; Miehlke 2016; Tytor 2021).

##### Secondary outcomes

###### Serious adverse events

Fourteen studies provided data on serious adverse events when corticosteroids were compared to placebo (Alexander 2012; Bhardwaj 2017; Butz 2014; Dellon 2017; Dellon 2022a; Dohil 2010; Gupta 2015; Hirano 2020f; Hirano 2021; Konikoff 2006; Lucendo 2019; Miehlke 2016; Straumann 2010b; Tytor 2021).

Corticosteroids (n = 11/678) may result in little to no difference in serious adverse events compared to placebo (n = 14/354) (RR 0.35, 95% CI 0.17 to 0.73; Analysis 1.41). The results are of low certainty due to serious imprecision.

###### Total adverse events

Thirteen studies provided data on total adverse events when corticosteroids were compared to placebo (Alexander 2012; Butz 2014; Dellon 2017; Dellon 2022a; Dohil 2010; Gupta 2015; Hirano 2020f; Hirano 2021; Konikoff 2006; Lucendo 2019; Miehlke 2016; Straumann 2010b; Tytor 2021).

Corticosteroids (n = 290/669) probably result in little to no difference in total adverse events compared to placebo (n = 111/345) (RR 1.14, 95% CI 0.94 to 1.40; Analysis 1.42). The results are of moderate certainty due to imprecision.

###### Quality of life

One study provided continuous data on quality of life when corticosteroids were compared to placebo (Lucendo 2019).

Corticosteroids (n = 59) may result in little to no difference in quality of life compared to placebo (n = 29) (mean difference (MD) 0.20, 95% CI ‐0.14 to 0.54; Analysis 1.43). The results are of low certainty due to serious imprecision.

#### Corticosteroids versus placebo for maintenance of remission

Three studies compared corticosteroids to placebo for maintenance of remission (Dellon 2021b; Straumann 2011; Straumann 2020).

##### Primary outcomes

###### Clinical improvement

Two studies compared corticosteroids to placebo for clinical improvement as a dichotomous outcome (Dellon 2021b; Straumann 2020).

We cannot draw any conclusions on the effects of corticosteroids (n = 118/161) on clinical improvement for maintenance of remission compared to placebo (n = 20/91), measured as a dichotomous outcome (RR 2.17, 95% CI 0.75 to 6.27). The results are of very low certainty due to serious inconsistency and imprecision (Analysis 2.1; Table 2).

Three studies compared corticosteroids to placebo for clinical improvement as a continuous outcome (Dellon 2021b; Straumann 2011; Straumann 2020).

We cannot draw any conclusions on the effects of corticosteroids (n = 169) on clinical improvement for maintenance of remission compared to placebo (n = 100), measured as a continuous outcome (SMD 0.51, 95% CI ‐0.49 to 1.52). The results are of very low certainty due to serious inconsistency and imprecision (Analysis 2.2; Table 2).

###### Histological improvement

Three studies compared corticosteroids to placebo for histological improvement as a dichotomous outcome (Dellon 2021b; Straumann 2011; Straumann 2020).

Corticosteroids (n = 146/175) probably result in histological improvement for maintenance of remission compared to placebo (n = 14/105), measured as a dichotomous outcome (RR 4.58, 95% CI 1.66 to 12.62, NNTB = 3). The results are of moderate certainty, downgraded once due to imprecision (Analysis 2.3; Table 2).

Three studies compared corticosteroids to placebo for histological improvement as a continuous outcome (Dellon 2021b; Straumann 2011; Straumann 2020).

Corticosteroids (n = 169) probably result in large histological improvement for maintenance of remission compared to placebo (n = 100), measured as a continuous outcome (SMD 1.26, 95% CI 0.74 to 1.78). The results are of moderate certainty due to inconsistency (Analysis 2.4; Table 2).

###### Endoscopic improvement

Two studies compared corticosteroids to placebo for clinical improvement as a continuous outcome (Dellon 2021b; Straumann 2020).

We cannot draw any conclusions on the effects of corticosteroids (n = 154) on endoscopic improvement for maintenance of remission compared to placebo (n = 86), measured as a continuous outcome (SMD 1.34, 95% CI ‐0.27 to 2.95). The results are of very low certainty due to serious inconsistency and imprecision (Analysis 2.5; Table 2).

###### Withdrawals due to adverse events

Three studies provided data on withdrawals due to adverse events when corticosteroids were compared to placebo for maintenance of remission (Dellon 2021b; Straumann 2011; Straumann 2020).

Corticosteroids (n = 26/175) may lead to fewer withdrawals due to adverse events for maintenance of remission compared to placebo (n = 58/105) (RR 0.37, 95% CI 0.16 to 0.87). The results are of low certainty due to inconsistency and imprecision (Analysis 2.6; Table 2).

##### Secondary outcomes

###### Serious adverse events

Three studies provided data on serious adverse events when corticosteroids were compared to placebo for maintenance of remission (Dellon 2021b; Straumann 2011; Straumann 2020).

We cannot draw any conclusions on the effects of corticosteroids (n = 4/175) on serious adverse events for maintenance of remission compared to placebo (n = 1/105) (RR 1.27, 95% CI 0.09 to 18.03; Analysis 2.7). The results are of low certainty due to serious imprecision.

###### Total adverse events

Three studies provided data on total adverse events when corticosteroids were compared to placebo for maintenance of remission (Dellon 2021b; Straumann 2011; Straumann 2020).

We cannot draw any conclusions on the effects of corticosteroids (n = 133/175) on total adverse events for maintenance of remission compared to placebo (n = 75/105) (RR 1.10, 95% CI 0.75 to 1.62; Analysis 2.8). The results are of very low certainty due to serious inconsistency and imprecision.

###### Quality of life

One study provided continuous data on quality of life when corticosteroids were compared to placebo for maintenance of remission (Straumann 2020).

Corticosteroids (n = 136) may lead to improved quality of life compared to placebo (n = 68), measured as a continuous outcome (MD 0.60, 95% CI 0.40 to 0.80; Analysis 2.9). The results are of low certainty due to serious imprecision.

#### Biologics versus placebo for induction of remission

Nine studies compared biologics to placebo for induction of remission (Clayton 2014; Dellon 2022; Dellon 2022b; Hirano 2019; Hirano 2020; Rothenberg 2015; Rothenberg 2022; Spergel 2012; Straumann 2010a).

##### Primary outcomes

###### Clinical improvement

Five studies compared biologics to placebo for clinical improvement as a dichotomous outcome (Hirano 2019; Hirano 2020; Rothenberg 2015; Spergel 2012; Straumann 2010a).

Biologics (n = 169/281) may result in little to no difference in clinical improvement compared to placebo (n = 65/129), measured as a dichotomous outcome (RR 1.14, 95% CI 0.85 to 1.52). The results are of low certainty due to inconsistency and imprecision (Analysis 3.1; Table 3).

Sensitivity analyses using a fixed‐effect model (RR 1.10, 95% CI 0.92 to 1.32; Analysis 3.2), and based on validated instruments (RR 1.37, 95% CI 1.02 to 1.85; Analysis 3.3), showed similar results.

Subgroup analysis for participant age, based on only one study in children younger than 18 years old (Spergel 2012), and four studies in mixed adult and child participants (Hirano 2019; Hirano 2020; Rothenberg 2015; Straumann 2010a), showed differences between the two age groups, with the mixed age group showing a statistically significant effect for the intervention, while no effect was observed for the children. Subgroup analysis for biologic mechanism, based on three studies of anti‐IL‐13 or anti‐IL‐4ra (Hirano 2019; Hirano 2020; Rothenberg 2015), and two studies of anti‐IL‐5 (Spergel 2012; Straumann 2010a), showed differences between the two groups, with the IL‐13 group showing a statistically significant effect for the intervention, while no effect was observed for the IL‐5 group (Analysis 3.4; Analysis 3.5).

Seven studies compared biologics to placebo for clinical improvement as a continuous outcome (Clayton 2014; Dellon 2022; Hirano 2019; Hirano 2020; Rothenberg 2015; Rothenberg 2022; Straumann 2010a).

Biologics (n = 195) may result in better clinical improvement compared to placebo (n = 192), measured as a continuous outcome (SMD 0.50, 95% CI 0.22 to 0.78). The results are of moderate certainty due to imprecision (Analysis 3.6; Table 3).

Sensitivity analyses using a fixed‐effect model (SMD 0.48, 95% CI 0.28 to 0.69; Analysis 3.7), risk of bias (SMD 0.61, 95% CI 0.40 to 0.82; Analysis 3.9), and for validated instruments (SMD 0.62, 95% CI 0.37 to 0.88; Analysis 3.10) showed similar results.

A sensitivity analysis for peer‐reviewed manuscripts showed slightly different results (SMD 0.36, 95% CI ‐0.09 to 0.81; Analysis 3.8).

A subgroup analysis for biologic mechanism, based on one study of anti‐IgE (Clayton 2014), five studies of anti‐IL‐13/anti‐IL‐4ra (Dellon 2022; Hirano 2019; Hirano 2020; Rothenberg 2015; Rothenberg 2022), and one study of anti‐IL‐5 (Straumann 2010a), showed differences between the three groups, with statistically significant difference from placebo for anti‐IL13/anti‐IL‐4r, however the data are very limited for the other categories (Analysis 3.11).

###### Histological improvement

Eight studies compared biologics to placebo for histological improvement as a dichotomous outcome (Dellon 2022; Dellon 2022b; Hirano 2019; Hirano 2020; Rothenberg 2015; Rothenberg 2022; Spergel 2012; Straumann 2010a).

Biologics (n = 394/586) may result in better histological improvement compared to placebo (n = 39/339), measured as a dichotomous outcome (RR 6.73, 95% CI 2.58 to 17.52, NNTB = 2). The results are of moderate certainty, downgraded once due to imprecision (Analysis 3.12; Table 3).

Sensitivity analyses using a fixed‐effect model (RR 5.12, 95% CI 3.86 to 6.78; Analysis 3.13), peer‐reviewed manuscripts (RR 6.13, 95% CI 1.67 to 22.51; Analysis 3.14), risk of bias (RR 6.73, 95% CI 2.58 to 17.52; Analysis 3.15), and for different histological thresholds showed similar results (RR 5.61, 95% CI 1.00 to 31.32; Analysis 3.16; RR 8.85, 95% CI 5.73 to 13.67; Analysis 3.17; RR 18.01, 95% CI 7.24 to 44.83; Analysis 3.18).

A subgroup analysis for participant age, based on two studies in children younger than 18 years old (Dellon 2022b; Spergel 2012), six studies in mixed adult and children participants (Dellon 2022; Hirano 2019; Hirano 2020; Rothenberg 2015; Rothenberg 2022; Straumann 2010a), and one study that provided separate data on children and adults (Dellon 2022b), revealed no evidence of a difference between the age subgroups (Analysis 3.19).

A subgroup analysis for biologic mechanism (Analysis 3.20), based on one study on anti‐sialic acid binding Ig‐like lectin 8 (Dellon 2022b), five studies of anti‐IL‐13/anti‐IL‐4ra (Dellon 2022; Hirano 2019; Hirano 2020; Rothenberg 2015; Rothenberg 2022), and two studies of anti‐IL‐5 (Spergel 2012; Straumann 2010a), showed significant differences between all categories, but it was based on limited data.

Six studies compared biologics to placebo for histological improvement as a continuous outcome (Clayton 2014; Hirano 2019; Hirano 2020; Rothenberg 2015; Spergel 2012; Straumann 2010a).

We cannot draw any conclusions on the effects of biologics (n = 240) on histological improvement compared to placebo (n = 130) measured as a continuous outcome (SMD 1.01, 95% CI 0.36 to 1.66). The results are of very low certainty due to serious inconsistency and imprecision (Analysis 3.21; Table 3).

A sensitivity analysis using a fixed‐effect model showed that biologics (n = 240) may lead to better histological improvement when compared to placebo (n = 130), measured as a continuous outcome (SMD 1.25, 95% CI 1.01 to 1.49; Analysis 3.22). A sensitivity analysis for risk of bias showed that biologics (n = 224) probably result in better histological improvement compared to placebo (n = 116), measured as a continuous outcome (SMD 1.39, 95% CI 1.01 to 1.77; Analysis 3.23).

A subgroup analysis for participant age, based on one study in children younger than 18 years old (Spergel 2012), and five studies in mixed adult and child participants (Clayton 2014; Hirano 2019; Hirano 2020; Rothenberg 2015; Straumann 2010a), revealed no differences between age groups, however this was based on limited data (Analysis 3.24).

A subgroup analysis for biologic mechanism, based on one study of anti‐IgE (Clayton 2014), three studies of anti‐IL‐13/anti‐IL‐4ra (Hirano 2019; Hirano 2020; Rothenberg 2015), and two studies of anti‐IL‐5 (Spergel 2012; Straumann 2010a), showed differences between groups, with statistically significant effects for anti‐IL‐13 and anti‐IL‐5, however this was also based on limited data (Analysis 3.25).

###### Endoscopic improvement

One study compared biologics to placebo for endoscopic improvement, measured as a dichotomous outcome (Straumann 2010a).

Biologics (n = 0/5) may result in little to no difference in endoscopic improvement compared to placebo (n = 0/6), measured as a dichotomous outcome (effect not estimable). The results are of low certainty due to serious imprecision (Analysis 3.26; Table 3).

Three studies compared biologics to placebo for endoscopic improvement, measured as a continuous outcome (Dellon 2022; Hirano 2019; Hirano 2020).

We cannot draw any conclusions about the effects of biologics (n = 115) on endoscopic improvement compared to placebo (n = 82), measured as a continuous outcome (SMD 2.79, 95% CI 0.36 to 5.22; Analysis 3.27). The results are of very low certainty due to serious inconsistency and imprecision.

A sensitivity analysis using a fixed‐effect model showed similar results (SMD 1.20, 95% CI 0.86 to 1.55; Analysis 3.28). A sensitivity analysis for risk of bias showed that biologics (n = 80) may result in slightly better endoscopic improvement compared to placebo (n = 56), measured as a continuous outcome (SMD 0.82, 95% CI 0.42 to 1.21; Analysis 3.29). The results are of low certainty due to serious imprecision.

###### Withdrawals due to adverse events

Eight studies provided data on withdrawals due to adverse events when biologics were compared to placebo (Clayton 2014; Dellon 2022; Dellon 2022b; Hirano 2019; Hirano 2020; Rothenberg 2015; Spergel 2012; Straumann 2010a).

There may be no difference between biologics (n = 54/518) and placebo (n = 16/274) in withdrawals due to adverse events (RR 1.55, 95% CI 0.88 to 2.74). The results are of low certainty due to serious imprecision (Analysis 3.30; Table 3).

Sensitivity analyses using a fixed‐effect model (RR 1.53, 95% CI 0.89 to 2.64; Analysis 3.31), risk of bias (RR 1.55, 95% CI 0.88 to 2.74; Analysis 3.32), and peer‐reviewed publications (RR 1.55, 95% CI 0.88 to 2.74; Analysis 3.33) showed similar results.

Subgroup analyses for the following did not reveal differences between the subgroups (Analysis 3.34; Analysis 3.35): participant age, based on one study in children younger than 18 years old (Spergel 2012), and seven studies in mixed adult and child participants (Clayton 2014; Dellon 2022; Dellon 2022b; Hirano 2019; Hirano 2020; Rothenberg 2015; Straumann 2010a); and biologic mechanism, based on one study of anti‐IgE (Clayton 2014), one study of anti‐sialic acid‐binding Ig‐like lectin 8 (Dellon 2022b), four studies of anti‐IL‐13 (Dellon 2022; Hirano 2019; Hirano 2020; Rothenberg 2015), and two studies of anti‐IL‐5 (Spergel 2012; Straumann 2010a).

##### Secondary outcomes

###### Serious adverse events

Six studies provided data on serious adverse events when biologics were compared to placebo (Dellon 2022b; Hirano 2019; Hirano 2020; Rothenberg 2015; Spergel 2012; Straumann 2010a).

There may be no difference between biologics (n = 10/464) and placebo (n = 6/221) in serious adverse events (RR 0.70, 95% CI 0.25 to 1.97; Analysis 3.36). The results are of low certainty due to serious imprecision.

###### Total adverse events

Six studies provided data on total adverse events when biologics were compared to placebo (Dellon 2022b; Hirano 2019; Hirano 2020; Rothenberg 2015; Spergel 2012; Straumann 2010a).

There is probably no difference between biologics (n = 310/464) and placebo (n = 136/221) in total adverse events (RR 1.07, 95% CI 0.94 to 1.23; Analysis 3.37). The results are of moderate certainty due to imprecision.

###### Quality of life

One study provided continuous data on quality of life when biologics were compared to placebo (Hirano 2020).

There may be little to no difference between biologics (n = 23) and placebo (n = 24) in quality of life (MD 0.33, 95% CI ‐0.06 to 0.72; Analysis 3.38). The results are of low certainty due to serious imprecision.

#### Cromolyn sodium versus placebo

One study compared cromolyn sodium to placebo for induction of remission (Lieberman 2018).

##### Primary outcomes

###### Clinical improvement

Cromolyn sodium (n = 8) may result in little to no difference in clinical improvement compared to placebo (n = 6), measured as a continuous outcome (MD 4.70, 95% CI ‐12.09 to 21.49). The results are of low certainty due to serious imprecision (Analysis 4.1; Table 4).

###### Histological improvement

Cromolyn sodium (n = 9) may result in little to no difference in histological improvement compared to placebo (n = 6), measured as a continuous outcome (MD 14.20, 95% CI ‐36.90 to 65.30). The results are of low certainty due to serious imprecision (Analysis 4.2; Table 4).

###### Endoscopic improvement

There were no data for meta‐analysis for this outcome.

####### Withdrawals due to adverse events

Cromolyn sodium (n = 0/9) may result in little to no difference in withdrawals due to adverse events compared to placebo (n = 1/7) (RR 0.27, 95% CI 0.01 to 5.70). The results are of low certainty due to serious imprecision (Analysis 4.3; Table 4).

##### Secondary outcomes

###### Serious adverse events

Cromolyn sodium (n = 0/9) may result in little to no difference in serious adverse events compared to placebo (n = 0/7) (effect not estimable; Analysis 4.4). The results are of low certainty due to serious imprecision

###### Total adverse events

There were no data for this outcome.

###### Quality of life

There were no data for this outcome.

#### PGD2R antagonist OC000459 versus placebo

One study compared PGD2R antagonist OC000459 to placebo for induction of remission (Straumann 2013).

##### Primary outcomes

###### Clinical improvement

We cannot draw any conclusions on the effect of PGD2R antagonist (n = 14) on clinical improvement compared to placebo (n = 12), measured as a continuous outcome (MD ‐1.06, 95% CI ‐6.80 to 4.68). The results are of very low certainty due to serious imprecision and risk of bias (Analysis 5.1; Table 5).

###### Histological improvement

We cannot draw any conclusions on the effect of PGD2R antagonist (n = 14) on histological improvement compared to placebo (n = 12), measured as a continuous outcome (MD 26.21, 95% CI ‐23.78 to 76.20). The results are of very low certainty due to serious imprecision and risk of bias (Analysis 5.2; Table 5).

###### Endoscopic improvement

We cannot draw any conclusions on the effect of PGD2R antagonist (n = 14) on histological improvement compared to placebo (n = 12), measured as a continuous outcome (MD ‐0.49, 95% CI ‐2.05 to 1.07). The results are of very low certainty due to serious imprecision and risk of bias (Analysis 5.3; Table 5).

###### Withdrawals due to adverse events

We cannot draw any conclusions on the effect of PGD2R antagonist (n = 0/14) on withdrawals due to adverse events compared to placebo (n = 0/12) (effect not estimable). The results are of very low certainty due to serious imprecision and risk of bias (Analysis 5.4; Table 5).

##### Secondary outcomes

###### Serious adverse events

We cannot draw any conclusions on the effect of PGD2R antagonist (n = 1/14) on serious adverse events compared to placebo (n = 0/12) (RR 2.60, 95% CI 0.12 to 58.48 ; Analysis 5.5). The results are of very low certainty due to serious imprecision and risk of bias.

###### Total adverse events

There were no data for this outcome.

###### Quality of life

There were no data for this outcome.

#### Swallowed fluticasone versus oral prednisone

One study compared swallowed fluticasone to oral prednisone for induction of remission (Schaefer 2008).

##### Primary outcomes

###### Clinical improvement

We cannot draw any conclusions on the effects of swallowed fluticasone (n = 35/40) on clinical improvement compared to oral prednisone (n = 32/40), measured as dichotomous outcome (RR 1.09, 95% CI 0.90 to 1.33). The results are of very low certainty due to serious imprecision and risk of bias (Analysis 6.1; Table 6).

###### Histological improvement

We cannot draw any conclusions on the effects of swallowed fluticasone (n = 33/40) on histological improvement compared to oral prednisone (n = 30/40), measured as a dichotomous outcome (RR 1.10, 95% CI 0.87 to 1.38). The results are of very low certainty due to serious imprecision and risk of bias (Analysis 6.2; Table 6).

We cannot draw any conclusions on the effects of swallowed fluticasone (n = 36) on histological improvement compared to oral prednisone (n = 32/40), measured as a continuous outcome (MD ‐4.45, 95% CI ‐9.08 to 0.18). The results are of very low certainty due to serious imprecision and risk of bias (Analysis 6.3; Table 6).

###### Endoscopic improvement

We cannot draw any conclusions on the effects of swallowed fluticasone (n = 34/40) on endoscopic improvement compared to oral prednisone (n = 30/40), measured as dichotomous outcome (RR 1.13, 95% CI 0.91 to 1.41). The results are of very low certainty due to serious imprecision and risk of bias (Analysis 6.4; Table 6).

###### Withdrawals due to adverse events

We cannot draw any conclusions on the effects of swallowed fluticasone (n = 4/40) on withdrawals due to adverse events compared to oral prednisone (n = 8/40) (RR 0.50, 95% CI 0.16 to 1.53). The results are of very low certainty due to serious imprecision and risk of bias (Analysis 6.5; Table 6).

##### Secondary outcomes

###### Serious adverse events

We cannot draw any conclusions on the effects of swallowed fluticasone (n = 0/40) on serious adverse events compared to oral prednisone (n = 3/40) (RR 0.14, 95% CI 0.01 to 2.68; Analysis 6.6). The results are of very low certainty due to serious imprecision and risk of bias.

###### Total adverse events

We cannot draw any conclusions on the effects of swallowed fluticasone (n = 6/40) on total adverse events compared to oral prednisone (n = 16/40) (RR 0.38, 95% CI 0.16 to 0.86; Analysis 6.7). The results are of very low certainty due to serious imprecision and risk of bias.

###### Quality of life

There were no data for this outcome.

#### Oral viscous budesonide versus swallowed fluticasone

One study compared oral viscous budesonide to swallowed fluticasone for induction of remission (Dellon 2019).

##### Primary outcomes

###### Clinical improvement

Oral viscous budesonide (n = 46) may result in little to no difference in clinical improvement compared to swallowed fluticasone (n = 38), measured as a continuous outcome (MD ‐0.60, 95% CI ‐3.78 to 2.58). The results are of low certainty due to serious imprecision (Analysis 7.1; Table 7).

###### Histological improvement

Oral viscous budesonide (n = 40/65) may result in little to no difference in histological improvement compared to swallowed fluticasone (n = 35/64), measured as a dichotomous outcome (RR 1.13, 95% CI 0.84 to 1.51). The results are of low certainty due to serious imprecision (Analysis 7.2; Table 7).

Oral viscous budesonide (n = 56) may result in little to no difference in histological improvement compared to swallowed fluticasone (n = 55), measured as a continuous outcome (MD 6.20, 95% CI ‐5.63 to 18.03). The results are of low certainty due to serious imprecision (Analysis 7.3; Table 7).

###### Endoscopic improvement

Oral viscous budesonide (n = 56) may result in little to no difference in endoscopic improvement compared to swallowed fluticasone (n = 55), measured as a continuous outcome (MD 0.70, 95% CI ‐0.03 to 1.43). The results are of low certainty due to serious imprecision (Analysis 7.4; Table 7).

###### Withdrawals due to adverse events

Oral viscous budesonide (n = 9/65) may result in little to no difference in withdrawals due to adverse events compared to swallowed fluticasone (n = 9/64) (RR 0.98, 95% CI 0.42 to 2.32). The results are of low certainty due to serious imprecision (Analysis 7.5; Table 7).

##### Secondary outcomes

###### Serious adverse events

Oral viscous budesonide (n = 0/65) may result in little to no difference in serious adverse events compared to swallowed fluticasone (n = 1/64) (RR 0.33, 95% CI 0.01 to 7.91; Analysis 7.6). The results are of low certainty due to serious imprecision.

###### Total adverse events

Oral viscous budesonide (n = 10/65) may result in little to no difference in total adverse events compared to swallowed fluticasone (n = 15/64) (RR 0.66, 95% CI 0.32 to 1.35; Analysis 7.7). The results are of low certainty due to serious imprecision.

###### Quality of life

There were no data for this outcome.

#### Esomeprazole versus fluticasone

Two studies compared esomeprazole to fluticasone for induction of remission (Moawad 2013; Peterson 2010).

##### Primary outcomes

###### Clinical improvement

Both studies provided continuous data on clinical improvement (Moawad 2013; Peterson 2010).

We cannot draw any conclusions on the effects of esomeprazole (n = 32) on clinical improvement compared to fluticasone (n = 31), measured as a continuous outcome (SMD 0.32, 95% CI ‐0.88 to 1.52). The results are of very low certainty due to inconsistency, imprecision, and risk of bias (Analysis 8.1; Table 8).

###### Histological improvement

Both studies provided dichotomous and continuous data on histological improvement (Moawad 2013; Peterson 2010).

We cannot draw any conclusions on the effects of esomeprazole (n = 13/36) on histological improvement compared to fluticasone (n = 8/36), measured as a dichotomous outcome (RR 1.62, 95% CI 0.77 to 3.41). The results are of very low certainty due to serious imprecision and risk of bias (Analysis 8.2; Table 8).

We cannot draw any conclusions on the effects of esomeprazole (n = 33) on histological improvement compared to fluticasone (n = 34), measured as a continuous outcome (SMD 0.28, 95% ‐0.20 to 0.76). The results are of very low certainty due to serious imprecision and risk of bias (Analysis 8.3; Table 8).

###### Endoscopic improvement

No studies provided meta‐analysis data for this outcome.

They reported data on specific endoscopic findings, which can be found in Table 9.

###### Withdrawals due to adverse events

Both studies provided data on withdrawals due to adverse events (Moawad 2013; Peterson 2010).

We cannot draw any conclusions on the effects of esomeprazole (n = 3/36) on withdrawals due to adverse events compared to fluticasone (n = 3/36) (RR 0.95, 95% CI 0.07 to 13.38). The results are of very low certainty due to serious imprecision and risk of bias (Analysis 8.4; Table 8).

##### Secondary outcomes

###### Serious adverse events

We cannot draw any conclusions on the effects of esomeprazole (n = 0/36) on serious adverse events compared to fluticasone (n = 0/36) (effect not estimable; Analysis 8.5). The results are of very low certainty due to serious imprecision and risk of bias.

###### Total adverse events

We cannot draw any conclusions on the effects of esomeprazole (n = 0/33) on total adverse events compared to fluticasone (n = 3/34) (RR 0.14, 95% CI 0.01 to 2.61; Analysis 8.6). The results are of very low certainty due to serious imprecision and risk of bias.

###### Quality of life

There were no data for this outcome.

#### One‐food elimination diet versus four‐food elimination diet

One study compared a one‐food elimination diet to a four‐food elimination diet for induction of remission (Kliewer 2019)

##### Primary outcomes

###### Clinical improvement

We cannot draw any conclusions on the effects of a one‐food elimination diet (n = 33) on clinical improvement compared to a four‐food elimination diet (n = 17), measured as a continuous outcome (MD ‐7.50, 95% CI ‐16.28 to 1.28). The results are of very low certainty due to serious imprecision and risk of bias (Analysis 9.1; Table 10).

###### Histological improvement

We cannot draw any conclusions on the effects of a one‐food elimination diet (n = 24/38) on histological improvement compared to a four‐food elimination diet (n = 7/25), measured as a dichotomous outcome (RR 2.26, 95% CI 1.15 to 4.43). The results are of very low certainty due to serious imprecision and risk of bias (Analysis 9.2; Table 10).

###### Endoscopic improvement

We cannot draw any conclusions on the effects of a one‐food elimination diet (n = 22) on endoscopic improvement compared to a four‐food elimination diet (n = 12), measured as a continuous outcome (MD ‐0.60, 95% CI ‐2.15 to 0.95). The results are of very low certainty due to serious imprecision and risk of bias (Analysis 9.3; Table 10).

###### Withdrawals due to adverse events

We cannot draw any conclusions on the effects of a one‐food elimination diet (n = 4/38) on withdrawals due to adverse events compared to a four‐food elimination diet (n = 8/25) (RR 0.33, 95% CI 0.11 to 0.98). The results are of very low certainty due to serious imprecision and risk of bias (Analysis 9.4; Table 10).

##### Secondary outcomes

###### Serious adverse events

We cannot draw any conclusions on the effects of a one‐food elimination diet (n = 1/38) on serious adverse events compared to a four‐food elimination diet (n = 1/25) (RR 0.66, 95% CI 0.04 to 10.04; Analysis 9.5). The results are of very low certainty due to serious imprecision and risk of bias.

###### Total adverse events

We cannot draw any conclusions on the effects of a one‐food elimination diet (n = 5/38) on total adverse events compared to a four‐food elimination diet (n = 8/25) (RR 0.41, 95% CI 0.15 to 1.11; Analysis 9.6). The results are of very low certainty due to serious imprecision and risk of bias.

###### Quality of life

We cannot draw any conclusions on the effects of a one‐food elimination diet (n = 38) on quality of life compared to a four‐food elimination diet (n = 25), measured as a continuous outcome (MD 0.10, 95% CI ‐6.49 to 6.69; Analysis 9.7). The results are of very low certainty due to serious imprecision and risk of bias.

#### One‐food elimination diet versus six‐food elimination diet

One study compared a one‐food elimination diet to a six‐food elimination diet for induction of remission (Kliewer 2021).

##### Primary outcomes

###### Clinical improvement

We cannot draw any conclusions on the effects of a one‐food elimination diet (n = 67) on clinical improvement compared to a six‐food elimination diet (n = 62), measured as a continuous outcome (MD ‐5.20, 95% CI ‐11.06 to 0.66). The results are of very low certainty due to serious imprecision and risk of bias (Analysis 10.1; Table 11).

###### Histological improvement

We cannot draw any conclusions on the effects of a one‐food elimination diet (n = 23/67) on histological improvement compared to a six‐food elimination diet (n = 25/62), measured as a dichotomous outcome (RR 0.85, 95% CI 0.54 to 1.33). The results are of very low certainty due to serious imprecision and risk of bias (Analysis 10.2; Table 11).

We cannot draw any conclusions on the effects of a one‐food elimination diet (n = 67) on histological improvement compared to a six‐food elimination diet (n = 62), measured as a continuous outcome (MD 6.80, 95% CI ‐10.40 to 24.00). The results are of very low certainty due to serious imprecision and risk of bias (Analysis 10.3; Table 11).

###### Endoscopic improvement

We cannot draw any conclusions on the effects of a one‐food elimination diet (n = 67) on endoscopic improvement compared to a six‐food elimination diet (n = 62), measured as a continuous outcome (MD ‐0.42, 95% CI ‐1.67 to 0.83). The results are of very low certainty due to serious imprecision and risk of bias (Analysis 10.4; Table 11).

###### Withdrawals due to adverse events

We cannot draw any conclusions on the effects of a one‐food elimination diet (n = 2/67) on withdrawals due to adverse events compared to a six‐food elimination diet (n = 3/62) (RR 0.62, 95% CI 0.11 to 3.57). The results are of very low certainty due to serious imprecision and risk of bias (Analysis 10.5; Table 11).

##### Secondary outcomes

###### Serious adverse events

We cannot draw any conclusions on the effects of a one‐food elimination diet (n = 0/67) on serious adverse events compared to a six‐food elimination diet (n = 0/62) (effect not estimable; Analysis 10.6). The results are of very low certainty due to serious imprecision and risk of bias.

###### Total adverse events

We cannot draw any conclusions on the effects of a one‐food elimination diet (n = 1/67) on total adverse events compared to a six‐food elimination diet (n = 2/62) (RR 0.46, 95% CI 0.04 to 4.98; Analysis 10.7). The results are of very low certainty due to serious imprecision and risk of bias.

###### Quality of life

We cannot draw any conclusions on the effects of a one‐food elimination diet (n = 67) on quality of life compared to a six‐food elimination diet (n = 62), measured as a continuous outcome (MD 0.57, 95% CI ‐3.25 to 4.39; Analysis 10.8). The results are of very low certainty due to serious imprecision and risk of bias.

#### Four‐food elimination diet with omeprazole versus omeprazole

One study compared a four‐food elimination diet with omeprazole to omeprazole for induction of remission (Heine 2019).

##### Primary outcomes

###### Clinical improvement

There were no data for this outcome.

###### Histological improvement

We cannot draw any conclusions on the effects of a four‐food elimination diet with omeprazole (n = 22/32) on histological improvement compared to omeprazole (n = 14/32), measured as a dichotomous outcome (RR 1.57, 95% CI 0.99 to 2.48). The results are of very low certainty due to serious imprecision and risk of bias (Analysis 11.1; Table 12).

We cannot draw any conclusions on the effects of a four‐food elimination diet with omeprazole (n = 27) on histological improvement compared to omeprazole (n = 31), measured as a continuous outcome (MD 9.50, 95% CI ‐11.18 to 30.18). The results are of very low certainty due to serious imprecision and risk of bias (Analysis 11.2; Table 12).

###### Endoscopic improvement

There were no data for this outcome.

###### Withdrawals due to adverse events

We cannot draw any conclusions on the effects of a four‐food elimination diet with omeprazole (n = 5/32) on withdrawals due to adverse events compared to omeprazole (n = 1/32) (RR 5.00, 95% CI 0.62 to 40.44). The results are of very low certainty due to serious imprecision and risk of bias (Analysis 11.3; Table 12).

##### Secondary outcomes

There were no data for any of the secondary outcomes.

#### Four‐food elimination diet and amino acid formula versus four‐food elimination diet

One study compared a four‐food elimination diet with amino acid formula to a four‐food elimination diet for induction of remission (De Rooij 2022).

##### Primary outcomes

###### Clinical improvement

We cannot draw any conclusions on the effects of a four‐food elimination diet with amino acid formula (n = 21) on clinical improvement compared to a four‐food elimination diet (n = 20), measured as a continuous outcome (MD ‐0.50, 95% CI ‐2.41 to 1.41). The results are of very low certainty due to serious imprecision and risk of bias (Analysis 12.1; Table 13).

###### Histological improvement

We cannot draw any conclusions on the effects of a four‐food elimination diet with amino acid formula (n = 10/21) on histological improvement compared to a four‐food elimination diet (n = 5/20), measured as a dichotomous outcome (RR 1.90, 95% CI 0.79 to 4.60). The results are of very low certainty due to serious imprecision and risk of bias (Analysis 12.2; Table 13).

We cannot draw any conclusions on the effects of a four‐food elimination diet with amino acid formula (n = 21) on histological improvement compared to a four‐food elimination diet (n = 20), measured as a continuous outcome (MD 13.80, 95% CI ‐9.50 to 37.10). The results are of very low certainty due to serious imprecision and risk of bias (Analysis 12.3; Table 13).

###### Endoscopic improvement

We cannot draw any conclusions on the effect of a four‐food elimination diet with amino acid formula (n = 21) on endoscopic improvement compared to a four‐food elimination diet (n = 20), measured as a continuous outcome (MD ‐1.00, 95% CI ‐2.83 to 0.83). The results are of very low certainty due to serious imprecision and risk of bias (Analysis 12.4; Table 13).

###### Withdrawals due to adverse events

We cannot draw any conclusions on the effects of a four‐food elimination diet with amino‐acid formula (n = 1/21) compared to a four‐food elimination diet (n = 1/20) on withdrawals due to adverse events (RR 0.95, 95% CI 0.06 to 14.22). The results are of very low certainty due to serious imprecision and risk of bias (Analysis 12.5; Table 13).

##### Secondary outcomes

###### Serious adverse events

We cannot draw any conclusions on the effects of a four‐food elimination diet with amino‐acid formula (n = 0/21) compared to a four‐food elimination diet (n = 0/20) on serious adverse events (effect not estimable; Analysis 12.6). The results are of very low certainty due to serious imprecision and risk of bias.

###### Total adverse events

We cannot draw any conclusions on the effects of a four‐food elimination diet with amino‐acid formula (n = 1/21) compared to a four‐food elimination diet (n = 0/20) on total adverse events (RR 2.86, 95% CI 0.12 to 66.44). The results are of very low certainty due to serious imprecision and risk of bias (Analysis 12.7).

###### Quality of life

There were no data for this outcome.

#### Nebulized budesonide versus viscous budesonide

One study compared nebulized swallowed budesonide to viscous swallowed budesonide for induction of remission (Dellon 2012).

##### Primary outcomes

###### Clinical improvement

We cannot draw any conclusions on the effects of nebulized swallowed budesonide (n = 11) on clinical improvement compared to viscous swallowed budesonide (n = 11), measured as a continuous outcome (MD ‐6.00, 95% CI ‐18.30 to 6.30). The results are of very low certainty due to serious imprecision and risk of bias (Analysis 13.1; Table 14).

###### Histological improvement

We cannot draw any conclusions on the effects of nebulized swallowed budesonide (n = 11) on histological improvement compared to viscous swallowed budesonide (n = 11), measured as a continuous outcome (MD 78.00, 95% CI 20.81 to 135.19). The results are of very low certainty due to serious imprecision and risk of bias (Analysis 13.2; Table 14).

###### Endoscopic improvement

There were no data for this outcome.

###### Withdrawals due to adverse events

We cannot draw any conclusions on the effects of nebulized swallowed budesonide (n = 0/13) compared to viscous swallowed budesonide (n = 0/12) on withdrawals due to adverse events (effect not estimable). The results are of very low certainty due to serious imprecision and risk of bias (Table 13).

##### Secondary outcomes

###### Serious adverse events

We cannot draw any conclusions on the effects of nebulized swallowed budesonide (n = 0/13) compared to viscous swallowed budesonide (n = 0/12) on serious adverse events (effect not estimable; Analysis 13.3). The results are of very low certainty due to serious imprecision and risk of bias.

###### Total adverse events

We cannot draw any conclusions on the effects of nebulized swallowed budesonide (n = 2/13) compared to viscous swallowed budesonide (n = 2/12) on total adverse events (RR 0.92, 95% CI 0.15 to 5.56; Analysis 13.4). The results are of very low certainty due to serious imprecision and risk of bias.

###### Quality of life

There were no data for this outcome.

#### Viaskin milk patch versus placebo

One study compared Viaskin milk patch to placebo for induction of remission (Spergel 2020).

##### Primary outcomes

###### Clinical improvement

Viaskin milk patch (n = 7) may result in little to no difference in clinical improvement compared to placebo (n = 2), measured as a continuous outcome (MD 1.29, 95% CI ‐0.83 to 3.41). The results are of low certainty due to serious imprecision (Analysis 14.1; Table 15).

###### Histological improvement

Viaskin milk patch (n = 7) may result in little to no difference in histological improvement compared to placebo (n = 2), measured as a continuous outcome (MD 69.43, 95% CI ‐21.75 to 160.61). The results are of low certainty due to serious imprecision (Analysis 14.2; Table 15).

###### Endoscopic improvement

Viaskin milk patch (n = 15) may result in little to no difference in endoscopic improvement compared to placebo (n = 5), measured as a continuous outcome (MD ‐0.33, 95% CI ‐2.00 to 1.34). The results are of low certainty due to serious imprecision (Analysis 14.3; Table 15).

###### Withdrawals due to adverse events

Viaskin milk patch (n = 1/15) may result in little to no difference in withdrawals due to adverse events compared to placebo (n = 0/5) (RR 1.12, 95% CI 0.05 to 23.99). The results are of low certainty due to serious imprecision (Analysis 14.4; Table 15).

##### Secondary outcomes

###### Serious adverse events

Viaskin milk patch (n = 0/15) may result in little to no difference in serious adverse events compared to placebo (n = 1/5) (RR 0.13, 95% CI 0.01 to 2.67; Analysis 14.5). The results are of low certainty due to serious imprecision.

###### Total adverse events

Viaskin milk patch (n = 15/15) may result in little to no difference in total adverse events compared to placebo (n = 5/5) (RR 1.00, 95% CI 0.77 to 1.29; Analysis 14.6). The results are of low certainty due to serious imprecision.

###### Quality of life

Viaskin milk patch (n = 7) may result in little to no difference in quality of life compared to placebo (n = 2), measured as a continuous outcome (MD 13.60, 95% CI ‐16.12 to 43.32; Analysis 14.7). The results are of low certainty due to serious imprecision.

#### Leukotriene receptor antagonist versus placebo for maintenance of remission

One study compared leukotriene receptor antagonist to placebo for maintenance of remission (Alexander 2017).

##### Primary outcomes

###### Clinical improvement

We cannot draw any conclusions on the effects of leukotriene receptor antagonist (n = 8/20) compared to placebo (n = 5/21) on clinical improvement for maintenance of remission, measured as a dichotomous outcome (RR 1.68, 95% CI 0.66 to 4.28). The results are of very low certainty due to serious imprecision and risk of bias (Analysis 15.1; Table 16).

###### Histological improvement

There were no data for meta‐analysis for this outcome.

###### Endoscopic improvement

There were no data for meta‐analysis for this outcome.

###### Withdrawals due to adverse events

We cannot draw any conclusions on the effects of leukotriene receptor antagonist (n = 2/20) on withdrawals due to adverse events compared to placebo (n = 1/21) for maintenance of remission (RR 2.10, 95% CI 0.21 to 21.39). The results are of very low certainty due to serious imprecision and risk of bias (Analysis 15.2; Table 16).

##### Secondary outcomes

###### Serious adverse events

We cannot draw any conclusions on the effects of leukotriene receptor antagonist (n = 0/20) on serious adverse events compared to placebo (n = 0/21) for maintenance of remission (effect not estimable; Analysis 15.3). The results are of very low certainty due to serious imprecision and risk of bias.

###### Total adverse events

We cannot draw any conclusions on the effects of leukotriene receptor antagonist (n = 0/20) on total adverse events compared to placebo (n = 0/21) for maintenance of remission (effect not estimable; Analysis 15.4). The results are of very low certainty due to serious imprecision and risk of bias.

###### Quality of life

There were no data for this outcome.

#### Mepolizumab high‐dose (10 mg/kg) versus low‐dose (0.55 mg/kg)

One study compared a low dose of the biologic mepolizumab (0.55 mg/kg) to a medium dose (2.5 mg/kg) and to a high dose (10 mg/kg) (Assa'ad 2011). The study did not include any control groups other than mepolizumab.

##### Primary outcomes

###### Clinical improvement

There were no data for this outcome.

###### Histological improvement

We cannot draw any conclusions about the effects of mepolizumab 10 mg/kg (n = 5/20) compared to mepolizumab 0.55 mg/kg (n = 4/19) on histological improvement, measured as a dichotomous outcome (RR 1.19, 95% CI 0.37 to 3.77). The results are of very low certainty due to serious imprecision and risk of bias (Analysis 16.1; Table 17).

###### Endoscopic improvement

There were no data for this outcome.

###### Withdrawals due to adverse events

We cannot draw any conclusions about the effects of mepolizumab 10 mg/kg (n = 2/20) compared to mepolizumab 0.55 mg/kg (n = 3/19) on withdrawals due to adverse events (RR 0.63, 95% CI 0.12 to 3.38). The results are of very low certainty due to serious imprecision and risk of bias (Analysis 16.2; Table 17).

##### Secondary outcomes

###### Serious adverse events

The study reported three serious adverse events but did not specify in which group they occurred. No conclusions can be drawn. The results are of very low certainty due to serious risk of bias and imprecision.

###### Total adverse events

The study reported a total of 51/59 participants with more than one adverse event, but did not give specifics per group. The results are of very low certainty due to serious risk of bias and imprecision.

###### Quality of life

There were no data for this outcome.

#### Mepolizumab medium‐dose (2.5 mg/kg) versus low‐dose (0.55 mg/kg)

One study compared a low dose of the biologic mepolizumab (0.55 mg/kg) to a medium dose (2.5 mg/kg) and to a high dose (10 mg/kg) (Assa'ad 2011). The study did not include any control groups other than mepolizumab.

##### Primary outcomes

###### Clinical improvement

There were no data for this outcome.

###### Histological improvement

We cannot draw any conclusions about the effects of mepolizumab 2.5 mg/kg (n = 9/20) compared to mepolizumab 0.55 mg/kg (n = 4/19) on histological improvement, measured as a dichotomous outcome (RR 2.14, 95% CI 0.79 to 5.79). The results are of very low certainty due to serious imprecision and risk of bias (Analysis 17.1; Table 18).

###### Endoscopic improvement

There were no data for this outcome.

###### Withdrawals due to adverse events

We cannot draw any conclusions about the effects of mepolizumab 2.5 mg/kg (n = 1/20) compared to mepolizumab 0.55 mg/kg (n = 3/19) on withdrawals due to adverse events (RR 0.32, 95% CI 0.04 to 2.79). The results are of very low certainty due to serious imprecision and risk of bias (Analysis 17.2; Table 18).

##### Secondary outcomes

###### Serious adverse events

The study reported three serious adverse events but did not specify in which group they occurred. No conclusions can be drawn. The results are of very low certainty due to serious risk of bias and imprecision.

###### Total adverse events

The study reported a total of 51/59 participants with more than one adverse event, but did not give specifics per group. The results are of very low certainty due to serious risk of bias and imprecision.

###### Quality of life

There were no data for this outcome.

#### Mepolizumab high‐dose (10 mg/kg) versus medium‐dose (2.5 mg/kg)

One study compared a low dose of the biologic mepolizumab (0.55 mg/kg) to a medium dose (2.5 mg/kg) and to a high dose (10 mg/kg) (Assa'ad 2011). The study did not include any control groups other than mepolizumab.

##### Primary outcomes

###### Clinical improvement

There were no data for this outcome.

###### Histological improvement

We cannot draw any conclusions about the effects of mepolizumab 10 mg/kg (n = 5/20) compared to mepolizumab 2.5 mg/kg (n = 9/20) on histological improvement, measured as a dichotomous outcome (RR 0.56, 95% CI 0.23 to 1.37). The results are of very low certainty due to serious imprecision and risk of bias (Analysis 18.1; Table 19).

###### Endoscopic improvement

There were no data for this outcome.

###### Withdrawals due to adverse events

We cannot draw any conclusions about the effects of mepolizumab 10 mg/kg (n = 2/20) compared to mepolizumab 2.5 mg/kg (n = 1/20) on withdrawals due to adverse events (RR 2.00, 95% CI 0.20 to 20.33). The results are of very low certainty due to serious imprecision and risk of bias (Analysis 18.2; Table 19).

##### Secondary outcomes

###### Serious adverse events

The study reported three serious adverse events but did not specify in which group they occurred. No conclusions can be drawn. The results are of very low certainty due to serious risk of bias and imprecision.

###### Total adverse events

The study reported a total of 51/59 participants with more than one adverse event, but did not give specifics per group. The results are of very low certainty due to serious risk of bias and imprecision.

###### Quality of life

There were no data for this outcome.

#### Six‐food elimination diet versus swallowed fluticasone versus swallowed budesonide versus oral viscous budesonide

One study compared a six‐food elimination diet to swallowed fluticasone to swallowed budesonide and to oral viscous budesonide (Oliva 2018). This was an abstract report without any data that could be used for meta‐analysis of any outcome.

##### Primary outcomes

###### Clinical improvement

There were no data for this outcome.

###### Histological improvement

The study only reported percentages, with the numbers of patients in each group not reported. At eight weeks, 69% of participants in the six‐food elimination diet group achieved histological improvement, 67% in the swallowed fluticasone group, 75% in the swallowed budesonide group, and 85% in the oral viscous budesonide group. No conclusions can be drawn. These results are of very low certainty due to serious risk of bias and imprecision.

###### Endoscopic improvement

There were no data for this outcome.

###### Withdrawals due to adverse events

There were no data for this outcome.

##### Secondary outcomes

There were no data for any of the secondary outcomes.

## Discussion

### Summary of main results

In the studies comparing corticosteroids and placebo for induction, corticosteroids may lead to clinical symptom improvement when reported as both dichotomous and continuous outcomes. Corticosteroids lead to a large increase in histological improvement (dichotomous outcome) and may increase histological improvement (continuous outcome) when compared to placebo. Corticosteroids may increase endoscopic improvement (dichotomous and continuous outcome). Withdrawals due to adverse events as a dichotomous outcome may be lower for corticosteroids when compared to placebo.

In the studies comparing corticosteroids and placebo for maintenance, corticosteroids probably lead to a large increase in histological improvement (dichotomous outcome) and probably increase histological improvement (continuous outcome) when compared to placebo. No conclusions can be drawn for any other outcomes due to very low‐certainty evidence.

In the studies comparing biologics and placebo, biologics may lead to little to no clinical symptom improvement when reported as a dichotomous outcome, and may lead to an increase in clinical symptom improvement when reported as a continuous outcome. Biologics may result in increased histological improvement when reported as a dichotomous outcome, but this is uncertain when reported as a continuous outcome when compared to placebo. Biologics may increase endoscopic improvement when measured as a dichotomous outcome, but this is uncertain when measured as a continuous outcome. Withdrawals due to adverse events as a dichotomous outcome may occur as frequently when biologics are compared to placebo.

In the study comparing cromolyn sodium to placebo, cromolyn sodium may lead to clinical symptom and histological improvement, measured as a continuous outcome. There may be no difference between cromolyn sodium and placebo in withdrawals due to adverse events and serious adverse events. Other outcomes were not reported.

In the study comparing PGD2R antagonist to placebo, we could not reach any conclusions for any primary outcome or serious adverse events due to the very low certainty of the results. Other secondary outcomes were not reported.

In the study comparing swallowed fluticasone to oral prednisone, we could not reach any conclusions for any primary outcome, or serious or total adverse events, due to the very low certainty of the results. Quality of life was not reported.

In the study comparing oral viscous budesonide to swallowed fluticasone there may be little to no difference for all primary outcomes, or serious and total adverse events. Quality of life was not reported.

In the two studies comparing esomeprazole to fluticasone we cannot draw any conclusions for clinical and histological improvement, withdrawals due to adverse events, or serious and total adverse events, due to the very low certainty of the results. Endoscopic improvement and quality of life were not reported.

In the study comparing a one‐food elimination diet to a four‐food elimination diet we cannot draw any conclusions for any outcome, due to the very low certainty of the results.

In the study comparing a one‐food elimination diet to a six‐food elimination diet we cannot draw any conclusions for any outcome, due to the very low certainty of the results.

In the study comparing a four‐food elimination diet with omeprazole to omeprazole we cannot draw any conclusions for histological improvement or withdrawals due to adverse events, due to the very low certainty of the results. No other outcomes were reported.

In the study comparing a four‐food elimination diet with amino acid formula to a four‐food elimination diet, we could not reach any conclusions for endoscopic or histological improvement, or serious or total adverse events, due to the very low certainty of the results. Clinical improvement and quality of life were not reported.

In the study comparing nebulized swallowed budesonide to viscous swallowed budesonide we cannot draw any conclusions for clinical and histological improvement, withdrawals due to adverse events, or serious and total adverse events, due to the very low certainty of the results. Endoscopic improvement and quality of life were not reported.

In the study comparing Viaskin milk patch to placebo, Viaskin milk patch may result in little to no difference for all outcomes.

In the study comparing leukotriene receptor antagonist to placebo for maintenance of remission, we cannot draw any conclusions for clinical improvement, withdrawals due to adverse events, or serious and total adverse events, due to the very low certainty of the results. Other outcomes were not reported.

In the study comparing a low dose of the biologic mepolizumab (0.55 mg/kg) to a medium dose (2.5 mg/kg) and to a high dose (10 mg/kg), we cannot draw any conclusions for all combinations of comparisons, for histological improvement, withdrawals due to adverse events, or serious and total adverse events, due to the very low certainty of the results. Other outcomes were not reported.

In the study comparing a six‐food elimination diet to swallowed fluticasone to swallowed budesonide and to oral viscous budesonide, no meta‐analyses were possible for any combination of comparisons. We cannot draw any conclusions for histological improvement due to the very low certainty of the results. Other outcomes were not reported.

### Overall completeness and applicability of evidence

In a number of our primary outcomes in key groups of treatments, high‐ and moderate‐certainty evidence has been synthesized. However, there are several issues with the overall evidence from the included studies, which limits our ability to draw convergent conclusions to inform clinical practice and decision‐making.

There are limited studies specifically in children, often with high clinical and methodological heterogeneity. Most studies used mixed groups of adolescents and adults, but the data were combined. This not only limits the applicability to younger children, but it limits the completeness of the evidence in the adolescent age group.

The first primary outcome was clinical symptom improvement as defined by the individual study, but the assessments varied widely, with a lack of consistent validated, patient‐reported outcomes. This limited the scope for meta‐analysis and in turn reduced certainty, particularly in the pediatric setting. The limited data set in children makes it difficult to determine whether a lack of efficacy for these outcomes reflects issues with different symptomatology, the validity and reliability of such measures or instruments in children, or underlying poor efficacy. A similar point can be made about the adult studies. This is a key clinical question across all patient ages and so limits the applicability of these outcomes. Ideally, a single validated clinical outcome would be used across studies in order to facilitate data pooling and comparison of results.

There were also a range of therapies used within each of the two main groups seen, corticosteroids and biologics. The subgroup analysis does suggest that certain specific subclasses of medications within each of these groups have higher efficacy on a range of outcomes, but as the numbers of studies reduces substantially in these analyses, the certainty of the evidence is also lower. This is another key clinical area and until further targeted studies increase certainty, it represents an area of the evidence that is incomplete.

Histologic outcomes were also heterogenous, with different scoring systems, different thresholds, and the use of both dichotomous and continuous outcomes. This limits the scope for analysis and as such the certainty of evidence. Debate remains in the field as to which dichotomous histologic threshold is the preferred outcome and there may be differences in use of such a threshold. There is good but not universal agreement on a cut‐off threshold in clinical practice (i.e. < 15 eos/hpf in an appropriate clinical setting) or per FDA guidance (≤ 6 eos/hpf), or if so‐called "deep remission" (≤ 1 eos/hpf) is desired. Additionally, while the eosinophil count has traditionally been the biomarker of interest for response assessment in clinical practice and trials, and is also the most visually evident and readily available one to use, there is growing recognition that it may not fully reflect the underlying disease process. As such, the EoE Histologic Scoring System (EoE‐HSS; Collins 2017) has been used in some recent trials as a secondary or exploratory endpoint, but could not be included as an outcome in the present study. Future iterations of this review will hopefully be able to assess this outcome in more detail.

Reporting of endoscopic measures was less common and had similar impacts, limiting the completeness of evidence in this area. Quality of life was sparsely reported and represents another incomplete area of evidence.

The prior use of proton pump inhibitor (PPI) therapy before recruitment and the concomitant use of such therapy varied between studies. This clinical and methodological source of heterogeneity reduces the applicability of the findings.

This is a rapidly moving field with study designs changing constantly, moving targets for outcome measures, concomitant PPI use, and thresholds of treatment success. For many current studies prior PPI failure is no longer necessary at study inclusion. Issues like these further increase heterogeneity and reduce applicability.

The reporting of adverse events was inconsistent across the included studies, with a lack of uniformity on what constitutes an adverse event and a serious adverse event. While the outcome of withdrawal is a much more objective measure in this context, it can be argued that this is of less interest to patients and therefore does limit the application of these data.

Finally, the complex clinical and methodological heterogeneity issues limit the scope for meaningful subgroup analysis of key factors, such as gender, age, dosage, and type of corticosteroid or biologic, and extent of disease, ultimately limiting the completeness of the evidence.

### Quality of the evidence

The certainty of the evidence for a number of primary outcomes comparing corticosteroids and biologics to placebo was high and moderate, but most other outcomes and comparisons were of low certainty, primarily due to inconsistency and issues with risk of bias. A number of the inconsistency issues were explained in subgroup analysis as due to age (corticosteroids) and mechanism of agent (biologics), but these investigations in turn increased imprecision due to smaller participant numbers.

Imprecision was seen throughout due to the pervasive issues with heterogeneity in the specific outcomes measures used, as already discussed above. Whilst the homogeneous deployment of outcome measures would increase the scope for analysis and in turn reduce issues with imprecision, there were a number of studies with very low participant numbers. We have previously published work highlighting how common this type of difficulty in reporting sample size estimation is (Iheozor‐Ejiofor 2021), with a need for adequate sample size calculations using published resources (Gordon 2021).

Risk of bias within the primary studies was low for all judged criteria in a little under half of the studies, with the remainder exhibiting issues in a number of the areas.

### Potential biases in the review process

Gaps in information to judge risk of bias were pervasive, as discussed above. Given the contemporaneous nature of the evidence, with an exponential increase in studies since the last published version of this review, the review team considered it prudent and likely fruitful to reach out to primary authors to request clarification or additional information. Many did respond and as such judgments could be changed from those we made based on the published study reports, this could be considered a source of bias. Conversely, for those where no response was received, the judgments are based on the published forms of the studies.

The team aimed to include data that may become available in future updates, but this could represent a source of bias in the review, with 23 ongoing studies identified in the review process. Conversely, the use of such unpublished data can also be seen as a source of bias.

We are aware of the possibility that industry funding may affect the validity of the results. Funding from manufacturing companies or any conflicts of interests from both the primary studies and the review team have been reported.

For the main comparisons within this review, there were potential biases within the extraction and analysis process. When multiple outcomes were reported, we made decisions to report those that were validated and homogenous, but this may have introduced bias. There were a number of circumstances when calculations were needed to convert measures such as standard error to standard deviation or to convert units for histological outcomes. These have all been reported within the review, but could introduce bias.

Finally, the significant clinical heterogeneity remained a challenge when using our pre‐planned subgroups. We made consensus decisions on the classification of particular subgroup characteristics, but again this is a potential source of bias.

There was repeated heterogeneity in clinical, histologic, and endoscopic outcomes. Additionally, subgroups of children compared to adults, co‐therapy especially with proton pump inhibitors, differences in drug delivery, differences in dosage, differences in frequency, and differences in mechanism were all identified as potential biases.

With varying and occasionally multiple histological thresholds reported in the studies, the analysis did not contain a single dichotomous histological threshold. To minimize bias and subjectivity, we elected to include all histological thresholds with most common being < 15, ≤ 6, and ≤ 1. In addition, we performed sensitivity analysis amongst the various histological thresholds, but the decision to include all such thresholds in a primary analysis could be considered source of bias.

### Agreements and disagreements with other studies or reviews

In 2010, a previous version of this review was published with only three randomized controlled trials (RCTs). As of 2022, the number of published RCTs for pediatric and adult eosinophilic esophagitis that meet our inclusion criteria has grown to 41, with approximately half completed in the past five years. Given the rapid pace of clinical trial publications for eosinophilic esophagitis and changing outcome metrics, prior publications appear outdated, even those as recent as 2020. As an example, a recent American Gastroenterological Association (AGA) technical review of eosinophilic esophagitis management reported nine corticosteroid trials (compared to 14) and four biologic clinical trials (compared to nine) (Rank 2020). Another example is the European guidelines, which included even fewer RCTs as they were published three years earlier (Lucendo 2017). The AGA technical review clearly employed GRADE and when RCTs were synthesized, the broad findings were similar, although the certainty broadly increased in our review in key highly studied areas (biologics and non‐systemic steroids) due to enhanced quality of reporting and precision (Rank 2020).

A recent UK joint adult and pediatric national consensus guideline, which considered a similar body of evidence, did state alignment with GRADE methodology (Dhar 2022). However, no technical review details are presented and in several key areas of management, the GRADE of evidence included in this review does not match what is reported. For example, several forms of diet therapy with different comparators are noted to have moderate or low GRADE certainty of evidence in their guideline. However, all of them are very low‐certainty GRADE outcomes in our review for all primary outcome measures. It is difficult to explain this difference as the detailed technical review information is missing.

The focus of prior reviews has been primarily on histologic outcomes and adverse events. Governmental pharmaceutical regulatory agencies have emphasized that outcomes for eosinophilic esophagitis must focus on patient‐reported symptom outcomes and the newer assessments of esophageal function. This current Cochrane Review includes dichotomous and continuous outcomes for symptom, histologic, and endoscopic outcomes.

## Authors' conclusions

**Implications for practice**

The evidence from this review demonstrates that for induction of remission, corticosteroids improve histologic outcomes (high certainty) and that biologic anti‐IL‐13 and anti‐IL‐4r therapies may improve clinical outcomes (low to moderate certainty). Eleven studies included children up to 18 years and 30 studies included adolescents and adults.

Corticosteroid therapy compared to placebo may lead to slightly better clinical improvement, as a dichotomous outcome (low certainty), leads to a large histological improvement (high certainty, number needed to treat for an additional beneficial outcome (NNTB) = 3), and may lead to fewer adverse event withdrawals (low certainty). Biologic anti‐IL‐5 therapy may result in little to no difference in clinical improvement (low certainty), and may lead to slightly better histological improvement (low certainty). Anti‐IL‐13 and anti‐IL‐4r therapy may lead to slightly better clinical improvement (low certainty) and may result in better histological improvement (moderate certainty, NNTB = 3). For anti‐sialic acid binding Ig‐like lectin 8 therapy, compared to placebo, clinical outcomes could not be reported due to incomplete published data; for histologic dichotomous outcomes anti‐sialic acid binding Ig‐like lectin 8 therapy may lead to slightly better improvement (low certainty). For anti‐IgE compared to placebo, no conclusions can be made regarding clinical improvement (very low certainty). In studies comparing cromolyn sodium or Viaskin milk patch to placebo there may be no difference in clinical improvement, histologic improvement, or adverse event withdrawals (low certainty).

We could not draw conclusions about clinical improvement, histological improvement, or adverse event outcomes for the following: active comparator studies with PGD2R antagonist OC000459 versus placebo, esomeprazole versus fluticasone, swallowed fluticasone versus oral prednisone, nebulized swallowed budesonide versus swallowed viscous budesonide, oral viscous budesonide versus swallowed fluticasone, anti‐IL‐5 (10 mg/kg versus 0.55 mg/kg), anti‐IL‐5 (2.5 mg/kg versus 0.55 mg/kg), anti‐IL‐5 (10 mg/kg versus 2.5 mg/kg), a one‐food elimination diet versus a four‐food elimination diet, a one‐food elimination diet versus a six‐food elimination diet, or a four‐food elimination diet with an amino acid formula versus a four‐food elimination diet (all low‐ or very low‐certainty evidence).

The evidence from this review demonstrates that for maintenance of remission, corticosteroids probably result in histological improvement (moderate certainty), but no other conclusions can be drawn (very low certainty). No conclusions can be made regarding leukotriene receptor antagonists in achieving maintenance of remission (very low certainty).

There were no clinical trials that compared either proton pump inhibitor (PPI) medication or dietary elimination therapies to a placebo for induction or maintenance of remission.

**Implications for research**

As heterogeneity in reporting of outcomes and the thresholds for specific outcomes was pervasive in the evidence base, the use of validated tools and standardized thresholds for success is key for future research. These should align with regulatory guidelines that employ such defined and publicly available homogenous outcomes in all key areas, including histologic outcome systems (Collins 2017), validated patient‐reported outcomes (PROs) of symptoms and health‐related quality of life (Hudgens 2017), validated endoscopic outcomes (Ma 2022a), and validated assessments of esophageal function (e.g. esophageal distensibility testing; Donnan 2020). This could build on initial work done by the COREOS group, which proposed an initial consensus core outcomes set for eosinophilic esophagitis (Ma 2022b). Clinical outcome results for children are more heterogenous than adults, and pediatric patient‐reported outcomes are an important area for future research and development. Studies should follow clear reporting guidelines in line with the CONSORT statement to reduce the risk of bias and enhance the certainty of the evidence base as a whole, regardless of findings.

There is a clear direction for future research of head‐to‐head comparisons of corticosteroids and biologics. Randomized, placebo‐controlled clinical trials for proton pump inhibitor medications and dietary elimination are also needed. Future direction can also include personalized medicine clinical trials with precision medicine‐focused therapies compared to conventional therapies.

In accordance with the findings of other recently published reviews, clinical guidelines, and regulatory statements within eosinophilic esophagitis, large‐scale, long‐term measurement of outcomes to include safety and adverse events is needed.

## What's new

**Table:** 

**Date**	**Event**	**Description**
20 July 2023	New search has been performed	The number of included studies has increased from 3 to 41, and we have updated the methodology to modern standards. Consequently, the results, discussion, and conclusions have changed considerably since this review was last published. None of the authors of the previously published version are authors on the update (EEJ, TD, MJE). All authors of the update are first‐time authors on this review. There have been adjustments in the primary and secondary outcomes, as well as the pre‐planned subgroup and sensitivity analyses, since the previous version, reflecting current knowledge on eosinophilic esophagitis. We were not able to perform any pre‐planned subgroup or sensitivity analyses for any of the comparisons presented in summary of findings table 2 and tables 4 to 10 due to very limited data. Any pre‐planned subgroup and sensitivity analyses not listed under analyses for the comparisons corticosteroids versus placebo for induction of remission, and biologics versus placebo for induction of remission, were also not performed due to lack of data. Funnel plots to judge publication bias were only possible in one instance (Figure 3), as in all other cases there was not a sufficient number of studies (> 10).
20 July 2023	New citation required and conclusions have changed	**Initial version of review** The initial version of this review, published on 17 March 2010, identified three studies that met the inclusion criteria (Konikoff 2006; Schaefer 2008; Straumann 2010a). These studies examined the efficacy of fluticasone propionate (N = 20, 400 µg twice‐daily for 3 months) via metered dose inhaler and swallowed versus placebo (N = 11), oral prednisone (N = 32, 1 mg/kg/dose twice‐daily for four weeks) versus topical (swallowed) fluticasone via metered dose inhaler (N =36, 110 µg per puff (age 1 to 10 years) and 22 µg per puff (> 11 years of age)), and a dose escalation pilot study of mepolizumab compared to placebo (N = 11), respectively. The total number of included participants was 127. The authors concluded the following: Comparing topical fluticasone with placebo, fluticasone decreased vomiting more than placebo (67% versus (versus) 27%, P < 0.05) but did not improve dysphagia. Histological remission was reported in the fluticasone group compared with the placebo group (50% versus 9%, P = 0.05; RR 5.5, 95% CI 0.81 to 37.49). Comparing fluticasone with oral prednisone, symptom resolution and improvement of esophagitis were similar, with the majority of participants being symptom‐free at four weeks, with no difference between groups (RR 1.03, 95% CI 0.95 to 1.11). Comparing mepolizumab to placebo, there was no difference in symptom response with mepolizumab compared to placebo, but the decrease in esophageal eosinophil count was greater with mepolizumab than placebo (67% versus 25%). **Current version of review** The current version of this review includes an additional 38 studies, which increases the number of analyses from 3 to 18 and increases the total number of participants to 3253. Regarding the conclusions that have changed from the initial review to the present, an additional four trials compared fluticasone versus placebo for histological remission (Alexander 2012; Butz 2014; Dellon 2022a; Hirano 2020f). These included a total of 250 participants and together confirm and reinforce the original conclusion that fluticasone is effective for treatment of EoE as measured by histological dichotomous remission (68% versus 6%, P < 0.00001; RR 7.57, 95% CI 3.36 to 17.08; I^2^ = 0%). There were no new studies that compared fluticasone with oral prednisone or mepolizumab to placebo, so the results from these analyses did not change in the current review. In addition to these changes in the results, the current version of the review includes 14 new classes of analyses: Corticosteroids versus placebo for induction of remission Corticosteroids versus placebo for maintenance of remission Biologics versus placebo for induction of remission Cromolyn sodium versus placebo PGD2R antagonist OC000459 versus placebo Oral viscous budesonide versus swallowed fluticasone Esomeprazole versus fluticasone One‐food elimination diet versus four‐food elimination diet One‐food elimination diet versus six‐food elimination diet Four‐food elimination diet with omeprazole versus omeprazole Four‐food elimination diet with amino acid formula versus four‐food elimination diet Nebulized budesonide versus viscous budesonide Viaskin milk patch versus placebo Leukotriene receptor antagonist versus placebo for maintenance of remission Each class of analysis examines, as primary outcomes, clinical (continuous, dichotomous), histological (continuous, dichotomous), and endoscopic (continuous, dichotomous) improvement, and withdrawals due to adverse events (dichotomous), and as secondary outcomes, serious adverse events (dichotomous), total adverse events (dichotomous), and quality of life (continuous, dichotomous), at end of trial when prespecified by the study authors. The sensitivity analyses conducted include analyses based on fixed‐effect model, eos/hpf threshold, validated instruments, peer‐reviewed publications, and less than high risk of bias, as appropriate. The subgroup analyses conducted include age group, type of steroid (TCSs only), delivery method (TCSs only), and mechanism (biologics only), as appropriate.

## History

Protocol first published: Issue 1, 2003 Review first published: Issue 3, 2004

**Table:** 

**Date**	**Event**	**Description**
27 January 2010	New search has been performed	New studies added, review updated.
27 January 2010	New citation required but conclusions have not changed	Author line changed.
30 October 2008	Amended	Converted to new review format.
8 May 2006	New search has been performed	Minor update.
2 February 2006	Amended	New studies sought but none found.
15 April 2004	New citation required and conclusions have changed	Substantive amendment.

## Appendices

### Appendix 1. CENTRAL via Cochrane Library search strategy

Date Run: 04/03/2023 01:04:38

#1 [mh "Eosinophilic Esophagitis"] OR ([mh Esophagitis] AND [mh Eosinophilia]) OR ((Eosinophil* OR Eosinophyl*) AND (Esophag* OR Oesophag*)) with Cochrane Library publication date Between Oct 2021 and Mar 2023, in Trials 94

### Appendix 2. MEDLINE via Ovid SP search strategy

Database: Ovid MEDLINE(R) ALL <1946 to March 02, 2023>

1 ((Randomized Controlled Trial or Controlled Clinical Trial).pt. or (Randomi?ed or Placebo or Randomly or Trial or Groups).ab. or Drug Therapy.fs.) not (exp Animals/ not Humans.sh.) (4899157)

2 Eosinophilic Esophagitis/ or (Esophagitis/ and Eosinophilia/) or ((Eosinophil* or Eosinophyl*) and (Esophag* or Oesophag*)).tw,kw. (4480)

3 1 and 2 (1182)

4 limit 3 to ed=20211023‐20230303 (173)

5 limit 3 to dt=20211023‐20230303 (121)

6 4 or 5 (194)

### Appendix 3. Embase via Ovid SP search strategy

Database: Embase <1974 to 2023 Week 08>

1 Randomized controlled trial/ or Controlled clinical study/ or randomization/ or intermethod comparison/ or double blind procedure/ or human experiment/ or (random$ or placebo or (open adj label) or ((double or single or doubly or singly) adj (blind or blinded or blindly)) or parallel group$1 or crossover or cross over or ((assign$ or match or matched or allocation) adj5 (alternate or group$1 or intervention$1 or patient$1 or subject$1 or participant$1)) or assigned or allocated or (controlled adj7 (study or design or trial)) or volunteer or volunteers).ti,ab. or (compare or compared or comparison or trial).ti. or ((evaluated or evaluate or evaluating or assessed or assess) and (compare or compared or comparing or comparison)).ab. (6208790)

2 (random$ adj sampl$ adj7 ("cross section$" or questionnaire$1 or survey$ or database$1)).ti,ab. not (comparative study/ or controlled study/ or randomi?ed controlled.ti,ab. or randomly assigned.ti,ab.) (9365)

3 Cross‐sectional study/ not (randomized controlled trial/ or controlled clinical study/ or controlled study/ or (randomi?ed controlled or control group$1).ti,ab.) (338867)

4 (((case adj control$) and random$) not randomi?ed controlled).ti,ab. (21248)

5 (Systematic review not (trial or study)).ti. (251119)

6 (nonrandom$ not random$).ti,ab. (18726)

7 ("Random field$" or (random cluster adj3 sampl$)).ti,ab. (4426)

8 (review.ab. and review.pt.) not trial.ti. (1090581)

9 "we searched".ab. and (review.ti. or review.pt.) (48360)

10 ("update review" or (databases adj4 searched)).ab. (60294)

11 (rat or rats or mouse or mice or swine or porcine or murine or sheep or lambs or pigs or piglets or rabbit or rabbits or cat or cats or dog or dogs or cattle or bovine or monkey or monkeys or trout or marmoset$1).ti. and animal experiment/ (1214071)

12 Animal experiment/ not (human experiment/ or human/) (2549714)

13 or/2‐12 (4261110)

14 1 not 13 (5483547)

15 Eosinophilic Esophagitis/ or (Esophagitis/ and (Eosinophilia/ or Eosinophilic Gastrointestinal Disorder/)) or ((Eosinophil* or Eosinophyl*) and (Esophag* or Oesophag*)).tw,kw. (10952)

16 14 and 15 (2068)

17 limit 16 to em=202142‐202308 (316)

### Appendix 4. ClinicalTrials.gov search strategy

**Advanced Search**

***Condition or disease:*** Eosinophilic Esophagitis

***Study type:*** Interventional Studies (Clinical Trials)

***First posted from*** 10/14/2021 ***to*** 03/03/2023

24 Studies found

### Appendix 5. WHO ICTRP search strategy

**Advanced Search**

Eosinophilic Esophagitis ***in the Condition***

***Recruitment status is*** ALL

***Date of registration is between*** 01/01/2021 ***and*** 03/03/2023

59 records for 40 trials found

## Data and analyses

**Comparison 1:** Corticosteroids vs placebo for induction of remission

Outcome or subgroup title	No. of studies	No. of participants	Statistical method	Effect size
1.1 Clinical improvement at study endpoint (dichotomous)	6	583	Risk Ratio (M‐H, Random, 95% CI)	1.74 [1.08, 2.80]
1.2 Clinical improvement at study endpoint (dichotomous), sensitivity analysis, fixed‐effect	6	583	Risk Ratio (M‐H, Fixed, 95% CI)	1.54 [1.25, 1.89]
1.3 Clinical improvement at study endpoint (dichotomous), sensitivity analysis, validated instruments	2	360	Risk Ratio (M‐H, Random, 95% CI)	1.39 [1.08, 1.79]
1.4 Clinical improvement at study endpoint (dichotomous), subgrouped by age	6	583	Risk Ratio (M‐H, Random, 95% CI)	1.74 [1.08, 2.80]
1.4.1 Children (18 years and younger)	1	81	Risk Ratio (M‐H, Random, 95% CI)	0.88 [0.60, 1.27]
1.4.2 Mixed children and adults (18 years and older)	5	502	Risk Ratio (M‐H, Random, 95% CI)	2.13 [1.27, 3.57]
1.5 Clinical improvement at study endpoint (dichotomous), subgrouped by type of steroid	6	583	Risk Ratio (M‐H, Random, 95% CI)	1.74 [1.08, 2.80]
1.5.1 Beclomethasone	1	18	Risk Ratio (M‐H, Random, 95% CI)	3.00 [0.38, 23.68]
1.5.2 Budesonide	4	523	Risk Ratio (M‐H, Random, 95% CI)	1.74 [0.95, 3.16]
1.5.3 Fluticasone	1	42	Risk Ratio (M‐H, Random, 95% CI)	1.71 [0.84, 3.48]
1.6 Clinical improvement at study endpoint (dichotomous), subgrouped by delivery method	6	583	Risk Ratio (M‐H, Random, 95% CI)	1.74 [1.08, 2.80]
1.6.1 Adapted asthma	3	96	Risk Ratio (M‐H, Random, 95% CI)	2.23 [1.30, 3.83]
1.6.2 Esophageal‐specific	3	487	Risk Ratio (M‐H, Random, 95% CI)	1.48 [0.79, 2.77]
1.7 Clinical improvement at study endpoint (continuous)	5	475	Std. Mean Difference (IV, Random, 95% CI)	0.51 [0.17, 0.85]
1.8 Clinical improvement at study endpoint (continuous), sensitivity analysis, fixed‐effect	5	475	Std. Mean Difference (IV, Fixed, 95% CI)	0.37 [0.18, 0.56]
1.9 Clinical improvement at study endpoint (continuous), sensitivity analysis, validated instruments	3	407	Std. Mean Difference (IV, Random, 95% CI)	0.35 [0.07, 0.64]
1.10 Clinical improvement at study endpoint (continuous), subgrouped by age	5	475	Std. Mean Difference (IV, Random, 95% CI)	0.51 [0.17, 0.85]
1.10.1 Children (18 years and younger)	1	32	Std. Mean Difference (IV, Random, 95% CI)	0.34 [‐0.39, 1.08]
1.10.2 Mixed children and adults (18 years and older)	4	443	Std. Mean Difference (IV, Random, 95% CI)	0.55 [0.14, 0.97]
1.11 Clinical improvement at study endpoint (continuous), subgrouped by type of steroid	5	475	Std. Mean Difference (IV, Random, 95% CI)	0.51 [0.17, 0.85]
1.11.1 Budesonide	4	442	Std. Mean Difference (IV, Random, 95% CI)	0.51 [0.10, 0.91]
1.11.2 Mometasone	1	33	Std. Mean Difference (IV, Random, 95% CI)	0.59 [‐0.11, 1.29]
1.12 Clinical improvement at study endpoint (continuous), subgrouped by delivery method	5	475	Std. Mean Difference (IV, Random, 95% CI)	0.51 [0.17, 0.85]
1.12.1 Adapted asthma	1	36	Std. Mean Difference (IV, Random, 95% CI)	1.23 [0.51, 1.95]
1.12.2 Esophageal‐specific	4	439	Std. Mean Difference (IV, Random, 95% CI)	0.31 [0.11, 0.51]
1.13 Histological improvement at study endpoint (dichotomous)	12	978	Risk Ratio (M‐H, Random, 95% CI)	11.94 [6.56, 21.75]
1.14 Histological improvement at study endpoint (dichotomous), sensitivity analysis, fixed‐effect	12	978	Risk Ratio (M‐H, Fixed, 95% CI)	18.87 [10.57, 33.71]
1.15 Histological improvement at study endpoint (dichotomous), sensitivity analysis, threshold < 15 eos/hpf	4	476	Risk Ratio (M‐H, Random, 95% CI)	18.47 [4.45, 76.72]
1.16 Histological improvement at study endpoint (dichotomous), sensitivity analysis, threshold ≤ 6 eos/hpf	10	912	Risk Ratio (M‐H, Random, 95% CI)	14.03 [6.73, 29.26]
1.17 Histological improvement at study endpoint (dichotomous), sensitivity analysis, threshold ≤ 1 eos/hpf	4	424	Risk Ratio (M‐H, Random, 95% CI)	10.97 [3.12, 38.55]
1.18 Histological improvement at study endpoint (dichotomous), subgrouped by age	12	978	Risk Ratio (M‐H, Random, 95% CI)	11.94 [6.56, 21.75]
1.18.1 Children (18 years and younger)	3	149	Risk Ratio (M‐H, Random, 95% CI)	5.76 [2.07, 16.02]
1.18.2 Mixed children and adults (18 years and older)	9	829	Risk Ratio (M‐H, Random, 95% CI)	15.17 [7.88, 29.23]
1.19 Histological improvement at study endpoint (dichotomous), subgrouped by type of steroid	12	978	Risk Ratio (M‐H, Random, 95% CI)	11.94 [6.56, 21.75]
1.19.1 Budesonide	7	728	Risk Ratio (M‐H, Random, 95% CI)	16.70 [7.60, 36.70]
1.19.2 Fluticasone	5	250	Risk Ratio (M‐H, Random, 95% CI)	7.57 [3.36, 17.08]
1.20 Histological improvement at study endpoint (dichotomous), subgrouped by delivery method	12	978	Risk Ratio (M‐H, Random, 95% CI)	11.94 [6.56, 21.75]
1.20.1 Esophageal‐specific	8	822	Risk Ratio (M‐H, Random, 95% CI)	18.20 [8.29, 39.95]
1.20.2 Adapted asthma, allergy	4	156	Risk Ratio (M‐H, Random, 95% CI)	7.30 [3.37, 15.84]
1.21 Histological improvement at study endpoint (continuous)	5	449	Std. Mean Difference (IV, Random, 95% CI)	1.42 [1.02, 1.82]
1.22 Histological improvement at study endpoint (continuous), sensitivity analysis, fixed‐effect	5	449	Std. Mean Difference (IV, Fixed, 95% CI)	1.33 [1.12, 1.55]
1.23 Histological improvement at study endpoint (continuous), subgrouped by age	5	492	Std. Mean Difference (IV, Random, 95% CI)	1.46 [1.03, 1.89]
1.23.1 Children (18 years and younger)	1	32	Std. Mean Difference (IV, Random, 95% CI)	2.31 [1.36, 3.26]
1.23.2 Mixed children and adults (18 years and older)	4	460	Std. Mean Difference (IV, Random, 95% CI)	1.31 [0.94, 1.67]
1.24 Histological improvement at study endpoint (continuous), subgrouped by type of steroid	5	449	Std. Mean Difference (IV, Random, 95% CI)	1.42 [1.02, 1.82]
1.24.1 Beclomethasone	1	9	Std. Mean Difference (IV, Random, 95% CI)	1.02 [‐0.44, 2.47]
1.24.2 Budesonide	4	440	Std. Mean Difference (IV, Random, 95% CI)	1.46 [1.02, 1.91]
1.25 Histological improvement at study endpoint (continuous), subgrouped by delivery method	5	449	Std. Mean Difference (IV, Random, 95% CI)	1.42 [1.02, 1.82]
1.25.1 Adapted asthma	2	45	Std. Mean Difference (IV, Random, 95% CI)	1.72 [0.76, 2.67]
1.25.2 Esophageal‐specific	3	404	Std. Mean Difference (IV, Random, 95% CI)	1.32 [0.88, 1.75]
1.26 Endoscopic improvement at study endpoint (dichotomous)	3	102	Risk Ratio (M‐H, Random, 95% CI)	2.60 [0.82, 8.19]
1.27 Endoscopic improvement at study endpoint (dichotomous), sensitivity analysis, fixed‐effect	3	102	Risk Ratio (M‐H, Fixed, 95% CI)	2.73 [1.27, 5.86]
1.28 Endoscopic improvement at study endpoint (dichotomous), sensitivity analysis, validated instruments	2	66	Risk Ratio (M‐H, Random, 95% CI)	5.87 [1.11, 31.02]
1.29 Endoscopic improvement at study endpoint (dichotomous), subgrouped by age	3	102	Risk Ratio (M‐H, Random, 95% CI)	2.60 [0.82, 8.19]
1.29.1 Children (18 years and younger)	1	36	Risk Ratio (M‐H, Random, 95% CI)	1.57 [0.69, 3.58]
1.29.2 Mixed children and adults (18 years and older)	2	66	Risk Ratio (M‐H, Random, 95% CI)	5.87 [1.11, 31.02]
1.30 Endoscopic improvement at study endpoint (dichotomous), subgrouped by delivery method	3	102	Risk Ratio (M‐H, Random, 95% CI)	2.60 [0.82, 8.19]
1.30.1 Adapted asthma	2	78	Risk Ratio (M‐H, Random, 95% CI)	1.78 [0.83, 3.83]
1.30.2 Esophageal‐specific	1	24	Risk Ratio (M‐H, Random, 95% CI)	11.12 [0.73, 168.69]
1.31 Endoscopic improvement at study endpoint (continuous)	5	596	Std. Mean Difference (IV, Random, 95% CI)	1.33 [0.59, 2.08]
1.32 Endoscopic improvement at study endpoint (continuous), sensitivity analysis, fixed‐effect	5	596	Std. Mean Difference (IV, Fixed, 95% CI)	0.93 [0.74, 1.11]
1.33 Endoscopic improvement at study endpoint (continuous), sensitivity analysis, validated instruments	4	572	Std. Mean Difference (IV, Random, 95% CI)	1.31 [0.46, 2.17]
1.34 Endoscopic improvement at study endpoint (continuous), subgrouped by age	5	596	Std. Mean Difference (IV, Random, 95% CI)	1.33 [0.59, 2.08]
1.34.1 Children (18 years and younger)	1	24	Std. Mean Difference (IV, Random, 95% CI)	1.44 [0.50, 2.38]
1.34.2 Mixed children and adults (18 years and older)	4	572	Std. Mean Difference (IV, Random, 95% CI)	1.31 [0.46, 2.17]
1.35 Endoscopic improvement at study endpoint (continuous), subgrouped by type of steroid	5	596	Std. Mean Difference (IV, Random, 95% CI)	1.33 [0.59, 2.08]
1.35.1 Budesonide	4	493	Std. Mean Difference (IV, Random, 95% CI)	1.41 [0.44, 2.37]
1.35.2 Fluticasone	1	103	Std. Mean Difference (IV, Random, 95% CI)	1.10 [0.58, 1.62]
1.36 Withdrawals due to adverse events	14	1032	Risk Ratio (M‐H, Random, 95% CI)	0.64 [0.43, 0.96]
1.37 Withdrawals due to adverse events, sensitivity analysis, fixed‐effect	14	1032	Risk Ratio (M‐H, Fixed, 95% CI)	0.65 [0.44, 0.94]
1.38 Withdrawals due to adverse events, subgrouped by age	14	1032	Risk Ratio (M‐H, Random, 95% CI)	0.64 [0.43, 0.96]
1.38.1 Children (18 years and younger)	3	149	Risk Ratio (M‐H, Random, 95% CI)	0.88 [0.28, 2.72]
1.38.2 Mixed children and adults (18 years and older)	11	883	Risk Ratio (M‐H, Random, 95% CI)	0.57 [0.36, 0.90]
1.39 Withdrawals due to adverse events, subgrouped by type of steroid	14	1032	Risk Ratio (M‐H, Random, 95% CI)	0.64 [0.43, 0.96]
1.39.1 Beclomethasone	1	0	Risk Ratio (M‐H, Random, 95% CI)	Not estimable
1.39.2 Budesonide	7	728	Risk Ratio (M‐H, Random, 95% CI)	0.80 [0.49, 1.31]
1.39.3 Fluticasone	5	250	Risk Ratio (M‐H, Random, 95% CI)	0.42 [0.20, 0.91]
1.39.4 Mometasone	1	36	Risk Ratio (M‐H, Random, 95% CI)	0.56 [0.06, 5.63]
1.40 Withdrawals due to adverse events, subgrouped by delivery method	14	1032	Risk Ratio (M‐H, Random, 95% CI)	0.64 [0.43, 0.96]
1.40.1 Adapted asthma	5	174	Risk Ratio (M‐H, Random, 95% CI)	0.51 [0.12, 2.12]
1.40.2 Esophageal‐specific	9	858	Risk Ratio (M‐H, Random, 95% CI)	0.68 [0.44, 1.04]
1.41 Serious adverse events	14	1032	Risk Ratio (M‐H, Random, 95% CI)	0.35 [0.17, 0.73]
1.42 Total adverse events	13	1014	Risk Ratio (M‐H, Random, 95% CI)	1.14 [0.94, 1.40]
1.43 Quality of life at study endpoint (continuous)	1	88	Mean Difference (IV, Random, 95% CI)	0.20 [‐0.14, 0.54]

**Comparison 2:** Corticosteroids vs placebo for maintenance of remission

Outcome or subgroup title	No. of studies	No. of participants	Statistical method	Effect size
2.1 Clinical improvement at study endpoint (dichotomous)	2	252	Risk Ratio (M‐H, Random, 95% CI)	2.17 [0.75, 6.27]
2.2 Clinical improvement at study endpoint (continuous)	3	269	Std. Mean Difference (IV, Random, 95% CI)	0.51 [‐0.49, 1.52]
2.3 Histological improvement at study endpoint (dichotomous)	3	280	Risk Ratio (M‐H, Random, 95% CI)	4.58 [1.66, 12.62]
2.4 Histological improvement at study endpoint (continuous)	3	269	Std. Mean Difference (IV, Random, 95% CI)	1.26 [0.74, 1.78]
2.5 Endoscopic improvement at study endpoint (continuous)	2	240	Std. Mean Difference (IV, Random, 95% CI)	1.34 [‐0.27, 2.95]
2.6 Withdrawals due to adverse events	3	280	Risk Ratio (M‐H, Random, 95% CI)	0.37 [0.16, 0.87]
2.7 Serious adverse events	3	280	Risk Ratio (M‐H, Random, 95% CI)	1.27 [0.09, 18.03]
2.8 Total adverse events	3	280	Risk Ratio (M‐H, Random, 95% CI)	1.10 [0.75, 1.62]
2.9 Quality of life at study endpoint (continuous)	1	204	Mean Difference (IV, Random, 95% CI)	0.60 [0.40, 0.80]

**Comparison 3:** Biologics vs placebo for induction of remission

Outcome or subgroup title	No. of studies	No. of participants	Statistical method	Effect size
3.1 Clinical improvement at study endpoint (dichotomous)	5	410	Risk Ratio (M‐H, Random, 95% CI)	1.14 [0.85, 1.52]
3.2 Clinical improvement at study endpoint (dichotomous), sensitivity analysis, fixed‐effect	5	410	Risk Ratio (M‐H, Fixed, 95% CI)	1.10 [0.92, 1.32]
3.3 Clinical improvement at study endpoint (dichotomous), sensitivity analysis, validated instruments	3	172	Risk Ratio (M‐H, Random, 95% CI)	1.37 [1.02, 1.85]
3.4 Clinical improvement at study endpoint (dichotomous), subgrouped by age	5	410	Risk Ratio (M‐H, Random, 95% CI)	1.14 [0.85, 1.52]
3.4.1 Children (18 years and younger)	1	227	Risk Ratio (M‐H, Random, 95% CI)	0.91 [0.72, 1.14]
3.4.2 Mixed children and adults (18 years and older)	4	183	Risk Ratio (M‐H, Random, 95% CI)	1.36 [1.02, 1.81]
3.5 Clinical improvement at study endpoint (dichotomous), subgrouped by mechanism	5	410	Risk Ratio (M‐H, Random, 95% CI)	1.14 [0.85, 1.52]
3.5.1 Anti‐IL‐13 (RPC4046, alias cendakimab; QAX576, alias dectrekumab) and anti‐IL‐4r (dupilumab)	3	172	Risk Ratio (M‐H, Random, 95% CI)	1.37 [1.02, 1.85]
3.5.2 Anti‐IL‐5 (mepolizumab, reslizumab)	2	238	Risk Ratio (M‐H, Random, 95% CI)	0.92 [0.73, 1.15]
3.6 Clinical improvement at study endpoint (continuous)	7	387	Std. Mean Difference (IV, Random, 95% CI)	0.50 [0.22, 0.78]
3.7 Clinical improvement at study endpoint (continuous), sensitivity analysis, fixed‐effect	7	387	Std. Mean Difference (IV, Fixed, 95% CI)	0.48 [0.28, 0.69]
3.8 Clinical improvement at study endpoint (continuous), sensitivity analysis, peer‐reviewed manuscripts	5	162	Std. Mean Difference (IV, Random, 95% CI)	0.36 [‐0.09, 0.81]
3.9 Clinical improvement at study endpoint (continuous), sensitivity analysis, less than high risk of bias	6	357	Std. Mean Difference (IV, Random, 95% CI)	0.61 [0.40, 0.82]
3.10 Clinical improvement at study endpoint (continuous), sensitivity analysis, validated instruments	3	247	Std. Mean Difference (IV, Random, 95% CI)	0.62 [0.37, 0.88]
3.11 Clinical improvement at study endpoint (continuous), subgrouped by mechanism	7	387	Std. Mean Difference (IV, Random, 95% CI)	0.40 [0.05, 0.74]
3.11.1 Anti‐IgE (omalizumab)	1	30	Std. Mean Difference (IV, Random, 95% CI)	‐0.47 [‐1.20, 0.26]
3.11.2 Anti‐IL‐13 (RPC4046, alias cendakimab; QAX576, alias dectrekumab) and anti‐IL‐4r (dupilumab)	5	346	Std. Mean Difference (IV, Random, 95% CI)	0.60 [0.39, 0.82]
3.11.3 Anti‐IL‐5 (mepolizumab, reslizumab)	1	11	Std. Mean Difference (IV, Random, 95% CI)	‐0.79 [‐2.05, 0.46]
3.12 Histological improvement at study endpoint (dichotomous)	8	925	Risk Ratio (M‐H, Random, 95% CI)	6.73 [2.58, 17.52]
3.13 Histological improvement at study endpoint (dichotomous), sensitivity analysis, fixed‐effect	8	925	Risk Ratio (M‐H, Fixed, 95% CI)	5.12 [3.86, 6.78]
3.14 Histological improvement at study endpoint (dichotomous), sensitivity analysis, peer‐reviewed manuscripts	6	685	Risk Ratio (M‐H, Random, 95% CI)	6.13 [1.67, 22.51]
3.15 Histological improvement at study endpoint (dichotomous), sensitivity analysis, less than high risk of bias	8	925	Risk Ratio (M‐H, Random, 95% CI)	6.73 [2.58, 17.52]
3.16 Histological improvement at study endpoint (dichotomous), sensitivity analysis, threshold < 15 eos/hpf	4	418	Risk Ratio (M‐H, Random, 95% CI)	5.61 [1.00, 31.32]
3.17 Histological improvement at study endpoint (dichotomous), sensitivity analysis, threshold ≤ 6 eos/hpf	6	674	Risk Ratio (M‐H, Random, 95% CI)	8.85 [5.73, 13.67]
3.18 Histological improvement at study endpoint (dichotomous), sensitivity analysis, threshold ≤ 1 eos/hpf	2	323	Risk Ratio (M‐H, Random, 95% CI)	18.01 [7.24, 44.83]
3.19 Histological improvement at study endpoint (dichotomous), subgrouped by age	8	925	Risk Ratio (M‐H, Random, 95% CI)	7.18 [2.93, 17.56]
3.19.1 Children (18 years and younger)	2	277	Risk Ratio (M‐H, Random, 95% CI)	4.51 [0.40, 50.36]
3.19.2 Mixed children and adults (18 years and older)	6	423	Risk Ratio (M‐H, Random, 95% CI)	9.01 [4.88, 16.62]
3.19.3 Adults only (over 18 years)	1	225	Risk Ratio (M‐H, Random, 95% CI)	7.44 [4.02, 13.77]
3.20 Histological improvement at study endpoint (dichotomous), subgrouped by mechanism	8	925	Risk Ratio (M‐H, Random, 95% CI)	6.73 [2.58, 17.52]
3.20.1 Anti‐sialic acid‐binding Ig‐like lectin 8 (lirentelimab)	1	276	Risk Ratio (M‐H, Random, 95% CI)	8.30 [4.61, 14.93]
3.20.2 Anti‐IL‐13 (RPC4046, alias cendakimab; QAX576, alias dectrekumab) and anti‐IL‐4r (dupilumab)	5	412	Risk Ratio (M‐H, Random, 95% CI)	9.01 [4.88, 16.62]
3.20.3 Anti‐IL‐5 (mepolizumab, reslizumab)	2	237	Risk Ratio (M‐H, Random, 95% CI)	1.75 [1.21, 2.55]
3.21 Histological improvement at study endpoint (continuous)	6	370	Std. Mean Difference (IV, Random, 95% CI)	1.01 [0.36, 1.66]
3.22 Histological improvement at study endpoint (continuous), sensitivity analysis, fixed‐effect	6	370	Std. Mean Difference (IV, Fixed, 95% CI)	1.25 [1.01, 1.49]
3.23 Histological improvement at study endpoint (continuous), sensitivity analysis, less than high risk of bias	5	340	Std. Mean Difference (IV, Random, 95% CI)	1.39 [1.01, 1.77]
3.24 Histological improvement at study endpoint (continuous), subgrouped by age	6	370	Std. Mean Difference (IV, Random, 95% CI)	1.01 [0.36, 1.66]
3.24.1 Children (18 years and younger)	1	169	Std. Mean Difference (IV, Random, 95% CI)	1.64 [1.25, 2.02]
3.24.2 Mixed children and adults (18 years and older)	5	201	Std. Mean Difference (IV, Random, 95% CI)	0.85 [0.04, 1.66]
3.25 Histological improvement at study endpoint (continuous), subgrouped by mechanism	6	370	Std. Mean Difference (IV, Random, 95% CI)	1.01 [0.36, 1.66]
3.25.1 Anti‐IgE (omalizumab)	1	30	Std. Mean Difference (IV, Random, 95% CI)	‐0.43 [‐1.15, 0.30]
3.25.2 Anti‐IL‐13 (RPC4046, alias cendakimab; QAX576, alias dectrekumab) and anti‐IL‐4r (dupilumab)	3	160	Std. Mean Difference (IV, Random, 95% CI)	1.32 [0.72, 1.91]
3.25.3 Anti‐IL‐5 (mepolizumab, reslizumab)	2	180	Std. Mean Difference (IV, Random, 95% CI)	1.42 [0.69, 2.15]
3.26 Endoscopic improvement at study endpoint (dichotomous)	1	0	Risk Ratio (M‐H, Random, 95% CI)	Not estimable
3.27 Endoscopic improvement at study endpoint (continuous)	3	197	Std. Mean Difference (IV, Random, 95% CI)	2.79 [0.36, 5.22]
3.28 Endoscopic improvement at study endpoint (continuous), sensitivity analysis, fixed‐effect	3	197	Std. Mean Difference (IV, Fixed, 95% CI)	1.20 [0.86, 1.55]
3.29 Endoscopic improvement at study endpoint (continuous), sensitivity analysis, less than high risk of bias	2	136	Std. Mean Difference (IV, Random, 95% CI)	0.82 [0.42, 1.21]
3.30 Withdrawals due to adverse events	8	792	Risk Ratio (M‐H, Random, 95% CI)	1.55 [0.88, 2.74]
3.31 Withdrawals due to adverse events, sensitivity analysis, fixed‐effect	8	792	Risk Ratio (M‐H, Fixed, 95% CI)	1.53 [0.89, 2.64]
3.32 Withdrawals due to adverse events, sensitivity analysis, less than high risk of bias	6	681	Risk Ratio (M‐H, Random, 95% CI)	1.55 [0.88, 2.74]
3.33 Withdrawals due to adverse events, sensitivity analysis, peer reviewed manuscripts	7	711	Risk Ratio (M‐H, Random, 95% CI)	1.55 [0.88, 2.74]
3.34 Withdrawals due to adverse events, subgrouped by age	8	792	Risk Ratio (M‐H, Random, 95% CI)	1.55 [0.88, 2.74]
3.34.1 Children (18 years and younger)	1	227	Risk Ratio (M‐H, Random, 95% CI)	1.45 [0.63, 3.35]
3.34.2 Mixed children and adults (18 years and older)	7	565	Risk Ratio (M‐H, Random, 95% CI)	1.59 [0.68, 3.74]
3.35 Withdrawals due to adverse events, subgrouped by mechanism	8	792	Risk Ratio (M‐H, Random, 95% CI)	1.31 [0.66, 2.59]
3.35.1 Anti‐IgE (omalizumab)	1	0	Risk Ratio (M‐H, Random, 95% CI)	Not estimable
3.35.2 Anti‐sialic acid‐binding Ig‐like lectin 8 (lirentelimab)	1	276	Risk Ratio (M‐H, Random, 95% CI)	2.12 [0.74, 6.13]
3.35.3 Anti‐IL‐13 (RPC4046, alias cendakimab; QAX576, alias dectrekumab) and anti‐IL‐4r (dupilumab)	4	248	Risk Ratio (M‐H, Random, 95% CI)	0.91 [0.36, 2.33]
3.35.4 Anti‐IL‐5 (mepolizumab, reslizumab)	2	238	Risk Ratio (M‐H, Random, 95% CI)	1.02 [0.04, 24.63]
3.36 Serious adverse events	6	685	Risk Ratio (M‐H, Random, 95% CI)	0.70 [0.25, 1.97]
3.37 Total adverse events	6	685	Risk Ratio (M‐H, Random, 95% CI)	1.07 [0.94, 1.23]
3.38 Quality of life at study endpoint (continuous)	1	47	Mean Difference (IV, Random, 95% CI)	0.33 [‐0.06, 0.72]

**Comparison 4:** Cromolyn sodium vs placebo

Outcome or subgroup title	No. of studies	No. of participants	Statistical method	Effect size
4.1 Clinical improvement at study endpoint (continuous)	1	14	Mean Difference (IV, Random, 95% CI)	4.70 [‐12.09, 21.49]
4.2 Histological improvement at study endpoint (continuous)	1	15	Mean Difference (IV, Random, 95% CI)	14.20 [‐36.90, 65.30]
4.3 Withdrawals due to adverse events	1	16	Risk Ratio (M‐H, Random, 95% CI)	0.27 [0.01, 5.70]
4.4 Serious adverse events	1	0	Risk Ratio (M‐H, Random, 95% CI)	Not estimable

**Comparison 5:** PGD2R antagonist OC000459 vs placebo

Outcome or subgroup title	No. of studies	No. of participants	Statistical method	Effect size
5.1 Clinical improvement at study endpoint (continuous)	1	26	Mean Difference (IV, Random, 95% CI)	‐1.06 [‐6.80, 4.68]
5.2 Histological improvement at study endpoint (continuous)	1	26	Mean Difference (IV, Random, 95% CI)	26.21 [‐23.78, 76.20]
5.3 Endoscopic improvement at study endpoint (continuous)	1	26	Mean Difference (IV, Random, 95% CI)	‐0.49 [‐2.05, 1.07]
5.4 Withdrawals due to adverse events	1	0	Risk Ratio (M‐H, Random, 95% CI)	Not estimable
5.5 Serious adverse events	1	26	Risk Ratio (M‐H, Random, 95% CI)	2.60 [0.12, 58.48]

**Comparison 6:** Swallowed fluticasone vs oral prednisone

Outcome or subgroup title	No. of studies	No. of participants	Statistical method	Effect size
6.1 Clinical improvement at study endpoint (dichotomous)	1	80	Risk Ratio (M‐H, Random, 95% CI)	1.09 [0.90, 1.33]
6.2 Histological improvement at study endpoint (dichotomous)	1	80	Risk Ratio (M‐H, Random, 95% CI)	1.10 [0.87, 1.38]
6.3 Histological improvement at study endpoint (continuous)	1	68	Mean Difference (IV, Random, 95% CI)	‐4.45 [‐9.08, 0.18]
6.4 Endoscopic improvement at study endpoint (dichotomous)	1	80	Risk Ratio (M‐H, Random, 95% CI)	1.13 [0.91, 1.41]
6.5 Withdrawals due to adverse events	1	80	Risk Ratio (M‐H, Random, 95% CI)	0.50 [0.16, 1.53]
6.6 Serious adverse events	1	80	Risk Ratio (M‐H, Random, 95% CI)	0.14 [0.01, 2.68]
6.7 Total adverse events	1	80	Risk Ratio (M‐H, Random, 95% CI)	0.38 [0.16, 0.86]

**Comparison 7:** Oral viscous budesonide vs swallowed fluticasone

Outcome or subgroup title	No. of studies	No. of participants	Statistical method	Effect size
7.1 Clinical improvement at study endpoint (continuous)	1	84	Mean Difference (IV, Random, 95% CI)	‐0.60 [‐3.78, 2.58]
7.2 Histological improvement at study endpoint (dichotomous)	1	129	Risk Ratio (M‐H, Random, 95% CI)	1.13 [0.84, 1.51]
7.3 Histological improvement at study endpoint (continuous)	1	111	Mean Difference (IV, Random, 95% CI)	6.20 [‐5.63, 18.03]
7.4 Endoscopic improvement at study endpoint (continuous)	1	111	Mean Difference (IV, Random, 95% CI)	0.70 [‐0.03, 1.43]
7.5 Withdrawals due to adverse events	1	129	Risk Ratio (M‐H, Random, 95% CI)	0.98 [0.42, 2.32]
7.6 Serious adverse events	1	129	Risk Ratio (M‐H, Random, 95% CI)	0.33 [0.01, 7.91]
7.7 Total adverse events	1	129	Risk Ratio (M‐H, Random, 95% CI)	0.66 [0.32, 1.35]

**Comparison 8:** Esomeprazole vs fluticasone

Outcome or subgroup title	No. of studies	No. of participants	Statistical method	Effect size
8.1 Clinical improvement at study endpoint (continuous)	2	63	Std. Mean Difference (IV, Random, 95% CI)	0.32 [‐0.88, 1.52]
8.2 Histological improvement at study endpoint (dichotomous)	2	72	Risk Ratio (M‐H, Random, 95% CI)	1.62 [0.77, 3.41]
8.3 Histological improvement at study endpoint (continuous)	2	67	Std. Mean Difference (IV, Random, 95% CI)	0.28 [‐0.20, 0.76]
8.4 Withdrawals due to adverse events	2	72	Risk Ratio (M‐H, Random, 95% CI)	0.95 [0.07, 13.38]
8.5 Serious adverse events	2	0	Risk Ratio (M‐H, Random, 95% CI)	Not estimable
8.6 Total adverse events	2	72	Risk Ratio (M‐H, Random, 95% CI)	0.14 [0.01, 2.61]

**Comparison 9:** One‐food elimination diet vs four‐food elimination diet

Outcome or subgroup title	No. of studies	No. of participants	Statistical method	Effect size
9.1 Clinical improvement at study endpoint (continuous)	1	50	Mean Difference (IV, Random, 95% CI)	‐7.50 [‐16.28, 1.28]
9.2 Histological improvement at study endpoint (dichotomous)	1	63	Risk Ratio (M‐H, Random, 95% CI)	2.26 [1.15, 4.43]
9.3 Endoscopic improvement at study endpoint (continuous)	1	34	Mean Difference (IV, Random, 95% CI)	‐0.60 [‐2.15, 0.95]
9.4 Withdrawals due to adverse events	1	63	Risk Ratio (M‐H, Random, 95% CI)	0.33 [0.11, 0.98]
9.5 Serious adverse events	1	63	Risk Ratio (M‐H, Random, 95% CI)	0.66 [0.04, 10.04]
9.6 Total adverse events	1	63	Risk Ratio (M‐H, Random, 95% CI)	0.41 [0.15, 1.11]
9.7 Quality of life at study endpoint (continuous)	1	63	Mean Difference (IV, Random, 95% CI)	0.10 [‐6.49, 6.69]

**Comparison 10:** One‐food elimination diet vs six‐food elimination diet

Outcome or subgroup title	No. of studies	No. of participants	Statistical method	Effect size
10.1 Clinical improvement at study endpoint (continuous)	1	129	Mean Difference (IV, Random, 95% CI)	‐5.20 [‐11.06, 0.66]
10.2 Histological improvement at study endpoint (dichotomous)	1	129	Risk Ratio (M‐H, Random, 95% CI)	0.85 [0.54, 1.33]
10.3 Histological improvement at study endpoint (continuous)	1	129	Mean Difference (IV, Random, 95% CI)	6.80 [‐10.40, 24.00]
10.4 Endoscopic improvement at study endpoint (continuous)	1	129	Mean Difference (IV, Random, 95% CI)	‐0.42 [‐1.67, 0.83]
10.5 Withdrawals due to adverse events	1	129	Risk Ratio (M‐H, Random, 95% CI)	0.62 [0.11, 3.57]
10.6 Serious adverse events	1	0	Risk Ratio (M‐H, Random, 95% CI)	Not estimable
10.7 Total adverse events	1	129	Risk Ratio (M‐H, Random, 95% CI)	0.46 [0.04, 4.98]
10.8 Quality of life at study endpoint (continuous)	1	129	Mean Difference (IV, Random, 95% CI)	0.57 [‐3.25, 4.39]

**Comparison 11:** Four‐food elimination diet with omeprazole vs omeprazole

Outcome or subgroup title	No. of studies	No. of participants	Statistical method	Effect size
11.1 Histological improvement at study endpoint (dichotomous)	1	64	Risk Ratio (M‐H, Random, 95% CI)	1.57 [0.99, 2.48]
11.2 Histological improvement at study endpoint (continuous)	1	58	Mean Difference (IV, Random, 95% CI)	9.50 [‐11.18, 30.18]
11.3 Withdrawals due to adverse events	1	64	Risk Ratio (M‐H, Random, 95% CI)	5.00 [0.62, 40.44]

**Comparison 12:** Four‐food elimination diet with amino acid formula vs four‐food elimination diet

Outcome or subgroup title	No. of studies	No. of participants	Statistical method	Effect size
12.1 Clinical improvement at study endpoint (continuous)	1	41	Mean Difference (IV, Random, 95% CI)	‐0.50 [‐2.41, 1.41]
12.2 Histological improvement at study endpoint (dichotomous)	1	41	Risk Ratio (M‐H, Random, 95% CI)	1.90 [0.79, 4.60]
12.3 Histological improvement at study endpoint (continuous)	1	41	Mean Difference (IV, Random, 95% CI)	13.80 [‐9.50, 37.10]
12.4 Endoscopic improvement at study endpoint (continuous)	1	41	Mean Difference (IV, Random, 95% CI)	‐1.00 [‐2.83, 0.83]
12.5 Withdrawals due to adverse events	1	41	Risk Ratio (M‐H, Random, 95% CI)	0.95 [0.06, 14.22]
12.6 Serious adverse events	1	0	Risk Ratio (M‐H, Random, 95% CI)	Not estimable
12.7 Total adverse events	1	41	Risk Ratio (M‐H, Random, 95% CI)	2.86 [0.12, 66.44]

**Comparison 13:** Nebulized budesonide vs viscous budesonide

Outcome or subgroup title	No. of studies	No. of participants	Statistical method	Effect size
13.1 Clinical improvement at study endpoint (continuous)	1	22	Mean Difference (IV, Random, 95% CI)	‐6.00 [‐18.30, 6.30]
13.2 Histological improvement at study endpoint (continuous)	1	22	Mean Difference (IV, Random, 95% CI)	78.00 [20.81, 135.19]
13.3 Serious adverse events	1	0	Risk Ratio (M‐H, Random, 95% CI)	Not estimable
13.4 Total adverse events	1	25	Risk Ratio (M‐H, Random, 95% CI)	0.92 [0.15, 5.56]

**Comparison 14:** Viaskin milk patch vs placebo

Outcome or subgroup title	No. of studies	No. of participants	Statistical method	Effect size
14.1 Clinical improvement at study endpoint (continuous)	1	9	Mean Difference (IV, Random, 95% CI)	1.29 [‐0.83, 3.41]
14.2 Histological improvement at study endpoint (continuous)	1	9	Mean Difference (IV, Random, 95% CI)	69.43 [‐21.75, 160.61]
14.3 Endoscopic improvement at study endpoint (continuous)	1	20	Mean Difference (IV, Random, 95% CI)	‐0.33 [‐2.00, 1.34]
14.4 Withdrawals due to adverse events	1	20	Risk Ratio (M‐H, Random, 95% CI)	1.12 [0.05, 23.99]
14.5 Serious adverse events	1	20	Risk Ratio (M‐H, Random, 95% CI)	0.13 [0.01, 2.67]
14.6 Total adverse events	1	20	Risk Ratio (M‐H, Random, 95% CI)	1.00 [0.77, 1.29]
14.7 Quality of life at study endpoint (continuous)	1	9	Mean Difference (IV, Random, 95% CI)	13.60 [‐16.12, 43.32]

**Comparison 15:** Leukotriene receptor antagonist vs placebo for maintenance of remission

Outcome or subgroup title	No. of studies	No. of participants	Statistical method	Effect size
15.1 Clinical improvement at study endpoint (dichotomous)	1	41	Risk Ratio (M‐H, Random, 95% CI)	1.68 [0.66, 4.28]
15.2 Withdrawals due to adverse events	1	41	Risk Ratio (M‐H, Random, 95% CI)	2.10 [0.21, 21.39]
15.3 Serious adverse events	1	0	Risk Ratio (M‐H, Random, 95% CI)	Not estimable
15.4 Total adverse events	1	0	Risk Ratio (M‐H, Random, 95% CI)	Not estimable

**Comparison 16:** Mepolizumab 10 mg/kg vs mepolizumab 0.55 mg/kg

Outcome or subgroup title	No. of studies	No. of participants	Statistical method	Effect size
16.1 Histological improvement at study endpoint (dichotomous)	1	39	Risk Ratio (IV, Random, 95% CI)	1.19 [0.37, 3.77]
16.2 Withdrawals due to adverse events	1	39	Risk Ratio (IV, Random, 95% CI)	0.63 [0.12, 3.38]

**Comparison 17:** Mepolizumab 2.5 mg/kg vs mepolizumab 0.55 mg/kg

Outcome or subgroup title	No. of studies	No. of participants	Statistical method	Effect size
17.1 Histological improvement at study endpoint (dichotomous)	1	39	Risk Ratio (IV, Random, 95% CI)	2.14 [0.79, 5.79]
17.2 Withdrawals due to adverse events	1	39	Risk Ratio (IV, Random, 95% CI)	0.32 [0.04, 2.79]

**Comparison 18:** Mepolizumab 10 mg/kg vs mepolizumab 2.5 mg/kg

Outcome or subgroup title	No. of studies	No. of participants	Statistical method	Effect size
18.1 Histological improvement at study endpoint (dichotomous)	1	40	Risk Ratio (IV, Random, 95% CI)	0.56 [0.23, 1.37]
18.2 Withdrawals due to adverse events	1	40	Risk Ratio (IV, Random, 95% CI)	2.00 [0.20, 20.33]

## Characteristics of studies

### Characteristics of included studies [ordered by study ID]

**Table:** Alexander 2012

***Study characteristics***		
Methods	**RCT design and number of study arms:** RCT; 2 arms **Single‐center or multi‐center:** single‐center; Esophageal Clinic at Mayo Clinic Rochester Minnesota **Countries:** USA **Study dates:** October 2005 to December 2009	
Participants	**Active EoE or inactive EoE at beginning of study:** active **EoE definition/diagnostic criteria: ≥** 20 eosinophils/hpf (peak) **Inclusion criteria:** 18 to 65 years of age Mayo Dysphagia Questionnaire: 1a) “Have you had trouble swallowing unrelated to a sore throat or cold?” ‐ yes; 1c) Severity ≥ moderate; 2) Frequency ‐ at least once per week ≥ 20 peak eosinophils/hpf on biopsies from mid esophagus (hpf = high‐powered field) **Exclusion criteria:** Patients with endoscopic evidence of stricture, Schatzki ring, mass, or Los Angeles grade C or D esophagitis at endoscopy Topical steroid therapy for EoE at any time in the past Esophageal dilation at the index endoscopy or at any time in the past **Age at beginning of study per study group:** fluticasone mean (range): 37 years (19 to 59); placebo mean (range): 38 (20 to 57) **Sex (m/f) per study group:** fluticasone (m/f): 18/3; placebo (m/f): 16/5 **Number randomized per study group:** fluticasone: 21; placebo: 21 **Number reaching end of study per study group:** fluticasone: 19; placebo: 15	
Interventions	**Study group 1 (placebo):** placebo inhaler swallowed twice a day for 6 weeks **Study group 2 (fluticasone):** aerosolized swallowed fluticasone 880 µg twice a day for 6 weeks	
Outcomes	**Primary outcomes of the study:** number of participants with complete response to dysphagia (time frame: 2 weeks), measured by the Mayo Dysphagia Questionnaire, a validated 28‐item instrument: 0 = no dysphagia, higher levels indicate greater dysphagia severity. A complete symptom response was defined as an answer of "no" to the question "In the past 2 weeks, have you had trouble swallowing, not associated with other cold symptoms (such as strep throat or mono)?" **Secondary outcomes of the study:** Number of participants with complete response to dysphagia (time frame: 2 weeks), measured by the Mayo Dysphagia Questionnaire, a validated 28 item instrument; 0 = no dysphagia, higher levels indicate greater dysphagia severity. A partial symptom response was defined as an answer of "yes" to the question "In the past 2 weeks, have you had trouble swallowing, not associated with other cold symptoms (such as strep throat or mono)?" and a decrease in severity of at least 2 levels, or a decrease in frequency of at least 1 level. Number of participants with complete histologic response (time frame: 2 weeks) A complete histologic response was defined as a > 90% decrease in mean eosinophil count/high‐powered field. A partial response was defined as a decrease of more than 50% from the pre‐treatment value.	
Notes	**Funding source:** Mayo Clinic **Conflicts of interest:** Jeffrey Alexander is a consultant for Meritage Pharmacia and has stock ownership in Meritage Pharmacia	
***Risk of bias***		
**Bias**	**Authors' judgement**	**Support for judgement**
Random sequence generation (selection bias)	Low risk	Computer‐generated, coded randomization method.
Allocation concealment (selection bias)	Low risk	"The pharmacist and esophageal nurse saw the actual inhalers which were similar but not identical; all other study personnel were blinded to the study treatment allocation. Other than the inhaler education session, the esophageal nurse had no other subject contact or involvement with the study."
Blinding of participants and personnel (performance bias) All outcomes	Low risk	The author confirmed that both treatments "were inhalers with no particular taste" and caregivers were also blinded.
Blinding of outcome assessment (detection bias) All outcomes	Low risk	The author confirmed that "the outcome assessors were blinded".
Incomplete outcome data (attrition bias) All outcomes	Low risk	2 participants from the fluticasone arm (1 travel, 1 scheduling) and 6 participants from the placebo arm (1 withdrawal by participant, 1 travel, 2 scheduling, 2 family issues) did not finish the study. Balanced and explained attrition that likely has no effect on outcome.
Selective reporting (reporting bias)	Low risk	Protocol is available and all pre‐specified outcomes of interest were reported in the pre‐specified way.
Other bias	Low risk	PPI use was continued during the study by 26.3% (5 of 19) of the fluticasone group and 0% (0 of 15) of the placebo group No major baseline differences between groups. No other concerns.

**Table:** Alexander 2017

***Study characteristics***		
Methods	**RCT design and number of study arms:** RCT, 2 arms **Single‐center or multi‐center:** single‐center, Esophageal Clinic at Mayo Clinic in Rochester Minnesota **Countries:** USA **Study dates:** April 2008 to February 2015	
Participants	**Active EoE or inactive EoE at beginning of study:** inactive **EoE definition/diagnostic criteria:** ≥ 20 eosinophils/hpf (peak); Los Angeles Grade A or B after steroid treatment **Inclusion criteria:** 18 to 65 years of age Mayo Dysphagia Questionnaire: (1a) “Have you had trouble swallowing unrelated to a sore throat or cold?” ‐ yes; (1c) Severity ≥ moderate; (2) Frequency ≥ 1/week ≥ 20 peak eosinophils/HPF on biopsies from mid‐esophagus at baseline Los Angeles Grade A or B after steroid treatment **Exclusion criteria:** Patients with endoscopic evidence of stricture, Schatzki ring, mass, or Los Angeles grade C or D esophagitis at endoscopy Patients with other diseases known to be associated with esophageal eosinophilia Patients who underwent esophageal dilation within a year Patients without a complete symptomatic response to topical steroid therapy **Age at beginning of study per study group:** montelukast mean (SD): 44.4 (13.7); placebo mean (SD): 40.4 (12.1) **Sex (m/f) per study group:** montelukast m/f = 13/7 (65.0%/35%); placebo m/f = 12/9 (57.1%/42.9%) **Number randomized per study group:** montelukast n = 20; placebo n = 21 **Number reaching end of study per study group:** montelukast n = 18; placebo n = 20	
Interventions	**Study group 1:** placebo tablets, similar‐appearing **Study group 2:** montelukast 20 mg tablets for 26 weeks or until they developed symptomatic dysphagia	
Outcomes	**Primary outcomes of the study:** symptomatic remission is defined as the absence of dysphagia as defined above **Secondary outcomes of the study:** unclear ‐ outcomes not properly defined in the paper but defined in the protocol: "evaluate safety of montelukast in eosinophilic esophagitis"	
Notes	**Funding source:** this work was supported by a grant from Merck (Kenilworth, NJ) **Conflicts of interest: "**These authors disclose the following: Jeffrey A. Alexander owns stock in Meritage Pharmacia/Shire, and has received research funding from Merck and Shire, and David A. Katzka has received research funding from Shire and Covidien. The remaining authors disclose no conflicts."	
***Risk of bias***		
**Bias**	**Authors' judgement**	**Support for judgement**
Random sequence generation (selection bias)	Low risk	Computer‐generated, coded randomization method.
Allocation concealment (selection bias)	Low risk	Placebo tablets were similar‐appearing to the montelukast tablets. Only a pharmacist, who had no participant involvement, was unblinded to the participants.
Blinding of participants and personnel (performance bias) All outcomes	Low risk	The study was blinded to all caregivers but was known only by the pharmacist who had no participant involvement.
Blinding of outcome assessment (detection bias) All outcomes	Low risk	The author confirmed that the outcome assessors were blinded.
Incomplete outcome data (attrition bias) All outcomes	Low risk	Balanced and explained attrition that likely has no effect on outcome.
Selective reporting (reporting bias)	Unclear risk	The inclusion criteria of ≥ 20 eosinophils/HPF in the full text is higher than that in the protocol (> 14 eosinophils). This retrospectively changed higher cut‐off may have potentially helped the authors achieve a desired response/remission. The author responded that the change was made because the initial "was a criteria used very early in EoE. We wanted to limit as much as possible overlap with GERD often associated with mild eosinophilia and the plan was changed". As this is an appropriate a priori change, we have made the judgment of low risk. Unclear definition of pre‐planned secondary outcomes.
Other bias	Low risk	No major baseline differences between groups. No other concerns.

**Table:** Assa'ad 2011

***Study characteristics***		
Methods	**RCT design and number of study arms:** RCT with 3 arms **Single‐center or multi‐center:** multi‐center **Countries:** USA and UK **Study dates:** 11 September 2006 to 25 November 2008	
Participants	**Active EoE or inactive EoE at beginning of study:** active EoE **EoE definition/diagnostic criteria: "**pediatric patients with isolated EoE, defined as ≥ 20 eos/hpf" ‐ hpf size = 0.3 mm² **Inclusion criteria:** Age 2 to 17 History of inadequate histopathologic response or intolerance to prior EoE therapy **Exclusion criteria:** concurrent eosinophilic gastrointestinal enteropathy based on the baseline endoscopy; gastroesophageal reflux disease or other causes of esophagitis; and presence or history of hypereosinophilic syndromes or collagen vascular disease, vasculitis, allergic drug reaction with peripheral eosinophilia, graft‐versus‐host disease, chronic idiopathic inflammatory bowel disorders, or celiac disease **Age at beginning of study per study group:** mean age (SD) in the 3 study groups (low‐, medium‐, high‐dose) was: 10.4 (4.3), 10.5 (5.2), and 10.4 (4.7) **Sex (m/f) per study group:** sex (m/f) in the 3 study groups (low‐, medium‐, high‐dose) was: 16/3; 14/6; 17/3 **Number randomized per study group:** 19/59, 20/59, 20/59 in the low‐, medium‐, and high‐dose groups, respectively **Number reaching end of study per study group:** 15, 19, 18 in the low‐, medium‐, and high dose groups, respectively	
Interventions	**Study group 1 (control ‐ low‐dose):** 0.55 mg/kg mepolizumab (note: "The lowest dose was expected to be minimally efficacious and serve as a comparator group. A placebo group was not included.") **Study group 2 (medium‐dose):** 2.5 mg/kg **Study group 3 (high‐dose):** 10 mg/kg	
Outcomes	**Primary outcomes of the study:** The proportion of patients with a peak eosinophil count < 5 eos/hpf in the week 12 biopsy Safety, tolerability, and pharmacokinetics of mepolizumab **Secondary outcomes of the study:** Changes in peak and mean eosinophil counts Improvements in histopathologic findings Improvements in endoscopic findings Blood eosinophils counts Frequency and severity of EoE symptoms Proportion of partial responders (eosinophil counts 5 to 19 eos/hpf)	
Notes	**Funding source:** GSK **Conflicts of interest:** All investigators, including all non‐GlaxoSmithKline authors, received funding from the study sponsor (to their institution and not personally) for recruiting patients and conducting the study at their respective sites. Margaret H. Collins received funding (to the institution and not personally) to process and interpret biopsy specimens as the central review pathologist for this study. Amal H. Assa’ad has been an advisory board member and consultant for GlaxoSmithKline. Sandeep K. Gupta has been a consultant for GlaxoSmithKline. Margaret H. Collins has been an advisory board member for GlaxoSmithKline and received funding (to the institution and not personally) to process and/or interpret biopsy specimens as central review pathologist for Ception Therapeutics (now Cephalon) and Meritage Pharmaceuticals and is a consultant for Sunovion. Mike Thomson discloses no conflicts. Amy T. Heath, Deborah A. Smith, Teresa L. Perschy, Cynthia H. Jurgensen, and Hector G. Ortega are employees of GlaxoSmithKline. Seema S. Aceves has been an advisory board member for GlaxoSmithKline and has financial arrangements with Meritage Pharmaceuticals. Editorial support in the form of outline development, collating author comments, grammatical editing, and referencing was provided by Elaine F. Griffin, DPhil, at Evidence Scientific Solutions and was funded by GlaxoSmithKline.	
***Risk of bias***		
**Bias**	**Authors' judgement**	**Support for judgement**
Random sequence generation (selection bias)	Unclear risk	A randomization number was given but no details on how it was generated. Authors contacted for further details in November 2022. No response received.
Allocation concealment (selection bias)	Low risk	Patients were assigned to study treatment in accordance with the randomization schedule generated and maintained by the study sponsor. Each participant was assigned a subject number, a randomization number, and a container number. All study personnel and patients were blinded to study group allocation.
Blinding of participants and personnel (performance bias) All outcomes	Low risk	Participants and personnel were blinded and randomization schedule maintained by the study sponsor. No details on how blinding was achieved, but since the study is stated to be "double‐blind" and the infusions used can be easily masked, we chose to rate this domain as low risk.
Blinding of outcome assessment (detection bias) All outcomes	Unclear risk	The authors state all personnel and patients were blinded but no further details were given. Authors contacted for further details in November 2022. No response received.
Incomplete outcome data (attrition bias) All outcomes	Low risk	Low and balanced attrition that likely has no effect on outcome.
Selective reporting (reporting bias)	High risk	Not all primary outcomes were reported. The pre‐specified outcomes in the trial registration look different than those reported in the paper. Specifically, there are a number of outcomes missing related to exploratory outcomes not related to our review. For the clinical outcomes, the authors did not report means and SDs. For serious and total adverse events, authors did not specify the group in which they occurred. Authors contacted for further details in November 2022. No response received.
Other bias	Low risk	No major baseline differences between groups. No other concerns.

**Table:** Bhardwaj 2017

***Study characteristics***		
Methods	**RCT design and number of study arms:** randomized, double‐blind, placebo‐controlled, cross‐over study. 2 arms followed by washout, then 2 arms. **Single Center:** Penn State College of Medicine, Hershey, Pennsylvania **Countries:** USA **Study dates:** April 2010 and June 2011	
Participants	**Active EoE or inactive EoE at beginning of study:** active EoE **EoE definition/diagnostic criteria:** ≥ 15 eos/hpf at enrollment (not followed for 2 participants) **Inclusion criteria:** Male or female 18 to 65 years of age With a biopsy proven diagnosis of EoE Able and willing to provide consent for repeated endoscopies with esophageal biopsies and blood work as per study protocol **Exclusion criteria:** Participants with suspected or proven inflammatory bowel disease, malignancy, or collagen‐vascular disease Participants who had used oral, inhaled, or swallowed corticosteroids in the past 3 months Participants who were pregnant or breastfeeding Participants who were not able to swallow beclomethasone dipropionate or who were intolerant to the medication Participants with a history of ischemic heart disease, diabetes, or dyslipidemia, unless they had been stable in the past 6 months **Age at beginning of study per study group:** not provided **Sex (m/f) per study group:** Data were reported for participants who completed the study Group 1: beclomethasone dipropionate (BDP) 80 µg 2 puffs 2x/day (4/0) Group 2: placebo (2/3) **Number randomized per study group:** total n = 13, but Group 1 ‐ BDP n = 9, group 2 ‐ placebo n = 9 **Number reaching end of study per study group (numbers of patients):** Group 1 BDP ‐ 4 (pre‐cross‐over) Group 2 placebo ‐ 5 (pre‐cross‐over)	
Interventions	**Study group 1:** inhaler x 8 weeks; beclomethasone dipropionate 80 µg 2 puffs 2 x/day **Study group 2:** inhaler (not entirely clear) x 8 weeks, drug: placebo Matched placebo swallowed 2 puffs twice‐daily	
Outcomes	**Primary outcomes of the study:** number of eosinophils (peak) in esophageal tissue measured at baseline and at the end of each treatment period (mean difference) **Secondary outcomes of the study:** collected non‐validated score Unclear ‐ outcomes not properly defined in the paper but defined in the protocol: "evaluate safety of montelukast in eosinophilic esophagitis"	
Notes	**Funding source:** Foundation of Young Faculty Award of the American College of Allergy, Asthma and Immunology **Conflicts of interest:** none	
***Risk of bias***		
**Bias**	**Authors' judgement**	**Support for judgement**
Random sequence generation (selection bias)	Low risk	Author correspondence confirmed randomization was computer‐generated and performed by a statistician.
Allocation concealment (selection bias)	Low risk	"A physician not involved with the study other than setting up the randomization scheme and medication packets held the randomization key, and did not play any other role in the study to prevent unblinding."
Blinding of participants and personnel (performance bias) All outcomes	Low risk	Author correspondence confirmed participants and personnel were blinded.
Blinding of outcome assessment (detection bias) All outcomes	Low risk	Author correspondence confirmed outcome assessors were blinded.
Incomplete outcome data (attrition bias) All outcomes	Unclear risk	13 randomized, but 4 withdrew consent although unclear from which groups. Authors could not provide further information on this.
Selective reporting (reporting bias)	Unclear risk	Trial has been registered prospectively. The only outcome registered was symptom improvement at 5 months, which differed from the outcomes presented in the final paper. Adverse events not reported and authors did not provide information.
Other bias	Low risk	No obvious baseline imbalance. However, the baseline population did not meet their own inclusion criteria as 2 participants without EoE were included for unclear reasons. Authors responded that all participants had clinicopathological diagnosis at one point. No other concerns.

**Table:** Butz 2014

***Study characteristics***		
Methods	**RCT design and number of study arms:** RCT, 2 arms **Single‐center or multi‐center:** multi‐center **Countries:** USA	
Participants	**Active EoE or inactive EoE at beginning of study:** active **EoE definition/diagnostic criteria:** ≥ 24 eosinophils/high‐power field (HPF) in the proximal/distal esophagus while being treated with a proton pump inhibitor (PPI) for at least 2 months or having a negative pH probe **Inclusion criteria:** Signed informed consent for study by participant, or parent/guardian if the participant is a minor. Assent obtained from all minors 11 years of age and older. Histological findings on esophageal biopsy to include peak eosinophil density ≥ 24 per high‐power field (400x) in the proximal or distal esophagus validated by a pathologist at CCHMC Allergy evaluation including skin‐prick testing with multiple food antigens to ensure elimination diet is not indicated Have undergone a minimum 3 months of elimination diet as indicated by skin‐prick testing without detectable resolution by repeat endoscopy with biopsies demonstrating persistent eosonophilic esophagitis OR participant/parental refusal to follow an elimination diet. If the participant/parent refuses the elimination diet, they are eligible for this study. Treatment with a proton pump inhibitor for at least 2 months (rounded to the nearest month) prior to endoscopy OR failure of histological improvement as defined by < 1 eosinophil per HPF after 2 months (rounded to the nearest month) trial of proton pump inhibitor documented by prior endoscopy. The PPI must be used prior to endoscopy to rule out the possibility of GERD. **Exclusion criteria:** History of poor tolerance to fluticasone propionate defined as multiple episodes of oral candidiasis, hypothalamic‐pituitary‐adrenal axis suppression as evidenced by signs of Cushing syndrome, headaches, or increased respiratory infections during exposure to Flovent An inability to use a metered‐dose inhaler, concurrent or recent use of systemic corticosteroids Comorbid eosinophilic disorders, or a diagnosis of or being at risk for diabetes (type I or II) **Age at beginning of study per study group:** fluticasone 12.2 (3.54 to 26.90); placebo 13.5 (4.10 to 29.80) **Sex (m/f) per study group:** fluticasone 22/6 (79%); placebo 13/1 (93%) **Number randomized per study group:** fluticasone n = 28; placebo n = 14 **Number reaching end of study per study group:** fluticasone n = 23; placebo n = 13	
Interventions	**Study group 1:** placebo **Study group 2:** daily 1760 µg fluticasone propionate (2 x 880 µg)	
Outcomes	**Primary outcomes of the study:** remission at 3 months, remission is considered achieved when the highest eosinophil count per high‐power field (HPF) in all esophageal biopsies is ≤ 1 eosinophil/HPF after 3 months of therapy **Secondary outcomes of the study:** The secondary objectives were to measure safety via cortisol, glucose, and adverse reaction data. The relationship between fluticasone propionate responsiveness and participant age, height, weight, BMI, Z‐score, race, ethnicity, atopic status, compliance, and screening eosinophil level. Atopic status was defined by a personal history of allergic disease (allergic rhinitis, hay fever, atopic dermatitis, eczema, food anaphylaxis, asthma, or positive skin prick tests).	
Notes	**Funding source:** National Institute of Allergy and Infectious Diseases grant(U01AI088806 to M.E.R.), a GlaxoSmithKline grant (109928), and the national center for Research Resources and the National Center for AdvancingTranslational Sciences of the National Institutes of Health (8 UL1 TR000077‐05) **Conflicts of interest:** Marc Rothenberg and Ting Wen are co‐inventors for a pending patent based on the Eosinophilic Esophagitis Diagnostic Panel test described in this article	
***Risk of bias***		
**Bias**	**Authors' judgement**	**Support for judgement**
Random sequence generation (selection bias)	Low risk	Computer‐generated, coded randomization method
Allocation concealment (selection bias)	Low risk	The treatment was centrally allocated.
Blinding of participants and personnel (performance bias) All outcomes	Low risk	The allocation sequence was known only to the central pharmacist at study start. Following the first 3 months of the study, a site pharmacist and a staff member were made aware of assignments to facilitate stratification.
Blinding of outcome assessment (detection bias) All outcomes	Low risk	The authors confirmed in November 2022 that the outcome assessors were blinded.
Incomplete outcome data (attrition bias) All outcomes	Low risk	Balanced and explained attrition that likely has no effect on outcome.
Selective reporting (reporting bias)	Unclear risk	The study protocol is available. An EoE symptom score was used but scores were not reported.
Other bias	Low risk	No major baseline differences between groups. No other concerns.

**Table:** Clayton 2014

***Study characteristics***		
Methods	**RCT design and number of study arms:** RCT **Single‐center or multi‐center:** dual‐center, University of Utah Hospital and Primary Children’s Hospital, Salt Lake City, UT, both referral centers **Countries:** USA **Study dates:** NR	
Participants	**Active EoE or inactive EoE at beginning of study:** active **EoE definition/diagnostic criteria:** did not specifically define EoE in the text. Defined in the protocol as "Eosinophilic esophagitis (EE) is an increasingly recognized condition characterized by dysphagia, food impaction or other obstructive esophageal symptoms in children and young adults." **Inclusion criteria:** Adults (> 18 years) (3 participants in the omalizumab trial aged 15 to 17) Active eosinophilic esophagitis ≥ 15 eosinophils/hpf in esophageal biopsy specimen Not responsive to maximal‐dose proton pump inhibitors Not currently being treated with steroids and had failed steroid therapy Serum IgE 30 to 700 IU/mL Acceptable medical history, physical exam, and laboratory test results, specifically no history of bleeding diathesis, significant cardio‐pulmonary disease, or other contraindications to upper endoscopy Inclusion for controls – healthy adults > 18 years with no medical disease **Exclusion criteria:** Not mentioned in the full text but copied below from the protocol: Need for esophageal dilation at enrollment due to food impaction or inability to pass endoscope The inability of participant to provide informed consent (if ages 18 to 60), or the inability of children (ages 12 to 17) to provide assent History of esophagogastric surgery Presence of other esophageal pathology that could account for patients' symptoms including eosinophil infiltration due to gastroesophageal reflux disease (GERD) Incarceration Pregnancy Women of childbearing age that are not using the contraception method(s) Patients with elevated serum IgE levels for reasons other than atopy Patients taking cromolyn sodium or nedocromil sodium within 1 month of visit 1 Patients taking oral or topical corticosteroids within one month of visit 1 Patients taking leukotriene receptor inhibitors within one month of visit 1 Patients with a severe medical condition(s) that in the view of the investigator prohibit participation in the study Patients with a history of noncompliance to medical regimens or who were considered potentially unreliable Use of any other investigational agent in the last 30 days Patients with a known hypersensitivity to any ingredient of rhuMAb‐E25, study rescue medication Patients with Barrett's esophagus excluded if found endoscopically or pathologically at biopsy Currently treated with omalizumab or treated with omalizumab within the past 6 months. **Age at beginning of study per study group:** mean (range; no SD given) omalizumab 32 (16 to 52), placebo 28 (15 to 39) **Sex (m/f) per study group:** omalizumab m/f = 13/3, placebo m/f = 11/3 **Number randomized per study group:** omalizumab n = 16, placebo n = 14 **Number reaching end of study per study group:** omalizumab n = 16, placebo n = 14	
Interventions	**Study group 1:** placebo (saline) subcutaneous injection every 2 to 4 weeks for 16 weeks **Study group 2:** omalizumab subcutaneous injection every 2 to 4 weeks for 16 weeks, using a weight and serum IgE‐based dosing protocol	
Outcomes	**Primary outcomes of the study:** primary endpoint was reducing esophageal biopsy eosinophil content **Secondary outcomes of the study:** a secondary endpoint was a reduction in dysphagia symptom	
Notes	**Funding**: supported by a Castell grant (K.A.P.). Novartis funded, but did not interpret or primarily design, the omalizumab trial. **Conflict of interests**: nothing to disclose	
***Risk of bias***		
**Bias**	**Authors' judgement**	**Support for judgement**
Random sequence generation (selection bias)	Low risk	Computer‐generated randomization method.
Allocation concealment (selection bias)	Low risk	Placebo participants were given injections of material seemingly identical to the omalizumab.
Blinding of participants and personnel (performance bias) All outcomes	Unclear risk	No mention in manuscript or supplemental material of all who were or were not blinded.
Blinding of outcome assessment (detection bias) All outcomes	Low risk	"All statistical comparisons were based on blinded analysis of number‐coded slides, tissue homogenates, sera, or of subjects for whom treatment status was known only by a research pharmacist."
Incomplete outcome data (attrition bias) All outcomes	Low risk	All randomized patients completed the study and their data were included in the analysis.
Selective reporting (reporting bias)	Unclear risk	The authors described their primary endpoint as a reduction in eosinophil count per high‐power field. This matches the planned analysis. The authors provide P values for secondary, though not the primary outcome. The authors were contacted for clarification without response.
Other bias	Low risk	No major baseline differences between groups. No other concerns.

**Table:** De Rooij 2022

***Study characteristics***		
Methods	**RCT design and number of study arms:** open‐label RCT; 2 arms **Single‐center or multi‐center:** single‐center; Amsterdam UMC motility center **Countries:** Netherlands **Study dates:** December 2017 to January 2020	
Participants	**Active EoE or inactive EoE at beginning of study? (and numbers if mixed, per IG/CG):** active **EoE definition/diagnostic criteria:** symptoms of esophageal dysfunction (Straumann Dysphagia Instrument score of ≥ 1) and ≥ 15 eos/HPF on baseline biopsy **Inclusion criteria:** Symptoms of esophageal dysfunction (Straumann Dysphagia Instrument score of ≥ 1) and ≥ 15 eos/HPF on baseline biopsy **Exclusion criteria:** Severe comorbidity scored as the ASA physical classification system class 4 or higher Recent history of GI cancer or major GI surgery Inability to stop anti‐inflammatory drugs **Age at beginning of study per study group:** NR **Sex (m/f) per study group:** NR **Number randomized per study group:** four food elimination diet (FFED) n = 20; **:** four food elimination diet + amino acid formula (FFED + AAF) n = 21 **Number reaching end of study per study group (numbers of patients):** four food elimination diet (FFED) n = 20; **:** four food elimination diet + amino acid formula (FFED + AAF) n = 20	
Interventions	**Study group 1:** four food elimination diet (FFED) **Study group 2:** four food elimination diet + amino acid formula (FFED + AAF)	
Outcomes	**Primary outcomes of the study:** change in peak eos count (PEC) **Secondary outcomes of the study:** histologic remission (< 15 eos/HPF), endoscopic signs (EREFS), Straumann Dysphagia Instrument measure, EoEQoL survey, clinical and nutritional outcomes, diet feasibility, adherence, weight loss, BMI	
Notes	**Funding source:** NR **Conflicts of interest:** NR	
***Risk of bias***		
**Bias**	**Authors' judgement**	**Support for judgement**
Random sequence generation (selection bias)	Unclear risk	"Patients were randomized [...] using a blocked randomization protocol with sealed envelopes". There is no further information regarding randomization.
Allocation concealment (selection bias)	Low risk	"Patients were randomized [...] using a blocked randomization protocol with sealed envelopes".
Blinding of participants and personnel (performance bias) All outcomes	Unclear risk	The manuscript mentioned "blinded personnel" but unclear how. The authors were contacted in November 2020. No response.
Blinding of outcome assessment (detection bias) All outcomes	Unclear risk	The manuscript mentioned "blinded personnel" but unclear how. The authors were contacted in November 2020. No response.
Incomplete outcome data (attrition bias) All outcomes	Low risk	Balanced and explained attrition that likely has no effect on outcome.
Selective reporting (reporting bias)	Low risk	Trial registry is available and all pre‐specified outcomes of interest were reported in the pre‐specified way.
Other bias	Low risk	No major baseline differences between groups. No other concerns.

**Table:** Dellon 2012

***Study characteristics***		
Methods	**RCT design and number of study arms:** randomized, prospective, open‐label, parallel‐arm **Single‐center or multi‐center?** single‐center, University of North Carolina (UNC) Center **Countries:** USA **Dates**: March 2010 and May 2011	
Participants	**Active EoE or inactive EoE at beginning of study? (and number if mixed, per IG/CG): a**ctive **EoE definition/diagnostic criteria:** included participants reported symptoms of esophageal dysfunction and had persistent esophageal eosinophilia (≥ 15 eosinophils in one high‐power field) after 8 weeks of treatment with twice‐daily proton pump inhibitor **Inclusion criteria:** 18 years or older with an incident diagnosis of eosinophilic esophagitis Symptoms of esophageal dysfunction Persistent esophageal eosinophilia (³ 15 eosinophils in one high‐power field microscopy field (eos/hpf; hpf area = 0.24 mm²) after 8 weeks of treatment with a twice‐daily proton pump inhibitor **Exclusion criteria:** Proton pump inhibitor responsive esophageal eosinophilia Previous diagnosis of eosinophilic esophagitis Previous allergic reaction to steroid medications Current use of systemic steroids Previous treatment with topical steroids, had Barrett’s esophagus or previous esophageal surgery, had an inability to read or understand English, or were pregnant **Age at beginning of study per study group:** mean ± SD Budesonide, nebulized: 34.9 ± 7.3 Budesonide, oral viscous: 34.4 ± 7.5 **Sex (m/f) per study group:** Budesonide, nebulized: 8/5 Budesonide, oral viscous: 7/5 **Number randomized per study group:** total n = 25 Budesonide, nebulized: 13/25 Budesonide, oral viscous: 12/25 **Number reaching end of study per study group:** Budesonide, nebulized: 11/13 Budesonide, oral viscous: 11/12	
Interventions	**Group 1 (NEB)**: nebulized/swallowed budesonide solution (1 mg/2 mL) continuously swallowed for over 5 minutes until the dose was depleted **Group 2 (OVB)**: viscous/swallowed budesonide solution (1 mg/2 mL) was mixed with 5 g of sucralose into a viscous slurry and swallowed	
Outcomes	**Primary outcomes** Esophageal eosinophilia as measured by the eosinophil count Symptom of dysphagia as measured by the Mayo Dysphagia Questionnaire ‐ 30 days **Secondary outcomes** Mucosal contact time as measured by nuclear scintigraphy Levels of histologic response on esophageal biopsy Endoscopic findings of EoE Adrenal insufficiency as measured by a standard cortisol stimulation test	
Notes	**Funding source:** This study was sponsored by AstraZeneca **Conflicts of interest:** No potential conflicts of interest for any of the authors were reported for this study. The funding organizations had no role in the following: design and conduct of the study; collection, management, analysis, and interpretation of the data; and drafting of the manuscript.	
***Risk of bias***		
**Bias**	**Authors' judgement**	**Support for judgement**
Random sequence generation (selection bias)	Low risk	Computer‐generated randomization method.
Allocation concealment (selection bias)	Low risk	Treatment was centrally allocated.
Blinding of participants and personnel (performance bias) All outcomes	High risk	This was an open‐label study.
Blinding of outcome assessment (detection bias) All outcomes	Low risk	Biopsies were masked and provided to the study pathologists for analysis.
Incomplete outcome data (attrition bias) All outcomes	Low risk	The CONSORT flow diagram in the supplementary information states 1 patient discontinued from the NEB group (13 to 1 = 12). However, 11 completed the study with no information on n = 1. The author confirmed in November 2022 that 1 patient was lost to follow‐up and 1 discontinued. There were different patients, so 11 is correct.
Selective reporting (reporting bias)	Low risk	Trial registry is available and all pre‐specified outcomes of interest were reported in the pre‐specified way.
Other bias	Low risk	No major baseline differences between groups. No other concerns.

**Table:** Dellon 2017

***Study characteristics***		
Methods	**RCT design and number of study arms**: randomized, double‐blind, placebo‐controlled, parallel‐group; 2 arms **Single‐center or multi‐center:** multi‐center **Countries:** USA **Study dates**: July 2012 to October 2014	
Participants	**Active EoE or inactive EoE at beginning of study:** active **EoE definition/diagnostic criteria:** symptoms of esophageal dysfunction and at least 15 intra‐epithelial eosinophils per high‐power field (eos/hpf) (hpf area: 0.3 mm²) after an 8‐week, high‐dose (refers to a total daily dose, which could be administered as a once‐ or twice‐daily dosing regimen), proton pump inhibitor (PPI) trial using any approved PPI. The PPI trial was either historical or could have been performed during the screening period of this study. **Inclusion criteria**: Patients with EoE were required to have at least 15 eos/HPF from at least 2 esophageal levels on screening endoscopy, at least 4 days with symptoms of dysphagia over the last 2 weeks of a 4‐week blinded placebo run‐in period, and at least 70% compliance with a daily symptom diary Males and females, age 11 to 40 Histologic evidence of EoE History of clinical symptoms of EoE including dysphagia Willing to continue with dietary, environmental, or medical therapy Ability to read and understand English Written consent **Exclusion criteria**: Older patients who are more likely to have fibrostenotic disease and typically are not amenable to anti‐inflammatory treatment alone Other potential causes of esophageal eosinophilia had also been excluded Non‐EoE gastrointestinal diseases, including eosinophilic gastroenteritis/colitis, inflammatory bowel disease, celiac disease, *Helicobacter pylori* infection, esophageal candidiasis (defined based on investigator discretion), or esophageal varices; diseases causing systemic eosinophilia; or esophageal stricture on screening endoscopy that precluded passage of an adult upper endoscope Gastroesophageal reflux disease and erosive esophagitis were not formal exclusion criteria, but patients with esophageal eosinophilia related to gastroesophageal reflux disease were excluded, as based on PPI‐responsive eosinophilia. Other exclusion criteria were: use of corticosteroids (topical or systemic) in the 4 weeks preceding the screening endoscopy; use of immunomodulatory therapy in the 8 weeks preceding the screening endoscopy; change in the dosing regimen of PPIs, allergy medications, or inhaled corticosteroids; pregnancy; and medical instability. **Age at beginning of study per study group**: budesonide oral suspension (BOS) = 22.3 ± 7.9; placebo = 20.8 ± 7.5 **Sex (m/f) per study group:** Budesonide oral suspension = 35/16; placebo = 29/13 **Number randomized per study group:** Budesonide oral suspension = 51; placebo = 42 **Number reaching end of study per study group:** Budesonide oral suspension = 49/51; placebo = 38/42	
Interventions	**Study group 1:** placebo suspension **Study group 2:** budesonide oral suspension (BOS) 2 mg twice‐daily (given as 10 mL, once in the morning after breakfast and once in the evening before bedtime to provide a total daily dose of 4 mg)	
Outcomes	**Primary outcomes of the study:** Change in the DSQ Histologic response, defined as eos ≤ 6/hpf **Secondary outcomes of the study**: endoscopic findings and safety	
Notes	**Funding source**: This study was sponsored by Meritage Pharma, Inc, now a part of the Shire group of companies. Meritage Pharma, Inc contributed to the design and conduct of the study, collection and management of the data, and reviewed the manuscript for medical accuracy. Approval of the manuscript and the decision to submit the manuscript for publication were the responsibility of the authors. **Conflicts of interest:** The authors disclose the following: Evan S. Dellon has received research funding from Meritage, Receptos, and Regeneron; and is a consultant forAptalis, Banner Life Sciences, Novartis, Receptos, Regeneron, and Roche.David A. Katzka has received research funding from Meritage. Margaret H.Collins has received research funding from Meritage, Receptos, Regeneron,and Biogen Idec. Mohamed Hamdani is an employee and stockholder of Shire. Sandeep K. Gupta has received research funding from Meritage. IkuoHirano has received research funding from Meritage, and is a consultant forMeritage, Receptos, and Regeneron.	
***Risk of bias***		
**Bias**	**Authors' judgement**	**Support for judgement**
Random sequence generation (selection bias)	Low risk	"The randomization schedule was generated by SynteractHCR, Inc. and was verified for accuracy using strict quality‐control procedures".
Allocation concealment (selection bias)	Low risk	The active study medication and placebo were dispensed in identical amber glass bottles to maintain the blind. However, the authors did not give explicit details on the similarity of both suspensions. The author was contacted in November 20202 and confirmed the allocators were not involved in any other part of the study.
Blinding of participants and personnel (performance bias) All outcomes	Low risk	"Participants, investigators, the sponsor, study site personnel, and the central pathologist were blinded to patients’ treatment, until after all patients had completed the treatment period and the database was locked."
Blinding of outcome assessment (detection bias) All outcomes	Low risk	"Participants, investigators, the sponsor, study site personnel, and the central pathologist were blinded to patients’ treatment, until after all patients had completed the treatment period and the database was locked."
Incomplete outcome data (attrition bias) All outcomes	Low risk	Balanced and explained attrition that likely has no effect on outcome.
Selective reporting (reporting bias)	Low risk	Trial registry is available and all pre‐specified outcomes of interest were reported in the pre‐specified way.
Other bias	Low risk	No major baseline differences between groups. No other concerns.

**Table:** Dellon 2019

***Study characteristics***		
Methods	**RCT design and number of study arms:** RCT, 2 arms **Single‐center or multi‐center?** single‐center **Countries:** USA **Study dates:** 2014 to 2018	
Participants	**Active EoE or inactive EoE at beginning of study:** active EoE **EoE definition/diagnostic criteria:** dysphagia or other symptoms of esophageal dysfunction, > or equal to 15 eos/hpf in at least one field after 8 weeks of treatment with twice‐daily PPI and other causes of esophageal eosinophilia excluded **Inclusion criteria:** dysphagia or other symptoms of esophageal dysfunction, > or equal to 15 eos/hpf in at least one field after 8 weeks of treatment with twice‐daily PPI and other causes of esophageal eosinophilia excluded **Exclusion criteria:** concomitant EG, swallowed/topical steroids for EoE or systemic steroids within 4 weeks before baseline endoscopy, inability to pass 9 mm upper endoscope due to narrowing, previous esophageal surgery, esophageal or gastric cancer, esophageal varices, inability to stop anticoagulation or active GI bleeding, medical instability precluding endoscopy, inability to read or understand English, or pregnancy **Age at beginning of study per study group:** mean age ± SD budesonide, oral viscous: 36.2 ± 19.1 and mean age fluticasone 39.0 ± 14.5 **Sex (m/f) per study group**: budesonide, oral viscous 40 males/25 females and fluticasone 44 M/20 F **Number randomized per study group:** 65 to budesonide, oral viscous and 64 to fluticasone **Number reaching end of study per study group:** 1 did not receive intervention in each group and 8 lost in follow‐up in each group	
Interventions	**Study group 1:** budesonide, oral viscous (1 mg/4 mL budesonide with 10 g sucralose twice‐daily) + placebo inhaler **Study group 2:** fluticasone (220 µg, 4 puffs (880 µg) twice‐daily) + placebo slurry	
Outcomes	**Primary outcomes of the study:** Primary: post‐treatment peak eos (eos/hpf; hpf 0.24 mm²) Co‐primary: dysphagia score by the Dysphagia Symptom Questionnaire **Secondary outcomes of the study:** Secondary: EREFS, levels of histologic response (< 15 eos/hpf, < 5 eos/hpf, < 1 eos/hpf), EoE Symptom Activity Index (EEsAI) Also: medication compliance and adverse events	
Notes	**Funding source:** This study was supported by National Institutes of Health (NIH) R01 DK101856, and used resources from University of North Carolina (UNC) Center for GI Biology and Disease (NIH P30 DK034987) and the UNC Translational Pathology Lab, which is supported in part by grants from the National Cancer Institute (2‐P30‐CA016086‐40), National Institute of Environmental Health Sciences (2‐P30ES010126‐15A1), University Cancer Research Fund, and North Carolina Biotechnology Center (2015‐IDG‐1007) **Conflicts of interest:** These authors disclose the following: Dr Dellon has received research funding from Adare, Allakos, GSK, Meritage, Miraca, Nutricia, Celgene/Receptos, Regeneron, and Shire; has received consulting fees from Adare, Alivio, Allakos, AstraZeneca, Banner, Calypso, Enumeral, EsoCap, Celgene/Receptos, GSK, Regeneron, Robarts, Shire, and educational grants from Allakos, Banner, and Holoclara. The remaining authors disclose no conflicts.	
***Risk of bias***		
**Bias**	**Authors' judgement**	**Support for judgement**
Random sequence generation (selection bias)	Low risk	Computer‐generated randomization method.
Allocation concealment (selection bias)	Low risk	Medications were premixed by the pharmacy and dispensed such that placebo and active treatments were identical.
Blinding of participants and personnel (performance bias) All outcomes	Low risk	There was one unblinded study pharmacist who dispensed the study medications.
Blinding of outcome assessment (detection bias) All outcomes	Low risk	"Subjects, investigators, endoscopists, statisticians, and study staff were all masked as to treatment allocation".
Incomplete outcome data (attrition bias) All outcomes	Low risk	Balanced and explained attrition that likely has no effect on outcome. Within the CONSORT diagram, the authors do not fully explicate attrition per treatment arm. The author was contacted in November 2022 and confirmed attrition details.
Selective reporting (reporting bias)	Low risk	Trial registry is available and all pre‐specified outcomes of interest were reported in the pre‐specified way.
Other bias	Low risk	No major baseline differences between groups. No other concerns.

**Table:** Dellon 2021b

***Study characteristics***		
Methods	**RCT design and number of study arms:** RCT with 3 arms, NCT02736409 (extension of NCT02605837) **Single‐center or multi‐center:** multi‐center **Countries:** 60 US sites **Study dates:** 2016 to 2019	
Participants	**Active EoE or inactive EoE at beginning of study:** mixed because the beginning of this study was a continuation of an induction treatment study **EoE definition/diagnostic criteria:** based on Hirano et al (2022). Histologic EoE with > 15 eos/hpf from at least 2 levels of the esophagus and dysphagia (using DSQ) on at least 4 days in 2 consecutive weeks including the 2 weeks before randomization **Inclusion criteria:** patients who completed the induction study. For that study, patients willing and able to maintain dietary/environmental therapy and medical regimens in place at screening. **Inclusion criteria:** Participant completed SHP621‐301 induction study Participant is able to provide written informed consent (participant, parent or legal guardian and, as appropriate, participant assent) to participate in the study before completing any study‐related procedures Participant is male or female aged 11 to 55 years, inclusive, at time of consent for SHP621‐301 study Participant is willing and able to continue any dietary therapy, environmental therapy, and/or medical regimens (including gastric acid suppression; see exclusions below) in effect at the screening visit (Visit 0). There should be no changes to these regimens during study participation. All female participants must have a negative serum pregnancy test (beta‐human chorionic gonadotropin [β‐hCG)) prior to enrollment into the study. Females of childbearing potential must agree to continue acceptable birth control measures (e.g. abstinence, stable oral contraceptives, or double‐barrier methods) throughout study participation and for 30 days following the last dose of investigational product. Participant is willing and has an understanding and ability to fully comply with study procedures including DSQ compliance (completed the DSQ on ≥ 70% of days in any 2 consecutive weeks of the screening period)and restrictions defined in this protocol (implicit) Patients who were defined as having undergone remission (< 7 eosinophils per high‐power field (eos/hpf) and ≥ 30% reduction in the Dysphagia Symptom Questionnaire score) **Exclusion criteria:** patients with high‐grade stricture. Also changes in diet or medications; use of immunomodulatory therapy, swallowed topical corticosteroids, or systemic corticosteroids, or P450 inhibitors; use of inhaled or nasal corticosteroids with unstable dosing; changed dosing regimen of PPI, H2 antagonists, antacids or leukotriene inhibitors; use of pure liquid or the 6‐food elimination diet; and unresponsive esophageal or oropharyngeal candidiasis. Participant has changes in medications that could affect the study or diet in the weeks since the final treatment evaluation visit (Visit 4) of the SHP621‐301 study. Participant using immunomodulatory therapy since the final treatment evaluation visit (Visit 4) of the SHP621‐301 study or anticipated use of immunomodulatory therapy during the treatment period (except for any ongoing regimen of allergy shots); any temporary use (≤ 7 days) or initiation of new steroid treatment during the study should be documented and discussed with the medical monitor prospectively but cannot occur within 4 weeks of scheduled EGDs. Participant using swallowed topical corticosteroid for EoE or systemic corticosteroid for any condition since the final treatment evaluation visit (Visit 4) of the SHP621‐301 study or anticipated use during the treatment period; any temporary use (≤ 7 days) or initiation of new steroid treatment during the study should be documented and discussed with medical monitor prospectively but cannot occur within the 4 weeks of the scheduled EGDs. Participant on inhaled or intranasal steroids and not on a stable dose between the baseline visit (Visit 1) of the SHP621‐301 study and the screening EGD of this study. Participant has initiated, discontinued, or changed dosage regimen of proton pump inhibitors (PPIs), H2 antagonists, antacids, antihistamines, or leukotriene inhibitors for any condition (such as gastroesophageal reflux disease, asthma or allergic rhinitis) since the final treatment evaluation visit (Visit 4) of the SHP621‐301 study or anticipated changes in the use of such medications during the treatment period. Participant using Cytochrome P450 3A4 inhibitors (e.g. ketoconazole, grapefruit juice) since the final treatment evaluation visit (Visit 4) of the SHP621‐301 study or anticipated use of such medications during the treatment period. Participant has an appearance on screening EGD of an esophageal stricture (high‐grade), as defined by the presence of a lesion that does not allow passage of a diagnostic adult upper endoscope (e.g. with an insertion tube diameter of > 9 mm). Participant is on a pure liquid diet or the 6‐food elimination diet. Participant has presence of esophageal varices at the EGD at the final treatment evaluation visit (Visit 4) of the SHP621‐301 study. Participant has any current disease of the gastrointestinal tract, aside from EoE, including eosinophilic gastritis, enteritis, colitis, or proctitis, inflammatory bowel disease, or celiac disease. Participant has other diseases causing or associated with esophageal eosinophilia, including hypereosinophilic syndrome, collagen vascular disease, vasculitis, achalasia, or parasitic infection. Participant has oropharyngeal or esophageal candidiasis that failed to respond to previous treatment. Diagnosis with oropharyngeal or esophageal candidiasis at or since the final treatment evaluation visit (Visit 4) of the SHP621‐301 study is not an exclusion as long as the participant received treatment for candidiasis and is expected to respond to treatment. Participant has acute or chronic infection or immunodeficiency condition, including tuberculosis, fungal, bacterial, viral/parasite infection, ocular herpes simplex, or chicken pox/measles. Participant has upper gastrointestinal bleeding identified in the EGD at the final treatment evaluation visit (Visit 4) of the SHP621‐301 study or since the final treatment evaluation visit (Visit 4) of the SHP621‐301 study. Participant has evidence of active infection with *Helicobacter pylori*. Participant has evidence of unstable asthma since the final treatment evaluation visit (Visit 4) of the SHP621‐301 study. Participant is female and pregnant or nursing. Participant has a history of intolerance, hypersensitivity, or idiosyncratic reaction to budesonide (or any other corticosteroids), or to any other ingredients of the study medication. Participant has a history or high risk of noncompliance with treatment or regular clinic visits. Participant is on sucralfate or anticipates using sucralfate during the treatment period. **Age at beginning of study per study group:** 1) Arm 1 budesonide oral suspension (BOS) group: 36.8 (14.1) and placebo group: 36.1 (11.7); 2) Arm 2: 33.1 (12.0); and 3) Arm 3: 33.5 (12.6) **Sex (m/f) per study groups:** 1) Arm 1 BOS group 14 M/11 F and placebo group 16 M/7 F; 2) Arm 2 had 64 M/42 F; and 3) Arm 3 had 39 M/26 F **Number randomized per study group:** 1) 48 induction full responders were in Arm 1 and underwent randomized withdrawal (n = 25 randomized to continue BOS and n = 23 randomized to placebo); 2) For arm 2, 106 induction partial responders and non‐responders received BOS; and 3) For arm 3, 65 induction placebo patients received BOS **Number reaching end of study per study group:** for safety analysis, any patient taking at least one dose and for per protocol analysis, patients without significant deviation from study protocol. For per protocol analysis (Arm 1) participants who continued BOS, n = 18 completed and of the participants who changed to placebo, n = 18 completed. Seven patients relapsed and reinitiated BOS (from placebo group)	
Interventions	**Study group 1 of Arm 1:** full responders who continued budesonide oral suspension (continuation of BOS in previous RCT) **Study group 2 of Arm 1:** full responders who changed to placebo (change from BOS in previous RCT)	
Outcomes	**Primary outcomes of study:** proportion of induction full responders (BOS or placebo) who experienced histologic and dysphagia symptom relapse **Secondary outcomes of study:** Proportions of induction partial responder and non‐responders who achieved full responses at week 36 (52 weeks total of BOS) Measured at weeks 12 and 36, proportions of induction full responders who achieved histologic responses (< 1, < 6, and < 15), additional dysphagia symptom responses, and maintained full responses. Percentage reductions in DSQ scores as changes from the induction and extension study baselines. Also included: change in DSQ scores, peak eosinophil counts, and EREFS from the extension study baseline to weeks 12 and 36	
Notes	**Funding source:** Shire ViroPharma, Inc of Takeda Pharmaceuticals **Conflicts of interest:** The authors disclose the following: Evan S. Dellon has received research funding from Adare Pharmaceuticals, Allakos, AstraZeneca, GlaxoSmithKline, Meritage Pharma, Inc, Miraca Life Sciences, Nutricia, Receptos/Celgene,Regeneron Pharmaceuticals, and Shire, a Takeda Company, and is a consultant for Abbott Laboratories, Adare Pharmaceuticals, Aimmune Therapeutics, Allakos, Amgen, Arena Pharmaceuticals, AstraZeneca, Biorasi,Calypso Biotech, Celldex Therapeutics, Inc, EsoCap Biotech, GlaxoSmithKline, Gossamer Bio, Lilly, Parexel/Calyx Clinical Trial Solutions,Receptos/Celgene, Regeneron Pharmaceuticals, Robarts Clinical Trials, Inc/Alimentiv, Inc, Salix Pharmaceuticals, Sanofi, and Shire, a Takeda Company, and has received educational grants from Allakos, Banner Life Sciences, and Holoclara; Margaret H. Collins has received research funding from MeritagePharma, Inc, Receptos/Celgene, Regeneron Pharmaceuticals, and Shire, a Takeda Company, and is a consultant for Allakos, Arena Pharmaceuticals, AstraZeneca, Calypso Biotech, EsoCap Biotech, GlaxoSmithKline, Receptos/Celgene, Regeneron Pharmaceuticals, Robarts Clinical Trials, Inc/Alimentiv,Inc, and Shire, a Takeda Company; David A. Katzka has received research funding from Shire, a Takeda Company, and a consulting fee from Receptos/Celgene; Vincent A. Mukkada has received research funding from Meritage Pharma, Inc, and Shire, a Takeda Company, and is a consultant for Shire, a Takeda Company; Gary W. Falk has received research funding from Adare Pharmaceuticals, Allakos, Lucid, Receptos/Celgene, Regeneron Pharmaceuticals, and Shire, a Takeda Company, and is a consultant for Adare Pharmaceuticals, Allakos, Bristol Myers Squibb, Lucid, Regeneron Pharmaceuticals, and Shire, a Takeda Company; Robin Morey, Bridgett Goodwin, Nirav K. Desai, and James Williams are employees of Takeda Development Center Americas, Inc, and stockholders of Takeda Pharmaceutical Company Limited; Jessica D. Eisner was an employee and stockholder of Takeda Pharmaceuticals USA, Inc, and a stockholder of Takeda Pharmaceutical Company Limited, at the time of the study; Lan Lan was an employee of Takeda Development Center Americas, Inc, and a stockholder of Takeda Pharmaceutical Company Limited, at the time of the study; and Ikuo Hirano has received research funding from Adare Pharmaceuticals, Allakos, Arena Pharmaceuticals, AstraZeneca, Meritage Pharma, Inc, Receptos/Celgene, Regeneron Pharmaceuticals, and Shire, a Takeda company, and is a consultant for Adare Pharmaceuticals, Allakos, Arena Pharmaceuticals, AstraZeneca, EsoCap Biotech, Gossamer Bio, Lilly, MeritagePharma, Inc, Receptos/Celgene, Regeneron Pharmaceuticals, and Shire, aTakeda Company.	
***Risk of bias***		
**Bias**	**Authors' judgement**	**Support for judgement**
Random sequence generation (selection bias)	Low risk	This was a follow‐up study. For the initial induction study, randomization was performed centrally via "Interactive Web‐based Response System". Full responders after induction were "randomized" to continue active treatment or to placebo. The details of the second randomization process were not stated in the paper or supplemental information.
Allocation concealment (selection bias)	Low risk	The author was contacted in November 2022 and confirmed central allocation.
Blinding of participants and personnel (performance bias) All outcomes	Low risk	Study sites and teams were blinded but no additional details were provided.
Blinding of outcome assessment (detection bias) All outcomes	Low risk	Study sites and teams were blinded. There was a separate analysis team that was not blinded.
Incomplete outcome data (attrition bias) All outcomes	Low risk	No major attrition imbalances or imbalances in reasons for attrition that could have impacted our outcomes, based on the flow diagram.
Selective reporting (reporting bias)	Low risk	Trial registry is available and all pre‐specified outcomes of interest were reported in the pre‐specified way.
Other bias	Low risk	No major baseline differences between groups. No other concerns.

**Table:** Dellon 2022

***Study characteristics***		
Methods	**RCT design and number of study arms:** part A of a 3‐part, randomized, placebo‐controlled phase 3 study, 2 arms **Single‐center or multi‐center:** multi‐center; 95 study locations worldwide **Countries:** US, Australia, Belgium, Canada, France, Germany, Italy, Netherlands, Spain, Sweden, Switzerland, and the UK **Study dates:** 24 September 2018 to 9 September 2021	
Participants	**Active EoE or inactive EoE at beginning of study:** active **EoE definition/diagnostic criteria:** "A documented diagnosis of EoE by endoscopic biopsy." **Inclusion criteria:** A documented diagnosis of EoE by endoscopic biopsy Baseline endoscopic biopsies with a demonstration on central reading of intraepithelial eosinophilic infiltration History (by patient report) of an average of at least 2 episodes of dysphagia (with intake of solids) per week in the 4 weeks prior to screening **Exclusion criteria:** Body weight ≤ 40 kg Prior participation in a dupilumab clinical trial, or past or current treatment with dupilumab Initiation or change of a food‐elimination diet regimen or re‐introduction of a previously eliminated food group in the 6 weeks prior to screening. Other causes of esophageal eosinophilia or the following conditions: hypereosinophilic syndrome and eosinophilic granulomatosis with polyangiitis (Churg‐Strauss syndrome) Active *Helicobacter pylori* infection History of achalasia, Crohn's disease, ulcerative colitis, celiac disease, and prior esophageal surgery Any esophageal stricture unable to be passed with a standard, diagnostic, 9 mm to 10 mm upper endoscope or any critical esophageal stricture that requires dilation at screening History of bleeding disorders or esophageal varices Pregnant or breastfeeding women, or women planning to become pregnant or breastfeed during the study **Age at beginning of study per study group:** not stated **Sex (m/f) per study groups:** not stated **Number randomized per study group:** 42 patients treated with dupilumab and 39 patients treated with placebo **Number reaching end of study per study group:** Dupilumab 300 mg weekly: 42 (100%) Placebo: 39 (100%)	
Interventions	**Study group 1:** placebo **Study group 2:** dupilumab 300 mg weekly	
Outcomes	**Primary outcomes of study:** this was included in another study Proportion of patients achieving a peak esophageal intraepithelial eosinophil (eos) count of < 6 eos/hpf Absolute change from baseline in Dysphagia Symptom Questionnaire (DSQ) score **Secondary outcomes of study:** dupilumab's effect versus placebo on HRQoL and symptom burden Absolute change from baseline in EoE histologic scoring system (EoEHSS) mean grade and stage scores Absolute change in total EoE Endoscopic Reference Score (EREFS) Proportion of patients achieving a peak eos count of < 15 eos/hpf	
Notes	**Funding source:** Research sponsored by Sanofi and Regeneron Pharmaceuticals, Inc. **Conflicts of interest:** Dr. Dellon ‐ Consultant: Abbott, Adare, Aimmune, Allakos, Amgen, Arena, AstraZeneca, Biorasi, Calypso, Eli Lilly, EsoCap, Gossamer Bio, GlaxoSmithKline, Parexel, Receptos/Celgene/Bristol Myers Squibb, Regeneron Pharmaceuticals, Inc., Robarts, Salix, Shire/Takeda; research funding: Adare, Allakos, GlaxoSmithKline, Meritage, Miraca, Nutricia, Receptos/Celegene/Bristol Myers Squibb, Regeneron Pharmaceuticals, Inc., Shire/Takeda; educational grant: Allakos, Banner, Holoclara. Dr. Rothenberg ‐ Consultant: Allakos, AstraZeneca, Bristol Myers Squibb, ClostraBio, Pulm One, Spoon Guru; equity interest: ClostraBio, Pulm One, Spoon Guru; royalties from reslizumab: Teva Pharmaceuticals; royalties from PEESSv2: Mapi Research Trust; royalties: UpToDate; inventor of patents owned by Cincinnati Children’s Hospital. Dr. Collins ‐ consultant: Allakos, Arena, AstraZeneca, Bristol Myers Squibb, Calypso, Esocap, GlaxoSmithKline, Regeneron Pharmaceuticals, Inc.,Shire; research funding: Receptos/Bristol Myers Squibb, Regeneron Pharmaceuticals, Inc., Shire. Dr. Hirano ‐ consultant: Adare, Receptos/Bristol Myers Squibb, Regeneron Pharmaceuticals, Inc., Shire; research funding: Meritage, Receptos/Bristol Myers Squibb, Regeneron Pharmaceuticals, Inc., Shire. Dr. Chehade ‐ consultant: Adare, Allakos, Astra Zeneca, Nutricia, Regeneron Pharmaceuticals, Inc., Shire; research funding: Allakos, RegeneronPharmaceuticals Inc., Shire; honoraria for lectures: Medscape, Nutricia. Dr. Bredenoord – consultant: Arena, AstraZeneca, Calypso, EsoCap, Falk, Gossamer Bio, Medtronic, Laborie, RB, Regeneron, Robarts; research funding: Bayer, Nutricia, SST; equity interest: SST. Dr. Lucendo – Consultant: EsoCap, Dr. Falk Pharma; research funding: Dr. Falk Pharma, Regeneron Pharmaceuticals. Dr. Spergel – Consultant: Regeneron, Shire, Takeda, Allakos, DBV Technology, Novartis; Grant Support: Regeneron, DBV Technology. Q Zhao, JD Hamilton, B Beazley, S Kamat, M Ruddy, B Akinlade, N Amin, A Radin, B Shumel, J Maloney: Regeneron Pharmaceuticals, Inc. – employees and shareholders. I Guillemin: Sanofi – prior employee, may hold stock and/or stock options in the company L Mannent, E Laws: Sanofi – employees, may hold stock and/or stock options in the company.	
***Risk of bias***		
**Bias**	**Authors' judgement**	**Support for judgement**
Random sequence generation (selection bias)	Low risk	The author was contacted in November 2022 and confirmed that randomization was computer‐generated.
Allocation concealment (selection bias)	Low risk	The author was contacted in November 2022 and confirmed central allocation.
Blinding of participants and personnel (performance bias) All outcomes	Low risk	The author was contacted in November 2022 and confirmed blinding of participants and personnel.
Blinding of outcome assessment (detection bias) All outcomes	Low risk	The author was contacted in November 2022 and confirmed blinding of outcome assessors.
Incomplete outcome data (attrition bias) All outcomes	Low risk	There was no loss to follow‐up in either study arm.
Selective reporting (reporting bias)	Low risk	Protocol is available and all pre‐specified outcomes of interest were reported. Full data reported for all patients.
Other bias	Low risk	No major baseline differences between groups. No other concerns.

**Table:** Dellon 2022a

***Study characteristics***		
Methods	**RCT design and number of study arms:** RCT; 5 arms (4 intervention, 1 placebo) **Single‐center or multi‐center?** multi‐center (93 centers in 6 countries: US, Canada, Belgium, Switzerland, Spain, and Germany) **Countries:** several **Study dates:** May 2017 to August 2018	
Participants	**Active EoE or inactive EoE at beginning of study:** active **EoE definition/diagnostic criteria:** "defined as 3 episodes of dysphagia per week during the last 14 days of the 4‐ week baseline symptom assessment phase and a Global EoE Symptom Score of >3), and active esophageal eosinophilia (after evaluation of 5 biopsies from proximal and distal esophageal locations and at least 1 biopsy with a peak count of 15 eos/HPF) after documentation of failed histologic response on 8 weeks of high‐dose PPI." "High‐dose PPI was defined as 20 to 40 mg daily of any marketed PPI." **Inclusion criteria:** Adults (> or equal to 18 to < or equal to 75) with active EoE (with dysphagia at least 3 times weekly and global EoE symptom score > 3 as well as at least 15 eos/hpf) and failed histologic response on at least 8 weeks high‐dose PPI (20 mg to 40 mg daily) **Exclusion criteria:** Known esophageal mucosal disease or esophageal dysmotility unrelated to EoE History of esophageal stricture requiring dilation within 12 weeks prior or with severe stricture precluding passage of standard 8 mm to 10 mm scope was also an exclusion criterion Corticosteroids, change in diet, biologics, and immunomodulators were prohibited **Age at beginning of study per study group:** mean age overall: 39.3 ± 12.0 years. For 3 mg twice‐daily (n = 20): 36.8 ± 9.2; 3 mg every night at bedtime (n = 21): 42.9 ± 11.5; 1.5 mg twice‐daily (n = 22): 41.3 ± 12.2; 1.5 mg every night at bedtime (n = 21): 36.8 ± 11.5; placebo (n = 19): 38.6 ± 14.7 **Sex (m/f) per study group:** Sex: males 70/103 overall. For 3 mg twice‐daily: 16/20 male; 3 mg every night at bedtime: 11/21 male; 1.5 mg twice‐daily: 15/22 male; 1.5 mg every night at bedtime: 14/21 male; and placebo: 14/19 male. 106 patients randomized (details for each group shown above) **Number randomized per study group:** 3 mg twice‐daily – n = 20 3 mg every night at bedtime – n = 22 1.5 mg twice‐daily – n = 22 1.5 mg every night at bedtime – n = 21 Placebo twice‐daily – n = 21 **Number reaching end of study per study group:** 3 mg twice‐daily – n = 19 3 mg every night at bedtime – n = 20 1.5 mg twice‐daily – n = 20 1.5 mg every night at bedtime – n = 17 Placebo twice‐daily – n = 16	
Interventions	**Study group 1 (placebo):** placebo twice‐daily **Study groups 2, 3, 4, 5 (fluticasone):** 3 mg twice‐daily 3 mg every night at bedtime 1.5 mg twice‐daily 1.5 mg every night at bedtime	
Outcomes	**Primary outcomes of the study:** histologic response at 12 weeks (% participants with < or equal to 6 eos/HPF) **Secondary outcomes of the study:** Percentage of responders with sustained histologic response at weeks 26 and 52 Endoscopic severity measured by change from baseline in EREFS at weeks 12, 26, and 52 Percentage with < 1 and < 15 eos/HPF at weeks 12, 26, and 52 Secondary symptomatic outcomes: Change from baseline in the Global EoE Symptom Score multiple times in week 52 Change in frequency of reported dysphagia episodes over a 14‐day period from baseline through weeks 12, 26, and 52	
Notes	**Funding source**: Adare/Ellodi Pharmaceuticals, Inc. **Conflicts**: Dellon, Lucendo, Schoepfer, Falk, and Hirano research funding from Adare/Ellodi; Dellon, Schlag, Schoepfer, Falk, and Hirano consulting fees from Adare/Ellodi; Eagle, Nezamis, Comer, Knoop employees of Ellodi	
***Risk of bias***		
**Bias**	**Authors' judgement**	**Support for judgement**
Random sequence generation (selection bias)	Low risk	"Randomization with fixed block size of five using Interactive Web Response System"
Allocation concealment (selection bias)	Low risk	"Randomization with fixed block size of five using Interactive Web Response System"
Blinding of participants and personnel (performance bias) All outcomes	Low risk	Blinding is unclear in the manuscript. Study drug was identical to placebo. Confirmed by authors
Blinding of outcome assessment (detection bias) All outcomes	Low risk	No details provided except stating "blinded". The author was contacted in November 2022 and confirmed assessors were blinded.
Incomplete outcome data (attrition bias) All outcomes	Low risk	There was no loss to follow‐up for this study.
Selective reporting (reporting bias)	Low risk	Trial registry is available and all pre‐specified outcomes of interest were reported in the pre‐specified way.
Other bias	Low risk	No major baseline differences between groups. No other concerns.

**Table:** Dellon 2022b

***Study characteristics***		
Methods	**RCT design and number of study arms:** RCT, 3 arms **Single‐center or multi‐center:** multi‐center **Countries:** Australia, Netherlands, United States **Study dates:** 6 July 2020 to December 2021	
Participants	**Active EoE or inactive EoE at beginning of study:** active **EoE definition/diagnostic criteria:** esophagus: ≥ 15 eos/high‐power field (hpf) in 1 hpf and active moderate to severe symptoms – Dysphagia Symptom Questionnaire (DSQ) ≥ 12 **Inclusion criteria:** Dysphagia Symptom Questionnaire (DSQ) ≥ 1 Confirmed diagnosis of EoE and esophageal intraepithelial eosinophilic infiltration of ≥15 eosinophils/HPF in 1 hpf from a biopsy collected during the screening EGD without any other cause for the esophageal eosinophilia History (by patient report) of an average of ≥ 2 episodes of dysphagia with intake of solid foods per week during the 4 weeks prior to screening Participants must have failed or not be adequately controlled on standard of care treatments for EoE symptoms, which could include PPI, systemic or topical corticosteroids, and/or diet, among others If on an allowed treatment for EoE, stable dose for at least 4 weeks prior to screening and willingness to continue that dose for the study duration If patient is on pre‐existing dietary restrictions, willingness to maintain dietary restrictions throughout the study, as much as possible Able and willing to comply with all study procedures Female participants must be either post‐menopausal for at least 1 year with FSH level > 30 mIU/mL at screening or surgically sterile (tubal ligation, hysterectomy, or bilateral oophorectomy) for at least 3 months, or if of childbearing potential, have a negative pregnancy test and agree to use dual methods of contraception, or abstain from sexual activity from screening until the end of the study, or for 120 days following the last dose of study drug, whichever is longer. Male participants with female partners of childbearing potential must agree to use a highly effective method of contraception from screening until the end of the study or for 120 days following the last dose of study drug, whichever is longer. All fertile men with female partners of childbearing potential should be instructed to contact the Investigator immediately if they suspect their partner might be pregnant at any time during study participation. **Exclusion criteria:** Concomitant EG, EoD, or eosinophilic colitis (EC) EG and/or EoD (≥ 30 eosinophils/hpf in 5 hpf in the stomach and/or ≥ 30 eosinophils/hpf in 3 hpf in the duodenum) as determined by central histology assessment of biopsies collected during the screening EG Causes of esophageal eosinophilia other than EoE or one the following: hypereosinophilic syndrome, eosinophilic granulomatosis with polyangiitis, or peripheral blood absolute eosinophil count of > 1500 eosinophils/μL History of inflammatory bowel disease, celiac disease, achalasia, and/or esophageal surgery Any esophageal stricture unable to be passed with a standard diagnostic 9 mm to 10 mm upper endoscope or any critical esophageal stricture that requires dilation during screening History of bleeding disorders or esophageal varices History of malignancy; except carcinoma in situ, early stage prostate cancer, or non‐melanoma skin cancers. However, cancers that have been in remission for more than 5 years and are considered cured, can be enrolled (with the exception of breast cancer). All history of malignancy (including diagnosis, dates, and compliance with cancer screening recommendations) must be documented and certified by the Investigator, along with the statement that in their clinical judgment the tissue eosinophilia is attributable to EGID, rather than recurrence of malignancy. Active *Helicobacter pylori* infection (as determined by central histology staining of the biopsy collected during the screening EGD), unless treated and confirmed to be negative prior to randomization and symptoms remain consistent. Positive Ova and Parasite (O&P) test at screening, seropositive for *Strongyloides stercoralis* at screening, and/or treatment for a clinically significant helminthic parasitic infection within 6 months of screening Seropositive for HIV or hepatitis at screening, except for vaccinated patients or patients with a history of hepatitis that has since resolved. Prior exposure to AK002 or hypersensitivity to any constituent of AK002 Change in dose of inhaled corticosteroids, nasal corticosteroids, PPI, and/or diet therapy within 4 weeks prior to screening Use of oral corticosteroids (swallowed topical or systemic corticosteroids) within 8 weeks prior to screening Use of any biologics or medications that may interfere with the study, such as immunosuppressive or immunomodulatory drugs including azathioprine, JAK inhibitors, 6‐mercaptopurine, methotrexate, cyclosporine, tacrolimus, anti‐TNF, anti‐IL‐4 receptor, e.g. dupilumab), anti‐IL‐5 (e.g. mepolizumab), anti‐IL‐5 receptor (e.g. benralizumab), anti‐IL‐13 (e.g. lebrikizumab), anti‐IgE (e.g. omalizumab), within 12 weeks prior to screening. Participation in a concurrent interventional study with the last intervention occurring within 30 days prior to administration of study drug or 90 days or 5 half‐lives, whichever is longer, for biologic products Vaccination with live attenuated vaccines ≤ 30 days prior to initiation of treatment in the study, during the treatment period, or vaccination expected ≤ 5 half‐lives (≤ 4 months) following study drug administration Treatment with chemotherapy or radiotherapy in the preceding 6 months The presence of abnormal laboratory values is considered by the investigator to be clinically significant Any disease, condition (medical or surgical), or cardiac abnormality, in the opinion of the Investigator, would place the participant at increased risk Known history of alcohol, drug, or other substance abuse or dependence Women who are pregnant, breastfeeding or planning to become pregnant while participating in the study Any other reason that in the opinion of the Investigator or Medical Monitor makes the patient unsuitable for enrollment **Age at beginning of study per study group** Group 1 high‐dose lirentelimab (n = 91) 29 (12 to 69) Group 2 low‐dose lirentelimab (n = 93) 34 (12 to 67) Group 3 placebo (n = 92) 32 (12 to 70) **Sex (m/f) per study group** Group 1 high‐dose lirentelimab (n = 91) 29% (26) Group 2 low‐dose lirentelimab (n = 93) 43% (40) Group 3 placebo (n = 92) 40% (37) **Number randomized per study group** Group 1 high‐dose lirentelimab (n = 91) Group 2 low‐dose lirentelimab (n = 93) Group 3 placebo (n = 92) **Number reaching end of study per study group** NR	
Interventions	**Study group 1:** high‐dose lirentelimab 3 mg/kg **Study group 2:** low‐dose lirentelimab 1 mg/kg **Group 3:** placebo	
Outcomes	**Primary outcomes of the study:** **Histologic co‐primary endpoint –** proportion of tissue eosinophil responders: esophagus: ≤ 6 eos/hpf in peak hpf **Symptom co‐primary endpoint –** absolute change in Dysphagia Symptom Questionnaire (DSQ) score **Secondary outcomes of the study:** **Secondary endpoints –** percent change in DSQ from baseline Achieved peak esophageal eos ≤ 1 eos/hpf at week 24	
Notes	**Funding source:** Allakos **Conflicts of interest:** Evan S. Dellon, MD, MPH	
***Risk of bias***		
**Bias**	**Authors' judgement**	**Support for judgement**
Random sequence generation (selection bias)	Low risk	This is not stated, and only a protocol and abstract presentation are available for review. Authors contacted for further details and confirmed in November 2022 that the schedule was computer‐generated and centrally allocated.
Allocation concealment (selection bias)	Low risk	This is not stated, and only a protocol and abstract presentation are available for review. Authors contacted for further details and confirmed in November 2022 that the schedule was computer‐generated and centrally allocated.
Blinding of participants and personnel (performance bias) All outcomes	Low risk	The manuscript states "Quadruple (Participant, Care Provider, Investigator, Outcomes Assessor)" but doesn't state how this was achieved. Authors contacted for further details and confirmed in November 2022 that personnel and participants were blinded.
Blinding of outcome assessment (detection bias) All outcomes	Low risk	"Quadruple masking Outcomes Assessor"; does not state how this was achieved. Authors contacted for further details and confirmed in November 2022 that personnel and participants were blinded.
Incomplete outcome data (attrition bias) All outcomes	Low risk	277 recruited and 276 received medication ‐ outcome for all is recorded with no attrition.
Selective reporting (reporting bias)	Low risk	Trial registration reviewed. Primary outcomes appropriate and match the published abstract with none missing. NCT04322708
Other bias	Low risk	No balance bias from the baseline table. No other concerns.

**Table:** Dohil 2010

***Study characteristics***		
Methods	**RCT design and number of study arms:** RCT; 2 arms **Single‐center or multi‐center:** single‐center; Eosinophilic Esophagitis Clinic at Rady Children’s Hospital, San Diego **Countries:** USA **Study dates:** patients were recruited between February 2008 and July 2009 and the study treatment lasted 3 months	
Participants	**Active EoE or inactive EoE at beginning of the study:** active **EoE definition/diagnostic criteria:** peak eos ≥ 20/hpf **Inclusion criteria:** Histologic evidence of eosinophilic esophagitis defined as greater than 20 eosinophils per HPF on esophageal biopsy Ages 1 years and older Ability to continue the same diet that the patient was on at the time of EGD with biopsy **Exclusion criteria:** Adverse reaction or allergy to budesonide Pregnancy Chronic diseases requiring immunomodulatory therapy Use of swallowed topical corticosteroids for eosinophilic esophagitis within the past 3 months Use of systemic steroids 2 months prior to study entry Upper gastrointestinal bleed within 4 months of study entry Chronic use of medications that predispose to upper gastrointestinal bleeding including non‐steroidal anti‐inflammatory medications or anticoagulants Evidence of adrenal suppression prior to study entry Evidence of concurrent eosinophilic gastritis, enteritis, colitis, or proctitis Recent changes in asthma or allergic rhinitis therapy for 3 months **Age at beginning of study per study group:** NR **Sex (m/f) per study group:** NR **Number randomized per study group:** Budesonide, oral viscous: n = 21 Placebo: n = 11 **Number reaching the end of study per study group:** Budesonide, oral viscous: n = 15 Placebo n = 9	
Interventions	**Study group 1:** sterile water **Study group 2:** budesonide oral viscous suspension (0.5 mg/2 mL)	
Outcomes	**Primary outcomes of the study:** primary outcome measure was an improvement of esophageal eosinophilia. This was determined by comparing peak eos counts/hpf at baseline and after treatment. Patients were categorized into responders (0 to 6 eos/hpf), partial responders (7 to 19 eos/hpf), and non‐responders (> 20 eos/hpf). **Secondary outcomes of the study:** secondary outcome measurements included the response of symptoms and endoscopic and histologic features to treatment	
Notes	**Conflicts of interest:** The authors disclose the following: The University of California, San Diego has a financial interest in Meritage Pharma, the company sponsoring this research. Drs Dohil, Bastian, and Aceves and the University of California may financially benefit from this interest if the company is successful in developing and marketing its own product that is related to this research. The terms of this arrangement have been reviewed and approved by the University of California, San Diego in accordance with its conflict of interest policies. The remaining authors disclose no conflicts. **Funding:** support was provided by a grant from Meritage Pharma, San Diego, CA	
***Risk of bias***		
**Bias**	**Authors' judgement**	**Support for judgement**
Random sequence generation (selection bias)	Low risk	Computer‐generated randomization method.
Allocation concealment (selection bias)	Low risk	Suspensions of the medications were placed by the investigational pharmacist into sealed light‐protective vials. Only the pharmacist had access to the randomization code.
Blinding of participants and personnel (performance bias) All outcomes	Unclear risk	The manuscript stated blinded personnel and patients. Patients were blinded due to sealed envelope but no details given on personnel blinding. Only the pharmacist had access to the randomization code.
Blinding of outcome assessment (detection bias) All outcomes	Low risk	"During the study, collected data were stored in a locked cabinet. One month after study completion, the database was locked and the randomisation code revealed".
Incomplete outcome data (attrition bias) All outcomes	Low risk	"Thirty‐one patients were randomized; 24 completed the study and were included in the final analysis. Of these 24 participants, 15 received OVB and PPI and 9 received placebo and PPI." The authors explain exclusions from analysis. Eight patients (2 placebo and 6 intervention) did not have a follow‐up endoscopy.
Selective reporting (reporting bias)	Low risk	Protocol is available and all pre‐specified outcomes of interest were reported in the pre‐specified way.
Other bias	Low risk	There were no major baseline differences between the groups. 1 patient included in the placebo group with 15 eosinophils per high‐power field, which was below the cutoff for inclusion. All patients were continued on PPI. Also diet differences from patient to patient as some patients were avoiding food groups.

**Table:** Gupta 2015

***Study characteristics***		
Methods	**RCT design and number of study arms:** RCT with 4 arms, age stratified (2 to 9 and 10 to 18 years old), parallel assignment, dose‐ranging **Single‐center or multi‐center:** multi‐center **Countries:** United States **Study dates:** January 2009 to April 2010	
Participants	**Active EoE or inactive EoE at beginning of study:** active **EoE definition/diagnostic criteria:** esophageal biopsy must show ≥ 20 eos per HPF (400x, 0.3 mm^2^ HPF) at 2 or more levels of the esophagus following 4 weeks of high dose PPI (type, actual dosage not specified) **Inclusion criteria:** Male and female participants between the ages of 2 to 18 years, inclusive History of clinical symptoms of esophageal dysfunction intermittently or continuously Histologic evidence of EoE with a peak eosinophil count of greater than or equal to 20 eosinophils per HPF, from 2 or more levels of the esophagus, within 6 weeks prior to the Baseline Visit At the Baseline Visit, participants must have symptoms with a total EoE Clinical Symptom Score of greater than or equal to 3 Willingness and ability to continue the dietary therapy, environmental therapy, and/or medical regimens (including gastric acid suppression, if any) in effect at the Screening Visit Females of childbearing potential must have a negative serum pregnancy test (beta human chorionic gonadotropin) prior to randomization into the study and sexually active participants must agree to continue acceptable birth control measures throughout the duration of the study Written informed consent (parent or legal guardian) and, as appropriate, participant assent **Exclusion criteria:** Current use of immunomodulatory therapy (or anticipated use within 12 weeks following the Baseline Visit) Diagnosis of inflammatory bowel disease Chronic viral infection or immunodeficiency condition (current) Use of swallowed topical corticosteroids for EoE in the 1 month prior to the biopsy required for entrance to this study or at any time between the biopsy and the Baseline Visit Use of systemic (oral or parenteral) corticosteroid within 1 month prior to the biopsy required for entrance to this study or at any time between the biopsy and the Baseline Visit Morning plasma cortisol level below the lower limit of normal (per central laboratory reference range) at the Screening Visit Upper gastrointestinal bleeding within 1 month prior to the Screening Visit or between the Screening Visit and Baseline Visit Current use of anticoagulants Current disease of the gastrointestinal tract aside from the current EoE diagnosis Evidence of concurrent eosinophilic gastritis, enteritis, colitis, or proctitis Evidence of active infection with *Helicobacter pylori* Evidence of unstable asthma or changes in asthma or allergic rhinitis therapy within 1 month prior to the biopsy required for entrance to this study Any female who is pregnant, who is planning to become pregnant, or who is breastfeeding Current evidence or history of hypersensitivity or idiosyncratic reaction to budesonide or any other ingredients of the study medication Current evidence of oropharyngeal or esophageal candidiasis Receipt of an investigational drug within 30 days prior to the biopsy required for entrance to this study Any condition or abnormality that, in the opinion of the Principal Investigator, would compromise the safety of the participant or successful conduct of the study **Age at beginning of study per study group**: placebo, 9.2 (4.36); low‐dose oral budesonide suspension (OBS), 9 (5.88); medium‐dose OBS, 10.2 (4.89); high‐dose OBS, 8.1 (4.58) **Sex (m/f) per study group:** placebo, 16/5; low‐dose OBS, 17/4; medium‐dose OBS, 17/4; high‐dose OBS, 16/5 **Number randomized per study group (numbers of patients):** placebo, 21; low‐dose OBS, 21; medium‐dose OBS, 19; high‐dose OBS, 20 **Number reaching end of study per study group (numbers of patients):** placebo, 18; low‐dose OBS, 17; medium‐dose OBS, 19; high‐dose OBS, 17	
Interventions	**(Group 1) placebo**: participants received placebo twice‐daily at bedtime (hs) and after breakfast (once a day in the morning, pc) for 12 weeks with a 3 week taper period **(Group 2) low‐dose OBS:** participants received oral budesonide suspension (OBS) 0.05 mg/mL at bedtime (hs) and placebo after breakfast (once a day in the morning, after meals) for 12 weeks, with a total daily dose of 0.35 mg (2 to 9 years) or 0.50 mg (10 to 18 years), followed by a 3‐week taper period **(Group 3) medium‐dose OBS:** participants received oral budesonide suspension (OBS) 0.2 mg/mL at bedtime (hs) and placebo after breakfast (once a day in the morning, after meals) for 12 weeks, with a total daily dose of 1.4 mg (2 to 9 years) or 2.0 mg (10 to 18 years), followed by a 3‐week taper period **(Group 4) high‐dose OBS:** participants received oral budesonide suspension (OBS) 0.2 mg/mL at bedtime (hs) and after breakfast (once a day in the morning, after meals) for 12 weeks, with a total daily dose of 2.8 mg (2 to 9 years) or 4.0 mg (10 to 18 years), followed by a 3‐week taper period	
Outcomes	**Primary outcome:** percent of participants who responded to therapy following 12 weeks of treatment The response was defined as a ≥ 50% reduction from baseline in the eosinophilic esophagitis (EoE) clinical symptom score (CSS) and a reduction in peak eosinophil count to ≤ 6/high‐power field (light microscopy) from esophageal biopsies collected at the final evaluation **Secondary outcome:** Percent of participants with histologic response following 12 weeks of treatment Histologic response was defined as a maximum peak eosinophil count at the final treatment evaluation of ≤ 6 eosinophils/high‐power field (light microscopy). The maximum peak was identified by examining the peak eosinophil counts obtained from the proximal, mid, and distal esophageal biopsies and selecting the maximum value. Percent of participants with histologic remission following 12 weeks of treatment Histologic remission was defined as a maximum peak eosinophil count at the final treatment evaluation of ≤ 1 eosinophils/high‐power field (light microscopy). The maximum peak was identified by examining the peak eosinophil counts obtained from the proximal, mid, and distal esophageal biopsies and selecting the maximum value. Percent change from baseline in peak eosinophil count following 12 weeks of treatment The maximum peak number of eosinophils at baseline and at the final treatment evaluation was identified by examining the peak eosinophil counts obtained from the proximal, mid, and distal esophageal biopsies and selecting the maximum value. A negative change from baseline indicates that eosinophil count has decreased. Change from baseline in endoscopy score following 12 weeks of treatment Percent of participants with clinical response following 12 weeks of treatment Response was defined as a ≥ 50% reduction from baseline in the eosinophilic esophagitis (EoE) clinical symptom score (CSS) Percent of participants with clinical remission following 12 weeks of treatment Clinical remission was defined as an eosinophilic esophagitis (EoE) clinical symptom score (CSS) of zero Percent change from baseline in eosinophilic esophagitis (EoE) clinical symptom score (CSS) following 12 weeks of treatment Change from baseline in physician's global assessment score of disease severity following 12 weeks of treatment Maximum plasma concentration (C_max_) of budesonide (time frame: week 2, 4, or 8, or at the final treatment evaluation) Time to maximum (Tmax) and half maximum (T_1/2_) plasma concentration of budesonide (time frame: week 2, 4, or 8, or at the final treatment evaluation) Area under the plasma concentration‐time curve (AUC) of budesonide from time zero to time of the last measurable concentration (AUC_0-last_) (time frame: week 2, 4, or 8, or at the final treatment evaluation) Percent of participants with potential corticosteroid‐related treatment‐emergent adverse events (TEAEs) (time frame: 15 weeks after the start of treatment) Mean change in blood pressure (BP) at end of treatment (time frame: baseline, 12 weeks after the start of treatment)	
Notes	**Sponsor:** Takeda (Shire)/Meritage Pharma, Inc **Conflicts of interest:** The authors disclose the following: Sandeep K. Gupta was a principal investigator for this study, is a consultant to Meritage Pharma, Inc, Abbott, and Receptos, Inc, and is a member of the Medical Advisory Panel of the AmericanPartnership for Eosinophilic Disorders and the executive committee of the International Gastrointestinal Eosinophil Researchers. Joanne M. Vitanza was employed by Meritage Pharma, Inc, as the medical monitor during the design and conduct of this study, is a consultant to Meritage Pharma, Inc, and owns shares of Meritage Pharma, Inc, stock. Margaret H. Collins performed the central pathology slide review for this study, is a consultant to Meritage Pharma, Inc, Novartis, Receptos, Inc, and Aptalis, and is a member of the Medical Advisory Panel of the American Partnership for Eosinophilic Disorders and the executive committee of the International Gastrointestinal Eosinophil Researchers.	
***Risk of bias***		
**Bias**	**Authors' judgement**	**Support for judgement**
Random sequence generation (selection bias)	Low risk	The study sponsor confirmed that the randomization schedule was generated using a computer program.
Allocation concealment (selection bias)	Low risk	The study sponsor has confirmed that "Labeling of study treatment bottles was performed by an independent clinical services provider according to a blinded randomization scheme; active study drug and matching placebo were dispensed in identical bottles according to a blinded randomization scheme; and study participants, investigators, and the sponsor remained blinded to the randomization scheme until the blind was formally broken after all participants completed the study and the database was locked".
Blinding of participants and personnel (performance bias) All outcomes	Low risk	Stated as "blinded participant, care provider, and investigator," and placebo drug looked similar to the intervention.
Blinding of outcome assessment (detection bias) All outcomes	Low risk	The study sponsor confirmed that the outcome assessors were blinded.
Incomplete outcome data (attrition bias) All outcomes	Low risk	Balanced and explained attrition that likely has no effect on outcome.
Selective reporting (reporting bias)	Low risk	Protocol is available and all pre‐specified outcomes of interest were reported in the pre‐specified way.
Other bias	Low risk	No major baseline differences between groups. Noted differences in PPI and H2 use, but this appears to be random associated with the selection process.

**Table:** Heine 2019

***Study characteristics***		
Methods	**RCT design and number of study arms:** RCT, 2 arms **Single‐center or multi‐center:** multi‐center ‐ Australia; The Royal Childrens Hospital ‐ Parkville; Women's and Childrens Hospital ‐ North Adelaide; Monash Medical Centre ‐ Clayton campus ‐ Clayton; The Children's Hospital at Westmead ‐ Westmead **Countries:** USA, Australia **Study dates:** August 2012 to July 2015	
Participants	**Active EoE or inactive EoE at beginning of study:** active at randomization **EoE definition/diagnostic criteria:** EoE (≥ 15 eosinophils per high‐power field; HPF) **Inclusion criteria:** Stage 1: All patients with possible EoE (age 1 to 18 years) undergoing a diagnostic gastroscopy at any of the 4 participating hospitals (at Royal Children’s Hospital Melbourne, Monash Medical Centre Melbourne, Children’s Hospital at Westmead Sydney, and Women’s and Children’s Hospital Adelaide) will be assessed for inclusion in the study. Patients undergoing a diagnostic gastroscopy will be assessed for upper gastrointestinal symptoms, including: nausea and vomiting, abdominal pain, diarrhea, weight loss, dysphagia, regurgitation, food bolus obstruction, food refusal Stage 2: Patients will be included in the randomized trial if a diagnosis of EoE was confirmed based on biopsies from the first gastroscopy. Biopsies will be analyzed by the hospital’s anatomical pathologist to assess whether the diagnostic criteria for EoE is confirmed. The diagnosis of EoE is based on the presence of at least 20 mucosal eosinophils/HPF (abstract says ≥ 15) in any of the upper or lower esophageal biopsies. NB: Patients who are already avoiding a food prior to the study as part of a known food allergy (e.g. egg or peanut allergy) can still be considered for the study if EoE was diagnosed while avoiding the food (which suggests that the food is not causing the EoE). **Exclusion criteria:** The patient does not fulfil the histological criteria for EoE (fewer than 20 mucosal eosinophils/HPF on histology). Recent treatment with systemic corticosteroids (prednisolone) or topical or inhaled corticosteroids (fluticasone aerosol or viscous budesonide) within 3 months of the gastroscopy. Recent or current treatment with omeprazole Patient already on multiple food elimination diet Previous failure of elimination diets (as it may skew the data towards non‐response) Inability to obtain informed consent Inability to comply with the prescribed 4‐food elimination diet or PPI treatment Contraindications to gastroscopy or general anesthesia **Age at beginning of study per study group:** mean age total study 9.1 years **Sex (m/f) per study group (numbers of patients):** NR **Number randomized per study group:** Proton pump inhibitor (PPI) plus 4‐food elimination diet: 32 Proton pump inhibitor (PPI): 32 **Number reaching end of study per study group (numbers of patients):** Proton pump inhibitor (PPI) plus 4‐food elimination diet: 27/32 Proton pump inhibitor (PPI): 30/32	
Interventions	**Study group 1:** active treatment, oral proton pump inhibitor (PPI) plus 4‐food elimination diet (strictly avoiding all foods containing cow’s milk, soy, wheat or egg) + omeprazole: 7.5 kg to 9.9 kg: 5 mg morning and 10 mg night, 10.0 kg to 14.9 kg: 10 mg twice‐daily, 15.0 kg to 19.9 kg: 15 mg twice‐daily, > 20 kg: 20 mg twice‐daily **Study group 2:** omeprazole: 7.5 kg to 9.9 kg: 5 mg morning and 10 mg night, 10.0 kg to 14.9 kg: 10 mg twice‐daily, 15.0 kg to 19.9 kg: 15 mg twice‐daily, > 20 kg: 20 mg twice‐daily	
Outcomes	**Primary outcomes of the study:** histological as number of eosinophils per high‐power microscopic field **Secondary outcomes of the study:** Clinical response (symptom score) Endoscopic appearance (endoscopy score)	
Notes	**Funding source:** National Health and Medical Research Council (NHMRC Project Grant #1029972) ‐ Australia **Conflicts of interest:** NR	
***Risk of bias***		
**Bias**	**Authors' judgement**	**Support for judgement**
Random sequence generation (selection bias)	Low risk	Computer‐generated randomization method.
Allocation concealment (selection bias)	Low risk	Treatment was centrally allocated.
Blinding of participants and personnel (performance bias) All outcomes	High risk	This was an open‐label trial.
Blinding of outcome assessment (detection bias) All outcomes	High risk	This was an open‐label trial. The authors were contacted in November 2022 to determine if assessors were blinded. No response.
Incomplete outcome data (attrition bias) All outcomes	Unclear risk	Attrition numbers were given per group but no reasons provided. Authors contacted in November of 2022. No response.
Selective reporting (reporting bias)	High risk	The authors did not provided their outcome of complete remission of < 5 eosinophils per high‐power field. Authors contacted in November of 2022. No response.
Other bias	Low risk	No major baseline differences between groups. No other concerns.

**Table:** Hirano 2019

***Study characteristics***		
Methods	**RCT design and number of study arms:** multi‐center, double‐blind trial, 3 arms **Single‐center or multi‐center:** multi‐center (30) **Countries:** US, Canada, Switzerland **Study dates:** September 2014 through December 2015	
Participants	**Active EoE or inactive EoE at beginning of study:** active **EoE definition/diagnostic criteria:** symptoms of dysphagia for a minimum of 4 days over 2 weeks (within the 4‐week screening period) and histologic evidence of EoE, defined as a peak count of ≥ 15 eosinophils per high‐power field (eos/hpf; microscope hpf = 0.3 mm^2^) at any 2 of 3 levels of the esophagus (proximal, mid, distal) when off anti‐inflammatory therapy for EoE. **Inclusion criteria:** Patients must have previously received an adequate trial of a proton pump inhibitor to exclude gastroesophageal reflux disease and proton pump inhibitor‒responsive esophageal eosinophilia as the primary cause of their symptoms. Prior treatment of patients with steroids for EoE was recorded, with steroid refractory defined as an adequate trial of systemic or swallowed topical steroids failing to result in a meaningful reduction in symptoms, as judged by the investigator. Participants with a partial response to a proton pump inhibitor (PPI) who met all other eligibility criteria could be enrolled; prospective participants who discontinued use of a PPI had to wait at least 4 weeks before their screening endoscopy; if a prospective participant was receiving a PPI at screening, the participant must have been receiving a stable dose for at least 4 weeks before the screening endoscopy and agreed to continue on the same dose through week 16; men and women of childbearing potential had to agree to use adequate birth control measures during the trial and for 5 months after their last dose of study drug; all women of childbearing potential must have had a negative serum pregnancy test at screening and a negative urine (or serum) pregnancy test before dosing on day 1. **Exclusion criteria:** Exclusion criteria included clinical or endoscopic evidence of the presence of any other disease that may have interfered with or affected the histologic, endoscopic, and clinical symptom endpoints for this trial (e.g. erosive esophagitis grade 2 or above, Barrett’s disease, upper gastrointestinal bleed, eosinophilic gastritis or gastroenteritis, duodenal or gastric eosinophilia on screening endoscopy, inflammatory bowel disease, significant hiatal hernia (> 3 cm)); presence of esophageal varices; evidence of severe endoscopic structural abnormality in esophagus (e.g. high‐grade stenosis where an 8 mm to 10 mm endoscope could not pass through the stricture without dilation at the time of endoscopy); primary causes of esophageal eosinophilia other than EoE; evidence of immunosuppression or were receiving systemic immunosuppressive or immunomodulating drugs (e.g. methotrexate, cyclosporine, interferon alpha, tumor necrosis factor alpha inhibitors, antibodies to immunoglobulin E) within 5 drug half‐lives before screening; were receiving systemic or swallowed topical corticosteroid medication; prospective participants with EoE treated with a corticosteroid must have not received a systemic corticosteroid within 8 weeks or swallowed topical corticosteroids within 4 weeks of the screening endoscopy or the start of the daily clinical symptom diary data collection during screening, whichever was performed first; presence of any other disease making conduct of the protocol or interpretation of the trial results difficult or that would have put the prospective participant at risk by participating in the trial (e.g. infection causing eosinophilia, gastritis, colitis, irritable bowel syndrome, and celiac disease, which have similar symptoms, neurologic or psychiatric illness that compromised the prospective participant’s ability to accurately document symptoms of EoE); liver function impairment or persisting elevations of aspartate aminotransferase or alanine aminotransferase > 2 times the upper limit of normal (ULN), or direct bilirubin > 1.5 times the ULN; systemic or diarrheal illness following travel or residence in endemic areas of parasitic/helminthic infections, history of clinical schistosomiasis, history of travel to endemic areas within preceding 6 months; ongoing infection (e.g. hepatitis B or C, human immunodeficiency virus, active tuberculosis); pregnancy or lactation; concurrent treatment with another investigational drug; prospective participants could not have participated in a concurrent investigational drug trial or have received an investigational drug within 5 drug half‐lives before signing the informed consent form for this trial; weight less than 40 kg (88.2 pounds) or greater than 125 kg (275 pounds); history of February 2019 RPC4046 EoE Phase 2 Trial 603.e2 idiopathic anaphylaxis or a known history of a major immunologic reaction (such as anaphylactic reaction, anaphylactoid reaction, or serum sickness) to an immunoglobulin G–containing agent; history of cancer or lymphoproliferative disease, other than a successfully treated nonmetastatic cutaneous squamous cell or basal cell carcinoma or adequately treated cervical carcinoma in situ, within 10 years of screening; esophageal dilation for symptom relief during the screening period and within 4 weeks before baseline assessment of dysphagia or anticipated to be performed during the trial. **Age at beginning of study per study group:** Mean ± SD: **placebo** 38.6 ± 11.03; **RPC4046 (cendakimab) 180 mg** 39.1 ± 9.87; **RPC4046 (cendakimab) 360 mg** 33.9 ± 10.92 Median (range): **placebo** 38.5 (19, 64); **RPC4046 (cendakimab) 180 mg** 40.0 (19, 59); **RPC4046 (cendakimab) 360 mg** 31.5 (18, 63) **Sex (m/f) per study group:** Sex, n (%): male **placebo** 22 (64.7); **RPC4046 (cendakimab) 180 mg** 19 (61.3); **RPC4046 (cendakimab) 360 mg** 20 (58.8); female: **placebo** 12 (35.3); **RPC4046 (cendakimab) 180 mg** 12 (38.7); **RPC4046 (cendakimab) 360 mg** 14 (41.2) **Number randomized per study group (numbers of patients):** Placebo: 34 RPC4046 (cendakimab) 180 mg: 32 RPC4046 (cendakimab) 360 mg: 34 **Number reaching end of study per study group (numbers of patients):** Placebo: 32 RPC4046 (cendakimab) 180 mg: 28 RPC4046 (cendakimab) 360 mg: 30	
Interventions	**Study group 1 (placebo):** placebo **Study group 2:** 180 mg RPC4046 (cendakimab) SC once‐weekly (with initial loading dose of 5 mg/kg IV) **Study group 3:** 360 mg RPC4046 (cendakimab) SC once‐weekly (with initial loading dose of 10 mg/kg IV)	
Outcomes	**Primary outcomes of the study:** change in mean esophageal eosinophil count in the 5 HPF with the highest level of inflammation **Secondary outcomes of the study:** mean change in the dysphagia clinical symptom frequency and severity from baseline to week 16 as assessed by DSD completed over 2 weeks before the week 16 endpoint. Other secondary outcomes included change in EEsAI PRO score, peak esophageal eosinophil count, EREFS, patient’s and clinician’s global assessments of disease severity, patient’s global impression of change in EoE symptoms, and esophageal histologic severity (grade) and extent (stage).	
Notes	**Funding source:** This study was sponsored by Celgene Corporation **Conflicts of interest:** These authors disclose the following: Ikuo Hirano has served as a consultant for Adare, Allakos, Celgene Corporation, Regeneron, and Shire, and has received grant/research support from Adare, Celgene Corporation, Regeneron, and Shire. Margaret H. Collins has served as a consultant for Celgene Corporation, Regeneron, and Shire and has received grant/research support from Celgene Corporation, Regeneron, and Shire. Sandeep Gupta has received grant/research support from Shire and served as a consultant for Abbott, Adare, Allakos, Celgene Corporation, and QOL. Alain M. Schoepfer has received grant/research support from Adare, Celgene Corporation, Falk, Merck Sharp & Dohme, and Regeneron, and has served as a consultant and advisor for AbbVie, Adare, Celgene Corporation, Falk, Merck Sharp & Dohme, and Regeneron. Alex Straumann has served as a consultant for Actelion, Calypso, Celgene Corporation, Falk, GlaxoSmithKline, Merck, Merck Sharp & Dohme, Novartis, Nutricia, Pfizer, Regeneron‐Sanofi, Roche‐Genentech, and Tillotts, and has received grant/research support from Celgene Corporation. Ekaterina Safroneeva has served as a consultant for Aptalis Pharma, Celgene Corporation, Novartis, and Regeneron. Michael Grimm, Heather Smith, Cindy‐ann Tompkins, Amy Woo, Robert Peach, Paul Frohna, Sheila Gujrathi, Darryl N. Penenberg, Caiyan Li, and Richard Aranda were employees of Receptos at the time of the study; Receptos is now a wholly owned subsidiary of Celgene Corporation. Gregory J. Opiteck and Allan Olson are employees of Celgene Corporation. Marc E. Rothenberg has served as a consultant for Adare, Allakos, AstraZeneca, Celgene Corporation, GlaxoSmithKline, NKT Therapeutics, Novartis, Pulm One, Shire, and Spoon Guru; has an equity interest in Immune Pharmaceuticals, NKT Therapeutics, Pulm One, and Spoon Guru; has received royalties from Teva for reslizumab; and is an inventor of patents owned by Cincinnati Children’s Hospital Medical Center. Evan S. Dellon has served as a consultant for Adare, Alivio, Allakos, Banner, Celgene Corporation, Enumeral, GSK, Regeneron, Robarts, and Shire, has received grant/research support from Adare, Banner, Celgene Corporation, Meritage, Miraca, Nutricia, Regeneron, and Shire, and has received educational grants from Banner and Holoclara. The remaining authors disclose no conflicts.	
***Risk of bias***		
**Bias**	**Authors' judgement**	**Support for judgement**
Random sequence generation (selection bias)	Low risk	Computer‐generated randomization method.
Allocation concealment (selection bias)	Low risk	Treatment was allocated centrally. "The study drug, RPC4046, and placebo solutions were identical in physical appearance."
Blinding of participants and personnel (performance bias) All outcomes	Low risk	"The treatment each patient received was not disclosed to the investigator, trial center personnel, patient, sponsor, or their representatives. Each patient’s treatment group assignment blind was not broken until all patients completed the doubleblind treatment period".
Blinding of outcome assessment (detection bias) All outcomes	Low risk	A central pathologist blinded to treatment allocation determined histologic changes.
Incomplete outcome data (attrition bias) All outcomes	Low risk	Balanced and explained attrition that likely has no effect on outcome.
Selective reporting (reporting bias)	Low risk	Trial protocol is available and all pre‐specified outcomes of interest were reported in the pre‐specified way.
Other bias	Low risk	Most baseline features balanced across both groups. IgE and eosinophils higher in intervention groups than in placebo group.

**Table:** Hirano 2020

***Study characteristics***		
Methods	**RCT design and number of study arms:** RCT (2 arms) **Single‐center or multi‐center:** multi‐center **Countries:** USA **Study dates:** May 2015 to July 2017	
Participants	**Active EoE or inactive EoE at beginning of study:** active **EoE definition/diagnostic criteria:** Active esophageal inflammation was to be evident at screening (i.e. peak cell count 15 eosinophils per high‐power field (eos/HPF): 400 magnification of a 0.3 mm2 field) as indicated by esophageal pinch biopsy specimens from at least 2 of 3 esophageal sites from endoscopy performed no more than 2 weeks after at least 8 weeks of treatment with high‐dose (or twice‐daily dosed) PPIs **Inclusion criteria:** Male or female, 18 to 65 years old, with a documented diagnosis of EoE by endoscopy before or at screening. Note: Must include a demonstration of intraepithelial eosinophilic infiltration (peak cell count 15 eos/HPF (400, 0.3 mm2)) from esophageal biopsy specimens from endoscopy performed no more than 2 weeks after at least 8 weeks of treatment with high‐dose (or twice‐daily dosing) PPIs. History (by patient report) of, on average, at least 2 episodes of dysphagia (with intake of solids off antiinflammatory therapy) per week in the 4 weeks before screening and, on average, at least 2 episodes of documented dysphagia per week in the weeks between screening and baseline. Dysphagia is defined as trouble swallowing solid food, or having solid food stick, by patient report. Must remain on a stabilized diet for at least 6 weeks before screening and during the course of the study; stable diet is defined as no initiation of single or multiple elimination diets or reintroduction of previously eliminated food groups. SDI PRO score 5 at screening and baseline. Documented history of or presence of 1 or more of any of the following: allergic disease (e.g. allergic asthma, allergic rhinitis, atomic dermatitis, or food allergies); blood eosinophil count 0.25 GI/L; serum total IgE 100 kU/L. Willing and able to comply with all clinic visits and study‐related procedures. Able to understand and complete study‐related questionnaires. Provide signed informed consent. Endoscopy with photographs performed at screening, with a demonstration of intraepithelial eosinophilic infiltration (peak cell count 15 eos/HPF) in at least 2 of the 3 biopsied esophageal regions (proximal, mid, or distal). **Exclusion criteria:** Prior participation in a dupilumab (anti‐IL4R) clinical trial. Other causes of esophageal eosinophilia or the following diseases: hypereosinophilic syndromes, Churg‐Strauss vasculitis, or eosinophilic gastroenteritis. History of achalasia, active *Helicobacter pylori* infection, Crohn’s disease, ulcerative colitis, celiac disease, or prior esophageal surgery before screening. Any esophageal stricture unable to be passed with a standard, diagnostic, adult (9 mm to 10 mm) upper endoscope, or any critical esophageal stricture that required dilation at screening. History of bleeding disorders or esophageal varices. Use of chronic aspirin, nonsteroidal agents, or anticoagulants within 2 weeks before screening; patients should not stop these agents solely to become eligible for entry into this study. Treatment with an investigational drug within 2 months or within 5 half‐lives (if known), whichever is longer, before screening. Use of systemic glucocorticoids within 3 months or swallowed topical glucocorticoids within 6 weeks before screening. Use of inhaled or nasal glucocorticoids within 3 months before screening and during the study, except stable dose for at least 3 months before screening biopsy (which cannot be changed during the study). Treatment with oral immunotherapy within 6 months before screening. Allergen immunotherapy (sublingual immunotherapy and/or subcutaneous immunotherapy), unless receiving stable dose for at least 1 year before screening. The following treatments within 3 months before the screening visit, or any condition that, in the opinion of the investigator, is likely to require such treatment(s) during the 3 months of study treatment: systemic immunosuppressive/immunomodulating drugs (e.g. omalizumab, cyclosporine, mycophenolate mofetil, interferon gamma, Janus kinase inhibitors, azathioprine, methotrexate, and leukotriene inhibitors (except stable dose for at least 3 months before screening)). Diagnosis of active parasitic infection or having suspected parasitic infection, unless clinical and (if necessary) laboratory assessments have ruled out active infection before randomization. Chronic or acute infection requiring treatment with systemic antibiotics, antivirals, or antifungals within 1 month before screening. Use of oral antibiotics/anti‐infectives within 2 weeks before screening, Known or suspected immunosuppression, including history of invasive opportunistic infections (e.g. tuberculosis, nontuberculous mycobacterial infections, histoplasmosis, listeriosis, coccidioidomycosis, pneumocystosis, aspergillosis) despite infection resolution, otherwise recurrent infections of abnormal frequency, or prolonged infections suggesting an immunocompromised status, as judged by the investigator. Known history of human immunodeficiency virus infection. Positive or indeterminate hepatitis B surface antigen or hepatitis C antibody at screening. Elevated transaminases (alanine aminotransferase and/or aspartate aminotransferase) more than 3 times the upper limit of normal at screening. History of malignancy within 5 years before screening, except completely treated in situ carcinoma of the cervix and completely treated and resolved nonmetastatic squamous or basal cell carcinoma of the skin. History of patient‐reported alcohol or drug abuse within 6 months before screening. Any other medical or psychological condition, including relevant laboratory result abnormalities at screening, that, in the opinion of the investigator, suggest a new and/or insufficiently understood disease, may present an unreasonable risk to the study patient as a result of his/her participation in this clinical trial, may make the patient’s participation unreliable, or may interfere with study assessments. The specific justification for patients excluded under this criterion was noted in study documents (chart notes, case report form, etc). Severe concomitant illness(es) that, in the investigator’s judgment, would adversely affect the patient’s participation in the study. Planned or anticipated use of any prohibited medications or procedures during study treatment. Treatment with a live (attenuated) vaccine within 3 months before screening. Patient or his/her immediate family is a member of the investigational team. Pregnant or breastfeeding women, or women planning to become pregnant or breastfeed during the study. Women unwilling to use adequate birth control, if of reproductive potential*, and sexually active. Adequate birth control is defined as agreement to consistently practice an effective and accepted method of contraception for the duration of the study and for 120 days after the last dose of study drug; these include hormonal contraceptives, an intrauterine device, double barrier contraception (i.e. condom þ diaphragm), or male partner with documented vasectomy. *For female participants, menopause is defined as at least 12 consecutive months without menses (if in question, follicle‐stimulating hormone of 25 U/mL must be documented). Hysterectomy, bilateral oophorectomy, or bilateral tubal ligation must be documented, as applicable, and women with these documented conditions are not required to use additional contraception. **Age at beginning of study per study group:** **Age, y, mean (SD):** placebo 36.1 (12.75); dupilumab 33.1 (8.70) **Sex (m/f) per study group:** placebo: 10/14; dupilumab: 13/10 **Number randomized per study group (numbers of patients):** placebo: 24; dupilumab: 23 **Number reaching end of study per study group (numbers of patients):** placebo: 20; dupilumab: 22	
Interventions	**Study group 1:** placebo **Study group 2:** weekly subcutaneous dupilumab 300 mg (loading dose, 600 mg on day 1)	
Outcomes	**Primary outcomes of the study:** The primary efficacy endpoint was the change in SDI PRO dysphagia score from baseline to week 10 **Secondary outcomes of the study:** Secondary SDI PRO endpoints included percent change in SDI PRO score from baseline to week 10 and percentage of patients with an SDI PRO score decrease of 3 points relative to baseline at week 10, which was proposed by Straumann et al as evidence of a clinical response. Other secondary endpoints, primarily evaluated at week 12, included histologic measures of type 2 inflammation in the esophagus (as measured by esophageal intraepithelial eosinophilia), endoscopically anatomic measures of esophageal disease (i.e. exudate, rings, edema, furrows, and strictures), distensibility measures of esophageal function, and additional PROs. These endpoints were assessed by measuring percent change in peak esophageal intraepithelial eos/HPF from baseline to week 12 and change in EoE Endoscopic Reference Scoring System (EREFS) score from baseline to week 12. Other secondary efficacy endpoints were percentage of patients requiring rescue medication or a procedure (e.g. esophageal dilation) through week 12 and the PRO and quality of life endpoints of absolute and percent change in weekly Eosinophilic Esophagitis Activity Index (EEsAI) PRO score from baseline to week 10, percentage of patients with 40% improvement or > 15‐ or > 30‐point improvement in EEsAI PRO score from baseline to week 10, and change in Adult Eosinophilic Esophagitis Quality of Life (EoE‐QOL‐A) score, version 3.0 from baseline to week 12. Symptomatic remission of EoE, defined as an EEsAI score of 20 at weeks 10 and 12, was also assessed in a post hoc analysis, as were the proportions of patients who achieved both histologic (< 6 eos/hpf at week 12) and symptomatic remission (SDI score reduction of 3 points relative to baseline at week 10) and both histologic and endoscopic remission. Safety was evaluated by incidence of treatment‐emergent adverse events (TEAEs) and serious adverse events from baseline to week > 28.	
Notes	**Funding source:** This research was sponsored by Sanofi and Regeneron Pharmaceuticals, Inc. **Conflicts of interest:** These authors disclose the following: Ikuo Hirano has been a consultant for Adare, Allakos, Receptos/Celgene, Regeneron Pharmaceuticals, Shire, Gossamer, Esocap and has received research funding from Adare, Allakos, Meritage, Receptos/Celgene, Regeneron Pharmaceuticals, and Shire. Evan S. Dellon has been a consultant for Alivio, Adare, Allakos, Banner, Calypso, Enumeral, EsoCap, GlaxoSmithKline, Receptos/Celgene, Regeneron Pharmaceuticals, Robarts, and Shire; has received research funding from Adare, Allakos, Meritage, Miraca, Nutricia, Receptos/Celgene, Regeneron Pharmaceuticals, and Shire; and received educational grants from Banner and Holoclara. Jennifer D. Hamilton, Qiong Zhao, Zhen Chen, Neil N. M. Graham, Bolanle Akinlade, and Allen Radin are employees and shareholders of Regeneron Pharmaceuticals. Margaret H. Collins has been a consultant for Allakos, Receptos/Celgene, Regeneron Pharmaceuticals, and Shire and has received research funding from Receptos/Celgene, Regeneron Pharmaceuticals, and Shire. Kathryn Peterson has received research funding from Janssen, Receptos/Celgene, and Regeneron Pharmaceuticals. Mirna Chehade has been a consultant for Actelion, Allakos, and Shire and received research funding from Nutricia, Regeneron Pharmaceuticals, and Shire. Alain M. Schoepfer has been a consultant for Adare, Aptalis, Dr Falk Pharma, and Regeneron Pharmaceuticals and has received research funding from AstraZeneca, Aptalis, Dr Falk Pharma, GlaxoSmithKline, Nestlé, Novartis, Receptos/Celgene, and Regeneron Pharmaceuticals. Ekaterina Safroneeva has been a consultant for Aptalis, Novartis, Receptos/Celgene, and Regeneron Pharmaceuticals. Marc E. Rothenberg has been a consultant for AstraZeneca, Celgene, GlaxoSmithKline, NKT Therapeutics, Novartis, PulmOne, Shire, and Spoon Guru; holds equity interest in Immune Pharmaceuticals, NKT Therapeutics, PulmOne, and Spoon Guru; receives royalties from reslizumab from Teva Pharmaceutical; and is the inventor of patents owned by Cincinnati Children’s Hospital Medical Center. Gary W. Falk has received research funding from Allakos; has been a consultant for Adare and Banner; and has received research funding from Adare, Meritage, Receptos/Celgene, Regeneron Pharmaceuticals, and Shire. Gianluca Pirozzi and Leda Mannent L are employees of Sanofi and may hold stock and/or stock options in the company. Brian N. Swanson is a former employee of Sanofi and may hold stock and/or stock options in the company. The remaining author discloses no conflicts.	
***Risk of bias***		
**Bias**	**Authors' judgement**	**Support for judgement**
Random sequence generation (selection bias)	Low risk	"Using a central interactive voice/web response system randomization".
Allocation concealment (selection bias)	Low risk	"Using a central interactive voice/web response system randomization".
Blinding of participants and personnel (performance bias) All outcomes	Low risk	Blinded study drug kits coded with a medication numbering system were used, and everyone involved was blinded to all randomization assignments. Study patients and study site personnel remained blinded via the use of matching placebo.
Blinding of outcome assessment (detection bias) All outcomes	Low risk	Outcome assessors were blinded.
Incomplete outcome data (attrition bias) All outcomes	Low risk	Balanced and explained attrition that likely has no effect on outcome.
Selective reporting (reporting bias)	Low risk	The trial registry is available and all pre‐specified outcomes of interest were reported.
Other bias	Low risk	Baseline characteristics evenly distributed between treatment arms except for total IgE level.

**Table:** Hirano 2020f

***Study characteristics***		
Methods	**RCT design and number of study arms:** randomized 3 arms **Single‐center or multi‐center:** multi‐center **Countries:** USA **Study dates:** October 2011 and October 2012	
Participants	**Active EoE or inactive EoE at beginning of study:** active **EoE definition/diagnostic criteria:** histologically confirmed EoE (esophageal mucosal peak eosinophil count ≥ 24 per high‐power field (HPF) (HPF; radius = 0.275 mm; 400×) in at least one biopsied site, within 30 days prior to and 21 days after the screening visit) **Inclusion criteria:** Eligible patients between 12 and 55 years age had histologically confirmed EoE (esophageal mucosal peak eosinophil count ≥24 per high‐power field (HPF) (HPF; radius = 0.275 mm; 400×) in at least one biopsied site (proximal/mid and/or distal), within 30 days prior to and 21 days after the screening visit) Histologically confirmed prior treatment failure of a high‐dose proton pump inhibitor (PPI), defined as peak eosinophil counts ≥24 per HPF after 8 weeks of 2× standard PPI dose per investigator, and at least one of the following symptoms: chest pain or discomfort, dysphagia or food impaction continuously or intermittently present within 30 days prior to the screening visit. Specific symptom severity was not required for this study. Females of child‐bearing potential must have agreed to use adequate contraception during the study and could not be pregnant or lactating at time of enrollment Written informed consent was obtained from each participant or caregiver/parent/guardian at screening **Exclusion criteria:** Patients were excluded from participating in the study if they met any of the following criteria: Presence of any condition, other than EoE, that affected the esophageal mucosa or motility Any contraindication to completing esophagogastroduodenoscopy, including stricture that blocked the passage of a standard endoscope History or presence of Crohn’s disease, celiac disease, or other gastrointestinal inflammatory disease Use of systemic, inhaled, intranasal or high‐potency dermal topical corticosteroids during the 30 days prior to enrollment Morning serum cortisol level ≤ 5 µg/dL or use of anti‐inflammatory or immunosuppressant drugs Also per the NCT registry: Known contraindication, hypersensitivity or intolerance to corticosteroids Any physical, mental, or social condition, history of illness or laboratory abnormality that, in the investigator’s judgment, might interfere with the study procedures or the ability of the participant to adhere to and complete the study Oral or esophageal mucosal infection of any type Any medical condition in which the use of anti‐inflammatory of immunosuppressant drugs are required or may be anticipated to be required during the study History of esophageal or gastric surgery Gastrointestinal bleeding Current chronic infection, immunosuppression, immunodeficiency Alcohol or drug abuse Participation in a clinical study involving an investigations drug within 30 days of the screening visit **Age at beginning of study per study group:** Age (y), mean (SD): Placebo 29.8 (13.9); APT‐1105 (fluticasone propionate orally disintegrating tablet) 1.5 mg twice‐daily: 23.4 (11.3) APT‐1105 (fluticasone propionate orally disintegrating tablet) 3.0 mg once‐daily: 24.6 (10.6) **Sex (m/f) per study group:** Placebo: 5/3 APT‐1105 (fluticasone propionate orally disintegrating tablet) 1.5 mg twice‐daily: 4/4 APT‐1105 (fluticasone propionate orally disintegrating tablet) 3.0 mg once‐daily: 6/2 **Number randomized per study group:** Placebo: 8 APT‐1105 (fluticasone propionate orally disintegrating tablet) 1.5 mg twice‐daily: 8 APT‐1105 (fluticasone propionate orally disintegrating tablet) 3.0 mg once‐daily: 8 **Number reaching end of study per study group:** Placebo: 6 APT‐1105 (fluticasone propionate orally disintegrating tablet) 1.5 mg twice‐daily: 8 APT‐1105 (fluticasone propionate orally disintegrating tablet) 3.0 mg once‐daily: 8	
Interventions	**Study group 1:** placebo **Study group 2:** APT‐1105 (fluticasone propionate orally disintegrating tablet) 1.5 mg twice‐daily **Study Group 3:** APT‐1105 (fluticasone propionate orally disintegrating tablet) 3.0 mg once‐daily	
Outcomes	**Primary outcomes of the study:** Morning serum cortisol (change from baseline measure) (time frame: screening visit (up to 21 days), week 4, week 8 and follow‐up) Standard safety laboratory tests (time frame: screening visit (up to 21 days), week 4, week 8 and follow‐up) Treatment‐emergent adverse events collection (time frame: screening visit (up to 21 days), randomization day, week 2 and week 6 (phone visit), week 4 and week 8 (office visit), follow‐up (office visit, up to 11 days after week 8 visit)) Physical examination and vital signs collection (time frame: screening (up to 21 days), week 4, week 8 and follow‐up) **Secondary outcomes of the study:** Esophagoduodenoscopy with multiple biopsies (time frame: screening (up to 21 days) and week 8) Patient‐reported outcomes and physician global assessment (time frame: screening (up to 21 days), week 4 and 8)	
Notes	**Funding source:** This study was funded by Adare Pharmaceuticals, Inc. The study sponsor had a role in the study design, collection, analysis and interpretation of the data, as well as in the writing of the report. **Conflicts of interest:** IH has received research funding from Adare, Allakos, Meritage, Celgene/Receptos, Regeneron, Shire/Takeda; and consulting fees from Adare, Allakos, Arena, AstraZeneca, Biorasi, Celgene/Receptos, Eli Lilly, EsoCap, Gossamer Bio, Regeneron, Shire/Takeda. GMC is a consultant for Adare. ES has served as a consultant for Adare, Aptalis, Novartis, Receptos and Regeneron. MCR has no conflicts of interest to report. AS has served as consultant for AbbVie, Adare, Falk Pharma GmbH, MSD, Receptos, Regeneron, Novartis, Pfizer, Takeda and Vifor, and has received research funding from Adare, Falk Pharma GmbH, Receptos and Regeneron. GWF has received research support from Allakos, Receptos/Celgene and Regeneron, and has received research support and has served as a consultant for Adare and Shire/Takeda. GE has served as a consultant for and is currently employed by Adare.	
***Risk of bias***		
**Bias**	**Authors' judgement**	**Support for judgement**
Random sequence generation (selection bias)	Low risk	Computer‐generated randomization method.
Allocation concealment (selection bias)	Unclear risk	Allocation concealment is not reported. "Participants were given two bottles – one for the morning dose and one for the evening dose".
Blinding of participants and personnel (performance bias) All outcomes	Low risk	All personnel, participants, caregivers, and the sponsor were blinded.
Blinding of outcome assessment (detection bias) All outcomes	Low risk	The outcome assessors were blinded.
Incomplete outcome data (attrition bias) All outcomes	Low risk	Minimal and explained attrition that likely has no effect on the outcome.
Selective reporting (reporting bias)	Unclear risk	In protocol outcomes not clearly defined, i.e. specific measures.
Other bias	Low risk	No major baseline differences between groups. No other concerns.

**Table:** Hirano 2021

***Study characteristics***		
Methods	**RCT design and number of study arms:** randomized, double‐blind, placebo‐controlled trial 2 arms **Single‐center or multi‐center:** multi‐center **Countries:** United States **Study dates:** 2015 to 2019	
Participants	**Active EoE or inactive EoE at beginning of study:** active **EoE definition/diagnostic criteria: (**15 eosinophils/high‐power field (eos/hpf) from at least 2 levels of the esophagus) during screening, with dysphagia on at least 4 days in any 2 consecutive weeks during screening and in the 2 weeks before randomization measured using the DSQ **Inclusion criteria:** Patients enrolled in this trial were 11 to 55 years of age. The lower limit of the age range chosen was considered to be the minimum age at which patients with eosinophilic esophagitis (EoE) could self‐report symptoms (PRO) using the DSQ and was also the lower‐bound age for which the DSQ had been validated. In addition, pediatric patients can present with different signs and symptoms than older patients with EoE. The upper limit of the age range was chosen as 55 years because patients older than this may present with fibrostenotic disease, so they are less likely to respond to anti‐inflammatory therapy alone. Other key inclusion criteria included a history of clinical symptoms of esophageal dysfunction intermittently or continuously at screening as previously described; an absence of histologic response to 6 to 8 weeks of high‐dose proton pump inhibitor (PPI) therapy, as per consensus guidelines in effect at the time of study onset (high‐dose therapy refers to the total daily dose, which may have been administered as once‐ or twice‐daily dosing); and a stable diet for at least 3 months before screening. A PPI trial may have occurred at the time of the qualifying esophagogastroduodenoscopy (EGD); in which case, the same PPI regimen was to continue, or this may have been done previously (in which case, PPI therapy may have been stopped if there was no response to therapy based on esophageal biopsy results). If PPI responsiveness was excluded by a previous EGD and biopsy, the historical EGD and biopsy must have been performed after the patient had been on a minimum of 6 weeks of high‐dose PPI therapy. **Exclusion criteria:** Key exclusion criteria included the following: immunomodulatory therapy use 8 weeks before the qualifying EGD or anticipated use during the study; use of swallowed topical corticosteroids for EoE or systemic corticosteroids for any condition 4 weeks before the qualifying EGD and baseline or anticipated use during the study; presence of a high‐grade esophageal stricture (defined as the presence of a lesion not allowing passage of a diagnostic adult upper endoscope (insertion tube diameter > 9 mm)); or following either a pure liquid diet or a 6‐food elimination diet. In addition to having a stable (i.e. no changes) diet 3 months before screening, dosing with inhaled or nasal corticosteroids and PPIs was to be stable for a specified period of time (inhaled corticosteroids for 3 months before screening; nasal corticosteroids and PPIs for 4 weeks prior to qualifying EGD). **Age at beginning of study per study group:** **Mean age, y (SD) :** Placebo: 33.9 (12.1) Budesonide oral suspension (BOS) 2.0 mg twice‐daily: 33.8 (11.9 ) **Sex (m/f):** Placebo: 62/43 Budesonide oral suspension (BOS) 2.0 mg twice‐daily: 129/84 **Number randomized per study group:** Placebo: 107 Budesonide oral suspension (BOS) 2.0 mg twice‐daily: 215 **Number reaching end of study per study group:** Placebo: 94 Budesonide oral suspension (BOS) 2.0 mg twice‐daily:202	
Interventions	**Study group 1:** placebo **Study group 2:** budesonide oral suspension (BOS) 2.0 mg twice‐daily	
Outcomes	**Primary outcomes of the study:** The co‐primary efficacy endpoints were (1) the proportion of stringent histologic responders (6 eos/hpf across all available esophageal levels (proximal, middle, or distal)) and (2) the proportion of dysphagia symptom responders (30% reduction in DSQ score) from baseline to week 12 of therapy **Secondary outcomes of the study:** The key secondary efficacy endpoint was the change in DSQ score from baseline to week 12 of treatment. Other secondary efficacy endpoints included the proportion of full responders, defined as a combined 2 stringent histologic response and dysphagia symptom response (6 eos/hpf and 30% reduction in DSQ score); the mean change in EoE Endoscopic Reference Score (EREFS) and maximum peak eosinophil count; the proportion of patients achieving a deep histologic response or histologic response (deep histologic response, 1 eos/hpf; histologic response, < 15 eos/hpf); and the mean change in the EoE Histology Scoring System (EoEHSS) total score ratios from baseline to week 12 of therapy	
Notes	**Funding source:** This study was funded by Shire ViroPharma, Inc, a member of the Takeda group of companies. **Conflicts of interest:** "**T**he authors disclose the following: Ikuo Hirano has received research funding from Adare Pharmaceuticals, Allakos, Arena Pharmaceuticals, AstraZeneca, Meritage Pharma, Inc, Receptos/Celgene, Regeneron Pharmaceuticals, and Shire, a Takeda company; and served as a consultant for Adare Pharmaceuticals, Allakos, Arena Pharmaceuticals, AstraZeneca, EsoCap Biotech, Gossamer Bio, Lilly, Meritage Pharma, Inc, Receptos/Celgene, Regeneron Pharmaceuticals, and Shire, a Takeda company. Margaret H. Collins has received research funding from Meritage Pharma, Inc, Receptos/Celgene, Regeneron Pharmaceuticals, and Shire, a Takeda company; and served as a consultant for Allakos, Arena Pharmaceuticals, AstraZeneca, Calypso Biotech, EsoCap Biotech, GlaxoSmithKline, Receptos/Celgene, Regeneron Pharmaceuticals, Robarts Clinical Trials, Inc./Alimentiv, Inc, and Shire, a Takeda company. David A. Katzka has received research funding from Shire, a Takeda company; and served as a consultant for Receptos/Celgene. Vincent A. Mukkada has received research funding from Meritage Pharma, Inc, and Shire, a Takeda company; and served as a consultant for Shire, a Takeda company. Gary W. Falk has received research funding from Adare Pharmaceuticals, Allakos, Lucid, Receptos/Celgene, Regeneron Pharmaceuticals, and Shire, a Takeda company; and served as a consultant for Adare Pharmaceuticals, Allakos, Bristol Myers Squibb, Lucid, Regeneron Pharmaceuticals, and Shire, a ‐ 2021 Budesonide Oral Suspension for EoE 9 Downloaded for Anonymous User (n/a) at Nemours Children's Hospital Delaware from ClinicalKey.com by Elsevier on February 06, 2022. For personal use only. No other uses without permission. Copyright ©2022. Elsevier Inc. All rights reserved." Takeda company. Robin Morey, Nirav K. Desai, and James Williams are employees of Takeda Development Center Americas, Inc, and stockholders of Takeda Pharmaceutical Company Limited. Lan Lan was an employee of Takeda Development Center Americas, Inc, and a stockholder of Takeda Pharmaceutical Company Limited at the time of the study. Evan S. Dellon has received research funding from Adare Pharmaceuticals, Allakos, AstraZeneca, GlaxoSmithKline, Meritage Pharma, Inc, Miraca Life Sciences, Nutricia, Receptos/Celgene, Regeneron Pharmaceuticals, and Shire, a Takeda company; and served as consultant for Abbott Laboratories, Adare Pharmaceuticals, Aimmune Therapeutics, Allakos, Amgen, Arena Pharmaceuticals, AstraZeneca, Biorasi, Calypso Biotech, Celldex Therapeutics, Inc, EsoCap Biotech, GlaxoSmithKline, Gossamer Bio, Lilly, Parexel, Receptos/Celgene, Regeneron Pharmaceuticals, Robarts Clinical Trials, Inc/Alimentiv, Inc, Salix Pharmaceuticals, Sanofi, and Shire, a Takeda company; and received educational grants from Allakos, Banner Life Sciences, and Holoclara.	
***Risk of bias***		
**Bias**	**Authors' judgement**	**Support for judgement**
Random sequence generation (selection bias)	Low risk	Computer‐generated coded randomization method.
Allocation concealment (selection bias)	Unclear risk	Allocation concealment is not described.
Blinding of participants and personnel (performance bias) All outcomes	Low risk	The study team, study sties, and patients were blinded. An unblinded and independent data team handled data processing, review and validation.
Blinding of outcome assessment (detection bias) All outcomes	Low risk	Outcome assessors were blinded.
Incomplete outcome data (attrition bias) All outcomes	Low risk	Balanced and explained attrition that likely has no effect on outcome.
Selective reporting (reporting bias)	Low risk	Trial protocol is available and all pre‐specified outcomes of interest were reported in the pre‐specified way.
Other bias	Low risk	No major baseline differences between groups. No other concerns.

**Table:** Kliewer 2019

***Study characteristics***		
Methods	**RCT design and number of study arms:** RCT; 2 groups for 12 weeks ‐ A and B. Non‐responders at 12 weeks go from A to B; and B go to C (1‐food, 4‐food, steroids are ABC) **Single‐center or multi‐center:** multi‐center **Countries**: USA **Study dates:** March 2016 to May 2018	
Participants	**Active EoE or inactive EoE at beginning of study**: active EoE (> 15 eos/hpf) **EoE definition/diagnostic criteria:** > 15 eos/hpf **Inclusion criteria:** Have diagnosis of EoE (based on consensus criteria) Aged 6 to 17 years Have histologically confirmed active disease > 15 eosinophils/hpf in either distal or proximal esophagus within 12 weeks of screening visit PPI confirmation Symptomatic (have experienced symptoms within the last month prior to enrollment) Has a negative urine pregnancy test at screening if of childbearing potential. Females of childbearing potential must have a negative urine pregnancy test (β‐hCG) prior to enrollment into the study (i.e. at screening). Subsequently, these participants must agree to use adequate birth control measures (e.g. condom, oral/injectable/subcutaneous contraceptives, intrauterine device, or sexual abstinence) during the study and for at least 1 month after the last dose of study drug which will be documented in the source documents. **Exclusion criteria:** Have been treated with topical swallowed steroids within the last 2 months or systemic steroids within the past 3 months Have eosinophilia in segments of the GI tract other than the esophagus Have been diagnosed with a GI malabsorption disorder (i.e. inflammatory bowel disease, Crohn's disease) or celiac disease Are currently on dietary therapy avoiding milk Have concurrent *H. pylori* gastritis or parasitic infection Are unable to obtain esophagogastroduodenoscopy with esophageal biopsies at Cincinnati Children's Hospital Medical Center (CCHMC) or other participating institution within 4 weeks of study completion Have previously failed (in a clinical trial setting) dietary therapy with one of these regimens or topical steroid treatment with fluticasone at a total dose of 1760 µg per day Have definitely responded (in a clinical trial setting) to either dietary therapy avoiding these antigens or to swallowed fluticasone at a total dose of 1760 µg per day Are concurrently receiving any of the prohibited medications listed in Table 2 On immunotherapy for pollen (if not on maintenance therapy) or immunoglobulin E (IgE)‐mediated food allergy **Age at beginning of study per study group**: 6 to 17 years **Sex (m/f) per study group**: NR **Number randomized per study group**: group A and 25 group B **Number reaching end of study per study group**: 33 group A; 16 group B. Data NA in published literature on A to B and B to C.	
Interventions	Study group 1 (control or placebo): no control group Group A got 1 food, B got 4 foods and C was steroids	
Outcomes	**Primary outcome measures:** Change from baseline in Pediatric EoE Symptom Score Version 2.0 (PEESS V2.0) at 12 weeks (time frame: baseline and 12 weeks) Within‐group comparisons (baseline vs week 12) of PEESS V2.0 scores (time frame: baseline and 12 weeks) **Secondary outcome measures:** Percent of participants in histologic remission (< 15 eosinophils per high‐power field) at 12 weeks (time frame: 12 weeks) Percent of participants on swallowed glucocorticoids (SGC) in histologic remission (< 15 eos/hpf) at 12 weeks in phase 2 (time frame: 12 weeks) Percent of 1FED non‐responders on 4FED in histologic remission (< 15 eos/hpf) at 12 weeks in phase 2 (time frame: 12 weeks) Change from baseline in Pediatric Quality of Life Inventory Version 3.0 EoE Module (PedsQL 3.0EoE) at 12 weeks (time frame: baseline and 12 weeks) Change from baseline in Pediatric Quality of Life Inventory Version 4.0 (PedsQL 4.0) Generic CoreScales at 12 weeks (time frame: baseline and 12 weeks) Change from baseline in Endoscopic Reference Score at 12 weeks (time frame: baseline and 12 weeks) Percent of participants with positive and negative milk skin prick tests responding to 1FED (time frame: baseline and 12 weeks)	
Notes	**Funding source**: Patient‐Centered Outcomes Research Institute **Conflicts of interest:** NR, several of the authors are conflicted	
***Risk of bias***		
**Bias**	**Authors' judgement**	**Support for judgement**
Random sequence generation (selection bias)	Low risk	The author confirmed "a computer‐generated randomization schedule was used (SAS random number generator)".
Allocation concealment (selection bias)	Low risk	The author confirmed that "the Data Management Coordinating Center who was not involved in the study allocated the participants to a treatment arm. The DMCC generated an electronic notification (email) to the study coordinator at randomization".
Blinding of participants and personnel (performance bias) All outcomes	High risk	This was an open‐label study.
Blinding of outcome assessment (detection bias) All outcomes	Low risk	The author confirmed that "the pathologists were blinded to the treatment allocation. The endoscopist was not actively blinded to treatment allocation".
Incomplete outcome data (attrition bias) All outcomes	Unclear risk	Slightly more people withdrew their participation from the 4FED group (n = 6) than the 1FED group (n = 2) during phase 1. In total 34 people completed 4FED compared to 17 completing 1FED. This might have impacted our outcomes.
Selective reporting (reporting bias)	Low risk	The results have been posted on the trial registry website and have been appropriately reported for the pre‐cross‐over phase 1 of the trial.
Other bias	Low risk	No major baseline differences between groups. No other concerns.

**Table:** Kliewer 2021

***Study characteristics***		
Methods	**RCT design and number of study arms:** RCT, 2 arms **Single‐center or multi‐center:** multi‐center, 10 sites **Countries:** USA **Study dates:** May 2016 to May 2019	
Participants	**Active EoE or inactive EoE at beginning of study:** active EoE **EoE definition/diagnostic criteria:** ≥ 15 eos/hpf + symptoms, and lack of PPI response **Inclusion criteria:** Have diagnosis of EoE (based on consensus criteria) Have histologically confirmed active disease > 15 eosinophils/hpf in either distal or proximal esophagus within 12 weeks of screening visit Symptomatic (have experienced symptoms within the last month prior to enrollment) Proton pump inhibitor (PPI) confirmation Have a negative urine pregnancy test at screening if of childbearing potential **Exclusion criteria:** Have been treated with topical swallowed steroids within the last 2 months or systemic steroids within the last 3 months Have pathological eosinophilia in segments of the GI tract other than the esophagus determined by local review Have been diagnosed with a GI malabsorption disorder (i.e. inflammatory bowel disease, Crohn's disease) or celiac disease Are currently on dietary therapy strictly avoiding milk or on a 6FED Have concurrent *H. pylori* gastritis or parasitic infection Have history of anaphylaxis to milk (with current avoidance of milk) Have previously failed strict dietary therapy clearly documented with one of these regimens or topical steroid treatment (i.e. have achieved histological remission of < 15 eos/hpf after having been on fluticasone or > 1 mg budesonide per day) Use of investigational drugs within 4 weeks (one month) prior to enrollment Are concurrently receiving any of the prohibited medications for the study On immunotherapy for pollen (if not on maintenance therapy) or immunoglobulin‐E (IgE)‐mediated food allergy Past or current medical problems or findings from physical examination or laboratory testing that are not listed above, which, in the opinion of the investigator, may pose additional risks from participation in the study, may interfere with the participant's ability to comply with study requirements or that may impact the quality or interpretation of the data obtained from the study. **Age at beginning of study per study group:** 1‐food elimination: 36.4 (10.2) 6‐food elimination: 37.8 (10.4) **Sex (m/f) per study group:** 1‐food elimination: 55%, 37/67, m/f 6‐food elimination: 53%, 33/62, m/f **Number randomized per study group:** 1‐food elimination: 67 6‐food elimination: 62 **Number reaching end of study per study group:** NR	
Interventions	**Study groups:** 1‐food elimination: animal milk 6‐food elimination: animal milk, wheat, egg, soy, tree nuts/peanuts, seafood	
Outcomes	**Primary outcome measures:** Percent of participants in histologic remission (< 15 eos/hpf) at 6 weeks **Secondary outcome measures:** Percent of participants in complete (≤ 1 eos/hpf) and partial histologic remission (2 to 14 eos/hpf) at 6 weeks Percent of participants in histologic remission following SGC in Phase 2 at 6 weeks Percent of participants in histologic remission following 6FED in Phase 2 at 6 weeks Change from baseline in peak eosinophil count at 6 weeks Change from baseline in EoE histology scoring system (EoEHSS) at 6 weeks Change from baseline in endoscopic reference score (EREFS) at 6 weeks Change from baseline in EoE Symptom Activity Index (EEsAI) at 6 weeks Change from baseline in quality of life (EoE‐QoL‐A) at 6 weeks	
Notes	**Funding source:** NIDDK NIAID NCATS, Office of Rare Diseases **Conflict of Interest**: NR	
***Risk of bias***		
**Bias**	**Authors' judgement**	**Support for judgement**
Random sequence generation (selection bias)	Low risk	The author confirmed "a computer‐generated randomization schedule was used (SAS random number generator)".
Allocation concealment (selection bias)	Low risk	The author confirmed that "the Data Management Coordinating Center who was not involved in the study allocated the participants to a treatment arm. The DMCC generated an electronic notification (email) to the study coordinator at randomization".
Blinding of participants and personnel (performance bias) All outcomes	High risk	This was an open‐label study.
Blinding of outcome assessment (detection bias) All outcomes	Low risk	The primary outcome was histologic response determined by blinded central pathology review.
Incomplete outcome data (attrition bias) All outcomes	Low risk	All participants completed the study.
Selective reporting (reporting bias)	Low risk	Protocol is available and all pre‐specified outcomes of interest were reported in the pre‐specified way, however 2 additional secondary outcomes were added to the study that were not included in the initial protocol, one of which was our secondary outcome of quality of life (using EoE‐QoL‐A). However, we did not think this could potentially bias our outcome results.
Other bias	Unclear risk	Chest pain is significantly higher in the 1‐food group relative to the 6‐food group at baseline. 85% of the participants in the 1‐food group and 74% of the participants in 6‐food received an endoscopy. Peak eos/hpf are 1.4‐fold higher in 1‐food group relative to the 6‐food group at baseline.

**Table:** Konikoff 2006

***Study characteristics***		
Methods	**RCT design and number of study arms:** double‐blind RCT, 2 arms **Single‐center or multi‐center:** Cincinnati Children’s Hospital Medical Center; 2 patients were also enrolled at Children’s Hospital, San Diego **Countries:** USA **Study dates:** 10 January 2003 and 16 August 2005	
Participants	**Active EoE or inactive EoE at beginning of study:** active **EoE definition/diagnostic criteria:** eosinophilic esophagitis was defined as the presence of ≥ 24 eosinophils in any 400x HPF in at least one biopsy specimen from either the proximal or distal esophagus and the presence of epithelial hyperplasia after careful examination of all microscopic fields **Inclusion criteria:** 3 and 30 years the presence of ≥ 24 eosinophils in any 400x HPF in at least one biopsy specimen from either the proximal or distal esophagus and the presence of epithelial hyperplasia after careful examination of all microscopic fields **Exclusion criteria:** Patients were excluded from the study if they had a history of poor tolerance to fluticasone propionate, were unable to co‐operate with the use of a metered‐dose inhaler, were pregnant, or had taken any corticosteroid (including inhaled, nasal, or systemic) within 3 months **Age at beginning of study per study group:** Group: age mean (SE, range) Fluticasone propionate: 8.5 (0.8, 3 to 16) Placebo: 11.2 (1.3, 3 to 18) **Sex (m/f) per study group:** Fluticasone propionate: M/F (M%) = 17/4 Placebo: M/F (M%) = 9/6 **Number randomized per study group:** Fluticasone propionate: n = 21 Placebo: n = 15 **Number reaching end of study per study group:** Fluticasone propionate: 20/21 (95%) Placebo: 11/15 (73%)	
Interventions	**Study group 1:** placebo twice‐daily for 3 months via metered dose inhaler **Study group 2:** fluticasone propionate (400 µg twice‐daily for 3 months) via metered dose inhaler and swallowed	
Outcomes	**Primary outcomes of the study:** histological remission (≤ 1 eosinophil per hpf) **Secondary outcomes of the study:** adverse effects	
Notes	**Funding**: supported by the Burroughs Wellcome Fund, the CURED Foundation, the Buckeye Foundation, an American Academy of Allergy Asthma & Immunology/Sanofi‐Aventis Women Physician in Allergy grant, and a grant from the US Public Health Service (NIH T32 DK007727) **Conflicts of interest:** NR	
***Risk of bias***		
**Bias**	**Authors' judgement**	**Support for judgement**
Random sequence generation (selection bias)	Low risk	Computer‐generated randomization method.
Allocation concealment (selection bias)	Low risk	"A clinical research coordinator dispensed metered‐dose inhalers containing either active drug or placebo to each patient according to a computer‐generated randomization list".
Blinding of participants and personnel (performance bias) All outcomes	Low risk	All participants and study personnel (with the exception of the above clinical research co‐ordinator) were blinded to treatment assignment for the duration of the study. "Only the study statisticians had access to the unblinded data, but they did not have contact with study participants".
Blinding of outcome assessment (detection bias) All outcomes	Low risk	All participants and study personnel (with the exception of the above clinical research co‐ordinator) were blinded to treatment assignment for the duration of the study. "Only the study statisticians had access to the unblinded data, but they did not have contact with study participants".
Incomplete outcome data (attrition bias) All outcomes	Low risk	Minimal and explained attrition that likely has no effect on outcomes.
Selective reporting (reporting bias)	Low risk	"The primary outcome measure [...] was complete histologic response to treatment. Secondary outcome measures included presence of endoscopic furrowing, presence of epithelial hyperplasia, and presence of clinical symptoms". The outcomes were all reported.
Other bias	Low risk	No major baseline differences between groups. No other concerns.

**Table:** Lieberman 2018

***Study characteristics***		
Methods	**RCT design and number of study arms:** RCT, 2 arms **Single‐center or multi‐center:** single‐center **Countries:** USA **Study dates:** December 2014 to December 2017	
Participants	**Active EoE or inactive EoE at beginning of study:** active **EoE definition/diagnostic criteria:** ≥ 15 eosinophils per high‐power field (eos/hpf) following at least 8 weeks of high‐dose PPI therapy and a normal esophageal pH probe **Inclusion criteria:** Diagnosis of eosinophilic esophagitis (previously PPI non‐responsive) Age 2 to 17 years of age **Exclusion criteria:** Concomitant treatment with swallowed corticosteroids (any prior use of swallowed corticosteroids required an 8‐week washout period) Pregnancy Evidence of pathologic eosinophilia in other locations in the gastrointestinal tract Active participation in another research protocol Renal or hepatic insufficiency **Age at beginning of study per study group:** (mean (SD)) Cromolyn: 10.4 (4.1) Placebo: 12.8 (3.7) **Sex (m/f) per study group:** Cromolyn: 4/5 (m/f) Placebo: 4/7 (m/f) **Number randomized per study group:** Cromolyn: 9 Placebo: 7 **Number reaching end of study per study group:** Cromolyn: 9/9 Placebo: 6/7	
Interventions	**Study group 1:** saline ampules, participants 2 to 12 years of age ‐ 1 ampule mixed with 1 teaspoon of sugar 4 times daily, participants 13 to 18 years of age ‐ 2 ampules mixed with 2 teaspoons of sugar 4 times daily **Study group 2:** participants 2 to 12 years of age ‐ 100 mg of cromolyn ‐ 1 ampule mixed with 1 teaspoon of sugar 4 times daily, participants 13 to 18 years of age ‐ 200 mg cromolyn ‐ 2 ampules mixed with 2 teaspoon of sugar 4 times daily	
Outcomes	**Primary outcomes of the study:** Change in peak esophageal eosinophilia after 8 weeks of therapy **Secondary outcomes of the study:** Change in PEESS score at 4 and 8 weeks of therapy Adverse events from therapy	
Notes	**Funding source:** American College of Allergy, Asthma, and Immunology Young Investigator Award (to Dr Lieberman) Drug provided for free by MEda Pharmaceuticals (Somerset, NJ) **Conflicts of interest:** Dr Lieberman is an associate editor for Annals of Allergy, Asthma, & Immunology He has served on the advisory board for Aimmune Therapeutics He is a principal investigator for clinical trials sponsored by Aimmune Therapeutics and Biotest Pharma Drs Zhang, Whitworth, and Cavender have no conflicts of interest to report	
***Risk of bias***		
**Bias**	**Authors' judgement**	**Support for judgement**
Random sequence generation (selection bias)	Low risk	The author confirmed that they performed a computer‐generated randomization.
Allocation concealment (selection bias)	Low risk	The author confirmed that "a research pharmacist had the blinded randomization schedule. She was not involved in any other part of study. She was contacted when someone was enrolled and allocated treatment based on the blinded computer generated sequence that only she had".
Blinding of participants and personnel (performance bias) All outcomes	Low risk	The author confirmed that "study drug was matched to placebo in terms of the liquid vials as much as possible. Investigator never saw study drug subject received. Assignment was blinded to subject and investigators during the trial until study completion".
Blinding of outcome assessment (detection bias) All outcomes	Low risk	The author confirmed that outcome assessors were blinded.
Incomplete outcome data (attrition bias) All outcomes	Low risk	Low and explained attrition that likely has no effect on outcomes.
Selective reporting (reporting bias)	Low risk	Trial protocol is available and all pre‐specified outcomes of interest were reported in the pre‐specified way.
Other bias	Low risk	No major baseline differences between groups. No other concerns.

**Table:** Lucendo 2019

***Study characteristics***		
Methods	**RCT design and number of study arms:** double‐blind, randomized, placebo‐controlled, 2 study arms **Single‐center or multi‐center:** multi‐center **Countries:** 26 centers in 6 countries (Belgium, Germany, Netherlands, Spain, Switzerland, UK) **Study dates:** November 2015 to October 2016	
Participants	**Active EoE or inactive EoE at beginning of study:** adults with active EoE **EoE definition/diagnostic criteria:** clinico‐histologic active EoE: Patients had to have a severity of 4 points on a 0 to 10 numerical rating scale (NRS) for either dysphagia or odynophagia for 1 day in the week before randomization. Additionally, Patient’s Global Assessment (PatGA) of EoE activity was to be 4 points on a 0 to 10 NRS. Histologic activity with peak eos ≥ 65/mm^2^ hpf in at least 1 hpf (corresponding to 20 eos/hpf), as measured in a total of 6 hpf derived from 6 biopsies, 2 each from the proximal, mid, and distal segments of the esophagus **Inclusion criteria:** aged 18 to 75 years with clinico‐histologic active EoE and refractory to treatment with a PPI for a 4‐week period severity of ≥ 4 points on NRS for dysphagia or odynophagia PatGA was ≥ 4 points. Histologic activity with peak EOS ≥ 65/mm^2^ hpf in at least 1 hpf. **Exclusion criteria:** Clinical and endoscopic suspicion for GERD Achalasia/scleroderma Evidence of causes other than EoE for esophageal eosinophilia Pathologic eosinophilic infiltration in gastric and duodenal biopsies History of esophageal surgery at any time/esophageal dilation procedures within the last 8 weeks before screening Any relevant systemic disease Systemic glucocorticosteroids, immunosuppressants, biologic drugs within 4 weeks before screening/topical glucocorticosteroids within 2 weeks before screening and onset of dietary restrictions within 4 weeks before screening **Age at beginning of study per study group:** y, mean (SD): budesonide orodispersible tablet (BOT): 37 (11.5); placebo: 37 (9.2) **Sex (m/f) per study group:** male, n (%): BOT: 48/59 (81%); placebo: 25/29 (86%) **Number randomized per study group:** 59 intervention group; 29 placebo group **Number reaching end of study per study group:** 56 intervention group; 25 placebo group	
Interventions	**Study group 1:** matching placebo **Study group 2:** budesonide orodispersible tablet 1 mg twice‐daily x 6 weeks, if no remission by 6 weeks were offered 6 weeks of open‐label treatment with BOT	
Outcomes	**Primary outcomes of the study:** Primary outcomes: clinical remission (including dysphagia and odynophagia severity + histologic remission) **Secondary outcomes of the study:** Secondary outcomes: histologic remission, change in peak eosinophil count, resolution of symptoms on each day in the week before the EoT and rate of clinical remission (EEsAI‐PRO ≤ 20 at EoT) **Further secondary efficacy variables** Clinical weekly sum of daily 0 to 10 NRS dysphagia (range: 0 to 70) Rate of patients with overall symptoms resolution defined as PatGA 2 at week 6 (LOCF) Change from baseline to week 6 (LOCF) in blood eosinophil counts (eos/mm^3^) Change from baseline to week 6 (LOCF) in total modified EREFS endoscopic score Change from baseline to week 6 (LOCF) in modified EREFS inflammatory signs subscore (0 to 4) Change from baseline to week 6 (LOCF) in modified EREFS fibrotic signs subscore (0 to 4) Rate of patients with histologic remission (i.e. peak eos < 48/mm^2^ hpf; equivalent to < 15 eos/hpf) at week 6 (LOCF) Change from baseline to EoT DB phase in modified SHS symptom burden Change from baseline to EoT DB phase in modified SHS social function Change from baseline to EoT DB phase in modified SHS disease–related worry Change from baseline to EoT DB phase in modified SHS general well‐being Change from baseline to EoT DB phase in EoE‐QoL‐A 30 items (weighted average) Change from baseline to EoT DB phase in EoE‐QoL‐A 24 items (weighted average) Change from baseline to EoT DB phase in EoE‐QoL‐A eating/diet impact 10 items (weighted average) Change from baseline to EoT DB phase in EoE‐QoL‐A eating/diet impact 4 items (weighted average) Change from baseline to EoT DB phase in EoE‐QoL‐A social impact (weighted average) (weighted average) Change from baseline to EoT DB phase in EoE‐QoL‐A emotional impact (weighted average) Change from baseline to EoT DB phase in EoE‐QoL‐A disease anxiety (weighted average) Change from baseline to EoT DB phase in EoE‐QoL‐A swallowing anxiety (weighted average)	
Notes	**Funding source:** Dr Falk Pharma GmbH, Freiburg, Germany funded this study and contributed to the design and conduct of the study; collection, management, analysis and scientific interpretation of the data; supported the manuscript preparation and reviewed the manuscript for medical and scientific accuracy. Approval of the manuscript, and the decision to submit the manuscript for publication was the responsibility of the authors. **Conflicts of interest:** These authors disclose the following: Alfredo J Lucendo has received research funding from Dr Falk Pharma; Stephan Miehlke is a member of advisory boards for Celgene and EsoCap and has received speaker’s fee from Dr Falk Pharma GmbH and Falk Foundation; Christoph Schlag has received consultant fees from EsoCap and speaker fees, travel and research funding from Dr Falk Pharma GmbH; Michael Vieth has received speaker and consultant fees from Dr Falk Pharma GmbH; Ulrike von Arnim is a member of MSD national advisory board, has received speaker fees from AbbVie, MSD, Falk Foundation, Pfizer, Takeda, and Vifor; Javier Molina‐Infante has received speaker and consultant fees from Dr Falk Pharma GmbH; Dirk Hartmann has no conflicts of interest to declare; Albert Jan Bredenoord has received research funding from Nutricia, Norgine, and Bayer and received speaker and/or consulting fees from Laborie, EsoCap, Diversatek, Medtronic, Dr Falk Pharma GmbH, Calypso, Thelial, Regeneron, Celgene, Bayer, Norgine, AstraZeneca, Almirall, and Allergan; Constanza Ciriza de los Rios has received speaker fees from Casen Recordati; Ahmed Madisch has received speaker fees from Dr Falk Pharma GmbH and Falk Foundation; Jamal Hayat has received speaker fees from Dr Falk Pharma GmbH; Stephen Attwood has received speaker and consulting fees from Dr Falk Pharma GmbH; Ralph Mueller and Roland Greinwald are employees of Dr Falk Pharma GmbH; Alain Schoepfer is a member of an advisory board for Dr Falk Pharma GmbH, Adare Pharmaceuticals, Celgene Pharmaceuticals, and Regeneron Pharmaceuticals. He has received research funding from Dr Falk Pharma GmbH, Adare Pharmaceuticals, Celgene Pharmaceuticals, and Regeneron Pharmaceuticals. He has received speaker’s fees from Dr Falk Pharma GmbH and Celgene Pharmaceuticals; Alex Straumann is a consultant of Calypso, EsoCap, Dr Falk Pharma GmbH, GSK, Receptos‐Celgene, Regeneron‐Sanofi, Shire and Tillotts, and has received speaker fees and research funding from Dr Falk Pharma GmbH. The remaining authors disclose no conflicts.	
***Risk of bias***		
**Bias**	**Authors' judgement**	**Support for judgement**
Random sequence generation (selection bias)	Low risk	Computer‐generated randomization method
Allocation concealment (selection bias)	Low risk	"Allocation concealment was ensured as patients, investigators and their study team, the sponsor, monitoring staff, central laboratory, and central pathologist, were all kept blinded to the randomization sequence, the block size, and patient’s treatment, until all patients had completed the study and the database was clean and locked. No individual unblinding was needed or performed".
Blinding of participants and personnel (performance bias) All outcomes	Low risk	All members of the study team and the participants were blinded for the study. BOT and corresponding placebo were identical in physical appearance and were administered twice‐daily
Blinding of outcome assessment (detection bias) All outcomes	Low risk	The study team including the central pathologist were blinded for the study.
Incomplete outcome data (attrition bias) All outcomes	Low risk	There was no attrition imbalance, and the attrition was explained.
Selective reporting (reporting bias)	Low risk	Trial protocol is available and all pre‐specified outcomes of interest were reported.
Other bias	Low risk	No major baseline differences between groups. No other concerns.

**Table:** Miehlke 2016

***Study characteristics***		
Methods	**RCT design and number of study arms:** RCT design, double‐blind, double‐dummy, 4 arms **Single‐center or multi‐center:** multi‐center **Countries:** Switzerland, Germany, and Belgium **Study dates:** June 2011 to April 2013	
Participants	**Active EoE or inactive EoE at beginning of the study:** active EoE **EoE definition/diagnostic criteria:** clinical symptoms of esophageal dysfunction (dysphagia score ≥ 3), peak eosinophils (eos) ≥ 65/mm2 high‐power fields (hpf) in at least 1 hpf (corresponding to ≥ 20 eos/hpf), and eosinophilic tissue infiltration with a mean cell density ≥ 16 eos/mm2, as measured in a total of 30 hpf derived from 6 biopsies, 2 each from the proximal, mid, and distal segments of the esophagus **Inclusion criteria:** Patients between 18 and 75 years of age and confirmed clinicopathological diagnosis of EoE according to the above criteria **Exclusion criteria:** Clinical and endoscopic suspicion for GERD, achalasia, or scleroderma History of abnormal pH monitoring of the distal esophagus or clinicopathological response to a treatment with proton pump inhibitors (PPIs) at a standard dose with a treatment duration of at least 2 weeks Other clinical evidence of causes other than EoE for esophageal eosinophilia Any concomitant esophageal disease and relevant GI disease History of esophageal surgery at any time or of esophageal dilation procedures within the last 8 weeks prior to screening Any relevant systemic disease if careful medical monitoring was not ensured Abnormal hepatic function, liver cirrhosis, or portal hypertension Abnormal renal function History of cancer in the last 5 years Upper GI bleeding within 8 weeks prior to screening Systemic therapies for any reason that may have affected assessment of primary and secondary end points (i.e. systemic glucocorticoids, histamine antagonists, mast cell stabilizers, leukotriene receptor antagonists, biologics, immunosuppressants) concomitantly or within 4 weeks prior to screening Treatment with topical therapies for any reason that may affect assessment of primary and secondary end points (i.e. topical glucocorticoids, inhaled sodium cromoglycate) concomitant or within 2 weeks prior to screening; concomitant therapy for more than 3 days with drugs, which might influence hepatic biotransformation (CYP3A inducers/inhibitors) Installation of dietary restrictions within 4 weeks prior to screening or during treatment Intake of grapefruit‐containing food or beverages during the study treatment phase Known intolerance/hypersensitivity to study drug; lack of patient’s co‐operation Existing or intended pregnancy or breastfeeding and positive pregnancy test at screening in women with childbearing potential **Age at beginning of study per study group:** mean (SD) Placebo: 36.3 (9.9) BET (budesonide effervescent tablet) 2 x 1 mg/day: 38.9 (12.6) BET (budesonide effervescent tablet) 2 x 2 mg/day: 37.2 (13.9) BVS (budesonide viscous suspension): 2 x 2 mg/day: 46.5 (14.1) **Sex (m/f) per study group:** Placebo: 16/3 BET (budesonide effervescent tablet) 2 x 1 mg/day: 17/2 BET (budesonide effervescent tablet) 2 x 2 mg/day: 16/3 BVS (budesonide viscous suspension): 2 x 2 mg/day: 14/5 **Number randomized per study group:** Placebo: 19 BET (budesonide effervescent tablet) 2 x 1 mg/day: 19 BET (budesonide effervescent tablet) 2 x 2 mg/day: 19 BVS (budesonide viscous suspension): 2 x 2 mg/day: 19 **Number reaching end of study per study group:** Placebo: 19 BET (budesonide effervescent tablet) 2 x 1 mg/day: 19 BET (budesonide effervescent tablet) 2 x 2 mg/day: 19 BVS (budesonide viscous suspension): 2 x 2 mg/day: 18	
Interventions	**Group 1:** Placebo for 2 weeks **Group 2:** BET (budesonide effervescent tablet) 2 x 1 mg/day for 2 weeks **Group 3:** BET (budesonide effervescent tablet) 2 x 2 mg/day for 2 weeks **Group 4:** BVS (budesonide viscous suspension): 2 x 2 mg/day for 2 weeks	
Outcomes	**Primary outcomes of the study:** Co‐primary outcome: rate of histological remission (mean of < 16 eos/mm^2^ hpf) Co‐primary outcome: change in the mean numbers of eos/mm2 HPF (eosinophil load) from baseline **Secondary outcomes of the study:** Endoscopic abnormality score, endoscopic intensity score and its subscores, endoscopic VAS score, dysphagia score, patient’s acceptance and preference of study drugs, adverse events, morning serum cortisol, and assessment of tolerability by investigator and patient	
Notes	**Funding source**: Dr Falk Pharma GmbH, Freiburg, Germany **Conflicts of interes**t: SM has received speaker’s honoraria from Dr Falk Pharma GmbH. MV, MB, AM, HW, HDA and MR have received speaker’s honoraria from the Falk Foundation. SS has received speaker’s honoraria from Abbvie, the Falk Foundation and MSD. RM, KD and RG are employees of Dr Falk Pharma GmbH. AS is a consultant to Dr Falk Pharma GmbH and has received consulting fees and/or speaker fees and/or research grants from Actelion, AG, Switzerland, AstraZeneca, AG, Switzerland, Aptalis Pharma, Glaxo SmithKline, AG, Nestlé S. A., Switzerland, Novartis, AG, Switzerland, Pfizer, AG, and Regeneron Pharmaceuticals	
***Risk of bias***		
**Bias**	**Authors' judgement**	**Support for judgement**
Random sequence generation (selection bias)	Low risk	Computer‐generated randomization method.
Allocation concealment (selection bias)	Low risk	There was central allocation of treatment.
Blinding of participants and personnel (performance bias) All outcomes	Low risk	Double‐dummy strategy to maintain blinding when using different pharmaceutical preparations.
Blinding of outcome assessment (detection bias) All outcomes	Low risk	Double‐dummy strategy to maintain blinding when using different pharmaceutical preparations. As the personnel used a double‐dummy strategy, outcome assessment should be blind. "The biopsies were immediately placed into separate tubes with neutral‐pH‐buffered 4% paraformaldehyde solution and sent to the primary central pathologist". Histology seems to have been blinded to the pathologies but endoscopy was assessed for endoscopic appearance by a gastroenterologist. Details whether the gastroenterologists performing endoscopy was involved or not were not provided.
Incomplete outcome data (attrition bias) All outcomes	Low risk	Attrition was minimal and explained.
Selective reporting (reporting bias)	Unclear risk	The authors did not report clinical response as defined in outcomes. They also stated significance in clinical outcome without numerical results.
Other bias	Low risk	No major baseline differences between groups. No other concerns.

**Table:** Moawad 2013

***Study characteristics***		
Methods	**RCT design and number of study arms:** single (investigator)‐blinded RCT, 2 arms **Single‐center or multi‐center:** single‐center, Esophageal Clinic at Mayo Clinic in Rochester Minnesota **Countries:** USA **Study dates:** April 2008 to October 2010	
Participants	**Active EoE or inactive EoE at beginning of the study:** active **EoE definition/diagnostic criteria:** one clinical symptom of esophageal dysfunction (dysphagia, food impaction, heartburn) with ≥ 15 eosinophils/hpf **Inclusion criteria:** Adult patients (age ≥ 18 years) All patients had at least one clinical symptom of esophageal dysfunction (dysphagia, food impaction, heartburn) with ≥ 15 eosinophils/HPF on index endoscopy **Exclusion criteria:** Secondary hypereosinophilic disorders Severe coagulopathy Pregnancy **Age at beginning of study per study group** (mean (SD), years)**:** Esomeprazole: 37.0 ± 11.1 Fluticasone: 38.0 ± 8.8 **Sex (m/f) per study group:** Esomeprazole: 19/2 Fluticasone: 19/2 **Number randomized per study group:** Esomeprazole: 21 Fluticasone: 21 **Number reaching end of study per study group:** Esomeprazole: 21 Fluticasone: 21	
Interventions	**Study group 1:** esomeprazole 40 mg once daily for 8 weeks **Study group 2:** fluticasone proportionate 440 µg twice‐daily for 8 weeks (metered dose inhaler)	
Outcomes	**Primary outcome**: histological response defined as achieving < 7 eos/hpf in both proximal and distal esophageal biopsies following 8 weeks of treatment **Secondary outcomes**: symptomatic change in dysphagia (score from the Mayo Dysphagia Questionnaire); change in endoscopic and other histological findings	
Notes	**Funding source:** none **Conflicts of interest:** none	
***Risk of bias***		
**Bias**	**Authors' judgement**	**Support for judgement**
Random sequence generation (selection bias)	Low risk	Computer‐generated randomization method.
Allocation concealment (selection bias)	Low risk	Sealed, opaque envelopes containing data on the sequence of randomization were maintained by a research pharmacist.
Blinding of participants and personnel (performance bias) All outcomes	High risk	A group of patients taking an oral tablet and the other taking an inhaler.
Blinding of outcome assessment (detection bias) All outcomes	Low risk	The author confirmed that endoscopists and pathologists were blinded.
Incomplete outcome data (attrition bias) All outcomes	Low risk	Minimal and explained attrition that likely has no effect on outcome.
Selective reporting (reporting bias)	Low risk	Trial protocol is available and all pre‐specified outcomes of interest were reported in the pre‐specified way.
Other bias	Low risk	No major baseline differences between groups. No other concerns.

**Table:** Oliva 2018

***Study characteristics***		
Methods	**RCT design and number of study arms:** RCT **Single‐center or multi‐center:** single‐center **Countries:** Italy **Study dates:** during 2 years (not specified)	
Participants	**Active EoE or inactive EoE at beginning of study:** active EoE **EoE definition/diagnostic criteria:** not specified **Inclusion criteria:** Active Pediatric **Exclusion criteria:** NR **Age at beginning of study per study group:** not reported (abstract) **Sex (m/f) per study group:** not reported **Number randomized per study group:** not reported (of 74 patients evaluated, 64 were enrolled, but 50 completed the study and were considered for the final analysis) **Number reaching end of study per study group:** NR	
Interventions	**Study group 1:** 6‐food elimination diet **Study group 2:** swallowed fluticasone **Study group 3:** swallowed budesonide **Study group 4**: oral viscous budesonide	
Outcomes	**Primary outcomes of the study:** percentage of histological responders (defined as < 15 eos/hpf) **Secondary outcomes of the study:** clinical symptom score, endoscopic score (not specified)	
Notes	**Funding source:** NR **Conflicts of interest:** NR	
***Risk of bias***		
**Bias**	**Authors' judgement**	**Support for judgement**
Random sequence generation (selection bias)	Unclear risk	Not reported.
Allocation concealment (selection bias)	Unclear risk	Not reported.
Blinding of participants and personnel (performance bias) All outcomes	High risk	Not reported but different treatments (including elimination diet) administered.
Blinding of outcome assessment (detection bias) All outcomes	High risk	Not reported but different treatments (including elimination diet) administered.
Incomplete outcome data (attrition bias) All outcomes	Unclear risk	64 patients randomized and 50 patients included (dropouts for the entire study, no report per treatment arm).
Selective reporting (reporting bias)	Unclear risk	Only histologic response rate reported.
Other bias	Unclear risk	No possible assessment of baseline imbalance.

**Table:** Peterson 2010

***Study characteristics***		
Methods	**RCT design and number of study arms:** RCT **Single‐center or multi‐center:** single‐center, University of Utah Health Sciences Center **Countries:** USA **Study dates**: NR	
Participants	**Active EoE or inactive EoE at beginning of study:** active **EoE definition/diagnostic criteria: ≥** 15 eosinophils averaged over 5 high‐power fields on esophageal biopsy in participants with symptoms of dysphagia, food impaction or chest pain **Inclusion criteria:** Patients aged 18 to 80 with eosinophilic esophagitis, are defined as a) dysphagia, food impaction or other upper gastrointestinal symptoms (chest pain, heartburn, regurgitation); b) multiple esophageal rings or furrows; c) the presence of > 20 eosinophils/high‐power field in the squamous epithelium or deeper tissues of the esophagus Ability to undergo esophageal manometry and ambulatory pH monitoring No history of bleeding diathesis, significant cardiopulmonary disease, or other contraindication to upper endoscopy Those who have had a 1‐month holiday from either esomeprazole therapy or fluticasone if they have been prescribed this prior to enrollment **Exclusion criteria:** Patients were excluded if they had to be dilated on the first exam (due to the inability to pass an upper endoscope) Had a history of a contraindication or intolerance of either fluticasone or PPIs Were pregnant or incarcerated Had a history of prior upper gastrointestinal surgery Had other potential alternative mechanisms for dysphagia demonstrated at endoscopy Current use of PPIs or glucocorticoids Symptoms of food impaction to food bolus size < 1 cm **Age at beginning of study per study group:** esomeprazole = 38.8 years (26 to 79), fluticasone = 34.6 years (18 to 58) **Sex (m/f) per study group (numbers of patients):** 12/M 3/F, 11/M 4/F **Number randomized per study group:** esomeprazole (n = 15), fluticasone (n = 15) **Number reaching end of study per study group:** NR (6 overall)	
Interventions	**Study group 1 (control or placebo):** esomeprazole 40 mg a day for 8 weeks **Study group 2:** swallowed aerosolized fluticasone (440 µg twice‐daily)	
Outcomes	**Primary outcomes of the study: "**We anticipated an 80% response rate (defined as a decrease in dysphagia score of at least two points) in the fluticasone arm as compared to a 33% response rate in the esomeprazole arm as the primary endpoint". **Secondary outcomes of the study:** Secondary endpoints included changes in eosinophilic infiltration in esophageal biopsies. "We arbitrarily defined partial resolution as B15 eos/HPF and complete resolution as B5 eos/HPF".	
Notes	**Funding source:** funding support was provided in part by an ASGE Research grant **Conflicts of interest:** NR	
***Risk of bias***		
**Bias**	**Authors' judgement**	**Support for judgement**
Random sequence generation (selection bias)	Low risk	Computer‐generated randomization method.
Allocation concealment (selection bias)	Low risk	The research staff assigned (central allocation) the participants to the treatment group after the investigator who performed the endoscopy enrolled the participant.
Blinding of participants and personnel (performance bias) All outcomes	High risk	Neither the participants nor the investigators were blinded to treatment.
Blinding of outcome assessment (detection bias) All outcomes	High risk	The pathologist who evaluated histology was blinded, however no other outcome assessment was blinded.
Incomplete outcome data (attrition bias) All outcomes	Low risk	There was low attrition that was explained by the personnel.
Selective reporting (reporting bias)	High risk	The authors swapped the primary and secondary outcomes between the protocol and manuscript.
Other bias	Low risk	No major baseline differences between groups. No other concerns.

**Table:** Rothenberg 2015

***Study characteristics***		
Methods	**RCT design and number of study arms:** RCT with 2 arms **Single‐center or multi‐center:** multi‐center **Countries:** United States **Study dates:** December 2009 to February 2012	
Participants	**Active EoE or inactive EoE at beginning of study:** active **EoE definition/diagnostic criteria:** esophageal biopsy must show ≥ 24 eos per HPF (400x) in the proximal or distal esophagus **Inclusion criteria:** Male and female participants with symptomatic eosinophilic esophagitis aged 18 to 50 years. Female participants were required to be surgically sterilized at least 6 months before study participation or postmenopausal (no regular bleeding for at least 2 years) confirmed by a plasma FSH level of > 40 IU/L at screening or baseline. Esophageal biopsy must show ≥ 24 eos per HPF (400x) in the proximal or distal esophagus, validated by a central laboratory pathologist. Elimination diet must either: not be indicated following allergy evaluation including skin prick testing with multiple food antigens, or have been refused to be followed by participants, or have undergone a minimum of 3 months of elimination diet as indicated by skin prick testing without detectable resolution by repeat endoscopy with biopsies, or participant refusal to follow elimination diet. Participants treated with a proton pump inhibitor (PPI) must have been on treatment for at least 2 months before enrollment. The PPI must be used before endoscopy to rule out the possibility of GERD in the proximal or distal esophagus. Failure of histological improvement is defined as eosinophil density ≥ 24 per HPF (400x) after 2 months treatment with a PPI or documented by prior endoscopy or lack of complete disappearance of symptoms. Participants had to be able to communicate well with the investigator, to understand and comply with the requirements of the study and also to understand and sign the written informed consent. **Exclusion criteria:** Participants have received systemic corticosteroid therapy, by any route of administration, within 3 months before dosing or have received topical corticosteroids (swallowed aerosolized fluticasone or budesonide) within 2 months before dosing. Comorbid eosinophilic disorders (other than atopic dermatitis not requiring chronic steroid therapy). History of exposure to human therapeutic antibody, immunoglobulin, or other plasma product within 6 months of dosing (e.g. Xolair). History of clinical schistosomiasis, or stool examination positive for ova or parasites or travel within the preceding 6 months to an area with endemic schistosomiasis, including but not limited to Southeast and Southwest Asia, South America, and Africa. Travel to these areas was not to be planned for at least 6 months after the last dose. Participants who have not had a trial of PPI or prior allergy testing/elimination diet and who did not fulfill entry criteria after PPI or initiation of elimination diet therapy. Participation in any clinical intervention with any drug administration within 4 weeks before initial dosing or longer if required by local regulations, and for any other limitation of participation based on local regulations. Donation or loss of 400 mL or more of blood within 8 weeks before initial dosing, or longer if required by local regulation. A past medical history of clinically significant ECG abnormalities. An abnormal ECG is defined as PR > 220 ms, QRS complex > 120 ms, QTcB > 430 ms, or any significant morphological changes, other than non‐specific T‐wave changes. Any surgical or medical condition that might significantly alter the absorption, distribution, metabolism, or excretion of drugs, or which may jeopardize the participant in case of participation in the study. The investigator was to make this determination in consideration of the participant’s medical history and/or clinical or laboratory evidence of any of the following: major gastrointestinal tract surgery such as gastrectomy, gastroenterostomy, or bowel resection. History of immunodeficiency diseases, including a positive HIV (ELISA and Western blot) test result. Positive hepatitis B surface antigen (HBsAg) or hepatitis C test result. History of drug or alcohol abuse within the 12 months before dosing, or evidence of such abuse as indicated by the laboratory assays conducted during the screening. Participants that took acetaminophen (paracetamol) chronically, that is, more than 1 g/day for more than 3 out of 7 days, or more than 2 g/day for more than 1 out of 7 days (added as part of Amendment 5). **Age at beginning of study per study group:** QAX576 (dectrekumab), 6 mg/kg: mean (SD), 30.7 (9.58) Placebo: mean (SD), 29.5 (11.22) **Sex (m/f) per study group:** QAX576 (dectrekumab), 6 mg/kg: m/f n, 16/1 Placebo: m/f n, 8/0 **Number randomized per study group:** QAX576 (dectrekumab), 6 mg/kg: 17 Placebo: 8 **Number reaching end of study per study group:** QAX576 (dectrekumab), 6 mg/kg: 13 Placebo: 5	
Interventions	**Study group 1:** placebo **Study group 2:** QAX576 (dectrekumab), 6 mg/kg	
Outcomes	**Primary outcomes of the study:** The primary endpoint in this study is the number of patients (responder) with a reduction of 75% or more in eosinophils per HPF (distal or proximal esophagus) from baseline to week 13 **Secondary outcomes of the study:** Efficacy: symptoms. Mayo Dysphagia Questionnaire (MDQ) with a 2‐week recall period (Grudell AB, Alexander JA, Enders FB, Pacifico R, Fredericksen M, Wise JL, et al. Validation of the Mayo Dysphagia Questionnaire. Dis Esophagus 2007;20:202‐5). Efficacy: molecular. The EoE transcriptome before and after study medication (QAX576 and placebo) was compared by using the NanoString nCounter technology (NanoString Technologies, Seattle, Wash) for direct multiplexed measurement of gene expression. Circulating protein biomarkers and histochemistry. Quantitative determination of periostin was performed by means of liquid chromatography mass spectrometry. Histochemical staining for collagen, periostin, and eotaxin was performed.	
Notes	**Funding source:** Novartis Pharmaceuticals **Conflicts of interest:** M. E. Rothenberg has received consultancy fees from Immune Pharmaceuticals, Receptos, Pluristem Pharmaceuticals, Regeneron, and Novartis; has an equity interest in Immune Pharmaceuticals and Receptos and can receive royalty fees from Teva for reslizumab, which is under development; and is a coinventor on patent applications owned by Cincinnati Children’s, concerning the eosinophilic esophagitis transcriptome. T. Wen is a coinventor of a patent application, owned by Cincinnati Children’s Hospital, concerning the eosinophilic esophagitis transcriptome. B. Enav reports personal fees from QOL Medical outside the submitted work. I. Hirano reports personal fees from Novartis, Meritage, Aptalis, and Receptos outside the submitted work. S. Kaiser, T. Peters, I. Jones, J. P. Arm, and K. A. Gunawardena are employees of Novartis. R. Strieter is the Global Head for Translational Medicine for Respiratory disorders at Novartis Institutes of Biomedical Research and has stock equity in Novartis. R. Sabo and A. Perez are consultants for Novartis. The rest of the authors declare that they have no relevant conflicts of interest. Paper states "Sample management, sample analysis, and technical assistance were provided by Martin Letzkus, Urs Affentranger, Aurelie Seguin, Tiziana Valensise, Stephan Bek, Junli Yu, and Shenglin Ma of the Biomarker Development Group of Novartis Institutes for Biomedical Research. support was provided by Saurabh Aggarwal, Senior Scientific Writer, Medical Communications, Novartis", suggesting that author and editorial input came from an interested pharmaceutical company they were unable to provide how much input they had.	
***Risk of bias***		
**Bias**	**Authors' judgement**	**Support for judgement**
Random sequence generation (selection bias)	Low risk	The authors responded that "this study was sponsored by Novartis and we received a randomization ID via email. Presumably the allocations were generated using a random sequence generator". On balance, we have judged this likely to be random.
Allocation concealment (selection bias)	Low risk	The randomization was performed by using a "Request & Response" exchange system by an email between the study site’s unblinded pharmacist and Novartis.
Blinding of participants and personnel (performance bias) All outcomes	Low risk	Patients, investigators, and study personnel were blinded to treatment assignment.
Blinding of outcome assessment (detection bias) All outcomes	Low risk	A blinded central pathologist assessed the primary outcome. Blinded personnel assessed clinical symptoms. Molecular outcomes were analyzed by the Biomarker Development group at Novartis.
Incomplete outcome data (attrition bias) All outcomes	Low risk	Minimal and explained attrition that likely has no effect on outcome.
Selective reporting (reporting bias)	Low risk	Protocol is available and all pre‐specified outcomes of interest were reported in the pre‐specified way.
Other bias	Low risk	No major baseline differences between groups. No other concerns.

**Table:** Rothenberg 2022

***Study characteristics***		
Methods	**RCT design and number of study arms:** part B of a 3‐part, randomized, placebo‐controlled phase 3 study, 2 arms **Single‐center or multi‐center:** multi‐center; 95 study locations worldwide **Countries:** US, Australia, Belgium, Canada, France, Germany, Italy, Netherlands, Spain, Sweden, Switzerland, and the UK **Study dates:** 24 September 2018 to 9 September 2021	
Participants	**Active EoE or inactive EoE at beginning of study:** active **EoE definition/diagnostic criteria:** "A documented diagnosis of EoE by endoscopic biopsy." **Inclusion criteria:** A documented diagnosis of EoE by endoscopic biopsy Baseline endoscopic biopsies with a demonstration on central reading of intraepithelial eosinophilic infiltration History (by patient report) of an average of at least 2 episodes of dysphagia (with intake of solids) per week in the 4 weeks prior to screening **Exclusion criteria:** Body weight ≤ 40 kg Prior participation in a dupilumab clinical trial, or past or current treatment with dupilumab Initiation or change of a food‐elimination diet regimen or re‐introduction of a previously eliminated food group in the 6 weeks prior to screening Other causes of esophageal eosinophilia or the following conditions: hypereosinophilic syndrome and eosinophilic granulomatosis with polyangiitis (Churg‐Strauss syndrome) Active *Helicobacter pylori* infection History of achalasia, Crohn's disease, ulcerative colitis, celiac disease, and prior esophageal surgery Any esophageal stricture unable to be passed with a standard, diagnostic, 9 mm to 10 mm upper endoscope or any critical esophageal stricture that requires dilation at screening History of bleeding disorders or esophageal varices Pregnant or breastfeeding women, or women planning to become pregnant or breastfeed during the study **Age at beginning of study per study group:** NR, "adolescents and adults" **Sex (m/f) per study group:** NR **Number randomized per study group:** Dupilumab: 80 Placebo: 79 **Number reaching end of study per study group:** NR	
Interventions	**Study group 1:** placebo, type not specified **Study group 2:** dupilumab 300 mg weekly	
Outcomes	**Primary outcomes of the study:** Proportion of patients achieving a peak esophageal intraepithelial eosinophil (eos) count of < 6 eos/hpf Absolute change from baseline in Dysphagia Symptom Questionnaire (DSQ) score **Secondary outcomes of the study:** Safety	
Notes	**Funding source:** Research sponsored by Sanofi and Regeneron Pharmaceuticals, Inc. **Conflicts of interest:** Dr. Dellon ‐ Consultant: Abbott, Adare, Aimmune, Allakos, Amgen, Arena, AstraZeneca, Biorasi, Calypso, Eli Lilly, EsoCap, Gossamer Bio,GlaxoSmithKline, Parexel, Receptos/Celgene/Bristol Myers Squibb, Regeneron Pharmaceuticals, Inc., Robarts, Salix, Shire/Takeda; research funding: Adare, Allakos, GlaxoSmithKline, Meritage, Miraca, Nutricia, Receptos/Celegene/Bristol Myers Squibb, Regeneron Pharmaceuticals, Inc., Shire/Takeda; educational grant: Allakos, Banner, Holoclara. Dr. Rothenberg ‐ Consultant: Allakos, AstraZeneca, Bristol Myers Squibb, ClostraBio, Pulm One, Spoon Guru; equity interest: ClostraBio, Pulm One,Spoon Guru; royalties from reslizumab: Teva Pharmaceuticals; royalties from PEESSv2: Mapi Research Trust; royalties: UpToDate; inventor of patents owned by Cincinnati Children’s Hospital. Dr. Collins ‐ consultant: Allakos, Arena, AstraZeneca, Bristol Myers Squibb, Calypso, Esocap, GlaxoSmithKline, Regeneron Pharmaceuticals, Inc., Shire; research funding: Receptos/Bristol Myers Squibb, Regeneron Pharmaceuticals, Inc., Shire. Dr. Hirano ‐ consultant: Adare, Receptos/Bristol Myers Squibb, Regeneron Pharmaceuticals, Inc., Shire; research funding: Meritage,Receptos/Bristol Myers Squibb, Regeneron Pharmaceuticals, Inc., Shire. Dr. Chehade ‐ consultant: Adare, Allakos, Astra Zeneca, Nutricia, Regeneron Pharmaceuticals, Inc., Shire; research funding: Allakos, RegeneronPharmaceuticals Inc., Shire; honoraria for lectures: Medscape, Nutricia. Dr. Bredenoord – consultant: Arena, AstraZeneca, Calypso, EsoCap, Falk, Gossamer Bio, Medtronic, Laborie, RB, Regeneron, Robarts; research funding: Bayer, Nutricia, SST; equity interest: SST. Dr. Lucendo – Consultant: EsoCap, Dr. Falk Pharma; Research funding: Dr. Falk Pharma, Regeneron Pharmaceuticals. Dr. Spergel – Consultant: Regeneron, Shire, Takeda, Allakos, DBV Technology, Novartis; Grant Support: Regeneron, DBV Technology. Q Zhao, JD Hamilton, B Beazley, S Kamat, M Ruddy, B Akinlade, N Amin, A Radin, B Shumel, J Maloney: Regeneron Pharmaceuticals, Inc. –Employees and Shareholders. I Guillemin: Sanofi – Prior employee, may hold stock and/or stock options in the company. L Mannent, E Laws: Sanofi – Employees, may hold stock and/or stock options in the company.	
***Risk of bias***		
**Bias**	**Authors' judgement**	**Support for judgement**
Random sequence generation (selection bias)	Low risk	The author confirmed the randomization was computer‐generated.
Allocation concealment (selection bias)	Low risk	The author confirmed that central allocation was completed by the sponsor.
Blinding of participants and personnel (performance bias) All outcomes	Low risk	The author confirmed the study was appropriately blinded.
Blinding of outcome assessment (detection bias) All outcomes	Low risk	The author confirmed the pathologist and endoscopist were blinded.
Incomplete outcome data (attrition bias) All outcomes	Low risk	No patients were lost to follow‐up. Imputation was used for continuous outcomes.
Selective reporting (reporting bias)	Unclear risk	Not all of the many registered outcomes have been reported as per the trial registration in this abstract publication.
Other bias	Low risk	No major baseline differences between groups. No other concerns.

**Table:** Schaefer 2008

***Study characteristics***		
Methods	**RCT design and number of study arms:** RCT with 2 arms **Single‐center or multi‐center:** single‐center **Countries:** United States **Study dates:** February 2000 to November 2004	
Participants	**Active EoE or inactive EoE at beginning of study:** active **EoE definition/diagnostic criteria:** esophageal mucosal biopsy specimens showing ≥ 15 eos/hpf with negative pH probe studies. **Inclusion criteria:** Age range 1 to 18 years Active eosinophilic esophagitis with ≥ 15 eosinophils per hpf and negative pH probe study **Exclusion criteria:** Co‐existing esophageal conditions (e.g. stricture, Barrett’s esophagus, caustic injury), *Helicobacter pylori* infection, inflammatory bowel disease, and inability to tolerate corticosteroids **Age at beginning of study per study group:** Prednisone: mean (SD), 7.0 (4.3) Fluticasone: mean (SD), 7.2 (4.1) **Sex (m/f) per study group:** Prednisone: m/f, 31/9 Fluticasone: m/f, 28/12 **Number randomized per study group:** Prednisone: 40 Fluticasone: 40 **Number reaching end of study per study group:** Prednisone: 32 through follow‐up EGD after 4 weeks of full strength therapy; 27 through 8‐week wean; 25 through week 18 follow‐up; 17 through week 24 follow‐up Fluticasone: 36 through follow‐up EGD after 4 weeks of full strength therapy; 27 through 8‐week wean; 22 through week 18 follow‐up; 19 through week 24 follow‐up	
Interventions	**Study group 1:** Prednisone: oral prednisone suspension/tablet (1 mg/kg/dose twice a day; maximum 30 mg twice a day) **Study group 2:** Fluticasone: swallowed fluticasone by metered dose inhaler (110 g per puff for ages 1 to 10 years and 220 g per puff for ages 11 years or older, 2 puffs 4 times/day)	
Outcomes	**Primary outcomes of the study:** A histologic response by an improvement in biopsy grade after 4 weeks of corticosteroid therapy. Points were assigned based on (1) basal cell zone thickness as a percentage of the epithelial thickness, and (2) the maximum number of eos/hpf (at 400 power using an eyepiece grid covering an area of 0.4 mm^2^). Points were summed and the totals were translated into histologic grades (normal, mild, moderate, and severe). Grades were assigned a numeric value for statistical analysis. **Secondary outcomes of the study:** Clinical response to corticosteroids based on the presence or absence of the presenting symptom by patient/guardian report and by physician assessment at predetermined intervals. Reported symptoms included vomiting, abdominal pain, epigastric pain, heartburn, dysphagia, feeding problems, foreign body/food impaction, and weight loss.	
Notes	**Funding source:** Clarian Values Grant, Clarian Health Partners, Inc, Indianapolis, IN **Conflicts of interest:** NR	
***Risk of bias***		
**Bias**	**Authors' judgement**	**Support for judgement**
Random sequence generation (selection bias)	Low risk	Random number assignment was used to generate a concealed allocation schedule that was maintained by a research co‐ordinator.
Allocation concealment (selection bias)	Low risk	Concealed allocation schedule that was maintained by a research co‐ordinator.
Blinding of participants and personnel (performance bias) All outcomes	High risk	This was an open‐label trial.
Blinding of outcome assessment (detection bias) All outcomes	Low risk	The authors confirmed in November 2022 that the outcome assessors were all blinded.
Incomplete outcome data (attrition bias) All outcomes	Low risk	No significant imbalance in withdrawals, which were all explained.
Selective reporting (reporting bias)	Low risk	Protocol is available and all pre‐specified outcomes of interest were reported in the pre‐specified way.
Other bias	Low risk	No major baseline differences between groups. No other concerns.

**Table:** Spergel 2012

***Study characteristics***		
Methods	**RCT design and number of study arms:** RCT, 4 arms **Single‐center or multi‐center:** multi‐center, 34 sites in the United States and 2 sites in Canada **Countries:** USA and Canada	
Participants	**Active EoE or inactive EoE at beginning of study:** active **EoE definition/diagnostic criteria:** defined as ≥ 24 eosinophils in ≥ 1 high‐power field (hpf)) **Inclusion criteria:** At least 1 active symptom (i.e. vomiting, regurgitation, abdominal pain, chest pain/heartburn, or dysphagia) of moderate severity or worse (as assessed by the patient) within the week before randomization An OGD with biopsy documenting active eosinophilic esophagitis (defined as > 24 eosinophils in > 1 high‐power field (hpf)) A history of eosinophilic esophagitis symptoms, and treatment with proton pump inhibitors with or without histamine H2 receptor antagonists for at least 4 weeks without symptom resolution or a normal pH probe (regardless of whether the patient had undergone a failed course of proton pump inhibitors) **Exclusion criteria:** If they had another disorder that could cause esophageal eosinophilia (e.g. hypereosinophilic syndrome, Churg‐Strauss vasculitis, eosinophilic gastroenteritis, or parasitic infection) Had a history of abnormal gastric or duodenal biopsy results or documented gastrointestinal disorders (e.g. celiac disease, Crohn disease, recurrent acute or chronic diarrhea, ulcerative colitis, malrotation, or active *Helicobacter pylori* infection) Had a history of gastrointestinal procedures (i.e. esophageal surgery (not including esophageal dilation), fundoplication, gastric surgery, or surgery for intestinal atresia) Had used systemic immunosuppressive or immunomodulating agents (e.g. oral corticosteroids, antibodies to IgE, methotrexate, cyclosporine, IFN‐a, or TNF‐a inhibitors) within 6 months before study entry Had received live attenuated vaccines within 3 months before study entry Had swallowed corticosteroids formulated for inhalation for the treatment of eosinophilic esophagitis within 1 month before study entry Had a stricture that would have prevented the passage of the endoscope Had an infection that might have interfered with study assessments; or had a concurrent immunodeficiency Patients were also excluded if they had previously participated in any investigational drug or device study within 30 days before study entry or any investigational study of a biologic therapy within 3 months before study entry or received mepolizumab within 4 months or reslizumab any time before study entry **Age at beginning of study per study group:** 1 mg/kg reslizumab: 12.3 (3.83) 2 mg/kg reslizumab: 11.8 (3.82) 3 mg/kg reslizumab: 11.5 (4.04) Placebo group: 11.9 (4.17) **Sex (m/f) per study group:** NR **Number randomized per study group:** 1 mg/kg reslizumab = 56 2 mg/kg reslizumab = 57 3 mg/kg reslizumab = 57 Placebo = 57 **Number reaching end of study per study group:** 1 mg/kg reslizumab = 48 2 mg/kg reslizumab = 47 3 mg/kg reslizumab = 50 Placebo = 51	
Interventions	**Placebo group:** placebo/saline infusion only **Intervention group 2, 3, 4:** Intervention group ‐ arm 1: 1 mg/kg reslizumab + saline infusion Intervention group ‐ arm 2: 2 mg/kg reslizumab + saline infusion Intervention group ‐ arm 3: 3 mg/kg reslizumab + saline infusion	
Outcomes	**Primary outcomes of the study:** Co‐primary efficacy measures were the percentage change from baseline to the end of therapy (or early withdrawal) in the peak esophageal eosinophil count The change from baseline to the end of therapy (or early withdrawal) in the physician’s eosinophilic esophagitis global assessment score **Secondary outcomes of the study:** no secondary outcome in FT ‐ secondary outcome in protocol as follows: Mean change from baseline to end of treatment in eosinophilic esophagitis predominant symptom assessment (time frame: baseline (day 1, pre‐treatment), end of treatment (week 15, 3 weeks (± 4 days) after the last dose of study drug, or at early withdrawal)) Mean percent change from baseline to end of treatment in the Child Health Questionnaire (CHQ) (time frame: baseline, end of treatment (up to 15 weeks ± 4 days))	
Notes	**Funding**: Sponsored by Ception Therapeutics, Inc, which has since been acquired by Cephalon, Inc. **Conflict of interest**: Sponsored by Ception Therapeutics, Inc, which has since been acquired by Cephalon, Inc. Disclosure of potential conflict of interest: J. M. Spergel is a consultant for DBV; has received research support from the Department of Defense (DOD), Cephalon, and the National Institutes of Health (NIH); is a member of the American Academy of Allergy, Asthma & Immunology; and is on the American Partnership for Eosinophilic Disorders (APFED) Medical Advisory Board. M. E. Rothenberg has equity interest in reslizumab through Cephalon; is consultant and chief scientific officer of Immune Pharmaceuticals; has received research support from the NIH, the Food Allergy & Anaphylaxis Network, and the DOD; is on the APFED Medical Advisory Board; and is on the International Eosinophil Society Executive Council. M. H. Collins is a central review pathologist for Cephalon, GlaxoSmithKline, and Meritage Pharma; is a consultant for Sunovion; and is president of the APFED Medical Advisory Board. G. T. Furuta is a consultant for Nutricia and Meritage and has received research support from the NIH, AstraZeneca, and the Thrasher Foundation. G. Fuchs III has received research support from Shire and Cephalon. J. P. Abonia has received research support from the NIH, Ception Therapeutics, and the Children’s Digestive Health and Nutrition Foundation. T. Henkel is a consultant for Cephalon and a shareholder in Ception Therapeutics. C. A. Liacouras is a speaker for Nutricia and is on the American Partnership for Eosinophilic Disorders Physician Board. The rest of the authors declare that they have no relevant conflicts of interest. Received for publication 21 September 2011.	
***Risk of bias***		
**Bias**	**Authors' judgement**	**Support for judgement**
Random sequence generation (selection bias)	Low risk	Computer‐generated randomization method.
Allocation concealment (selection bias)	Low risk	Central allocation was confirmed by the author.
Blinding of participants and personnel (performance bias) All outcomes	Low risk	Patients, investigators, and study personnel were blinded to treatment assignment throughout the study. The study site’s pharmacist was unblinded and was responsible for preparing and dispensing study medication.
Blinding of outcome assessment (detection bias) All outcomes	Low risk	All staff were blinded except the study site’s pharmacist was unblinded and was responsible for preparing and dispensing study medication.
Incomplete outcome data (attrition bias) All outcomes	Low risk	1 patient was discontinued due to an adverse event. The adverse event was not reported in the paper. The author responded that "for the one discontinuation due to mild abdominal pain and upper respiratory tract congestion. It was thought be unrelated to study medication". We thought this was unlikely to have influenced our outcomes of interest.
Selective reporting (reporting bias)	Low risk	Trial protocol is available and all pre‐specified outcomes of interest were reported in the pre‐specified way.
Other bias	Low risk	No major baseline differences between groups. No information on participant sex.

**Table:** Spergel 2020

***Study characteristics***		
Methods	**RCT design and number of study arms:** parallel, double‐blind RCT, 2 arms **Single‐center or multi‐center:** single‐center **Countries:** USA **Study dates:** screening 11 November 2015 to 20 December 2016. After enrollment, 9 months of treatment (milk out of the diet) and then 2 months with milk in the diet before another scope. After that scope 11 months open‐label extension.	
Participants	**Active EoE or inactive EoE at beginning of study:** inactive EoE at beginning of study. Patients had to have < 10 eos/hpf after milk‐free diet for 2 months to be eligible to participate. **EoE definition/diagnostic criteria:** the diagnosis of EoE was confirmed with an esophagogastroduodenoscopy (EGD) and biopsy showing 15 eos/HPF after at least a 2‐month period of high‐dose proton pump inhibitor (PPI) (1 to 2 mg/kg dose twice‐daily) **Inclusion criteria:** EGD on a milk‐containing diet showed 15 eos/HPF EGD with biopsy on milk‐free diet for 2 months showed < 10 eos/HPF Age 4 to 17 years **Exclusion criteria:** IgE‐mediated milk allergy (positive skin test or specific IgE to milk using standard techniques) Were pregnant or breastfeeding Were currently taking systemic corticosteroids, tricyclic antidepressants, beta‐blockers, or other medications for EoE outside of a PPI Patients with a history consistent with poorly controlled persistent asthma; comorbid cardiac, autoimmune, infectious gastrointestinal (GI) or pulmonary conditions; or diagnosis of eosinophilic colitis or gastritis **Age at beginning of study per study group:** For patients enrolled in the study there were 5 in the placebo group (median age 12.54 (range 9.55 to 14.55)) and 15 in the treatment group (median age 10.83 (range 5.86 to 15.37)) **Sex (m/f) per study group:** Study group 1 (placebo) included 5 males and 0 females and Study group 2 (treatment) included 10 males and 5 females **Number randomized per study group:** N = 5 for Study group 1 (placebo) and N = 15 for Study group 2 (treatment) **Number reaching end of study per study group:** 2 reached the end of the study (were analyzed) for Study group 1 and 7 reached the end of the study for Study group 2	
Interventions	**Study group 1:** Viaskin placebo participants epicutaneously administered daily (up to 24 hours application per day) with a patch containing a matching placebo formulation **Study group 2:** Viaskin milk 500 µg participants epicutaneously administered daily (up to 24 hours application per day) with a patch containing 500 µg cow's milk proteins For both groups the same dose of PPI was continued (as well as the same dose of medications for asthma and allergic rhinitis)	
Outcomes	**Primary outcomes of the study:** the primary efficacy endpoint is each patient’s maximum esophageal eosinophil count on all specimens obtained from the biopsy at the end of double‐blind treatment, after milk reintroduction **Secondary outcomes of the study:** 3 different symptom assessments (PEESS‐parent, PEESS‐patients and investigator assessment of symptoms; EREFS score	
Notes	**Funding source:** DBV technologies and the Children's Hospital of Philadelphia Eosinophilic Esophagitis Family Fund **Conflicts of interest:** consulting agreements and clinical trial grants with DBV Technologies and the first author has stock equity with DBV Technologies	
***Risk of bias***		
**Bias**	**Authors' judgement**	**Support for judgement**
Random sequence generation (selection bias)	Low risk	"The randomization list and treatment allocation were done and computer generated by an independent party, eXYSTAT (Paris, France), and they had no other role in the study."
Allocation concealment (selection bias)	Low risk	"The randomization list and treatment allocation were done and computer generated by an independent party, eXYSTAT (Paris, France), and they had no other role in the study."
Blinding of participants and personnel (performance bias) All outcomes	Low risk	"Individuals providing care, assessing outcomes, and participants were masked to group assignment." "Masking was done with identical‐looking Viaskin patches"
Blinding of outcome assessment (detection bias) All outcomes	Low risk	"Individuals providing care, assessing outcomes, and participants were masked to group assignment." "Masking was done with identical‐looking Viaskin patches"
Incomplete outcome data (attrition bias) All outcomes	Low risk	No attrition imbalances, or imbalances in reasons for attrition, based on the flow diagram of the study.
Selective reporting (reporting bias)	Low risk	Outcomes reported as per published protocol.
Other bias	Low risk	Low imbalance. No female patients in the placebo group compared to 5 (33%) of the Viaskin milk group.

**Table:** Straumann 2010a

***Study characteristics***		
Methods	**RCT design and a number of study arms:** double‐blind RCT; 2 arms **Single‐center or multi‐center:** single‐center (Olten, Switzerland) **Countries:** Switzerland **Dates:** December 2005 and May 2006	
Participants	**Active EoE or inactive EoE at beginning of the study:** active **EoE definition/diagnostic criteria:** EoE with a history of at least one episode of dysphagia per week in the 4 weeks prior to the start of study medication and a peak esophageal eosinophilia of > 20 eosinophils per hpf (peak eosinophil density) **Inclusion criteria:** Document evidence/presence of esophagitis prior to commencing trial drug Histological evidence of esophagitis: greater than 20x eosinophils per high‐power field (x400) on histology of esophageal mucosal biopsy At least one episode of dysphagia per week Inadequate response to routine eosinophilic esophagitis treatment (topical or systemic steroids) No other known causes of esophagitis, or esophageal or generalized eosinophilia Not pregnant or nursing **Exclusion criteria:** History of seasonal worsening of eosinophilic esophagitis symptoms or requirement of esophageal dilation Churg‐Strauss syndrome Wegener's granulomatosis Lymphoma, hematological malignancy, advanced and metastatic solid tumors Active *H. pylori* infection Any previous treatment with anti‐hIL‐5, anti‐IgE monoclonal antibody, or other biological agents Other causes of esophagitis (hypereosinophilic syndromes, eosinophilic gastroenteritis, and parasitic infection). GERD was excluded in all patients by pretreatment with PPIs in standard dosages plus negative endoscopy for signs of reflux disease, and by pH monitoring (optional) Any condition with the risk of requiring esophageal dilatation during the course of the study A history of seasonal exacerbation of EoE symptoms is expected to coincide with the period of investigation Active *Helicobacter pylori* infection and any unstable medical conditions Patients using mast cell stabilizers, leukotriene receptor antagonists, or immunosuppressive/immunomodulatory agents, and those with a history of allergic reactions to previous antibody treatment Any previous treatment with an anti‐IL‐5 antibody or any other biopharmaceutical agent Female patients were excluded if pregnant or breastfeeding, or if they were not taking adequate contraceptive measures **Age at beginning of study per study group:** intervention = 32.4; placebo = 34.0 (mean, no SD reported) **Sex (m/f) per study group:** M/F = 4/1; M/F = 3/3 **Number randomized per study group:** intervention = 5; placebo n = 6 **Number reaching the end of study per study group:** intervention = 5; placebo n = 6	
Interventions	**Study group 1:** placebo **Study group 2:** mepolizumab (GlaxoSmithKline, Greenford, UK) was administered by intravenous infusion at a dose of 750 mg diluted in 150 mL of 0.9% sodium chloride solution 2 doses day 0 and day 7	
Outcomes	**Primary outcomes of the study:** to reduce peak esophageal eosinophilia to ≤ 5 eos/hpf **Secondary outcomes of the study:** effect of treatment on symptoms, eosinophil levels, and inflammation biomarkers in esophagus tissue and blood	
Notes	**Funding**: This study was supported by GlaxoSmithKline (GSK), Greenford, UK. The trial was conducted under GSK protocol number MEE103226. **Conflicts of interest:** declared here.	
***Risk of bias***		
**Bias**	**Authors' judgement**	**Support for judgement**
Random sequence generation (selection bias)	Low risk	Computer‐generated randomization method.
Allocation concealment (selection bias)	Low risk	Central allocation via a telephonic randomization system. "To keep the treatment blinded, the infusions were made up by an independent pharmacist who obtained the treatment allocation via a telephonic randomisation system"
Blinding of participants and personnel (performance bias) All outcomes	Low risk	"Of the study personnel, only the pharmacist, responsible for the preparation of infusions, had access to the treatment assignments." "Patients allocated to the placebo arm received the corresponding infusions of saline only. To keep the treatment blinded, the infusions were made up by an independent pharmacist".
Blinding of outcome assessment (detection bias) All outcomes	Low risk	"Of the study personnel, only the pharmacist, responsible for the preparation of infusions, had access to the treatment assignments".
Incomplete outcome data (attrition bias) All outcomes	Low risk	There were no dropouts in any group.
Selective reporting (reporting bias)	Low risk	Outcomes were pre‐specified and were reported in the pre‐specified way.
Other bias	Low risk	No major baseline differences between groups. No other concerns.

**Table:** Straumann 2010b

***Study characteristics***		
Methods	**RCT design:** randomized, placebo‐controlled, parallel‐group clinical trial **Single‐center or multi‐center:** single‐center **Countries:** Switzerland **Study dates:** December 2005 to December 2008	
Participants	**Active EoE or inactive EoE:** active eosinophilic esophagitis **EoE definition/diagnostic criteria:** clinicopathologic definition of esophageal symptoms in combination with a dense esophageal eosinophilia, both being refractory to proton pump inhibition **Inclusion criteria:** Patients older than 14 years old Isolated esophageal eosinophilia Dysphagia almost always occurring with intake of solids when off anti‐inflammatory therapy or dietary restriction Eosinophilic tissue infiltration (mean cell density of ≥ 20 eosinophils per high‐power field) on esophageal histology biopsy specimens Exclusion of other causes of esophageal or systemic eosinophilia Exclusion of gastroesophageal reflux disease by proton pump inhibitor pretreatment plus negative endoscopy for signs of reflux disease and pH monitoring (optional) **Exclusion criteria:** Current use of specific treatments for eosinophilic esophagitis Secondary causes of esophageal eosinophilia Intolerance to budesonide Concomitant therapies for any reason that may affect the assessment Use of an investigational drug within 30 days of entering the study Recent history of suspicion of current drug abuse and alcohol abuse A positive serum pregnancy test at the screening visit Any unstable serious co‐existing medical condition **Age:** Budesonide – mean (SD): 33.1 ± 13.1 Placebo – mean (SD): 38.2 ± 12.4 **Sex:** Budesonide – 18 total patients: (m/f): 17/1 Placebo – 18 total patients: (m/f): 14/4 **Number randomized:** Budesonide n = 18 Placebo n = 18 **Number reaching the end:** Budesonide: 18/18 (100%) Placebo: 18/18 (100%)	
Interventions	**Study group 1: placebo** 0.9% saline Administered via nebulizer 4 mL twice per day at bedtime and in the morning after breakfast Administered into the oropharynx, patient instructed to swallow continuously the accumulated liquid Nothing to eat or drink for 30 minutes after administration **Study group 2: budesonide suspension 2.0 mg** 0.25 mg/mL suspension Administered via nebulizer 4 mL twice per day at bedtime and in the morning after breakfast Administered into the oropharynx, patient instructed to swallow continuously the accumulated liquid Nothing to eat or drink for 30 minutes after administration	
Outcomes	**Outcomes assessed on day 15** **Primary outcomes:** Reduction in the esophageal eosinophil load achieved with budesonide Eosinophil load – mean eosinophil number measured in a total of 40 higher‐power fields taken from 4 biopsy specimens each of the proximal and mid esophagus Also compared post‐treatment mean eosinophils per high‐power field < 5 cells, 5 to 20 cells, > 20 cells **Secondary outcomes:** the effects of budesonide on reducing eosinophilic esophagitis‐associated symptoms and on eosinophilic esophagitis relevant biomarkers in the esophagus and peripheral blood	
Notes	**Funding source:** Swiss National Science Foundation AstraZeneca, Switzerland **Other:** included patients who completed a 4‐week run‐in period after stopping eosinophilic esophagitis‐relevant therapies (steroids, leukotriene antagonists, histamine blockers, mast cell stabilizers) besides proton pump inhibition **Conflicts of interest:** Dr. Christoph Beglinger and Dr. Simon received research support from AstraZeneca	
***Risk of bias***		
**Bias**	**Authors' judgement**	**Support for judgement**
Random sequence generation (selection bias)	Low risk	Computer‐generated, coded randomization method.
Allocation concealment (selection bias)	Unclear risk	How allocation was concealed is not addressed in the manuscript or trial registry.
Blinding of participants and personnel (performance bias) All outcomes	Low risk	Patients, study center personnel, laboratory personnel, and the sponsor were blinded to treatment allocation.
Blinding of outcome assessment (detection bias) All outcomes	Unclear risk	Neither the manuscript nor the trial registry specifically address whether the interpreting pathologist(s) were blinded to treatment arm.
Incomplete outcome data (attrition bias) All outcomes	Low risk	All randomized patients completed the study and were included in the analysis.
Selective reporting (reporting bias)	Low risk	All pre‐specified outcomes including histologic, endoscopic, and symptoms were reported.
Other bias	Low risk	No major baseline differences between groups. No other concerns.

**Table:** Straumann 2011

***Study characteristics***		
Methods	**RCT design:** randomized, placebo‐controlled, single‐center clinical trial **Single‐center or multi‐center**: single‐center **Countries:** Switzerland **Study dates**: December 2005 to December 2008	
Participants	**Active EoE or inactive EoE at beginning of study**: inactive eosinophilic esophagitis at beginning of the study **EoE definition/diagnostic criteria**: clinically, endoscopically, and histologically confirmed eosinophilic esophagitis after proton pump inhibitor trial **Inclusion criteria:** Patients older than 14 years with clinically, endoscopically, and histologically confirmed eosinophilic esophagitis, previously brought successfully into remission with short‐term (= 15 days), high‐dose (= 2 mg/day) budesonide were eligible Remission was defined as follows: (1) a mean cell density for eosinophilic tissue infiltration of fewer than 5 eosinophils/high‐power field on esophageal histology biopsies, and (2) symptom score of 2 points or fewer **Exclusion criteria:** Current use of specific treatments for eosinophilic esophagitis Secondary causes of esophageal eosinophilia Intolerance to budesonide Concomitant therapies for any reason that may affect assessment Use of an investigational drug with 30 days of entering the study Recent history or suspicion of current drug abuse and alcohol abuse Positive serum pregnancy test at the screening visit Any unstable serious co‐existing medical condition **Age**: Budesonide: mean (SD): 38.0 ± 11.7 Placebo: mean (SD): 34.0 ± 13.9 **Sex**: Budesonide: m/f: 13/1 Placebo: m/f: 11/3 **Number randomized:** 14 intervention; 14 placebo **Number reaching end of study** Budesonide: 9/14 Placebo: 5/14	
Interventions	**Study group 1**: placebo: 0.9% saline 1 mL via an inhalation system consisting of a PARI UNI light compressor and PARI TIA nebulizer, twice per day at bedtime and in the morning after breakfast; patients instructed to nebulize the suspension into the oral cavity and to swallow continuously the accumulated liquid **Study group 2**: treatment arm: 0.5 mg/day budesonide as 0.25 mg/mL suspension formulation applied using an inhalation system consisting of a PARI UNI light compressor and PARI TIA nebulizer, twice per day at bedtime and in the morning after breakfast; patients instructed to nebulize the suspension into the oral cavity and to swallow continuously the accumulated liquid	
Outcomes	**Primary outcomes:** The primary endpoint was to determine the ability of a long‐term budesonide therapy in maintaining eosinophilic esophagitis in histologic remission, defined as an esophageal eosinophil load of fewer than 5 eosinophils per high‐power field. Eosinophil load is defined as the mean eosinophil number measured in a total of 40 high‐power fields from 2 x 4 biopsy specimens each, taken from the proximal and distal esophagus **Secondary outcomes:** To determine the course of the disease under placebo (natural course) after successful short‐term therapy To assess the ability of budesonide in controlling eosinophilic esophagitis symptoms To determine the effect of long‐term corticosteroid therapy on markers of inflammation and tissue damage, as well as on signs of esophageal remodeling To evaluate the safety of long‐term topical esophageal corticosteroid administration	
Notes	**Funding source:** supported by grants from the Swiss National Science Foundation AstraZeneca **Conflicts of interest:** Christoph Beglinger and Hans‐Uew Simon received research support for the clinical trial from AstraZeneca	
***Risk of bias***		
**Bias**	**Authors' judgement**	**Support for judgement**
Random sequence generation (selection bias)	Low risk	Computer‐generated randomization method.
Allocation concealment (selection bias)	Unclear risk	The method of allocation concealment is not fully explained.
Blinding of participants and personnel (performance bias) All outcomes	Low risk	Patients, study center, laboratory staff, and sponsor were blinded to treatment allocation.
Blinding of outcome assessment (detection bias) All outcomes	Unclear risk	Patients, study center, laboratory staff, and sponsor were blinded to treatment allocation. The manuscript does not specifically address whether the interpreting pathologist was blinded.
Incomplete outcome data (attrition bias) All outcomes	Low risk	There were data on all patients randomized at either study end or following clinical relapse.
Selective reporting (reporting bias)	Low risk	Per NCT00271349 and the manuscript, all pre‐specified outcomes were reported.
Other bias	Low risk	No major baseline differences between groups. No other concerns.

**Table:** Straumann 2013

***Study characteristics***		
Methods	**RCT design and number of study arms:** randomized, placebo‐controlled, parallel‐group, 2 arms **Single‐center or multi‐center**: single‐center **Countries**: Switzerland **Study dates**: August 2010 to June 2011	
Participants	**Active EoE of inactive EoE at beginning of study:** active EoE **EoE definition/diagnostic criteria**: patient aged 18 to 75 with previously clinically, endoscopically, and histologically confirmed EoE (according to Liacouras 2011 definition) **Age at beginning of study per group:** Placebo: 38.83 ± 14.48 years OC000459 (timapiprant): 43.71 ± 13.49 years **Sex (m/f) per study group:** Placebo: 8/4 OC000459 (timapiprant): 14/0 **Number randomized per study group:** Placebo: 12 OC000459 (timapiprant): 14 **Number reaching end of study per study group:** Placebo: 12 OC000459 (timapiprant): 14 **Inclusion criteria:** Aged 18 to 75 Previously diagnosed and symptomatic isolated EoE Able to swallow placebo medication successfully under supervision in the clinic Free of all medications for EoE (including topical steroids) for at least 2 weeks prior to baseline and free of systemic steroids for at least 90 days before screening. A PPI is allowed if required for treatment of secondary reflux. Inadequate response to topical and/or systemic corticosteroid therapy (e.g. corticosteroid refractory, corticosteroid dependency or necessity of high doses to control the inflammation) Dysphagia with almost every intake of solids Relevant eosinophilic infiltration of the epithelial layer of the esophagus (mean cell density of ≥ 20 eos/hpf in 8 biopsies) Exclusion of other causes of esophageal or systemic eosinophilia Gastroesophageal reflux disease was excluded by proton pump inhibitor (PPI) pretreatment plus negative endoscopy for signs of reflux disease and pH monitoring (optional) **Exclusion criteria:** Other causes of esophagitis (GERD, peptic ulceration, infection, etc.) Other causes of esophageal or generalized eosinophilia (i.e. hypereosinophilic syndromes, parasitic infection, GERD) The patient’s EoE is dependent on the level of seasonal allergies and the patient’s participation in the study will occur during the allergy season History of abnormal gastric or duodenal eosinophilia (e.g. HES, Churg Strauss vasculitis, EG or a parasitic infection) Receipt of forbidden prescribed or over‐the‐counter medication with the 4 weeks prior to the baseline visit and for the duration of the trial, including vitamins and herbal remedies	
Interventions	**Study group 1**: placebo; identical‐appearing placebo tablets **Study group 2**: OC000459 (timapiprant) monotherapy; 100 mg tablets, twice‐daily after meals for 8 weeks	
Outcomes	**Primary outcomes of the study:** Reduction in the esophageal eosinophil load, defined as the mean eosinophil number measured in a total of 40 hpf from 2 x 4 biopsies taken from the proximal and distal esophagus **Secondary outcomes:** The effects of OC000459 (timapiprant) on patient‐reported outcomes (PROs), on endoscopic alterations, and on EoE‐relevant biomarkers in the esophagus and peripheral blood Safety and tolerability	
Notes	**Funding source:** Swiss National Science Foundation Oxagen LTD **Conflicts of interest:** Alex Straumann and Christian Bussmann received research support for the clinical trial from the sponsor. Mike Perkins, Lisa Pearce Collins, Roy Pettipher, Michael Hunter, and Jan Steiner are employed by Oxagen Ltd.	
***Risk of bias***		
**Bias**	**Authors' judgement**	**Support for judgement**
Random sequence generation (selection bias)	Low risk	Computer‐generated randomization method.
Allocation concealment (selection bias)	Unclear risk	Allocation concealment was not explicitly addressed in the manuscript or registry.
Blinding of participants and personnel (performance bias) All outcomes	Low risk	Patients, study center personnel, and the sponsor were blinded.
Blinding of outcome assessment (detection bias) All outcomes	Unclear risk	It is not explicitly mentioned that the interpreting pathologist was blinded to allocation.
Incomplete outcome data (attrition bias) All outcomes	Low risk	All randomized patients completed the study and were included in the analysis.
Selective reporting (reporting bias)	Low risk	Trial registry is available and all pre‐specified outcomes of interest were reported in the pre‐specified way.
Other bias	Low risk	No major baseline differences between groups. No other concerns.

**Table:** Straumann 2020

***Study characteristics***		
Methods	**RCT design and number of study arms**: phase 3, randomized, double‐blind, placebo‐controlled trial **Singe‐center or multi‐center:** multi‐center **Countries**: multiple **Study dates:** 4 August 2016 to 4 October 2016	
Participants	**Active EoE or inactive EoE at beginning of study?** inactive **EoE definition/diagnostic criteria**: previously confirmed diagnosis of PPI‐refractory EoE according to consensus guidelines (Dellon et al. Gastroenterology 2018; Lucendo AJ et al. United European Gastroenterol J. 2017) **Inclusion criteria:** Confirmed clinico‐histologic remission at baseline after achieving study goals of a double‐blind, controlled induction treatment study (EOS‐1) with BOT 1.0 mg twice‐daily, or be receiving open‐label induction with budesonide orodispersible tablet (BOT) 1.0 mg twice‐daily for 6 weeks Clinical remission defined as a severity of ≤ 2 points on 1‐ to 10‐point numerical rating scale (NRS) for dysphagia and a severity of ≤ 2 points on a 0‐ to 10‐point NRS for odynophagia on each day in the last week of induction treatment Histologic remission defined as peak eosinophil count < 16 eos/mm^2^ hpf (corresponding to < 5 eos/hpf via prior method) at baseline, measured in hpf derived from 6 biopsies, 2 of each esophageal third **Exclusion criteria:** Clinical and endoscopic suspicion for gastroesophageal reflux disease; achalasia or scleroderma; evidence of reasons for esophageal eosinophilia other than EoE; pathologic eosinophilic infiltration in gastric and duodenal biopsies History of esophageal surgery at any time or of esophageal dilation procedures within the last 8 weeks before induction treatment Any relevant systemic disease **Age at beginning of study per study group:** Placebo: 36 ± 9.9 BOT 0.5 mg twice‐daily: 36 ± 10.9 BOT 1.0 mg twice‐daily: 37 ± 11.1 **Sex (m/f) per study group:** Placebo: 55/13 BOT 0.5 mg twice‐daily: 57/11 BOT 1.0 mg twice‐daily: 57/11 **Number randomized per study group:** Placebo: 68/204 BOT 0.5 mg twice‐daily: 68/204 BOT 1.0 mg twice‐daily: 68/204 **Number reaching end of study per study group:** Placebo: 23/68 BOT 0.5 mg: 59/68 BOT 1.0 mg: 59/68	
Interventions	**Study group 1:** placebo **Study group 2:** BOT 0.5 mg twice‐daily **Study group 3:** BOT 1.0 mg twice‐daily	
Outcomes	**Primary**: Remission at week 48 **Secondary**: Rate of histologic relapse (defined in primary outcome) at 48 weeks Change in the peak eos/mm^2^ hpf from baseline to EoT Rate of clinical relapse (defined in primary outcome) Food impaction requiring endoscopic intervention or endoscopic dilation Rate of clinical remission (eosinophilic esophagitis activity index‐patient reported outcome score ≤ 20)	
Notes	**Funding source:** Dr Falk Pharma GmbH, Freiburg, Germany **Conflicts of interest:** Alex Straumann reports receiving consulting fees from Allakos, AstraZeneca, EsoCap, Dr Falk Pharma, Gossamer, GSK, Receptos‐Celgene, and Regeneron‐Sanofi; receiving lecture fees from Dr Falk Pharma and Vifor; receiving payment from Dr Falk Pharma for the development of educational presentations; receiving payment from AstraZeneca for serving as member independent data monitor committee; and serving as a board member for European Society of Eosinophilic Oesophagitis (EUREOS) and The International Gastrointestinal Eosinophil Researchers (TIGERS). Alfredo Lucendo reports receiving consulting fees from EsoCap, and Dr Falk Pharma; receiving lecture fees from Dr Falk Pharma; and serving as a board member for EUREOS. Stephan Miehlke reports receiving consulting fees from Celgene, Dr Falk Pharma, and EsoCap; receiving lecture fees from Dr Falk Pharma and Vifor; receiving payment for the development of educational presentations from Dr Falk Pharma; and serving as a board member for EUREOS; Michael Vieth reports receiving lecture fees from Dr Falk Pharma, Janssen‐Cilag, Malesci, Menarini, Olympus, and Shire. Christoph Schlag reports receiving consulting fees from Adare, Celgene, EsoCap, and Dr Falk Pharma; receiving lecture fees from Dr Falk Pharma; and serving as a board member for EUREOS. Luc Biedermann reports receiving consulting fees from Calypso Biotech SA, Switzerland; Esocap AG, Switzerland; Vifor AG, Switzerland; receiving lecture fees from Dr Falk Pharma, Germany; Sanofi‐Aventis AG, Switzerland; and serving as a board member for EUREOS. Cecilio Santander Vaquero reports receiving lecture fees from Allergan and receiving payment for the development of educational presentations from Laborie. Constanza Ciriza de los Rios reports receiving consulting and/or lecture fees from Allergan and Casen Recordati. Ahmed Madisch reports receiving lecture fees from Dr Falk Pharma. Jamal Hayat reports receiving consulting fees from Dr Falk Pharma; and receiving lecture fees from Dr Falk Pharma. Ulrike von Arnim reports receiving consulting fees from Abbvie, Amgen, Eso Cap, Janssen, MSD, and Takeda; receiving lecture fees from Abbvie, Falk Foundation, Janssen, MSD, Reckitt Benckiser, Takeda, and Vifor; and serving as a board member for EUREOS. Albert Jan Bredenoord reports receiving research funding from Nutricia, Norgine, SideSleepTechnologies, and Bayer; receiving lecture and/or consulting fees from Laborie, Arena, EsoCap, Diversatek, Medtronic, Dr Falk Pharma, Calypso Biotech, Thelial, Robarts, Reckett Benkiser, Regeneron, Celgene, Bayer, Norgine, AstraZeneca, Almirall, Arena, and Allergan. Stefan Schubert reports receiving consulting fees from Abbvie, Takeda, Biogen, Amgen, and Janssen; receiving lecture fees from Abbvie, Dr. Falk Pharma, Takeda, Biogen, Amgen and Janssen; and serving as a board member for MSD, Takeda, and Janssen. Ralph Mueller reports being an employee of Dr Falk Pharma GmbH. Roland Greinwald reports being an employee of Dr Falk Pharma GmbH. Alain Schoepfer reports receiving consulting fees from Abbvie, Adare, Celgene, Dr Falk Pharma, Janssen‐Cilag, MSD, Pfizer, Receptos, Regeneron, and Vifor; receiving lecture fees from Abbvie, Celgene, Dr Falk Pharma, Pfizer, Receptos, Regeneron, and Vifor; and serving as a board member for TIGERS. Stephen Attwood reports receiving consulting fees from Dr Falk Pharma, EsoCap, AstraZeneca, and Reckitt Benkiser; receiving lecture fees from Dr Falk Pharma, Medtronic; receiving payment for the development of educational presentations from Dr Falk Pharma. The remaining authors disclose no conflicts.	
***Risk of bias***		
**Bias**	**Authors' judgement**	**Support for judgement**
Random sequence generation (selection bias)	Low risk	Computer‐generated randomization method.
Allocation concealment (selection bias)	Low risk	Interactive Web Response System using randomly generated sequence. Patients received identical‐appearing medications.
Blinding of participants and personnel (performance bias) All outcomes	Low risk	Patients, investigators, and their study team, the sponsor, monitoring staff, central laboratory, and central pathologist were all kept blinded.
Blinding of outcome assessment (detection bias) All outcomes	Low risk	Patients, investigators, and their study team, the sponsor, monitoring staff, central laboratory, and central pathologist were all kept blinded.
Incomplete outcome data (attrition bias) All outcomes	Low risk	There was comparable attrition between the treatment arms with expected attrition in the placebo arm (per design, suspect clinical). Suspected clinical failures were prematurely withdrawn from the double‐blind phase of the study; exactly how this was handled statistically somewhat unclear. All attrition explained in supplementary material.
Selective reporting (reporting bias)	Low risk	All outcomes of interest were reported and qualified as a priori.
Other bias	Low risk	There were no consistent imbalances between treatment arms, though this was subjective and P values were not provided.

**Table:** Tytor 2021

***Study characteristics***		
Methods	**RCT design and number of study arms**: RCT; 2 arms **Single‐center or multi‐center:** multi‐center (2 centers: NU‐Hospital Group, Trollhattan, and The Central Hospital, Skovde in Sweden **Countries**: Sweden **Study dates**: April 2012 to August 2018	
Participants	**Active EoE or inactive EoE at beginning of study:** active EoE **EoE definition/diagnostic criteria:** at least 15 eosinophils per high‐power field (magnification 10 times 40 = x400) in any field of view in any esophageal biopsy and concurrent symptoms of esophageal dysfunction, mainly dysphagia **Inclusion criteria:** Age 18 years Newly diagnosed EoE with a peak eosinophil count of at least 15 cells per HPF in any area in any of at least 6 esophageal biopsies including at least 3 biopsies from the upper‐ respective lower‐third part of the esophagus Total WDS score 5 **Exclusion criteria:** Ongoing infection – locally or general Glaucoma Planned elective surgery during the treatment period Systemic or topical corticosteroid treatment during the last 4 months Pregnancy, ongoing or planned. Other known cause of dysphagia. Other treatment that might affect dysphagia or motility during the trial (e.g. cisapride, erythromycin) Allergy or intolerance to any component in the drug Contraindication to steroid treatment (immune deficiency or immune suppression, gastroduodenal ulcer, diabetes mellitus) PPIs were not allowed from 2 weeks before the start and during the treatment period Other cause of dysphagia (cancer, autoimmune disease, neurologic disease) Inability to understand and provide autonomous informed consent **Age at beginning of study per study group**: Median (IQR) Placebo = 49.0 (37.0 to 63.0) Mometasone furoate = 42.0 (34.0 to 50.0) **Sex (m/f) per study group**: Placebo m/f = 18/1 Mometasone furoate m/f = 15/2 **Number randomized per study group:** Placebo = 19 Mometasone furoate = 17 **Number reaching end of study per study group:** Placebo = 17 Mometasone furoate = 16	
Interventions	**Study group 1:** placebo **Study group 2:** mometasone furoate 4 spray doses at 50 µg by mouth to be swallowed 4 times daily after meals with no eating or drinking allowed 30 minutes after intake Duration of treatment is 8 weeks	
Outcomes	**Primary outcomes of the study**: Watson Dysphagia Scale Score (WDS) (time frame: 2 months) Difference in WDS score during treatment in active as compared to placebo group **Secondary outcomes of the study:** The EORTC QLQ‐OES18 dysphagia scale, the eating scale and choking item (time frame: 2 months) "global health and social functioning dimensions" of SF‐36 (time frame: 2 months)	
Notes	**Funding source:** the study was supported by funding provided by the The Health & Medical Care Committee of the Regional Executive Board, Region Vastra Gotaland (Project 109901) **Conflicts of interest:** no potential conflict of interest was reported by the author(s)	
***Risk of bias***		
**Bias**	**Authors' judgement**	**Support for judgement**
Random sequence generation (selection bias)	Low risk	Computer‐generated randomization method.
Allocation concealment (selection bias)	Low risk	Treatment was allocated centrally. Intervention and placebo were delivered in similar packages.
Blinding of participants and personnel (performance bias) All outcomes	Low risk	"After providing informed consent, the patient was asked to follow the study nurse who separately registered the patient, noted the randomization number of the treatment drug package and handed it over to the study participant without knowledge of its contents".
Blinding of outcome assessment (detection bias) All outcomes	Low risk	The author confirmed in November 2022 that the random code was not broken for assessors. Patient questionnaires were returned anonymously and those analyzing did not have group data.
Incomplete outcome data (attrition bias) All outcomes	Low risk	No imbalance between placebo and intervention arm. All attrition was explained.
Selective reporting (reporting bias)	Low risk	Did not numerically present the secondary endpoints, although the authors mentioned that was due to lack of significance. We emailed the authors who sent the full data set.
Other bias	Low risk	No major baseline differences between groups. No other concerns.

AAF: amino acid formula; ASA: American Society of Anesthesiologists; BMI: body mass index; CG: control group; DB: double‐blind; DSD: daily symptom diary; DSQ: Dysphagia Symptom Questionnaire; EEsAI: Eosinophilic Esophagitis Activity Index; EGD: esophagogastroduodenoscopy; EG: eosinophilic gastroenteritis; EoE: eosinophilic esophagitis; EoEHSS: EoE histology scoring system; eos: eosinophils; EoT: end of treatment; EREFS: EoE Endoscopic Reference Score; f: female; FFED: four‐food elimination diet; GERD: gastroesophageal reflux disease; GI: gastrointestinal; H2: histamine H2‐receptor; HES: hypereosinophilic syndrome; hpf: high‐power field; HRQoL: health‐related quality of life; IG: intervention group; IQR: interquartile range; IV: intravenous; LOCF: last observation carried forward; m: male; NR: not reported; NRS: numerical rating scale; OVB: oral viscous budesonide; PEESS: Pediatric Eosinophilic Esophagitis Symptom Severity; PPI: proton pump inhibitor; PRO: patient‐reported outcome; RCT: randomized controlled trial; SD: standard deviation; SDI: Straumann Dysphagia Instrument; SGC: swallowed glucocorticoids; VAS: visual analogue scale; WDS: Watson Dysphagia Scale

### Characteristics of excluded studies [ordered by study ID]

**Table:**

Study	Reason for exclusion
Braathen 2006	Wrong population ‐ not EoE at baseline
Ceves 2005	Wrong study type ‐ not an RCT
Comer 2017	Wrong population ‐ not EoE
Della 2017	Wrong study type
Dellon 2020c	Wrong population
Eluri 2017	Wrong study type
Eluri 2017a	Wrong study type
EUCTR2014‐002465‐30‐IT 2014	Wrong study type
Francis 2012	Wrong study type
Hefner 2016	Wrong population
Helou 2008	Wrong study type
Hudgens 2017	Wrong study type
JPRN‐UMIN000021041 2016	Wrong study type ‐ not randomized
JPRN‐UMIN000026704 2017	Wrong study type
Kagalwalla 2006	Wrong study type ‐ not an RCT
Kavitt 2016	Wrong intervention (dilation)
Kruszewski 2016	Wrong study type
Kuzumoto 2021	Wrong study type
Molina‐Infante 2017	Wrong study type
NCT01458418 2011	Abandoned RCT ‐ no results
NCT01498497 2011	Wrong study type ‐ not an RCT
NCT01702701 2012	Abandoned RCT ‐ no results
NCT01821898 2013	Abandoned RCT ‐ no results
NTR4892 2014	Wrong study type ‐ not an RCT
Safroneeva 2015	Wrong study type ‐ not an RCT
Safroneeva 2018	Wrong study type ‐ not an RCT
Safroneeva 2018a	Wrong study type ‐ not an RCT
Savarino 2015	Wrong study type ‐ not an RCT
Song 2020	Wrong study type ‐ not an RCT
Spergel 2002	Wrong study type ‐ not an RCT
Spergel 2005	Wrong study type ‐ not an RCT
Syverson 2020	Wrong study type ‐ not an RCT
Tripp 2017	Wrong population – not EoE
Vazquez‐Elizondo 2013	Wrong study type ‐ not an RCT
Wang 2017	Wrong study type ‐ not an RCT
Warners 2016	Wrong study type ‐ not an RCT
Wechsler 2017	Wrong study type ‐ not an RCT
Wright 2020	Wrong population – not EoE
Wright 2021	Wrong population

EoE: eosinophilic esophagitis; RCT: randomized controlled trial

### Characteristics of studies awaiting classification [ordered by study ID]

**Table:** Amini 2022

Methods	RCT
Participants	30 children
Interventions	Both groups received the same treatment (elimination diet, topical steroid, and proton pump inhibitor). A synbiotic (KidiLact) was added to the medication regimen of 15 patients (case), while the next 15 patients received a placebo (control).
Outcomes	Severity and frequency of symptoms were assessed with a checklist derived from a validated scoring tool in both groups before and after 8 weeks of treatment
Notes	This study was identified during our update search and will be fully included when this review is updated

**Table:** NCT01846962 2012

Methods	RCT
Participants	64
Interventions	6‐food elimination diet Fluticasone Budesonide Oral viscous budesonide (OVB)
Outcomes	Primary: Efficacy clinical severity score Secondary: Efficacy severity score for endoscopy and histology
Notes	Author contacted on 22 March 2022 for update ‐ no response received

RCT: randomized controlled trial

### Characteristics of ongoing studies [ordered by study ID]

**Table:** ACTRN12619000141145 2019

Study name	'Two food group elimination diet versus swallowed fluticasone for the management of adult eosinophilic esophagitis, a single‐centred randomised prospective study'
Methods	RCT
Participants	194
Interventions	Arm 1 (swallowed topical steroids): fluticasone 500 µg swallowed twice‐daily using a metered dose inhaler for 8 weeks Arm 2 (2‐food group elimination diet): eliminating cow's milk and wheat under the guidance of a gastrointestinal dietitian for 8 weeks
Outcomes	**Primary outcomes**: Proportion of patients that have responded to each intervention as defined by histological remission. Histological remission will be defined as reduction of eosinophils to < 15 per HPF on both the distal and proximal esophageal biopsies. **Secondary outcomes:** Comparison of the Dysphagia Symptom Questionnaire between proton pump inhibitor responsive eosinophilia and patients with eosinophilic esophagitis Comparing the differences in eosinophil counts (using histology, esophageal biopsies) between PPI responsive eosinophilia and eosinophilic esophagitis Comparing the differences in serum anti‐TTG antibodies (using standard laboratory blood tests) between PPI responsive eosinophilia and eosinophilic esophagitis Comparing the differences in Dysphagia Symptom Score between PPI responsive eosinophilia and eosinophilic esophagitis Comparing the differences in Adult Eosinophilic Esophagitis Quality of Life Questionnaire between PPI responsive eosinophilia and eosinophilic esophagitis Comparing the differences between swallowed topical fluticasone 500 µg twice‐daily for 8 weeks (Arm 1) and the 2‐food (cow's milk and wheat) elimination diet for 8 weeks in Dysphagia Symptom Score Comparing the differences in serum IgE levels (using standard laboratory blood tests)between the PPI responsive eosinophilia and eosinophilic esophagitis
Starting date	4 February 2019
Contact information	Dr Abdulnasser Lafta nasserhawas@gmail.com
Notes	—

**Table:** ACTRN12621001406897

Study name	'Efficacy of fucoidan for eosinophilic oesophagitis: a phase 2 pilot study'
Methods	RCT phase 2
Participants	Adults
Interventions	Patients will be treated with steroids and proton pump inhibitor (PPI) therapy for a minimum of 6 weeks as part of the routine clinical management (prescribed by Gastroenterologist and/or in consultation with participants own GP). Only the study medication will be provided as part of the study. Participants will take the study medication in combination with routine clinical management for an initial period of 6 weeks, and then continue taking the study medication for a further 6 weeks (total fucoidan/placebo supplementation will be 12 weeks). Participants will be randomized to one of 2 treatment groups. (1) A formulation containing 100 mg daily of 85% Maritech (Marinova, Tasmania, Australia) (2) Placebo: same formulation as the fucoidan supplement but without the active ingredient The active treatment will be administered via an oral gel, once per day. Participants will be asked to drink the gel slowly, allowing it to coat the esophagus thoroughly, and asked to refrain from eating for 30 minutes afterwards.
Outcomes	**Primary outcomes:** Change in EoE disease activity based on esophageal eosinophil count (13 weeks post treatment initiation) Change in EoE disease activity based on change in Dysphagia Symptom Questionnaire score of > 30% (13 weeks post treatment initiation) **Secondary outcomes:** Esophageal microbiome: diversity score (count of identified operational taxonomic units) (13 weeks post treatment initiation) Fecal microbiome: diversity score (count of identified operational taxonomic units) (13 weeks post treatment initiation) Esophageal biopsy immune gene expression profiling: the ratio of Th/Th2 inflammatory pathways and cells (13 weeks post treatment initiation) Fecal microbiome: microbial composition (13 weeks post intervention initiation) Esophageal microbiome: microbial composition (13 weeks post treatment initiation)
Starting date	13 February 2023
Contact information	n.west@griffith.edu.au and r.ramsey@griffith.edu.au
Notes	—

**Table:** EUCTR2017‐003516‐39‐ES 2021

Study name	'A phase III clinical study in adult and adolescent patients with eosinophilic inflammation of the gullet to prove superiority compared to placebo of an episodic and/or a continuous 48‐week treatment with budesonide orodispersible tablets for maintaining remission'
Methods	RCT
Participants	110
Interventions	Budesonide 1 mg orodispersible tablets Budesonide 0.5 mg orodispersible tablets
Outcomes	**Primary outcome:** Proportion of patients free of treatment failure after a 48 weeks DB treatment phase **Secondary outcomes:** Proportion of patients with histological relapse at DB week 48 Proportion of patients with clinical relapse, or who have experienced a food impaction, who needed endoscopic intervention during the DB treatment phase Proportion of patients in clinico‐histological remission at DB week 48
Starting date	—
Contact information	—
Notes	—

**Table:** EUCTR2017‐003737‐29‐ES 2019

Study name	'Double‐blind (neither physician nor patient knows of the actual treatment which can be with or without active substance), randomized (patient will be allocated to a certain treatment group by chance), placebo‐controlled (one of the treatment groups receives medication without active substance), phase II/III study on the efficacy and tolerability of oral budesonide suspension in comparison with placebo in children and adolescents with eosinophilic esophagitis'
Methods	RCT
Participants	75
Interventions	Budesonide oral suspension (0.2 mg/mL)
Outcomes	**Primary outcome:** Double‐blind phase: rate of patients with pathological remission and clinical response at DB week 12 **Secondary outcomes:** Double‐blind phase: to further assess efficacy of budesonide oral suspension in children and adolescents with eosinophilic esophagitis (EoE)
Starting date	—
Contact information	—
Notes	—

**Table:** EUCTR2019‐002871‐32‐ES 2019

Study name	'A study of benralizumab in patients with eosinophilic esophagitis'
Methods	RCT
Participants	170
Interventions	Benralizumab
Outcomes	**Primary outcomes:** Dual‐primary endpoints: Proportion of patients with a histologic response at week 24, defined as a peak esophageal intraepithelial eosinophil count = 6 eos/hpf Changes from baseline in DSQ score at week 24 **Secondary outcomes:** DB treatment period: To evaluate the effect of benralizumab dose regimen 1 on: Clinical features of EoE and disease activity Patient‐reported QOL measures Healthcare resource utilization due to EoE Patient‐reported measures of disease severity and health status To assess the PK and immunogenicity of benralizumab dose regimen 1 in patients with EoE To assess the safety and tolerability of benralizumab dose regimen 1 in patients with EoE
Starting date	—
Contact information	—
Notes	—

**Table:** EUCTR2019‐004391‐19‐NL 2020

Study name	'A study of benralizumab in patients with eosinophilic esophagitis'
Methods	RCT
Participants	170
Interventions	Benralizumab
Outcomes	**Primary outcomes:** Dual‐primary endpoints: Proportion of patients with a histologic response at week 24, defined as a peak esophageal intraepithelial eosinophil count = 6 eos/hpf Changes from baseline in DSQ score at week 24 **Secondary outcomes:** DB treatment period: To evaluate the effect of benralizumab dose regimen 1 on: Clinical features of EoE and disease activity Patient‐reported QOL measures Healthcare resource utilization due to EoE Patient‐reported measures of disease severity and health status To assess the PK and immunogenicity of benralizumab dose regimen 1 in patients with EoE To assess the safety and tolerability of benralizumab dose regimen 1 in patients with EoE
Starting date	30 July 2020
Contact information	cpaterson@allakos.com
Notes	Sponsored by Allakos Inc.

**Table:** EUCTR2020‐000082‐16‐DE 2020

Study name	'A study to investigate the efficacy and tolerability of the drug ESO‐101 in adult patients with inflammation of the esophagus'
Methods	RCT
Participants	42
Interventions	ESO‐101
Outcomes	**Primary outcome:** Absolute change in peak eosinophil count from baseline to EOT **Secondary outcomes:** Histological response and clinical symptoms Clinical response assessed by patient‐reported outcome Endoscopic response Safety and tolerability Patient‐reported treatment satisfaction
Starting date	—
Contact information	—
Notes	—

**Table:** EUCTR2020‐001314‐37‐DE 2020

Study name	'Clinical study to show equal clinical efficacy of two dosing regimen of budesonid orodispersible tablets (twice daily vs. once daily) for treatment of inflammation of the esophagus'
Methods	RCT
Participants	242
Interventions	Budesonide 2 mg orodispersible tablets Budesonide 1 mg orodispersible tablets
Outcomes	**Primary outcomes:** Proportion of patients with histological remission **Secondary outcomes:** EoE‐associated clinical, endoscopic, and histological findings after 6? weeks treatment with budesonide orodispersible tablets Safety and tolerability as assessed by adverse events and laboratory parameters Patients’ quality of life
Starting date	—
Contact information	—
Notes	—

**Table:** EUCTR2020‐003226‐23‐BE 2020

Study name	'A study to assess the safety and efficacy of oral etrasimod in adult participants with eosinophilic esophagitis VOYAGE'
Methods	RCT
Participants	96
Interventions	Etrasimod
Outcomes	Percent change from baseline in esophageal peak eosinophil count (PEC) Absolute change from baseline in Dysphagia Symptom Questionnaire (DSQ) score (time frame: baseline to week 16) Absolute change from baseline in esophageal PEC (time frame: baseline to week 16) Number and severity of adverse events (double‐blind treatment period and extension treatment period) (time frame: up to approximately 56 weeks (24 weeks of double‐blind treatment period, 28 weeks of extension treatment period, and 4 weeks of safety follow‐up period)) Proportion of participants with esophageal PEC < 15 eosinophils per high‐powered field (eos/hpf) (time frame: baseline to week 16) Proportion of participants with esophageal PEC = 6 eos/hpf (time frame: baseline to week 16)
Starting date	—
Contact information	—
Notes	—

**Table:** EUCTR2020‐004336‐16‐DE 2021

Study name	'A study to evaluate the efficacy and safety of CC‐93538 in adult and adolescent patients who have eosinophilic esophagitis'
Methods	RCT
Participants	399
Interventions	Cendakimab
Outcomes	**Primary outcomes:** Mean change in dysphagia days (DD), evaluated over the prior 14‐day period using the modified Daily Symptom Diary (mDSD), from baseline to week 24 Proportion of participants with eosinophilic histologic response defined as a peak esophageal eosinophil count < 6/high‐power field (hpf) at week 24 **Secondary outcomes:** To assess the efficacy of CC‐93538 versus placebo at 24 weeks in improving: Endoscopic features of eosinophilic esophagitis (EoE) Histologic features of EoE To assess the persistence of effect of CC‐93538 at 48 weeks in reducing: Dysphagia symptoms Esophageal eosinophil counts To assess the persistence of effect of CC‐93538 through administration of a less frequent dosing regimen at 48 weeks in reducing: Dysphagia symptoms Esophageal eosinophil counts To assess the persistence of effect of CC‐93538 at 48 weeks in improving: Endoscopic features of EoE Histologic features of EoE To evaluate the time to and frequency of EoE flare events and use of rescue therapy during the study To evaluate the safety and tolerability of CC‐93538 including characterization of the immunogenicity profile To assess trough concentrations of CC‐93538 in participants with EoE
Starting date	—
Contact information	—
Notes	—

**Table:** Henry 2019

Study name	'A novel food additive removal diet for eosinophilic esophagitis (free study): conceptual design and clinical trial methods'
Methods	RCT (NCT03657771)
Participants	72 participants
Interventions	**Study group 1:** dairy‐free diet **Study group 2:** dairy‐free diet with food additive‐free diet
Outcomes	**Primary outcomes:** Eosinophils per high‐power field (eos/hpf) (time frame: 12 weeks) **Secondary outcomes:** Eosinophilic Esophagitis Endoscopic Reference Score (EREFS) (time frame: 12 weeks) **Other outcomes:** Pediatric Eosinophilic Esophagitis Symptom Severity Module (PEESS) (time frame: baseline, 4, 8, and 12 weeks) Pediatric Eosinophilic Esophagitis Symptom Severity Module (PEESS): parent report (time frame: baseline, 4, 8, and 12 weeks) Pediatric Quality of Life Inventory: Eosinophilic Esophagitis Module (PedsQL‐EoE) (time frame: baseline, 4, 8, and 12 weeks) Pediatric Quality of Life Inventory: Eosinophilic Esophagitis Module (PedsQL‐EoE): parent report (time frame: baseline, 4, 8, and 12 weeks)
Starting date	September 2018
Contact information	James Franciosi (review author): james.franciosi@nemours.org
Notes	**Funding source:** anonymous donor to the Nemours Foundation **Conflicts of interest:** not available

**Table:** IRCT20171230038142N14 2020

Study name	'Evaluation of efficacy of synbiotics in children with eosinophilic esophagitis'
Methods	RCT
Participants	40
Interventions	Omeprazole Diet Topical steroids Cidetabic synthetic intervention
Outcomes	Clinical symptoms (time point: before and 8 weeks after intervention); method of measurement: examination by a pediatrician Endoscopic findings (time point: before and 8 weeks after intervention); method of measurement: endoscopy in hospital by pediatric gastroenterologist Pathologic findings (time point: before and 8 weeks after intervention); method of measurement: gastric biopsy and eosinophil count by microscope
Starting date	—
Contact information	—
Notes	—

**Table:** IRCT20191211045703N1 2020

Study name	'Comparison of the efficay and side effects of nebulized oral Pulmicort and inhaler budesonide in patients with eosinophilic esophagitis'
Methods	RCT
Participants	60
Interventions	Nebulized oral Pulmicort Inhaler budesonide
Outcomes	The effect of oral budesonide nebulizer on improving eosinophilic esophagitis. Time point: patients in the oral Budesonide nebulizer group will receive 1 puff twice a day. If the effectiveness of the drug is not observed after 8 weeks, patients will receive 2 puffs twice a day. Then during weeks 4, 12, 8, and 16 patients will be followed up and examined. Method of measurement: at the beginning of the study and after the 16th week, patients will be re‐examined for histological and tissue pathology, as well as blood eosinophil counts and cortisol examination by enzyme‐linked immunosorbent test at 8 am. There will also be a routine laboratory test, such as hematology and biochemistry.
Starting date	—
Contact information	—
Notes	—

**Table:** JPRN‐jRCT2051200140

Study name	'A phase 3, multicenter, multinational, randomized, double‐blind, placebo‐controlled induction and maintenance study to evaluate the efficacy and safety of CC‐93538 in adult and adolescent subjects with eosinophilic esophagitis'
Methods	RCT
Participants	33 estimated
Interventions	CC‐93538 is administered subcutaneously at a dose of 360 mg weekly in adults and adolescent 12 years of age or older. 24 weeks after the initial dose, a dose of 360 mg is administered subcutaneously weekly or biweekly.
Outcomes	**Primary outcomes:** Induction phase endpoints at week 24: Change in DD clinical response: the mean change in dysphagia days (DD), evaluated over the prior 14‐day period using the modified Daily Symptom Diary (mDSD), from baseline to week 24 Eosinophil histologic response (≤ 6/hpf): the proportion of participants with eosinophilic histologic response defined as a peak esophageal eosinophil count ≤ 6/high‐power field (hpf) at week 24 **Secondary outcomes:** Induction phase key secondary endpoints at week 24: Eosinophil histologic response (< 15/hpf): proportion of participants with eosinophilic histologic response defined as a peak esophageal eosinophil count < 15/hpf at week 24 EREFS: mean change in the endoscopic features of EoE as measured by the EoE Endoscopic Reference Score (EREFS) from baseline to week 24 EoEHSS grade score: mean change in the mean adjusted histology grade score as measured by the EoE histology scoring system (EoEHSS) from baseline to week 24 EoEHSS stage score: mean change in the mean adjusted histology stage score as measured by the EoE histology scoring system (EoEHSS) from baseline to week 24 mDSD composite score: mean change in the modified Daily Symptom Diary (mDSD) composite score from baseline to week 24
Starting date	1 July 2021
Contact information	Name: Changliang Zhang Email: MG‐JP‐RCO‐JRCT@bms.com Affiliation: Bristol‐Myers Squibb
Notes	—

**Table:** NCT02873468

Study name	'Efficacy and safety of three doses of Florence oral suspension in adults with eosinophilic esophagitis'
Methods	RCT
Participants	116 estimated participants
Interventions	Florence 30 μg/mL Florence 60 μg/mL Florence 90 μg/mL Placebo
Outcomes	**Primary outcome**: proportion of participants presenting a histological response, defined as the presence of ≤ 6 eosinophils/high‐power field, at the end of treatment (time frame: 100 days) **Secondary outcomes**: incidence and severity of adverse events recorded during the study (time frame: 170 days)
Starting date	19 April 2021
Contact information	Arthur M Kummer, MD+551938879851 pesquisa.clinica@ems.com.br
Notes	—

**Table:** NCT03656380 2019

Study name	'Mepo for EoE Study'
Methods	RCT
Participants	72
Interventions	Mepolizumab 100 mg Mepolizumab 300 mg
Outcomes	Mean change in dysphagia from baseline to 3 months post‐treatment (time frame: baseline, month 3 post‐treatment) Absolute peak eosinophil count (measured in eos/hpf) after 3 months of treatment (time frame: after 3 months of treatment) Histologic response levels after 3 treatment months (time frame: after 3 months of treatment) Mean change in EoE Endoscopic Reference Score (EREFS) from baseline to 3 months post‐treatment (time frame: baseline, 3 months post‐treatment) Mean change in the Straumann Dysphagia Instrument (SDI) score from baseline to 3 months post‐treatment (time frame: baseline, 3 months post‐treatment) Proportion of participants with a clinical remission (EEsAI score of = 20 points) after 3 months of treatment (time frame: after 3 months of treatment) Proportion of participants with a clinical response (EEsAI score decrease of = 20 points) after 3 months of treatment (time frame: after 3 months of treatment)
Starting date	—
Contact information	—
Notes	—

**Table:** NCT03657771 2018

Study name	'A food additive removal diet for pediatric eosinophilic esophagitis FREE'
Methods	RCT
Participants	72
Interventions	DED FREE
Outcomes	**Primary outcome:** Eosinophils per high‐power field (eos/hpf) (time frame: 12 weeks) **Secondary outcomes:** Eosinophilic Esophagitis Endoscopic Reference Score (EREFS) (time frame: 12 weeks)
Starting date	—
Contact information	—
Notes	—

**Table:** NCT03781596

Study name	'EoE RCT fluticasone and omeprazole vs fluticasone alone'
Methods	RCT
Participants	100 estimated participants
Interventions	Fluticasone and omeprazole Fluticasone and placebo
Outcomes	**Primary outcome:** Change in esophageal eosinophilia (time frame: week 0 and week 8) **Secondary outcomes:** Change in endoscopic reference score (time frame: week 0 and week 8) Change in symptom scoring (time frame: week 0 and week 8)
Starting date	2 October 2018
Contact information	Claire P Daniels, M.D. 443‐904‐3353 claire.p.daniels.mil@mail.mil
Notes	—

**Table:** NCT04248712 2020

Study name	'Antihistamines in eosinophilic esophagitis ATEE'
Methods	RCT
Participants	50
Interventions	Famotidine Loratadine
Outcomes	**Primary outcomes:** Adverse Events (time frame: 12 weeks) Change in maximum eosinophil count (time frame: 12 weeks) **Secondary outcomes:** Change in endoscopic response, as measured by the endoscopic reference score (time frame: 12 weeks) Change in histologic response (time frame: 12 weeks) Change in symptoms of eosinophilic esophagitis, as measured by Dysphagia Symptom Questionnaire (time frame: 12 weeks)
Starting date	—
Contact information	—
Notes	—

**Table:** NCT04281108 2020

Study name	'Efficacy and safety APT‐1011 in adult subjects with eosinophilic esophagitis (EoE) (FLUTE‐2) FLUTE‐2'
Methods	RCT
Participants	143
Interventions	APT‐1011
Outcomes	**Primary outcomes:** Histologic responder rates at the end of the randomized withdrawal phase (RWS) (time frame: week 12 to week 52) Mean change in number of dysphagia episodes (time frame: week 0 to week 12) Percentage of APT‐1011 responders in Part A who remain symptomatic responders at the end of the RWS (time frame: week 0 to week 52) Week 12 histologic responder rates (time frame: week 12) **Secondary outcomes:** Change in EREFS from week 0 to week 12 (time frame: week 0 to week 12) Histological change from baseline to week 12 (time frame: week 0 to week 12) Mean change in dysphagia episodes (time frame: week 0 to week 52) Mean change in dysphagia‐free days (time frame: week 0 to week 52) Mean change in EREFS from week 0 to week 52 (time frame: week 0 to week 52) Mean change in PROSE day‐level difficulty swallowing (time frame: week 0 to week 12) Mean change in PROSE day‐level difficulty swallowing (time frame: week 0 to week 52) Mean change in PROSE day‐level symptom burden (time frame: week 0 to week 52) Mean change in PROSE symptom burden score (time frame: week 0 to week 12) Mean histologic change (time frame: week 0 to week 52) Mean number of dysphagia‐free days (time frame: week 0 to week 12) Percentage of participants with < 1 peak eos/HPF at week 12 (time frame: week 12) Percentage of participants with < 15 peak eos/HPF (time frame: week 12)
Starting date	—
Contact information	—
Notes	—

**Table:** NCT04394351 2020

Study name	'Study to investigate the efficacy and safety of dupilumab in pediatric patients with active eosinophilic esophagitis (EoE) EoE KIDS'
Methods	RCT
Participants	90
Interventions	Dupilumab
Outcomes	**Primary outcome:** Proportion of patients achieving peak esophageal intraepithelial eosinophil count = 6 eos/hpf (400×) (time frame: week 16) **Secondary outcomes:** Absolute change in Eosinophilic Esophagitis‐Endoscopic Reference (EoE EREFS) (time frame: week 16) Absolute change in EoE EREFS (time frame: week 52) Absolute change in mean eosinophilic esophagitis (EoE) Histology Scoring System (EoE‐HSS) (time frame: week 16) Absolute change in mean EoE‐HSS (time frame: week 52) Change in the proportion of days with 1 or more EoE signs as measured by the Pediatric EoE Sign/Symptom Questionnaire ‐ caregiver version (PESQ‐C) (time frame: week 16) Change in the proportion of days with 1 or more EoE signs as measured by the PESQ‐C (time frame: week 52) Change in the proportion of days with 1 or more EoE symptoms as measured by PESQ‐P (time frame: week 52) Change in the proportion of days with 1 or more EoE symptoms as measured by the Pediatric EoE Sign/Symptom Questionnaire ‐ patient version (PESQ‐P) (time frame: week 16) Change in the proportion of total segments within a day with 1 or more EoE signs as measured by PESQ‐C (time frame: week 16) Change in the proportion of total segments within a day with 1 or more EoE signs as measured by PESQ‐C (time frame: week 52) Change in the proportion of total segments within a day with 1 or more EoE symptoms as measured by PESQ‐P (time frame: week 16) Change in the proportion of total segments within a day with 1 or more EoE symptoms as measured by PESQ‐P (time frame: week 52) Change in the type 2 inflammation transcriptional signature (time frame: week 16) Change in the type 2 inflammation transcriptional signature (time frame: week 52) Change in total score as measured by the PEESSv2.0 ‐ caregiver version questionnaire (time frame: week 16) Concentration of functional dupilumab in serum (time frame: week 52) Incidence of treatment‐emergent adverse events (TEAEs) (time frame: week 52) Incidence of TEAEs leading to permanent discontinuation of study treatment (time frame: week 16) Incidence of TEAEs leading to permanent discontinuation of study treatment (time frame: week 52) Incidence of treatment‐emergent anti‐drug antibody (ADA) responses and titer (time frame: week 52) Incidence of TEAEs (time frame: week 16) Incidence of treatment‐emergent adverse events of special interest (AESIs) (time frame: week 16) Incidence of treatment‐emergent AESIs (time frame: week 52) Incidence of treatment‐emergent ADA responses and titer (time frame: week 16) Incidence of treatment‐emergent serious adverse events (SAEs) (time frame: week 52) Incidence of treatment‐emergent SAEs (time frame: week 16) Normalized Enrichment Scores (NES) for the relative change in the EoE diagnostic panel (EDP) transcriptome signature (time frame: week 52) NES for the relative change in the type 2 inflammation transcriptome signature (time frame: week 16) NES for the relative change in the type 2 inflammation transcriptome signature (time frame: week 52) NES for the relative change in the EDP transcriptome signature (time frame: week 16) Number of sign‐free days during the 14‐day period preceding week 16 as measured by the PESQ‐C (time frame: week 16) Number of sign‐free days during the 14‐day period preceding week 52 as measured by the PESQ‐C (time frame: week 52) Number of symptom‐free days during the 14‐day period preceding week 16 as measured by the PESQ‐P (patient version) (time frame: week 16) Number of symptom‐free days during the 14‐day period preceding week 52 as measured by the PESQ‐P (patient version) (time frame: week 52) Percent change in peak esophageal intraepithelial eosinophil count (eos/hpf) (time frame: week 16) Percent change in peak esophageal intraepithelial eosinophil count (eos/hpf) (time frame: week 52) Proportion of patients achieving peak esophageal intraepithelial eosinophil count = 6 eos/hpf (400×) (time frame: week 52) Proportion of patients achieving peak esophageal intraepithelial eosinophil count of < 15 eos/hpf (time frame: week 16) Proportion of patients achieving peak esophageal intraepithelial eosinophil count of < 15 eos/hpf (time frame: week 52)
Starting date	—
Contact information	—
Notes	—

**Table:** NCT04543409

Study name	'A study of benralizumab in patients with eosinophilic esophagitis (MESSINA)'
Methods	RCT
Participants	211 estimated participants
Interventions	Benralizumab Placebo
Outcomes	**Primary outcomes:** Proportion of patients with a histologic response at week 24, defined as a peak esophageal intraepithelial eosinophil count ≤ 6 eos/hpf (time frame: week 24) Changes from baseline in Dysphagia Symptom Questionnaire (DSQ) scores (time frame: week 24) **Secondary outcomes:** Percent change from baseline in tissue eosinophils (time frame: week 24) Change from baseline in Eosinophilic Esophagitis‐Histology Scoring System (EoE‐HSS) grade score (time frame: week 24) Change from baseline in Eosinophilic Esophagitis‐Histology Scoring System (EoE‐HSS) stage score (time frame: week 24) Changes from baseline in centrally read Endoscopic Reference Score (EREFS) (time frame: week 24) Treatment responder rate at week 24, defined as a composite of histological response (≤ 6 eos/hpf) and clinically meaningful improvement from baseline in Dysphagia Symptom Questionnaire (DSQ) scores (30% improvement) (time frame: week 24) Centrally read biopsies for additional histopathology including tissue eosinophil counts (time frame: week 24) Dysphagia‐free days as captured by the Dysphagia Symptom Questionnaire (DSQ) (time frame: week 24) Frequency of dysphagia episodes as captured by the Eosinophilic Esophagitis Daily Dysphagia Diary (EoE‐3D) (time frame: week 24) Changes from baseline in dysphagia‐associated pain, discomfort, and overall severity as captured by the EoE‐3D (time frame: week 24) Changes from baseline in abdominal pain and nausea as captured by the daily diary (time frame: week 24) Changes from baseline in PEESS (time frame: week 24) Changes from baseline in Adult Eosinophilic Esophagitis Quality of Life Questionnaire (EOE‐QoL‐A) (time frame: week 24) Change from baseline in Short Form 36‐item health survey (version 2, acute recall) (SF‐36v2) (time frame: week 24) Percent of patients with relevant concomitant procedures and healthcare resource utilization during the study through week 24 (time frame: week 24) Patient‐reported overall severity of disease as measured by Patient Global Impression of Severity (PGI‐S) (time frame: week 24) Patient‐reported change in health status since baseline as measured by Patient Global Impression of Change (PGI‐C) (time frame: week 24) **Other outcome measures:** Benralizumab pharmacokinetics (time frame: minimum of 52 weeks) Immunogenicity of benralizumab (time frame: minimum of 52 weeks) Safety and tolerability (time frame: minimum of 52 weeks)
Starting date	22 September 2020
Contact information	Marc E. Rothenberg, MD, PhD
Notes	—

**Table:** NCT04593251 2020

Study name	'Dose escalation study to evaluate an experimental new treatment (CALY‐002) in healthy subjects and subjects with celiac disease and eosinophilic esophagitis'
Methods	RCT
Participants	95
Interventions	CALY‐002
Outcomes	**Primary outcome:** Incidence of treatment‐emergent adverse events (time frame: through study completion, an average of 3 months post last dose)
Starting date	—
Contact information	—
Notes	—

**Table:** NCT04835168 2022

Study name	'Safety and pharmacokinetics of orodispersible BT‐11 in active eosinophilic esophagitis'
Methods	RCT
Participants	Withdrawn (the closure of the study was driven by the decision to redesign the study protocol for future studies)
Interventions	BT‐11 500 mg BT‐11 1000 mg
Outcomes	**Primary outcome:** Incidence and severity of adverse events (time frame: 12 weeks)
Starting date	—
Contact information	—
Notes	—

**Table:** NCT05084963 2021

Study name	'A study to assess the efficacy, safety and tolerability of IRL201104 in adults with active eosinophilic esophagitis'
Methods	RCT
Participants	36 participants
Interventions	Arm 1: IRL201104 Dose A Arm 2: IRL201104 Dose B
Outcomes	**Primary outcome:** Change from baseline in the peak esophageal intraepithelial eosinophil count at week 4 (time frame: 4 weeks) The change from baseline in histologic eosinophil count in each treatment group will be summarized as the mean, standard deviation, median, minimum, and maximum
Starting date	—
Contact information	—
Notes	—

**Table:** NCT05214599

Study name	'Pharmacokinetics, efficacy, tolerability and safety of different budesonide oral gel doses in adults' subjects of both genders with eosinophilic esophagitis (EoE) (BESIDE)'
Methods	RCT
Participants	36 estimated
Interventions	Budesonide gel low‐, medium‐ and high‐dose
Outcomes	**Primary outcomes:** Peak plasma concentration (Cmax) (time frame: first 24 hours after a single drug dose administration) Area under the plasma concentration versus time curve (AUC) (time frame: first 24 hours after a single drug dose administration) Half‐life (T1/2) (time frame: first 24 hours after a single drug dose administration) Oral clearance (CL/F) (time frame: first 24 hours after a single drug dose administration) ≤ 6 eosinophils per high‐power field (time frame: 8 weeks of treatment) Improvement in dysphagia symptoms consistent with the disease EAT (Eating Assessment Tool)‐10 questionnaire (time frame: 8 weeks of treatment) **Secondary outcomes:** Assessment of non‐serious and serious adverse events rate (time frame: through 8 weeks) Quality of life assessment of participants (time frame: through 8 weeks)
Starting date	2 September 2023
Contact information	No contacts or locations provided Bazell Pharma AG
Notes	—

**Table:** NCT05543512

Study name	'The Immune Directed Individualized Elimination Therapy (iDIET) Study (iDIET)'
Methods	RCT pilot/feasibility
Participants	100 estimated
Interventions	Participants will be randomized in a 1:1 fashion to follow an allergen‐specific immune signature‐directed diet or sham diet during the 8‐week treatment period
Outcomes	**Primary outcomes:** Post‐treatment peak eosinophil count (time frame: 8 weeks) **Secondary outcomes:** Dysphagia symptom score (time frame: 8 weeks) Endoscopic severity (time frame: 8 weeks) Percentage of histologic responders (time frame: 8 weeks) Change in peak eosinophil count (time frame: baseline and week 8)
Starting date	14 October 2022
Contact information	Evan S Dellon, MD, MPH
Notes	—

**Table:** NCT05583227

Study name	'Efficacy and safety of tezepelumab in patients with eosinophilic esophagitis (CROSSING)'
Methods	RCT
Participants	360 estimated
Interventions	Subjects will be randomized in a 1:1:1 ratio to receive either a low dose of tezepelumab, a high dose of tezepelumab, or placebo
Outcomes	**Primary outcomes:** Histologic response of peak esophageal eosinophil per HPF count of ≤ 6 across all available esophageal levels (time frame: week 24) Change from baseline in DSQ (Dysphagia Symptom Questionnaire) score (time frame: week 24) **Secondary outcomes:** Change from baseline in EoE EREFS (endoscopic reference score) (time frame: week 24, week 52) Change from baseline in EoE‐HSS (histologic scoring system) grade score (time frame: week 24) Change from baseline in EoE‐HSS (histologic scoring system) stage score (time frame: week 24) Histologic response of peak esophageal eosinophil per HPF count of ≤ 6 across all available esophageal levels (time frame: week 52) Change from baseline in DSQ (Dysphagia Symptom Questionnaire) score (time frame: week 52) Response of achieving clinico‐histological remission (time frame: week 24, week 52) **Other outcomes:** Change from baseline in peak esophageal eosinophil count (EOS/HPF) (time frame: week 24, week 52) Changes from baseline in PEESS module at week 24 (adolescents only) (time frame: week 24, week 52) Change from baseline in EoE‐HSS (histologic scoring system) stage score (time frame: week 52) Change from baseline in EoE‐HSS (histologic scoring system) grade score (time frame: week 52) Serum tezepelumab concentration (time frame: weeks 0, 4, 12, 24, and 52) Anti‐drug antibody (time frame: weeks 0, 12, 24, and 52)
Starting date	10 November 2022
Contact information	AstraZeneca Clinical Study Information Center 1‐877‐240‐9479 information.center@astrazeneca.com
Notes	—

**Table:** NCT05634746

Study name	'24‐week induction study of APT‐1011 in adult subjects with eosinophilic esophagitis (EoE) (FLUTE 3)'
Methods	RCT
Participants	200 participants estimated
Interventions	APT‐1011 3 mg Placebo
Outcomes	**Primary outcomes:** Histological remission (co‐primary outcome measure) (time frame: week 24) Complete symptomatic response (co‐primary outcome measure) (time frame: week 24) **Secondary outcomes:** Clinicopathologic responder rate (time frame: week 24) Percentage of participants with ≥ 70% reduction in dysphagia frequency (time frame: week 24) Mean change in dysphagia frequency (time frame: week 24) Mean change in PROSE difficulty swallowing (time frame: week 24) Mean change in PROSE pain with swallowing (time frame: week 24) Mean number of dysphagia‐free days (time frame: week 24) Percentage of responders (strictures and ≥ grade 2 rings) (time frame: week 24) Percentage of responders (strictures) (time frame: week 24) Percentage of responders (≥ grade 2 rings) (time frame: week 24) Mean change in EREFS (time frame: week 24) Time to first complete symptom response (time frame: week 24)
Starting date	29 December 2022
Contact information	ClinicalTrials@ellodipharma.com
Notes	—

**Table:** NCT05695456

Study name	'Targeted elimination diet in EoE patients following identification of trigger nutrients using confocal laser endomicroscopy (CLE‐EoE)'
Methods	RCT cross‐over
Participants	Estimated 25 participants
Interventions	Patients with a positive CLE reaction to one or two specific nutrients will then be randomized to a blinded exclusion diet for 6 weeks of those nutrients or to exclusion of another tested nutrient that yielded no change in CLE (= sham diet), in a cross‐over fashion. Patients with no CLE reaction will undergo an empirical exclusion diet of gluten‐containing grains for 6 weeks. To mirror the cross‐over character of the intervention, CLE negative patients will then undergo a milk exclusion diet for 6 weeks (order is interchangeable).
Outcomes	**Primary outcome:** Difference in response (time frame: 2 years) **Secondary outcomes:** Further histological outcomes (time frame: 2 years) Further histological outcomes (time frame: 2 years) Symptomatic changes in patients undergoing duodenal CLE‐targeted elimination diet compared to the sham diet (time frame: 2 years) Endoscopic changes in patients undergoing duodenal CLE‐targeted elimination diet compared to the sham diet (time frame: 2 years) Change in esophageal and duodenal permeability parameters 1 (time frame: 2 years) Change in esophageal and duodenal permeability parameters 2 (time frame: 2 years) Baseline and post‐exposure duodenal mast cell and eosinophil counts on histology (time frame: 2 years) Differences in duodenal permeability measures in CLE positive, CLE negative EoE patients and healthy controls before and after nutrient application 1 (time frame: 2 years) Differences in duodenal permeability measures in CLE positive, CLE negative EoE patients and healthy controls before and after nutrient application 2 (time frame: 2 years)
Starting date	16 February 2022 (retrospectively registered)
Contact information	Jan Tack, MD PhD +3216345514 jan.tack@kuleuven.be
Notes	—

CLE: confocal laser endomicroscopy; DB: double‐blind; DD: dysphagia days; DED: dairy elimination diet; DSD: daily symptom diary; DSQ: Dysphagia Symptom Questionnaire; EoE‐HSS: EoE histology scoring system; EoE: eosinophilic esophagitis; EOT: end of treatment; EREFS: Eosinophilic Esophagitis Endoscopic Reference Score; FREE: dairy elimination plus food additive elimination; GP: general practitioner; HPF/hpf: high‐power field; PEC: peak eosinophil count; PEESS: Pediatric Eosinophilic Esophagitis Symptom Severity; PK: pharmacokinetic; PPI: proton pump inhibitor; QOL: quality of life; RCT: randomized controlled trial; TTG: tissue transglutaminase

## Differences between protocol and review

There have been adjustments in the primary and secondary outcomes, as well as the pre‐planned subgroup and sensitivity analyses since the previous version, reflecting current knowledge on eosinophilic esophagitis.

We were not able to perform any pre‐planned subgroup or sensitivity analyses for any of the comparisons presented in summary of findings table 2 and tables 4 to 10 due to very limited data. Any pre‐planned subgroup and sensitivity analyses not listed under analyses for the comparisons corticosteroids versus placebo for induction of remission and biologics versus placebo for induction of remission, were also not performed due to lack of data.

Funnel plots to judge publication bias were only possible in one instance (Figure 3), as in all other cases there was not a sufficient number of studies (n ≥ 10).
